# Childhood stunting in relation to the pre- and postnatal environment during the first 2 years of life: The MAL-ED longitudinal birth cohort study

**DOI:** 10.1371/journal.pmed.1002408

**Published:** 2017-10-25

**Authors:** MAL-ED Network Investigators

**Affiliations:** Uppsala University, SWEDEN

## Abstract

**Background:**

Stunting is the most prevalent manifestation of childhood malnutrition. To characterize factors that contribute to stunting in resource-poor settings, we studied a priori selected biological and social factors collected longitudinally in a cohort of newborns.

**Methods and findings:**

We enrolled 1,868 children across 7 resource-poor settings in Bangladesh, Brazil, India, Nepal, Peru, South Africa, and Tanzania shortly after birth and followed them for 24 months between 2 November 2009 and 28 February 2014. We collected longitudinal anthropometry, sociodemographic factors, maternal-reported illnesses, and antibiotic use; child feeding practices; dietary intake starting at 9 months; and longitudinal blood, urine, and stool samples to investigate non-diarrheal enteropathogens, micronutrients, gut inflammation and permeability, and systemic inflammation. We categorized length-for-age *Z*-scores into 3 groups (not stunted, ≥−1; at risk, <−1 to −2; and stunted, <−2), and used multivariable ordinal logistic regression to model the cumulative odds of being in a lower length-for-age category (at risk or stunted). A total of 1,197 children with complete longitudinal data were available for analysis. The prevalence of having a length-for-age *Z*-score below −1 increased from 43% (range 37%–47% across sites) shortly after birth (mean 7.7 days post-delivery, range 0 to 17 days) to 74% (16%–96%) at 24 months. The prevalence of stunting increased 3-fold during this same time period. Factors that contributed to the odds of being in a lower length-for-age category at 24 months were lower enrollment weight-for-age (interquartile cumulative odds ratio = 1.82, 95% CI 1.49–2.23), shorter maternal height (2.38, 1.89–3.01), higher number of enteropathogens in non-diarrheal stools (1.36, 1.07–1.73), lower socioeconomic status (1.75, 1.20–2.55), and lower percent of energy from protein (1.39, 1.13–1.72). Site-specific analyses suggest that reported associations were similar across settings. While loss to follow-up and missing data are inevitable, some study sites had greater loss to follow-up and more missing data than others, which may limit the generalizability of the findings.

**Conclusions:**

Neonatal and maternal factors were early determinants of lower length-for-age, and their contribution remained important throughout the first 24 months of life, whereas the average number of enteropathogens in non-diarrheal stools, socioeconomic status, and dietary intake became increasingly important contributors by 24 months relative to neonatal and maternal factors.

## Abstract 2

Using data from the MAL-ED longitudinal birth cohort study, William Checkley and colleagues examine factors associated with childhood stunting during first two years of life in Bangladesh, Brazil, India, Nepal, Peru, South Africa and Tanzania.

## Author summary

**Why was this study done?:**

- MAL-ED (Etiology, Risk Factors, and Interactions of Enteric Infections and Malnutrition and the Consequences for Child Health and Development) is a multidisciplinary, prospective, longitudinal cohort study of the growth and development of children living at sites in southern Asia (Dhaka, Bangladesh; Vellore, India; Bhaktapur, Nepal), Latin America (Fortaleza, Brazil; Iquitos, Peru), and sub-Saharan Africa (Venda, South Africa; Haydom, Tanzania).
- The study goal was to better understand the complicated interrelationships among enteric infections, nutrition, and other environmental exposures and child growth. The overarching hypothesis of the MAL-ED study was that repeated enteropathogen infections in infants and young children affect physical growth.

**What did the researchers do and find?:**

- In our analysis of the MAL-ED study, we found that factors leading to childhood stunting were established early in life.
- Neonatal and maternal factors were found to play a more influential role than postnatal factors during early childhood, and their contributions remained significant throughout the first 24 months.
- Postnatal exposures, including a higher burden of non-diarrheal enteropathogens, lower socioeconomic status, and lower protein content of the diet became increasingly important contributors with age relative to neonatal and maternal factors.

**What do these findings mean?:**

- Maternal interventions, especially during pregnancy, are likely to have intergenerational effects and a lasting impact on birthweight and child growth outcomes.
- Initiatives to address childhood stunting should also consider improvements to the composition of complementary foods (i.e., higher protein) and strategies to reduce gut pathogen exposure.

## Introduction

**Fig 1:**
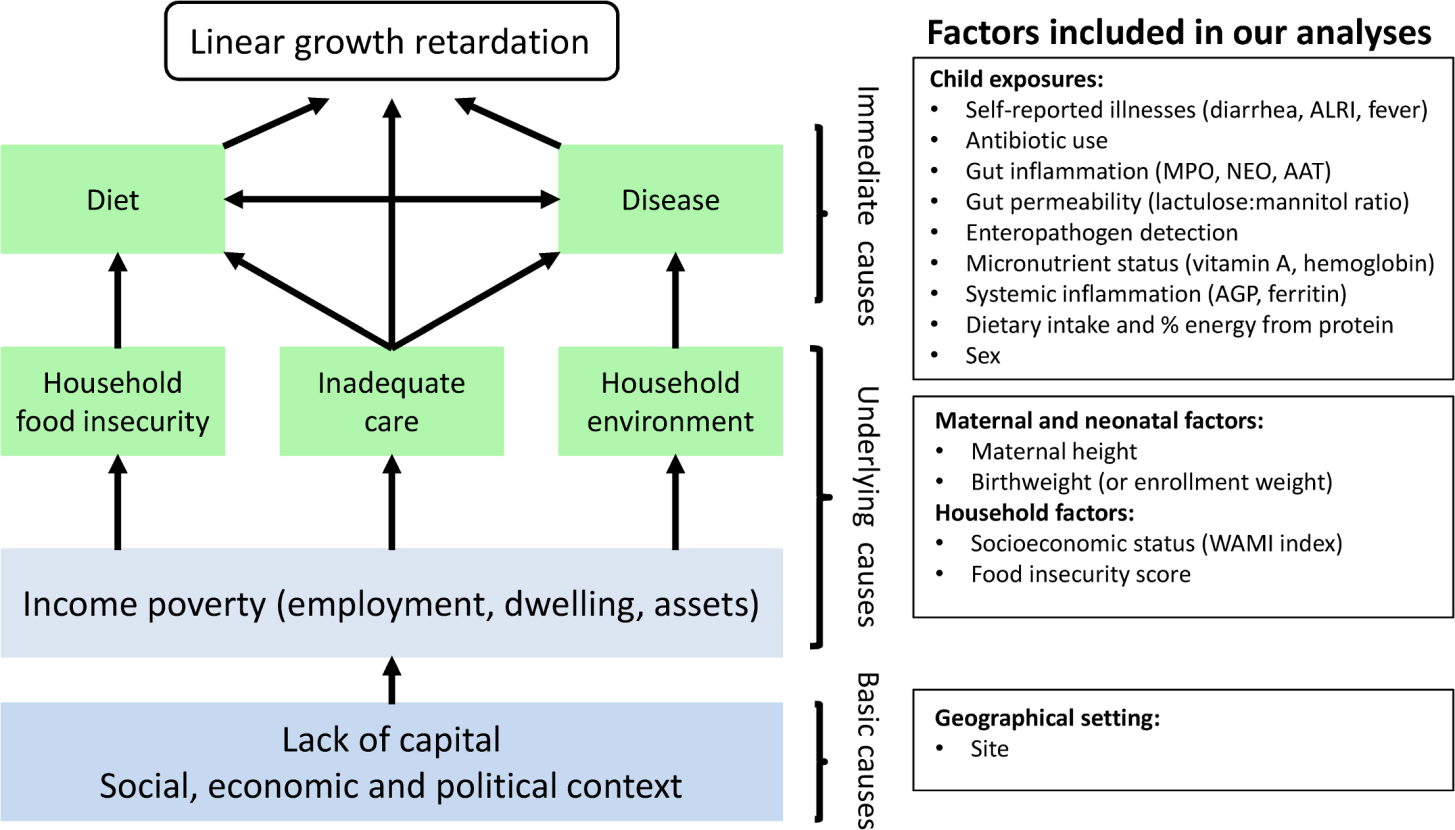
Modified version of the UNICEF malnutrition conceptual hierarchical framework and the maternal and household factors and childhood environmental exposures included in our analyses. AAT, alpha-1 antitrypsin; AGP, alpha-1-acid glycoprotein; ALRI, acute lower respiratory infection; MPO, myeloperoxidase; NEO, neopterin.

Stunting is the most prevalent condition of child malnutrition worldwide [1], and it is associated with negative health and economic outcomes later in life, including shorter adult height, less schooling, and reduced adult income [2]. An estimated 165 million children younger than 5 years old are stunted [1], and 90% of these children live in 36 countries, mostly in Asia and Africa [3]. Stunting typically begins in utero and usually reflects persistent, cumulative effects of poor nutrition and other deficits that often span across several generations [4,5]. Determinants of stunting may be affected by distal factors, such as geopolitics and economics, and proximal factors, such as inadequate diet and endemic disease [1]. Analyses of the World Health Organization Global Database on Child Growth and Malnutrition found that length-for-age starts close to the standard but falters dramatically in the first 2 years of life consistently across Asia, Africa, and Latin America, and this trend appears to have remained unchanged for decades [6,7]. Therefore, a comprehensive characterization of what occurs during this critical period, when substantial brain and cognitive function are also developing, is an urgent global priority. Potential contributors to growth shortfalls such as enteric infections and food intake warrant careful study to identify potentially effective interventions. It is precisely a better understanding of these risk factors in this critical formative window of early childhood that has been the focus of the MAL-ED (Etiology, Risk Factors, and Interactions of Enteric Infections and Malnutrition and the Consequences for Child Health and Development) study, a multi-center study aimed at evaluating risk factors for growth faltering and associated health outcomes in children. Here we sought to evaluate how select neonatal, maternal, and postnatal factors, which represent different hierarchical levels depicted in the UNICEF malnutrition framework [1,8], contribute to low length-for-age in the first 2 years of life (Fig 1).

## Methods

### Ethics

Ethics approval was obtained from the institutional review boards at participating institutions (S1 Table). Written informed consent was obtained from the parent or guardian of each participating child. This study is reported as per STROBE guidelines (S1 STROBE Checklist).

### Study design and participants

The MAL-ED study is a longitudinal study of the role of enteric infections in health outcomes in children followed from birth to age 24 months living in resource-poor settings across diverse geographical areas. The study design has been described in detail elsewhere (S1 Text) [9]. This study was conducted from 2 November 2009 to 28 February 2014 at 8 sites on 3 continents [10]: Dhaka, Bangladesh (BGD), Vellore, India (INV), Bhaktapur, Nepal (NEB), and Naushahro Feroze, Pakistan (PKN), in Asia; Fortaleza, Brazil (BRF), and Loreto, Peru (PEL), in the Americas; and Venda, South Africa (SAV), and Haydom, Tanzania (TZH), in Africa. A baseline census was conducted at each site to assess the number of pregnant women whose offspring would be available for inclusion.

Enrollment took place over a 2-year period with the goal of enrolling 200 children per site. While this sample size was established as the maximum number of children who could be followed intensively at each site, we tested the power of the study a priori under varying rates of enteropathogen detection, proportions of incident malnutrition outcomes at 24 months, and effect sizes. Under the assumption that 8% of children would be malnourished at birth, the effective sample size was estimated at 184 children per site, or 1,472 children for all sites. Therefore, if 40% of the children became malnourished (i.e., a weight-for-age *Z*-score < −2 or height-for-age *Z*-score < −2) at the end of 2 years and if an enteropathogen infection affected 50% of the children over 2 years, we would have 60% power for a single site and 100% power for all sites combined to detect a 50% higher risk of being malnourished with 95% confidence. The power of the study to detect a 70% increase in malnutrition risk associated with an infection (all other assumptions being the same) was 82% for a single site and 100% for all sites combined. Additional details are provided elsewhere (S2 Text).

All children were enrolled within 17 days of birth and followed uniformly for 24 months [11–14]. Exclusion criteria for participation were the following: the family planned to move outside the community in the next 6 months or planned an absence from the study area of greater than 30 consecutive days, maternal age < 16 years, the mother had another child in the MAL-ED study, multiple pregnancy, severe disease in the infant requiring hospitalization for something other than typical healthy birth, severe or chronic condition in the infant diagnosed by medical doctor (e.g., neonatal disease, renal disease, chronic heart failure, liver disease, cystic fibrosis, congenital conditions), birthweight < 1,500 g, enteropathies in the infant diagnosed by medical doctor, and mother unable to provide informed consent.

For this analysis, we excluded data from PKN because quality assurance procedures identified unexplained bias in a subset of length measurements. We also excluded children from our analysis who did not meet minimal criteria for longitudinal follow-up (S3 Text; S2 Table).

Below, we provide a summary of the factors included in our analysis. We provide additional details for each factor in S3 Table and the proportion of missing data in S1 Fig.

### Neonatal and maternal factors

We used enrollment weight (measured ≤17 days after birth) standardized into a *Z*-score using the WHO international growth standard [11] as a surrogate measure for birthweight, and maternal height as a surrogate measure for intergenerational growth [15].

### Household factors

Mothers or caregivers were questioned every 6 months about their sources of water and sanitation facilities, assets, income, and food security. Level of maternal education was collected in the baseline survey. We combined water and sanitation, assets, maternal education, and income into a composite score (WAMI index) [16], and calculated a food insecurity score as previously described [17]. As the WAMI index (S2 Fig) and food insecurity score (S3 Fig) showed small variation over time, we averaged their values across all collected time points (S4 Table).

### Illness factors

At twice weekly home visits, daily information on child illnesses and antibiotic use was collected from the mother. We evaluated 3 common illnesses—diarrhea, acute lower respiratory infection (ALRI), and fever—and, as detailed elsewhere, used standard definitions of illness onset and episodes [13]. We calculated the longitudinal prevalence of each of these as the cumulative number of days ill (or days with antibiotic use) divided by the total number of follow-up days. We also recorded the cumulative number of instances when a child was diagnosed with dehydrating diarrhea or pneumonia by a physician or healthcare provider, as self-reported by mothers after referrals made study staff or self-referrals to a medical center (S3 Table).

### Microbiology

Field workers collected monthly (during the first year of life) and quarterly (during the second year of life) non-diarrheal stool samples. A non-diarrheal stool sample was considered as any specimen collected 2 or more days apart from an episode of diarrhea. As previously described, these specimens were tested for ≥40 enteropathogens [11]. We calculated the average number of enteropathogens detected in non-diarrheal stool divided by the total number of stool samples tested. We assumed that cumulative non-diarrheal enteropathogen load was zero at enrollment, and we carried forward cumulative values for months with missing data.

### Infant feeding practices and dietary intake

Initiation of breastfeeding was recorded and surveillance of infant and young child feeding practices was undertaken by teams of field workers who visited the homes of participating children twice weekly. Days between twice weekly visits were assumed to have the same status as the preceding visit for the calculation of duration of each breastfeeding practice in days, as previously described [18]. We defined breastfeeding status as exclusive if the child received only breast milk with the exception of vitamins or medicine; as predominant if a child received water or water-based liquids such as juice or tea in addition to breast milk; as partial if the child received milk-based liquids or semisolid or solid food in addition to breast milk; or as none if there was no consumption of breast milk. For the purpose of this analysis, however, we only used information regarding the total proportion of days that the child was breastfed.

Additionally, starting at 9 months, field workers conducted 24-hour complementary food intake recall interviews once monthly; this information was linked to country-specific nutritional databases to quantify energy, macronutrient, and micronutrient intakes from non-breast-milk foods. Dietary intake over time was characterized by integrating these multiple types of data (e.g., cumulative proportion of days that a child was breastfed, cumulative energy intake [per 1,000 kilocalories], and average percent of energy from protein from non-breast-milk foods consumed on 1 day per month).

### Micronutrient status, systemic inflammation, and gut inflammation and permeability

Longitudinal visits were conducted to collect blood (at 7, 15, and 24 months), urine (at 3, 6, 9, and 15 months), and monthly or quarterly non-diarrheal stool samples (as specified above) for the assessment of micronutrient status, systemic inflammation, and gut inflammation and permeability markers. From blood, we evaluated concentrations of altitude-adjusted hemoglobin, inflammation-adjusted retinol, alpha-1-acid glycoprotein (AGP), and inflammation-adjusted plasma ferritin by averaging the values of the 3 measurements obtained during the study period. If a child had only 2 measurements, we averaged the 2 values; if only 1 measurement, we used that value alone. From non-diarrheal stools, we measured myeloperoxidase (MPO), neopterin (NEO), and alpha-1 antitrypsin (AAT) concentrations as previously described [19]. These gut inflammation biomarkers were measured monthly for the first 12 months and quarterly for the second 12 months; we calculated quarterly averages for the first year and used the collected quarterly measurements thereafter. From urine, we evaluated the lactulose:mannitol ratio as a measure of gut permeability [19]. We assigned the value collected at 3 months to the interval birth to 5 months, the 6-month value to the interval 6 to 8 months, the 9-month value to the interval 9 to 14 months, and the 15-month value to the interval 15 to 24 months.

### Outcome

Children were visited monthly to measure length and weight. We measured length with 3 different types of commercially made measuring boards to the nearest 0.1 cm and weight with 2 different types of scales to the nearest 10 g. We used 2 staff members at all times to conduct these assessments. A total of 152 field workers (range 7 to 36 per site) were standardized in the measurement of anthropometry by local investigators using a common protocol. To ensure quality control, extreme measurements were investigated, and secondary measurements were collected within 24 h for approximately 5% of all metrics. Reliability estimates for both weights and lengths were >0.9. Details of equipment, anthropometric assessments, and quality control procedures are found in S1 Text.

The main outcome variable was monthly length-for-age *Z*-score categorized into 3 groups: −1 SD or greater (not stunted), less than −1 SD to −2 SD (at risk), and less than −2 SD (stunted). We chose to stratify length-for-age into 3 categories because children who have a length-for-age *Z*-score from less than −1 SD to −2 SD, while technically not considered stunted, have an increased risk of death, especially from diarrhea and pneumonia, compared to those with *Z*-score ≥ −1 SD [1]. This categorization allowed us to have an ordinal outcome and evaluate the odds of being in a lower (at risk or stunted) length-for-age category [20]. We also examined longitudinal trends in length-for-age *Z*-scores by site.

### Biostatistical methods

We used a longitudinal multivariable ordinal logistic regression [20] to model the cumulative odds of being in a lower length-for-age category as a function of site, sex, and age, and a priori selected 22 basic, underlying, and immediate biological and social factors collected in the MAL-ED study (S3 Table). All factors except for sex and site were included as continuous variables. Stool concentrations of gut inflammation biomarkers were square-root-transformed. We modeled age using a linear piecewise spline connected at 6, 12, and 18 months to allow for nonlinear trajectories of the cumulative odds with age, and included interactions with all risk factors, site, and sex. To account for multiple measurements per child, we calculated robust standard errors [21]. Additional details about the regression model specification are presented elsewhere (S4 Text).

Results of the regression model are presented as the adjusted cumulative odds ratios (ORs) of being in a lower length-for-age category for the risk factor of interest. In this analysis, adjusted cumulative ORs for the population represent age-specific associations between each risk factor and being in a lower length-for-age category. We estimated interquartile cumulative ORs for continuous variables, i.e., the ratio of odds between the 25th and 75th percentiles or between the 75th and 25th percentiles of the risk factor, depending on the direction that indicated worse exposure. In subset analyses, we ran site-specific regressions to describe heterogeneity in ORs across sites.

An assumption of ordinal logistic regression is that the cumulative log odds are proportional among the different length-for-age thresholds. To graphically assess proportionality of odds, we compared the ORs and corresponding 95% confidence intervals for each factor obtained from a logistic regression model evaluated at each of the threshold points in length-for-age (−1 SD and −2 SD). There was substantial overlap in the 95% confidence intervals for each factor between threshold points, and departures from proportionality did not substantively impact the relative importance or factor-specific effects (S5 Text; S4 Fig). Goodness of fit was then visually assessed as the overlap between the observed and model-predicted probabilities. We calculated 2 standard error bands for the observed probabilities using standard methods, and a 95% confidence interval using 2,000 bootstrap replications in which children were chosen with replacement. We found good concordance between observed and predicted probabilities (S5 Text; S5 Fig). We conducted a sensitivity analysis using all data available instead of limiting our analysis to the data of children who met minimal criteria for longitudinal follow-up, and found similar results (S6 Fig). Additional details of variable construction, sample selection and missing data, model specification, testing of the proportionality of odds assumption, and goodness of fit are found elsewhere (S4 and S5 Texts; S3 Table). We conducted statistical analyses in R (https://www.r-project.org, Foundation for Statistical Computing, Vienna, Austria).

Our analysis followed our proposed analytical plan but for some minor changes (S6 Text). We originally planned to use birthweight in our analysis, but birthweight had a high proportion of missing data. Furthermore, birthweight was self-reported, in contrast to enrollment weight, which was collected by our group. Other refinements to our analysis that were not in our original analysis plan but were included in a revised plan after investigator meetings were the use of age by risk factor interaction terms, better differentiation between biomarkers of systemic inflammation and biomarkers of gut enteropathy, and limiting analysis of dietary intake data to total energy intake and average percent of energy from protein from non-breast-milk foods.

## Results

### Participant characteristics

A total of 1,868 children were enrolled across the 7 sites, of whom 11 died, 269 moved away, 87 were lost to follow-up, and 210 did not meet minimal criteria for longitudinal follow-up, leaving 1,291 (69%) children for our analysis. Of these, 1,197 children had complete data for all risk factors analyzed here, contributing 23,629 monthly observations. Factors under study exhibited considerable heterogeneity across sites (Table 1).

**Table 1:** Characteristics of risk factors for being at risk or stunted length-for-age by site.

Characteristic	Asia			Americas		Africa	
	BGD	INV	NEB	BRF	PEL	SAV	TZH
**Sample size at enrollment, *n***	265	251	240	233	303	314	262
**Sample size of children with complete data, *n* (percent)**	206 (78)	200 (80)	208 (87)	81 (35)	198 (65)	199 (63)	199 (76)
**Neonatal and maternal factors**							
Enrollment weight-for-age *Z*-score, mean (SD)	−1.3 (0.9)	−1.3 (1.0)	−0.9 (1.0)	−0.2 (1.1)	−0.6 (0.9)	−0.4 (1.0)	−0.1 (0.9)
Maternal height in cm, mean (SD)	148.9 (5.1)	151.2 (5.0)	149.6 (5.3)	154.9 (6.8)	149.8 (5.6)	158.7 (6.8)	155.8 (6.0)
**Household factors**							
WAMI index, mean (SD)	0.5 (0.1)	0.5 (0.1)	0.7 (0.1)	0.8 (0.1)	0.5 (0.1)	0.8 (0.1)	0.2 (0.1)
Food insecurity score, mean (SD)	1.3 (2.3)	2.0 (3.6)	0.8 (1.6)	10.7 (3.5)	7.1 (3.9)	3.9 (3.6)	1.7 (1.8)
**Maternal-reported illnesses/antibiotic use at 24 months**							
Diarrhea: longitudinal prevalence (percent days)	3.5	1.8	3.1	0.6	4.7	0.4	0.8
ALRI: longitudinal prevalence (percent days)	0.3	2.2	0.8	0.1	0.4	0.3	0.1
Fever: longitudinal prevalence (percent days)	6.3	5.5	4.7	0.5	4.0	0.5	2.0
Antibiotic use: longitudinal (percent days)	14.9	4.7	3.7	1.4	6.7	2.0	6.6
Diarrhea: number of diagnoses, mean (SD)	0.3 (1.1)	1.0 (0.6)	0.5 (3.0)	0 −	0.2 (1.2)	0.1 (1.0)	0.7 (2.4)
Pneumonia: number of diagnoses, mean (SD)	1.5 (5.0)	0.8 (2.7)	1.4 (3.7)	0.3 (1.6)	0.6 (2.7)	0.2 (1.3)	0.6 (2.0)
**Cumulative average number of non-diarrheal enteropathogens at 24 months**	1.1 (0.3)	1.1 (0.4)	0.8 (0.3)	1.2 (0.4)	1.0 (0.4)	0.6 (0.2)	1.3 (0.4)
**Micronutrient status and inflammation**							
Hemoglobin, g/l, mean (SD)	114 (12)	108 (10)	105 (09)	112 (14)	110 (09)	110 (11)	111 (11)
Percent with anemia (hemoglobin < 100 g/l) at 24 months	27%	42%	38%	26%	26%	42%	34%
Retinol concentration, mcg/dl, mean (SD)	23.7 (5.3)	29.4 (7.2)	25.7 (5.2)	31.1 (7.7)	23.1 (4.9)	20.2 (6.2)	16.8 (5.0)
Percent with vitamin A deficiency (retinol < 20 mcg/dl) at 24 months	25%	13%	26%	12%	30%	57%	68%
Alpha-1-acid glycoprotein concentration, mg/dl, mean (SD)	87.0 (22.6)	98.6 (24.0)	122.3 (30.2)	96.0 (24.8)	116.7 (29.2)	130.4 (37.9)	118.8 (39.3)
Plasma ferritin concentration, mcg/l, mean (SD)	22.4 (19.7)	22.6 (21.2)	23.3 (60.4)	28.2 (11.7)	32.0 (26.4)	31.2 (31.2)	19.7 (18.3)
**Breastfeeding over 24 months**							
Days with exclusive breastfeeding (mean)	103.5	75.8	58.8	63.7	39.4	28.7	44.8
Days with partial breastfeeding (mean)	528.0	402.1	585.2	396.2	409.4	477.4	504.3
**Non-breast-milk intake over 24 months**							
Non-breast-milk cumulative energy intake, per 1,000 kCal, mean (SD)	349.3 (135.3)	753.1 (224.0)	435.3 (155.7)	1,005.3 (203.3)	738.0 (178.0)	880.6 (196.8)	993.8 (191.6)
Non-breast-milk percent energy from protein, mean (SD)	11.4 (1.4)	11.3 (1.4)	11.2 (2.0)	16.8 (1.7)	10.8 (1.5)	12.2 (1.4)	11.8 (1.3)
**Gut inflammation and permeability biomarkers**							
Stool MPO, ng/ml (square root) in first 12 months, mean (SD)	85.7 (63.3)	119.2 (64.7)	88.1 (48.7)	65.6 (44.4)	106.7 (55.1)	81.7 (43.6)	97.8 (54.5)
Stool NEO, nmol/l (square root) in first 12 months, mean (SD)	41.8 (22.0)	50.6 (21.1)	46.6 (15.9)	43.1 (21.1)	55.9 (19.8)	69.9 (30.7)	38.2 (26.7)
Stool AAT, mg/g (square root), in first 12 months, mean (SD)	0.7 (0.3)	0.7 (0.3)	0.8 (0.3)	0.6 (0.3)	0.7 (0.3)	0.6 (0.3)	0.6 (0.3)
Lactulose:mannitol *Z*-score at 15 months, mean (SD)	−0.0 (0.7)	0.9 (0.8)	−0.1 (1.1)	−0.0 (1.1)	1.0 (0.7)	0.6 (1.4)	0.3 (1.2)

AAT, alpha-1 antitrypsin; ALRI, acute lower respiratory infection; BGD, Dhaka, Bangladesh; BRF, Fortaleza, Brazil; INV, Vellore, India; MPO, myeloperoxidase; NEB, Bhaktapur, Nepal; NEO, neopterin; PEL, Loreto, Peru; SAV, Venda, South Africa; TZH, Haydom, Tanzania.

### Longitudinal trends in length-for-age

**Fig 2:**
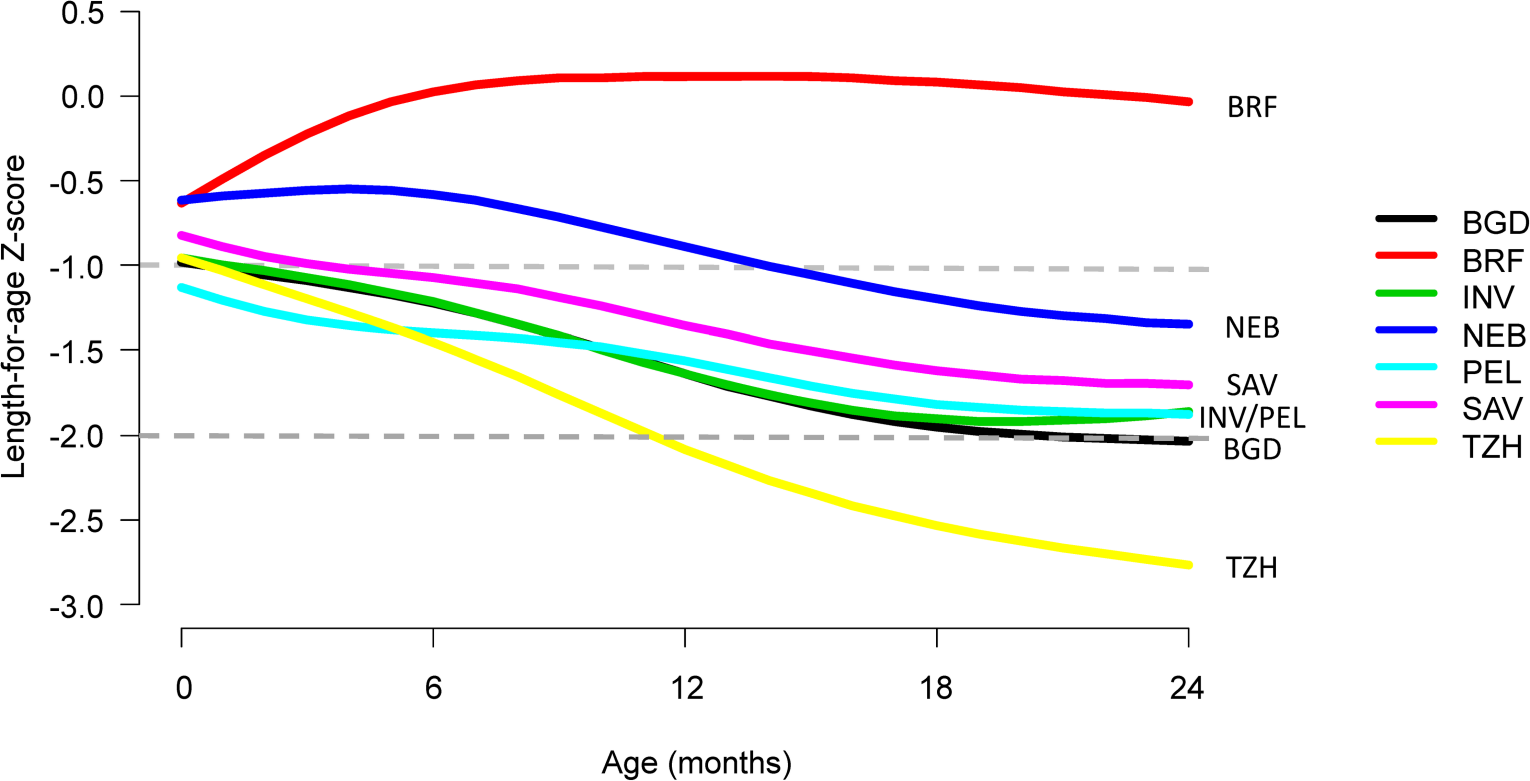
Length-for-age stratified by age and site. Site-specific length-for-age trajectories with age were smoothed using a smoothing spline. Sites include BGD, Dhaka, Bangladesh; BRF, Fortaleza, Brazil; INV, Vellore, India; NEB, Bhaktapur, Nepal; PEL, Loreto, Peru; SAV, Venda, South Africa; TZH, Haydom, Tanzania.

**Fig 3:**
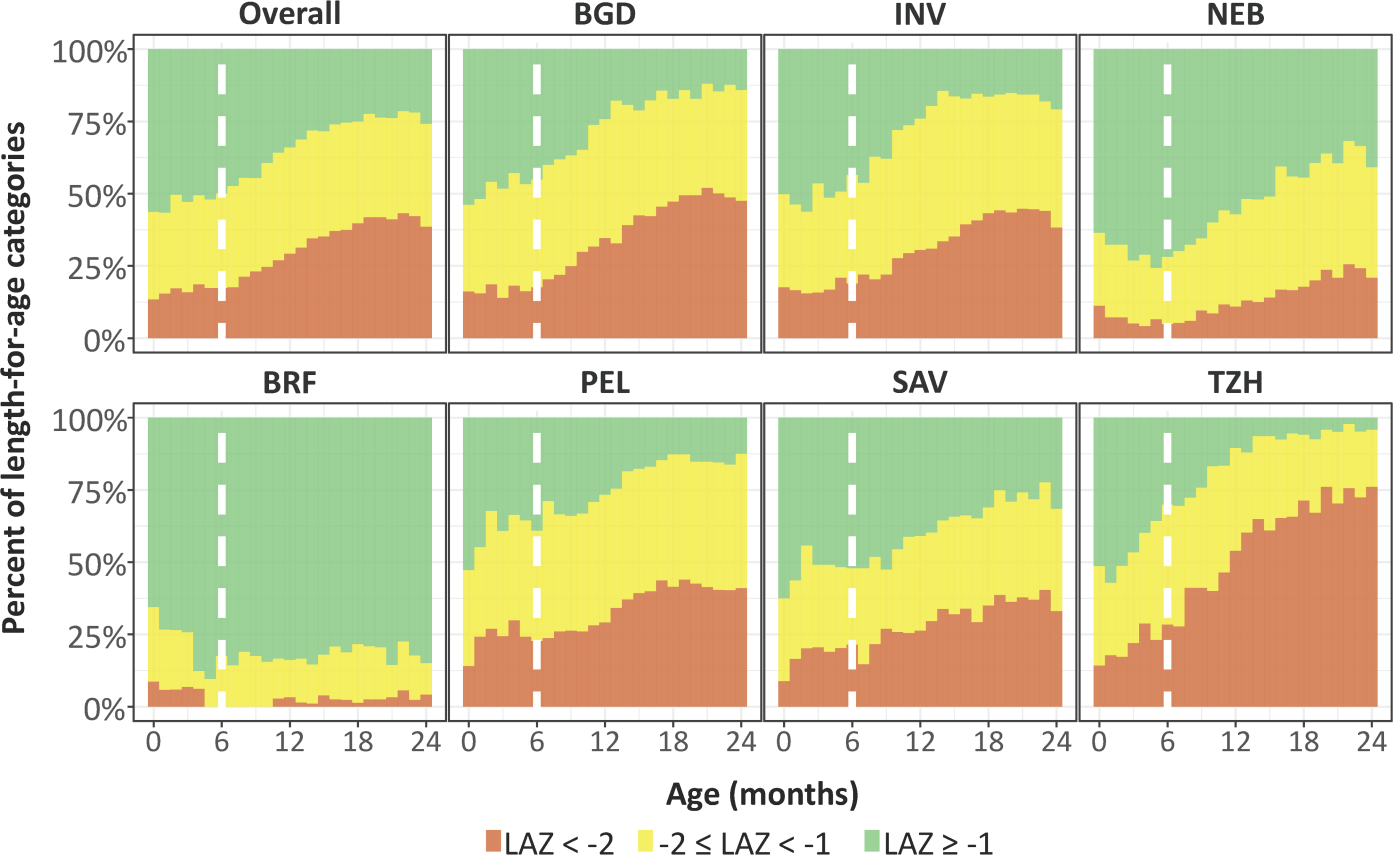
Categories of length-for-age stratified by exact month of age and site. In this figure, not stunted (length-for-age *Z*-score [LAZ] ≥ −1) is represented in green, at risk of being stunted (−2 ≤ LAZ < −1) is represented in yellow, and stunted (LAZ < −2) is represented in orange. Sites include BGD, Dhaka, Bangladesh; BRF, Fortaleza, Brazil; INV, Vellore, India; NEB, Bhaktapur, Nepal; PEL, Loreto, Peru; SAV, Venda, South Africa; TZH, Haydom, Tanzania. The vertical broken line represents 6 months of age.

First, we compared trajectories in length-for-age across 7 sites and saw an almost uniform decrease in length-for-age with age except in BRF (Fig 2). The average rate of decrease (excluding BRF) was 0.29 SD every 6 months (range from 0.19 SD in PEL to 0.49 SD in TZH). Then, we plotted the proportions of length-for-age categories by age (Fig 3).

The prevalence of stunting at enrollment was 13% (range of 10% in BRF and SAV to 16% in BGD and TZH), and it tripled to 41% by 24 months (range of 4% in BRF to 72% in TZH), with the greatest increases occurring after 6 months of age. The prevalence of having a length-for-age *Z*-score of <−1 to −2 was 30% at enrollment (range of 27% in BRF and NEB to 33% in PEL) and 37% at age 24 months (range of 13% in BRF to 46% in PEL). With the exception of BRF, which had minimal stunting, age patterns were the same; however, the magnitude was different among sites.

### Risk factors associated with being in a lower length-for-age category in the first 24 months of life

**Fig 4:**
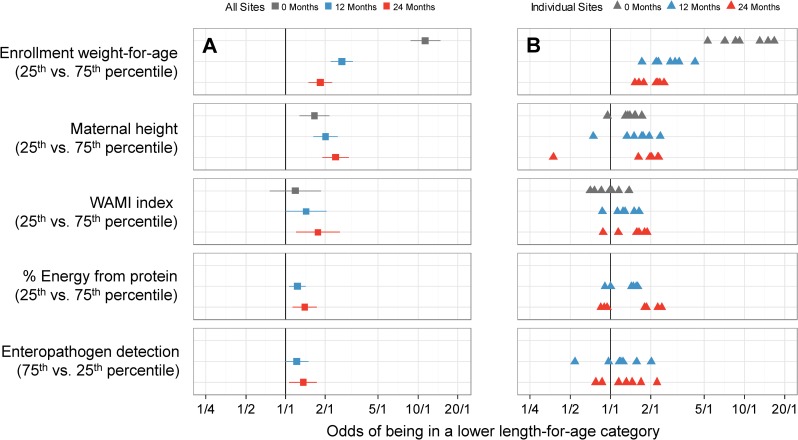
Interquartile cumulative odds ratios of being in a lower length-for-age category (at risk or stunted) at enrollment, 12 months, and 24 months for 5 risk factors, as obtained from the multivariable ordinal logistic regression model. In (A), we show adjusted interquartile cumulative odds ratios and corresponding 95% CIs. The interquartile cumulative odds ratio is calculated for the 75% and 25% percentiles of the risk factor. In (B), we show site-specific estimates represented by triangles.

We present single variable analyses of risk factors in S5 Table. In multivariable analysis, a child had greater odds of being in a lower length-for-age category (at risk or stunted) if he or she had a lower enrollment weight-for-age, was born to a shorter mother, had a higher average number of enteropathogens detected in non-diarrheal stools, lived in a household with lower socioeconomic status (SES) (WAMI index), or had consumed a lower percent of energy from protein. We plotted the adjusted cumulative ORs of being in a lower length-for age category at enrollment and 12 and 24 months for each of these 5 factors for the overall population (Fig 4A) and for each site (Fig 4B). The effect size increased with age for all factors except enrollment weight-for-age. At 24 months, the odds of being in a lower length-for-age category were greater for a lower enrollment weight-for-age (interquartile cumulative OR = 1.82, 95% CI 1.49–2.23), shorter maternal height (2.38, 1.89–3.01), higher number of non-diarrheal enteropathogens detected (1.36, 1.07–1.73), lower SES (1.75, 1.20–2.55), and lower percent of energy from protein (1.39, 1.13–1.72).

**Fig 5:**
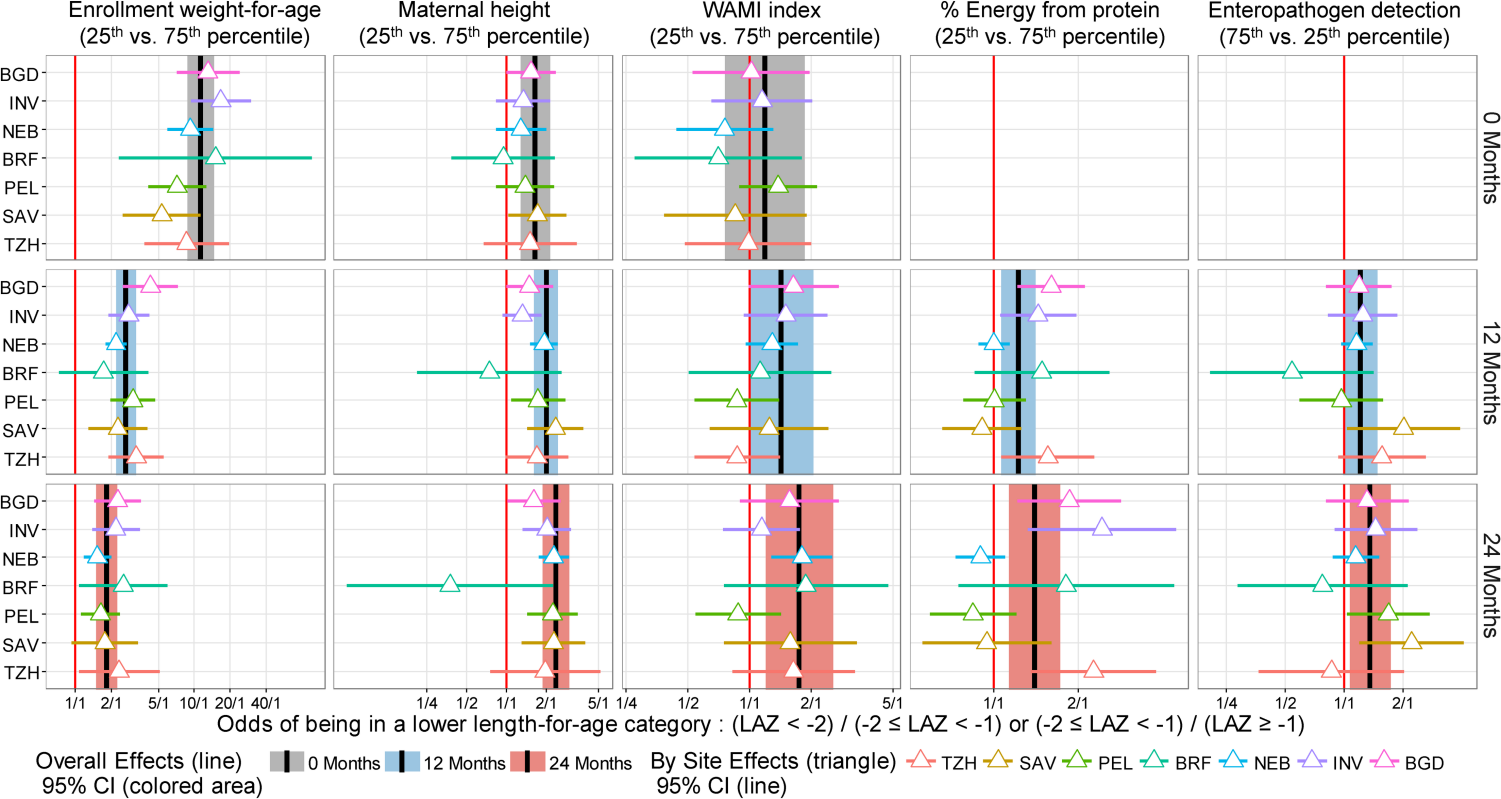
Site-specific estimates of the interquartile cumulative odds ratios of being in a lower length-for-age category (at risk or stunted) at enrollment, 12 months, and 24 months for 5 risk factors, as obtained from the multivariable ordinal logistic regression model. We contrast site-specific adjusted interquartile cumulative odds ratios and corresponding 95% CIs to the overall population interquartile cumulative odds ratios and 95% CIs. Overall interquartile cumulative odds ratios and corresponding 95% CIs at enrollment, 12 months, and 24 months are represented by black lines and grey (enrollment), blue (12 months), and red (24 months) shading. Site-specific interquartile cumulative odds ratios and corresponding 95% CIs are represented by triangles and horizontal bars. Overlap between the 95% CIs of site-specific and overall population interquartile cumulative odds ratios suggests that the associations were relatively similar across settings. BGD, Dhaka, Bangladesh; BRF, Fortaleza, Brazil; INV, Vellore, India; LAZ, length-for-age *Z*-score; NEB, Bhaktapur, Nepal; PEL, Loreto, Peru; SAV, Venda, South Africa; TZH, Haydom, Tanzania.

Adjusted interquartile cumulative ORs by site were mostly in the same direction for the majority of risk factors, especially for low enrollment weight-for-age and maternal height, with some exceptions. To assess heterogeneity, we estimated site-specific interquartile cumulative ORs and contrasted these results against those obtained in the overall population (Fig 5). While we noted great variability among site-specific estimates within each risk factor, these estimates were generally in the same direction and similar magnitude, with substantial overlap of the 95% confidence intervals between settings and the overall population. For example, while there was a 4-fold difference in the interquartile OR for maternal height between BRF and other sites or the overall population, the 95% confidence intervals for BRF were wide, which is reflective of the smaller sample size (*n* = 81) contributed by this site to the analysis.

**Fig 6:**
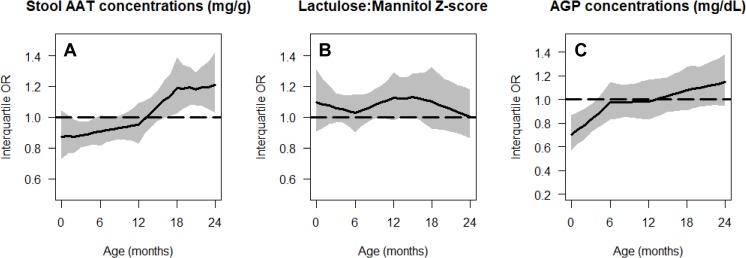
Age-specific cumulative odds ratios of being in a lower length-for-age category (at risk or stunted) for gut inflammation and permeability biomarkers. Stool AAT concentration (A), lactulose:mannitol *Z*-score (B), and AGP concentration (C). In all panels, we show the interquartile cumulative odds ratio (75th percentile versus 25th percentile) for each risk factor as a function of age. The black line represents the mean estimate, and the grey shading represents the 95% pointwise confidence interval. AAT, alpha-1 antitrypsin; AGP, alpha-1-acid glycoprotein; OR, odds ratio.

None of the maternal-reported illnesses analyzed were associated with lower length-for-age. These included the cumulative prevalences of diarrhea (*p =* 0.54), ALRI (*p =* 0.87), and fever (*p =* 0.28). Nor were 2 of the gut inflammation biomarkers, namely stool MPO (*p =* 0.77) and NEO concentrations (*p =* 0.07), associated with lower length-for-age. Higher AAT and AGP concentrations were associated with being in a lower length-for-age category at younger ages, but the direction of this association changed at older ages (Fig 6). A higher lactulose:mannitol *Z*-score was associated with being in a lower length-for-age category across all ages, but this increase was not statistically significant.

## Discussion

In the 7 resource-poor sites included in this analysis, children experienced length-for-age shortfalls relative to the WHO reference as they aged. Despite diverse cultures and geography, children at all sites (except Brazil) had linear growth faltering during childhood, with the prevalence of stunting reaching a plateau by 2 years, as per a previously reported observation [22]. Specifically, 74% of children were more than 1 SD below the WHO length standard by their second birthday, up from 43% at enrollment. Five factors contributed to being in a lower length-for-age category (at risk or stunted) during early childhood: lower enrollment weight, shorter maternal height, higher prevalence of enteropathogen detection, lower SES (WAMI index), and consumption of a lower percent of energy from protein in non-breast-milk foods. Neonatal and maternal factors were early determinants of childhood stunting, whereas 3 postnatal factors became increasingly important by 24 months.

Lower enrollment weight, a surrogate for lower birthweight, was associated with having a lower length-for-age during the first 24 months of life. While its relative contribution diminished with age, enrollment weight remained significantly associated with lower length-for-age at 24 months, i.e., these children never caught up. This finding is consistent with a meta-analysis conducted among 19 studies and 44,374 children less than 5 years of age that showed that low birthweight was associated with 3-fold higher odds of stunting [23]. Overall, 15%–20% of all births worldwide are of low birthweight, representing more than 20 million births [24].

Short maternal height is a key indicator for identifying populations at high risk for low birthweight [25]. As evidenced here, short maternal height was another factor associated with length-for-age shortfalls in their offspring, which points to the importance of intergenerational effects. Intergenerational improvements in height are achievable when adolescent girls and women of reproductive age are given adequate healthcare and nutrition, when children receive appropriate breastfeeding and complementary feeding practices during early childhood, and when children overall have better access to health care [26]. Nutritional improvement during pregnancy and early childhood can result in an increased mean height of 8 cm in just 1 generation [27].

Of the accumulated postnatal insults that we measured, the presence of enteropathogens in the absence of diarrhea, a lower SES, and a lower percent of energy from protein were all strongly associated with lower length-for-age, and the magnitude of their effects became stronger with age. The importance of protein in the diet for growth of children has recently been reported in an analysis of 2 other large cohort studies [28]. In this study, we quantified intakes from non-breast-milk foods only from 9 months of age onward. We did not attempt to quantify nutrient intakes from breast milk. While breast milk contributes energy, macronutrients, and micronutrients to the diet and children who are breastfed on average have lower intakes of non-breast-milk foods at any given age, there was a lot of overlap in intake of non-breast-milk foods at any age. As children age from 9 to 24 months, the energy from complementary food increases while the difference in average intake of complementary foods by breastfeeding status diminishes, as would be expected. Moreover, we used percent energy from protein in our statistical analyses. This percent was quantified from non-breast-milk foods, and it is a way of looking at protein density of the diet irrespective of the amount of food or energy intake. We thought that it would be problematic to impute total nutrient intakes by adding in published values of nutrient intakes from breast milk across published studies. Therefore, we did not take that approach. Rather, we adjusted for exclusive breastfeeding status.

In our analysis, we also focused on the accumulation of multiple enteropathogens—rather than on specific enteropathogens in non-diarrheal or diarrheal stools—and its association with lower length-for-age, because of its value as a surrogate measure of fecal contamination in the environment. Frequent enteropathogenic infections have a high metabolic cost. It has been estimated that infants and young children need to produce and secrete 3 to 5 g of secretory IgA into the gut per day to control enteropathogenic infections [29]. Prior studies support the hypothesis that asymptomatic enteric infections can result in adverse growth outcomes [30–32]. A high prevalence of non-diarrheal enteropathogenic infections may impact growth trajectories by exerting a continuous, negative pressure on the gut. However, neither gut inflammation nor permeability biomarkers clearly explained lower length-for-age during childhood across our study settings. For stool AAT and blood AGP concentrations, the association with lower length-for-age appeared to vary by age, suggesting that gut and systemic inflammation may be protective against growth faltering early in childhood, but deleterious later in childhood. Our analysis highlights the importance of carefully examining longitudinal trajectories in gut biomarkers and their association with growth outcomes [33]. High exposure to enteropathogens may also affect the gut microbiome, a factor that has become increasingly recognized as an important determinant of health, growth, and disease [34].

Illnesses reported by mothers were also not associated with being in a lower length-for-age category. The lack of association with diarrheal illness, when analyzed individually or alongside other risk factors, is surprising as diarrhea has historically been reported to be associated with adverse growth outcomes [22,35–38]. However, 50 years have passed since the first reports alerted public health practitioners and governments to the negative health consequences of diarrhea. For the same reasons that diarrhea-related mortality, but not morbidity have decreased, it is possible that increased awareness and education through public health interventions, coupled with better access to improved water and sanitation and early care and treatment, may have led to a shift toward less severe disease and may help to explain differences in findings between our study and previous work. There are other potential alternative explanations. We included a large number of factors in our analysis, and some of the included factors may be collinear with self-reported diarrhea. However, in single variable analysis we also did not find diarrhea alone to be associated with a being in a lower length-for-age category.

Our finding that lower SES was also associated with length-for-age shortfalls, a trend that was consistent across the majority of sites, suggests that targeted reductions in disparities of modifiable SES factors may be an effective intervention strategy to reduce stunting in resource-poor communities. While SES is a likely surrogate for more immediate causes, including poor nutrition and higher prevalence of infections, the association with stunting may also be mediated in part by social and psychological stress. Recent studies have shown that SES stressors may result in epigenetic changes and activation of inflammatory pathways [39]. A low percent of energy from protein in complementary foods was also associated with length-for-age shortfalls, and the magnitude of its effect increased with age. In our study settings, percent of energy from protein may also be a surrogate for the overall quality of the diet, particularly consumption of animal-source foods.

Our study has some potential shortcomings. First, complicated feedback loops between layers of risk factors render analysis difficult. For example, gut inflammation and permeability and systemic inflammation may co-vary with the prevalence of enteropathogenic infections or poor sanitary conditions. Second, although the study was conducted in 8 settings, bias in length-for-age measurements at the Pakistan site meant excluding data from this site from our analysis. Third, missing data were another drawback; with the breadth of data collected and range of settings, loss to follow-up and missing data were inevitable. Specifically, our analysis obtained longitudinal data for only 69% of the cohort. Fourth, some of the factors were averaged across age given the limited number of measurements available, which may not reflect true temporal associations. Some individuals had only 1 or 2 measurements of these factors. Therefore, to include these data without losing information, we thought it would be best to average the information across the follow-up period. This is a limitation and comes at the cost of being able to more carefully analyze the temporal contributions of these factors, but we thought it was better to include them crudely with an average than to not include them at all. However, the use of age by factor interaction terms allowed us to evaluate how these indices had a temporal effect on being in a lower length-for-age category with respect to age. Fifth, participating families in Fortaleza, Brazil, had a high dropout rate either because they left the study area due to problems with urban violence or because it was too dangerous for study staff to visit them routinely. Sixth, we recognize that some of our resource-poor populations may have been better off than others, as noted by differences in caloric and protein intake, WAMI index, and linear growth outcomes. The study populations in Brazil, Nepal, and South Africa, while poor, were indeed better off populations than many other resource-poor settings in these countries, thus limiting our capacity make generalizations from site-specific findings. Finally, gestational age at delivery was not assessed in our study, and this may have affected our assessment of length-for-age. It is important to note that our eligibility criteria excluded children weighing < 1.5 kg at birth (a proxy for preterm birth). However, our study also has several strengths. First, the MAL-ED study collected a comprehensive array of postnatal exposures across a diversity of settings worldwide. Second, all sites used a standardized protocol and data collection tools. Regular training, quality assurance, and quality control procedures enabled the study to maintain comparability across sites. Third, prospective follow-up with regular home visits allowed us to capture longitudinal morbidity and dietary information.

In summary, the results presented here from one of the most comprehensive, multi-center longitudinal studies to date suggest that factors leading to stunting are established early in childhood. Neonatal and maternal factors were found to play a more influential role than postnatal factors in defining length-for-age category during early childhood, and their contributions persisted throughout the first 24 months of life. Postnatal exposures, specifically a higher average number of non-diarrheal enteropathogens, lower SES, and lower protein content of the diet, became increasingly important contributors with age. Our results suggest that maternal interventions, especially during pregnancy, are likely to have intergenerational effects and a lasting impact on birthweight and child growth outcomes. Initiatives to address childhood stunting can also consider improvements to the composition of non-breast-milk foods (i.e., higher protein) and strategies to reduce gut pathogen exposure.

## Supporting information

S1 STROBE Checklist STROBE checklist. (DOCX)

S1 Fig Fraction of data missing by risk factor. (TIFF)

S2 Fig Longitudinal trajectories of WAMI index scores by age for each individual and for each site. The top panels show the time-varying values (wami), and the bottom panels show the averaged values (mean_wami).
(TIFF)

S3 Fig Longitudinal trajectories of food insecurity scores by age for each individual and for each site. The top panels show the time-varying values (fsqscore), and the bottom panels show the averaged values (mean_fsq).
(TIFF)

S4 Fig Interquartile cumulative odds ratios of being in a lower length-for-age category at enrollment, 12 months, and 24 months for 5 risk factors, with estimates obtained from logistic regression models dichotomized at the 2 different thresholds of length-for-age (−1 and −2) and those obtained from the ordinal logistic regression model. (TIFF)

S5 Fig Goodness of fit assessed as the overlap between the observed and model-predicted probabilities. We calculated 2 standard error bands for the observed probabilities using standard methods, and a 95% confidence interval using 2,000 bootstrap replications in which children were chosen with replacement.
(TIFF)

S6 Fig Interquartile cumulative odds ratios of being in a lower length-for-age category at enrollment, 12 months, and 24 months for 5 risk factors, with adjusted ORs and 95% CIs obtained when using the full dataset and analytical sample. We conducted a sensitivity analysis using all data available (full data), instead of limiting our analysis to the data of children who met minimal criteria for longitudinal follow-up. The adjusted cumulative ORs and 95% CIs were similar when either the full data or analytical sample were used for analysis, suggesting that our results are robust to the dataset used.
(TIFF)

S1 Table Institutional review board approvals. (DOCX)

S2 Table Differences in key factors between children included and those excluded from the analytical dataset. (DOCX)

S3 Table Factor description. The MAL-ED study accumulated a wide array of data collected over many assessments given at separate intervals in accordance with a priori considerations such as cost, relevance, and biological significance. For example, a socioeconomic questionnaire was administered every 6 months to capture changes in household socioeconomics, whereas the main anthropometric outcomes, length and weight, were obtained at regularly planned intervals in the first 2 years of life. In this table, we show the data that were used and when those data were targeted for collection.
(DOCX)

S4 Table Adjusted cumulative interquartile odds ratios for the WAMI index and food insecurity score when using average and time-varying values. (DOCX)

S5 Table Adjusted cumulative odds ratios and 95% confidence intervals estimated from the single multivariable analysis of the age by factor interactions adjusted for sex and site and accounting for child clusters at 3 different ages. (DOCX)

S1 Text MAL-ED manual of procedures. (DOCX)

S2 Text Sample size. (DOCX)

S3 Text Analytical sample. (DOCX)

S4 Text Model specification. (DOCX)

S5 Text Testing the proportionality of odds assumption and goodness of fit. (DOCX)

S6 Text Proposed analytical plan. (DOCX)

## Abbreviations

AAT: alpha-1 antitrypsin
AGP: alpha-1-acid glycoprotein
ALRI: acute lower respiratory infection
BGD: Dhaka, Bangladesh
BRF: Fortaleza, Brazil
INV: Vellore, India
MPO: myeloperoxidase
NEB: Bhaktapur, Nepal
NEO: neopterin
OR: odds ratio
PEL: Loreto, Peru
PKN: Naushahro Feroze, Pakistan
SAV: Venda, South Africa
SES: socioeconomic status
TZH: Haydom, Tanzania
