# Supplementary material for: Childhood stunting in relation to the pre- and postnatal environment during the first 2 years of life: The MAL-ED longitudinal birth cohort study
Source: PLoS Med. 2017 Oct 25;14(10):e1002408. doi: 10.1371/journal.pmed.1002408 (PMC5656304; doi:10.1371/journal.pmed.1002408)
Supplement: S1 STROBE Checklist — (DOCX) [file pmed.1002408.s001.docx]

**S1 STROBE Checklist. STROBE Statement.**

|  | | Item No | Recommendation |
| --- | --- | --- | --- |
| **Title and abstract** | | 1 | (*a*) Indicate the study’s design with a commonly used term in the title or the abstract: Done **Title and Abstract** |
|  |  |  | (*b*) Provide in the abstract an informative and balanced summary of what was done and what was found: **Abstract** |
| Introduction | | | |
| Background/rationale | | 2 | Explain the scientific background and rationale for the investigation being reported: **Introduction, paragraph 1** |
| Objectives | | 3 | State specific objectives, including any prespecified hypotheses: Done page **Introduction, paragraph 1** |
| Methods | | | |
| Study design | | 4 | Present key elements of study design early in the paper: Done page **Methods, Study design and participants, paragraph 1** |
| Setting | | 5 | Describe the setting, locations, and relevant dates, including periods of recruitment, exposure, follow-up, and data collection: **Methods, Study design and participants, paragraph 1** |
| Participants | | 6 | *Cohort study*—Give the eligibility criteria, and the sources and methods of selection of participants:  **Methods, Study design and participants, paragraph 2** |
| Variables | | 7 | Clearly define all outcomes, exposures, predictors, potential confounders, and effect modifiers. Give diagnostic criteria, if applicable: **Methods, Outcome subsection, paragraphs 1** |
| Data sources/ measurement | | 8* | For each variable of interest, give sources of data and details of methods of assessment (measurement). Describe comparability of assessment methods if there is more than one group: **Methods, Neonatal and maternal factors, Household factors, Illness variables, Microbiology, Infant feeding practices and dietary intake, Micronutrient status, systemic inflammation, and gut inflammation and permeability; Table S4** |
| Bias | | 9 | Describe any efforts to address potential sources of bias: **Discussion, paragraph 6** |
| Study size | | 10 | Explain how the study size was arrived at: Methods, **Study design and participants, paragraph 2, Table S5** |
| Quantitative variables | | 11 | Explain how quantitative variables were handled in the analyses. If applicable, describe which groupings were chosen and why: **Methods, Biostatistical Methods, Table S4, Table S6** |
| Statistical methods | | 12 | (*a*) Describe all statistical methods, including those used to control for confounding **Methods, Biostatistical Methods, Text S3** |
|  |  |  | (*b*) Describe any methods used to examine subgroups and interactions: Done page 7-8, **Methods, Biostatistical Methods** |
|  |  |  | (*c*) Explain how missing data were addressed: **Methods, Biostastistical Methods** |
|  |  |  | *Cohort study*—If applicable, describe analytical methods taking account of sampling strategy: **N/A** |
|  |  |  | (*e*) Describe any sensitivity analyses: **Methods, Biostastistical Methods** |
| Results | | | |
| Participants | 13* | (a) Report numbers of individuals at each stage of study—eg numbers potentially eligible, examined for eligibility, confirmed eligible, included in the study, completing follow-up, and analysed: **Results, paragraph 1** | |
|  |  | (b) Give reasons for non-participation at each stage: **Results, paragraph 1** | |
|  |  | (c) Consider use of a flow diagram: N/A | |
| Descriptive data | 14* | (a) Give characteristics of study participants (eg demographic, clinical, social) and information on exposures and potential confounders: **Table 1** | |
|  |  | (b) Indicate number of participants with missing data for each variable of interest: **Fig S1** | |
|  |  |  | |
| Outcome data | 15* | *Cohort study—*Report numbers of outcome events or summary measures: **Results, Fig 2, Fig 3** | |
| Main results | 16 | (*a*) Give unadjusted estimates and, if applicable, confounder-adjusted estimates and their precision (eg, 95% confidence interval). Make clear which confounders were adjusted for and why they were included: **Methods, Biostatistics Methods, Table S7, Fig 4, Fig 5** | |
|  |  | (*b*) Report category boundaries when continuous variables were categorized: **Methods, Outcome** | |
|  |  | (*c*) If relevant, consider translating estimates of relative risk into absolute risk for a meaningful time period: **N/A** | |
| Other analyses | 17 | Report other analyses done—eg analyses of subgroups and interactions, and sensitivity analyses: **Fig S4, Fig S5, Table S6** | |
| Discussion | | | |
| Key results | 18 | Summarise key results with reference to study objectives: **Results, Fig 4** | |
| Limitations | 19 | Discuss limitations of the study, taking into account sources of potential bias or imprecision. Discuss both direction and magnitude of any potential bias: **Discussion, paragraph 6** | |
| Interpretation | 20 | Give a cautious overall interpretation of results considering objectives, limitations, multiplicity of analyses, results from similar studies, and other relevant evidence: **Discussion (throughout)** | |
| Generalisability | 21 | Discuss the generalisability (external validity) of the study results: **Discussion (throughout)** | |
| Other information | | | |
| Funding | 22 | Give the source of funding and the role of the funders for the present study and, if applicable, for the original study on which the present article is based: **In the System** | |
