# Supplementary figures and images for: Childhood stunting in relation to the pre- and postnatal environment during the first 2 years of life: The MAL-ED longitudinal birth cohort study
Source: PLoS Med. 2017 Oct 25;14(10):e1002408. doi: 10.1371/journal.pmed.1002408 (PMC5656304; doi:10.1371/journal.pmed.1002408)

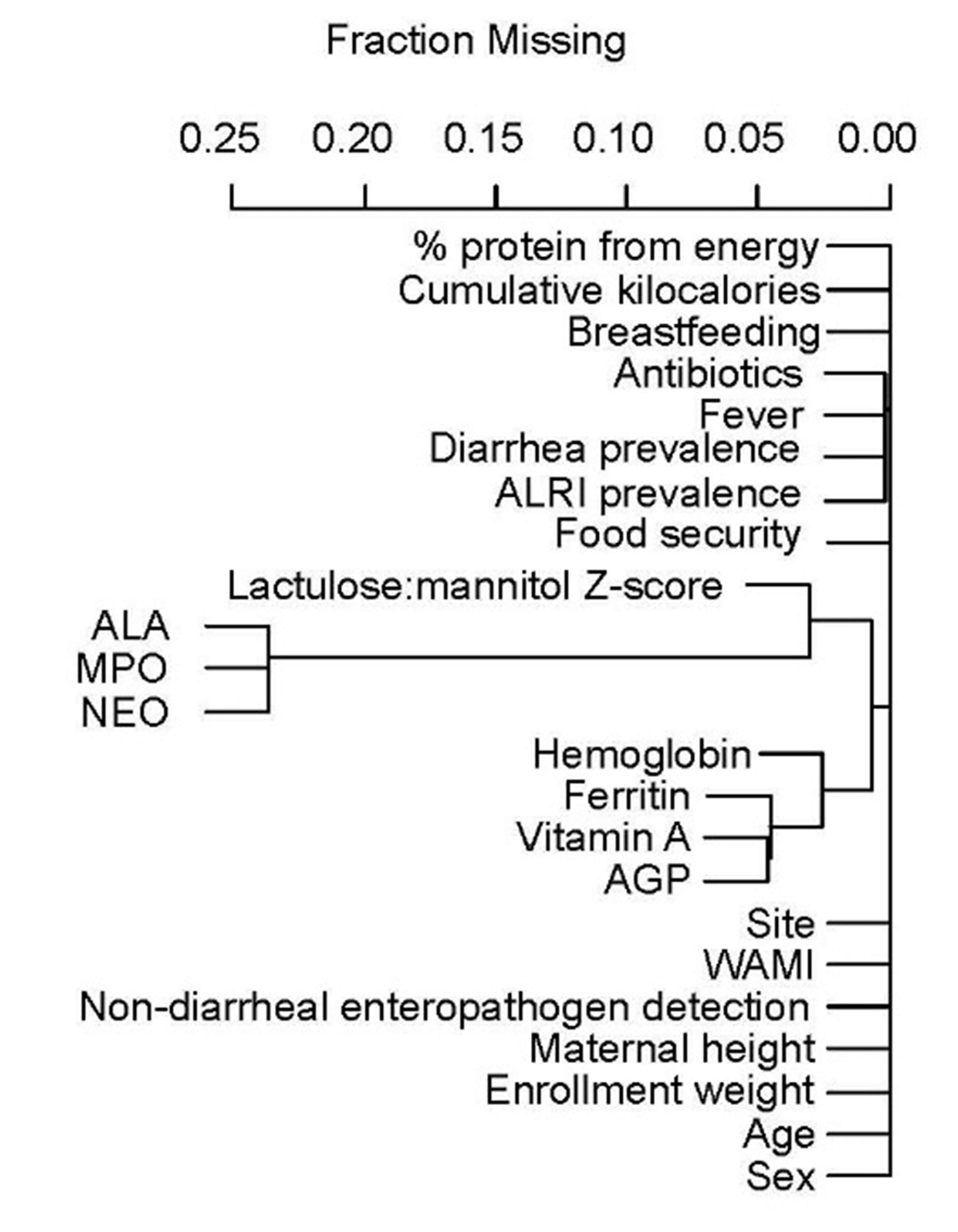

Supplement: S1 Fig — (TIFF) [file pmed.1002408.s002.tiff]

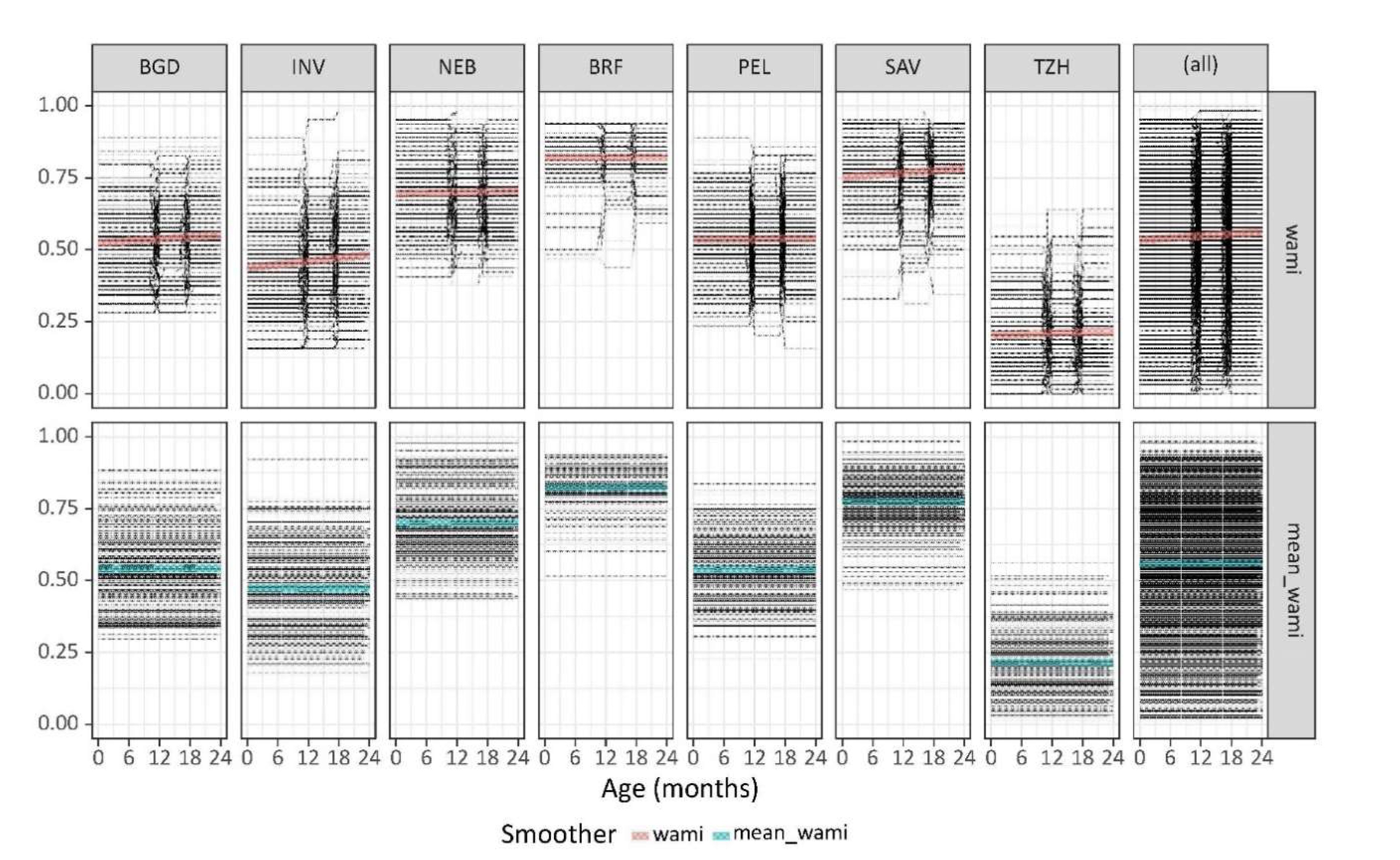

Supplement: S2 Fig — The top panels show the time-varying values (wami), and the bottom panels show the averaged values (mean_wami). (TIFF) [file pmed.1002408.s003.tiff]

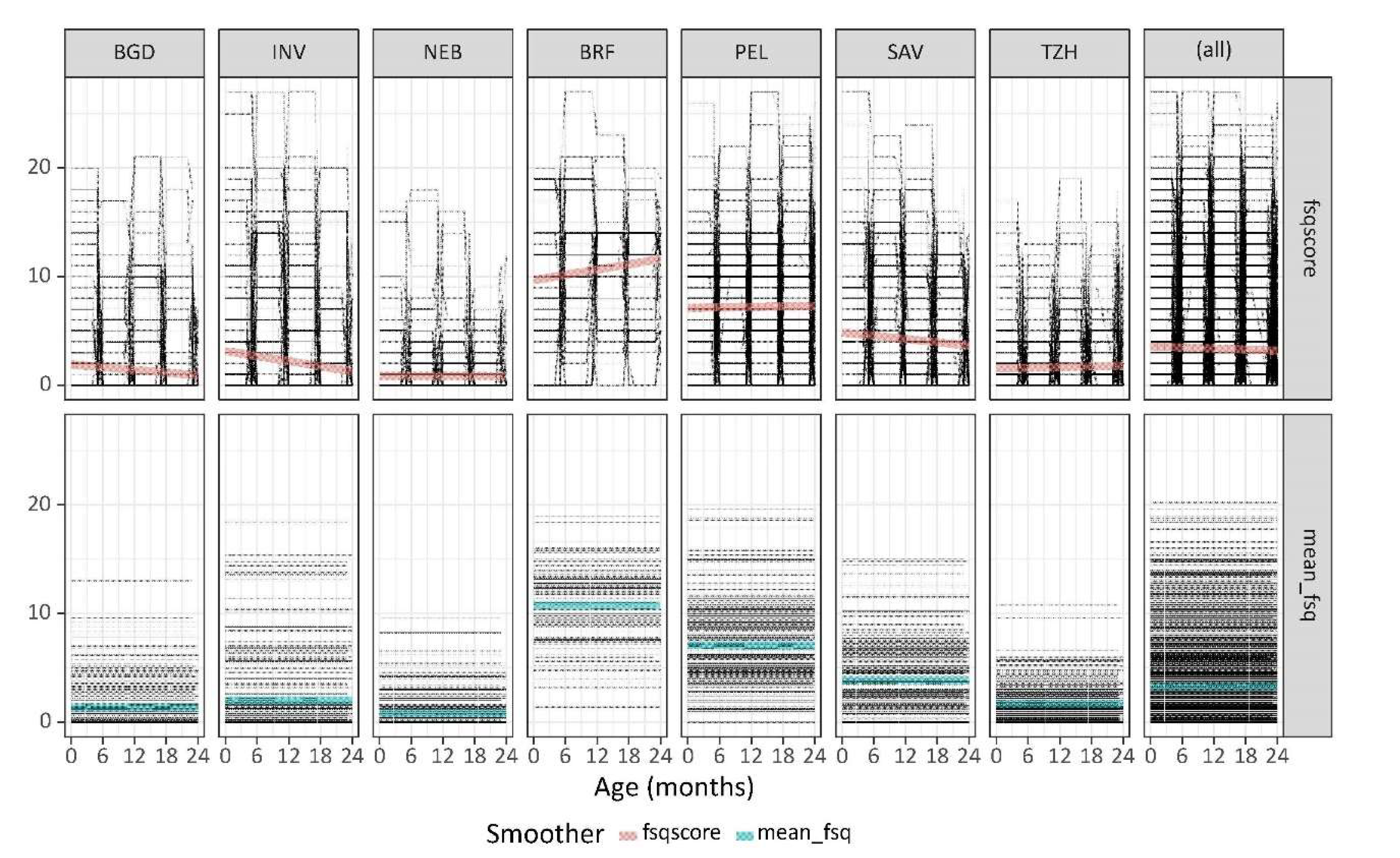

Supplement: S3 Fig — The top panels show the time-varying values (fsqscore), and the bottom panels show the averaged values (mean_fsq). (TIFF) [file pmed.1002408.s004.tiff]

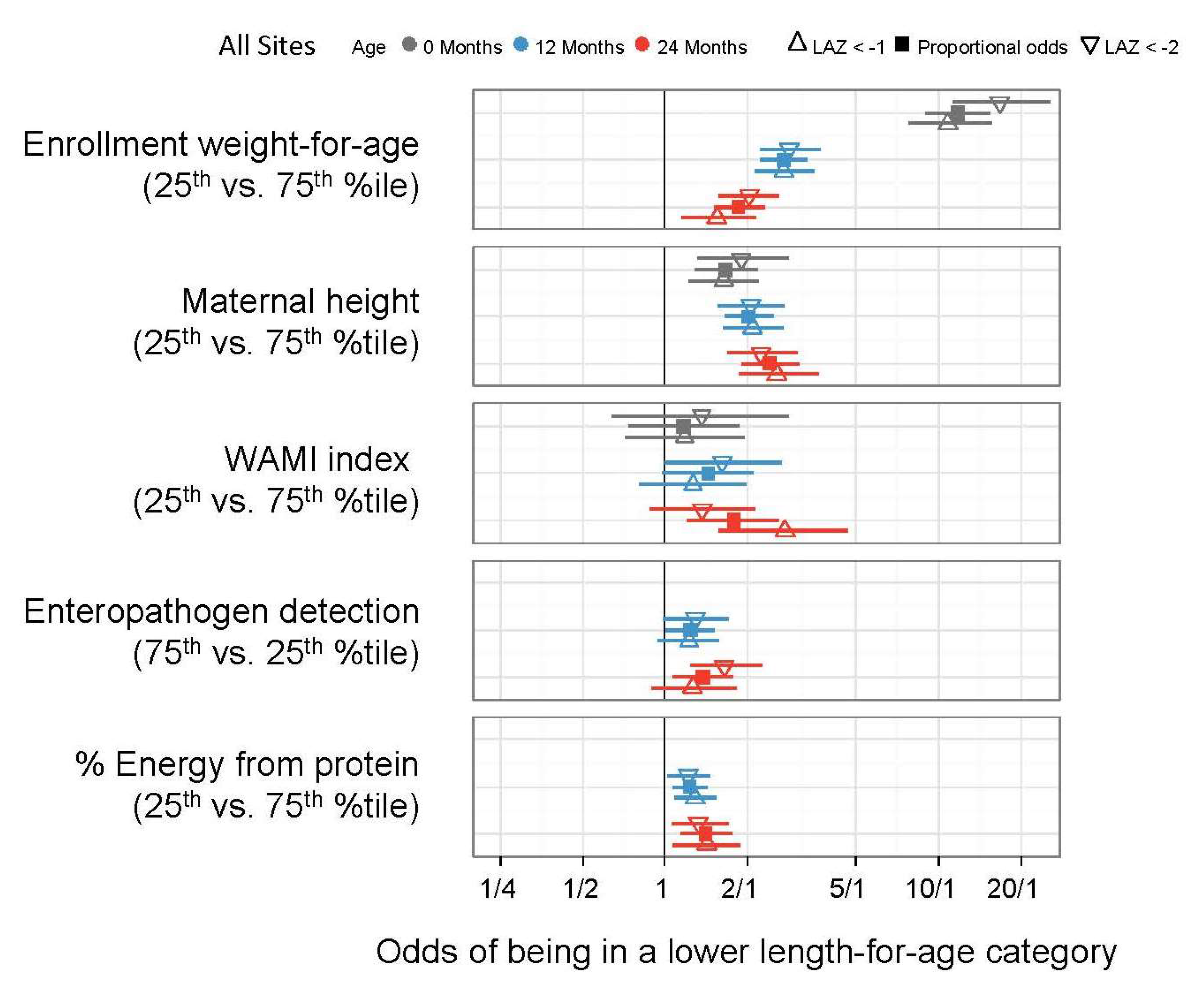

Supplement: S4 Fig — (TIFF) [file pmed.1002408.s005.tiff]

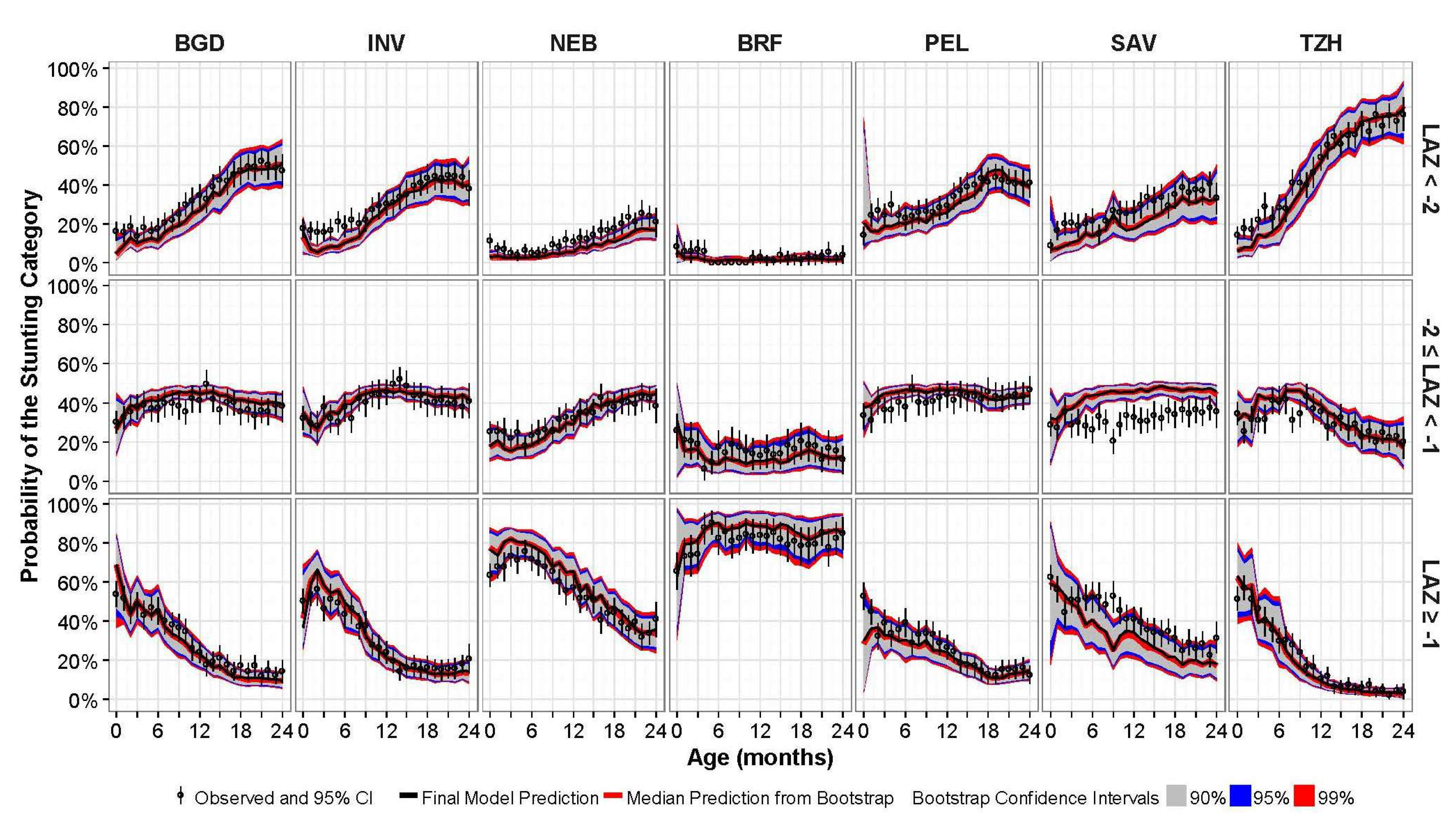

Supplement: S5 Fig — We calculated 2 standard error bands for the observed probabilities using standard methods, and a 95% confidence interval using 2,000 bootstrap replications in which children were chosen with replacement. (TIFF) [file pmed.1002408.s006.tiff]

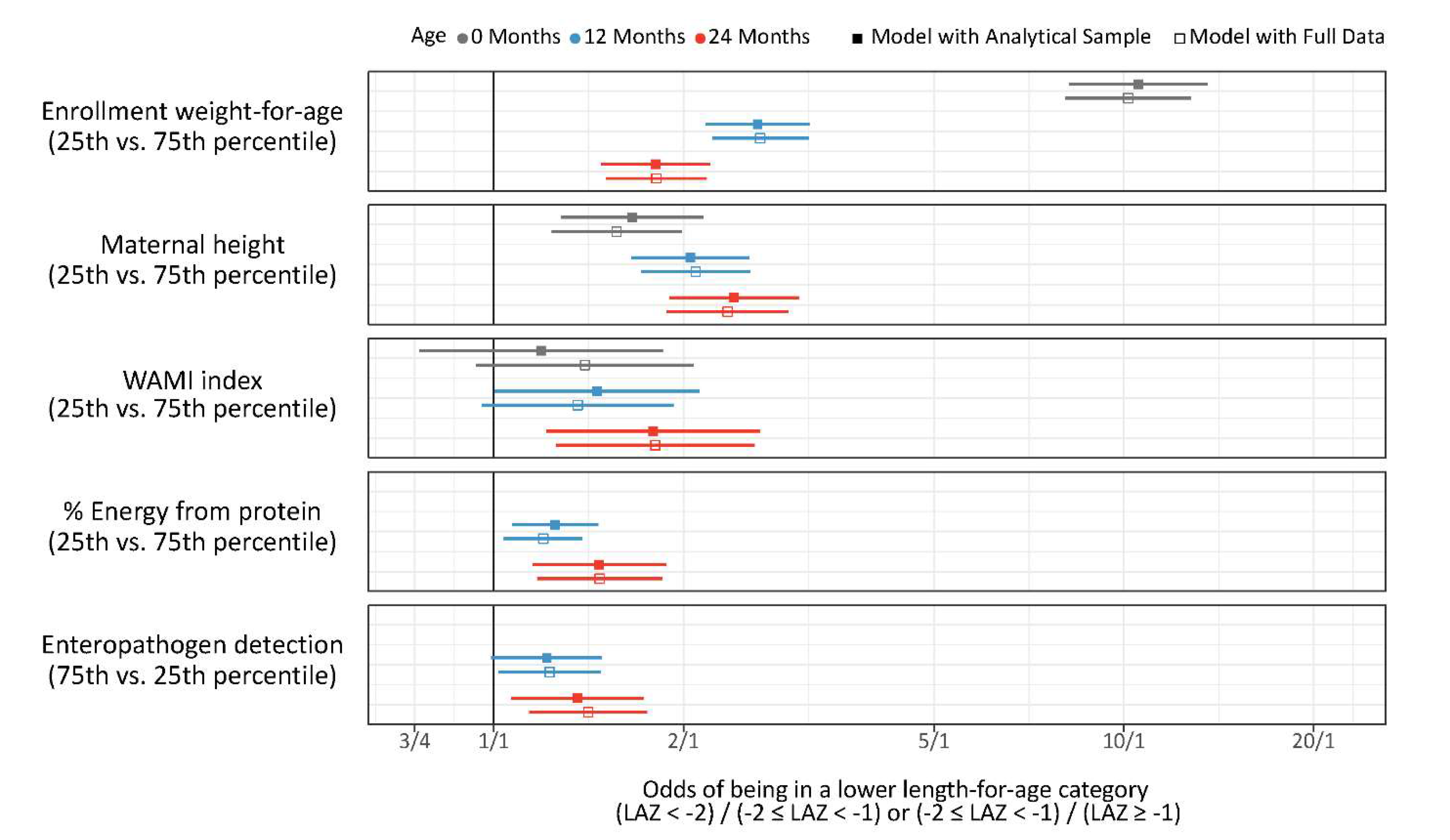

Supplement: S6 Fig — We conducted a sensitivity analysis using all data available (full data), instead of limiting our analysis to the data of children who met minimal criteria for longitudinal follow-up. The adjusted cumulative ORs and 95% CIs were similar when either the full data or analytical sample were used for analysis, suggesting that our results are robust to the dataset used. (TIFF) [file pmed.1002408.s007.tiff]
