# Supplementary material for: Childhood stunting in relation to the pre- and postnatal environment during the first 2 years of life: The MAL-ED longitudinal birth cohort study
Source: PLoS Med. 2017 Oct 25;14(10):e1002408. doi: 10.1371/journal.pmed.1002408 (PMC5656304; doi:10.1371/journal.pmed.1002408)
Supplement: S1 Table — (DOCX) [file pmed.1002408.s008.docx]

**S1 Table. Institutional Research Boards Approvals.**

| **Nepal** | |
| --- | --- |
|  | Institute of Medicine, TU; Institutional Review Board |
|  | Nepal Health Research Council; Ethical Review Board |
|  | Walter Reed Army Institute of Research; Institutional Review Board |
| **Pakistan** | |
|  | Aga Khan University; Ethical Review Committee |
| **Peru** | |
|  | Bloomberg School of Public Health, Johns Hopkins University; Institutional Review Board |
|  | A.B. PRISMA Ethics Committee |
|  | Health Ministry, Loreto |
| **South Africa** | |
|  | University of Venda; Health, Safety and Research Ethics Committee |
|  | Limpopo Provincial Government; Dept of Health and Social Development |
|  | University of Virginia; Institutional Review Board for Health Sciences Research |
| **Tanzania** | |
|  | National Institute for Medical Research; Medical Research Coordinating Committee |
|  | Ministry of Health and Social Welfare; Chief Medical Officer |
|  | University of Virginia; Institutional Review Board for Health Sciences Research |
| **India** | |
|  | Christian Medical College; Institutional Review Board |
|  | Indian Council of Medical Research; Health Ministry Screening Committee |
| **Brazil** | |
|  | Universidade Federal do Ceara; Committee for Ethics in Research |
|  | Health Ministry, Council of National Health; National Ethical Research Committee |
|  | University of Virginia; Institutional Review Board for Health Sciences Research |
| **Bangladesh** | |
|  | ICDDR,B; Ethical Review Committee |
|  | University of Virginia; Institutional Review Board for Health Sciences Research |
