# Supplementary material for: Childhood stunting in relation to the pre- and postnatal environment during the first 2 years of life: The MAL-ED longitudinal birth cohort study
Source: PLoS Med. 2017 Oct 25;14(10):e1002408. doi: 10.1371/journal.pmed.1002408 (PMC5656304; doi:10.1371/journal.pmed.1002408)
Supplement: S2 Table — (DOCX) [file pmed.1002408.s009.docx]

**S2 Table. Differences in key variables between children included and those excluded from the analytical dataset.**  In this table, we show a comparison of key variables between children included vs. those excluded from the analytical dataset. We used t-tests with a Bonferroni correction for multiple comparisons given that we had 7 countries.

|  | Enrollment weight (kg) | | Food insecurity  score (units) | | Maternal height (cm) | | Proportion with maternal education  ≥ 6 years | | Maximum age breastfeeding (days) | | Proportion of girls | | WAMI score at six months | |
| --- | --- | --- | --- | --- | --- | --- | --- | --- | --- | --- | --- | --- | --- | --- |
|  | Included vs. excluded | p | Included vs. excluded | p | Included vs. excluded | p | Included vs. excluded | p | Included vs. excluded | p | Included vs. excluded | p | Included vs. excluded | p |
| BGD | 2.77 vs. 2.69 | 1.0 | 1.35 vs. 2.46 | 0.45 | 148.90 vs. 148.69 | 1.0 | 0.36 vs. 0.52 | 0.83 | 103.53 vs. 83.37 | 0.11 | 0.49 vs. 0.59 | 1.0 | 0.53 vs. 0.53 | 1.0 |
| INV | 2.94 vs. 2.85 | 1.0 | 2.00 vs. 2.58 | 1.0 | 151.17 vs. 150.95 | 1.0 | 0.63 vs. 0.66 | 1.0 | 75.83 vs. 81.14 | 1.0 | 0.56 vs. 0.53 | 1.0 | 0.45 vs. 0.5 | 0.66 |
| NEB | 3.17 vs. 3.00 | 0.53 | 0.82 vs. 0.53 | 0.8 | 149.58 vs. 151.61 | 0.25 | 0.74 vs. 0.82 | 1.0 | 58.76 vs. 60.69 | 1.0 | 0.46 vs. 0.44 | 1.0 | 0.70 vs. 0.73 | 1.0 |
| BRF | 3.47 vs. 3.41 | 1.0 | 10.75 vs. 10.52 | 1.0 | 154.86 vs. 155.99 | 1.0 | 0.80 vs. 0.91 | 0.2 | 63.74 vs. 63.24 | 1.0 | 0.36 vs. 0.55 | 0.03 | 0.82 vs. 0.82 | 1.0 |
| PEL | 3.09 vs. 3.10 | 1.0 | 7.08 vs. 7.29 | 1.0 | 149.85 vs. 150.26 | 1.0 | 0.77 vs. 0.81 | 1.0 | 39.39 vs. 40.96 | 1.0 | 0.43 vs. 0.54 | 0.51 | 0.54 vs. 0.55 | 1.0 |
| SAV | 3.32 vs. 3.22 | 0.72 | 3.87 vs. 3.95 | 1.0 | 158.72 vs. 158.09 | 1.0 | 0.97 vs. 0.97 | 1.0 | 28.69 vs. 31.83 | 1.0 | 0.49 vs. 0.53 | 1.0 | 0.76 vs. 0.74 | 1.0 |
| TZH | 3.38 vs. 3.35 | 1.0 | 1.67 vs. 1.53 | 1.0 | 155.79 vs. 156.04 | 1.0 | 0.63 vs. 0.66 | 1.0 | 44.83 vs. 43.75 | 1.0 | 0.50 vs. 0.52 | 1.0 | 0.20 vs. 0.25 | 0.15 |
