# Supplementary material for: Childhood stunting in relation to the pre- and postnatal environment during the first 2 years of life: The MAL-ED longitudinal birth cohort study
Source: PLoS Med. 2017 Oct 25;14(10):e1002408. doi: 10.1371/journal.pmed.1002408 (PMC5656304; doi:10.1371/journal.pmed.1002408)
Supplement: S3 Table — The MAL-ED study accumulated a wide array of data collected over many assessments given at separate intervals in accordance with a priori considerations such as cost, relevance, and biological significance. For example, a socioeconomic questionnaire was administered every 6 months to capture changes in household socioeconomics, whereas the main anthropometric outcomes, length and weight, were obtained at regularly planned intervals in the first 2 years of life. In this table, we show the data that were used and when those data were targeted for collection. (DOCX) [file pmed.1002408.s010.docx]

**S3 Table. Variable description.** The MAL-ED study accumulated a wide array of data collected over many assessments given at separate intervals in accordance with *a priori* considerations such as cost, relevance, and biological significance. For example, a socioeconomic questionnaire was administered every six months to capture changes in household socioeconomics whereas the main anthropometric outcomes, length or weight, were obtained at regularly planned intervals in the first two years of life. In this table, we show the data that were used and when that data was targeted for collection.

|  | | |
| --- | --- | --- |
| **Variable** | **Description** | **Collected ,at:** |
| Serum hemoglobin concentration | Average hemoglobin concentration in mg/dL, calculated as the sum of measurements divided by the number of measurements available | 7, 15, and 24 months |
| Stool alpha-1 anti-trypsin concentration | mg/g | Months 1-12, 15, 18 and 24,  but after month 12 due to limited collections some systematic forward-filling occurs |
| Diagnosis of ALRI | Cumulative number of times ALRI was diagnosed | 0 to 24 months |
| Diagnosis of dehydrating diarrhea | Cumulative number of times dehydrating diarrhea was diagnosed | 0 to 24 months |
| Lactulose:mannitol Z-score | Time-varying value | 3,6, ,9 and 15 months: months 0-5 are filled with the value from month 3; 6-8, the value at month 6; 9-14, the value at month 9; 15 to 24, the value at month 15 |
| Maternal height | Maternal height (cm) | Enrolment |
| Mean ferritin | Average ferritin concentration (mcg/L or ng/ml): sum of measurements divided by number of measurements available | 7,15, and 24 months |
| Mean food security score | Average food insecurity score: sum of scores divided by number of assessments available | 0, 6, 12, 18, and 24 months |
| Mean hemoglobin | Average hemoglobin concentration (g/dL): sum of measurements divided by number of measurements available | 7, 15, and 24 months |
| Mean retinol concentration | Average retinol concentration (mcg/dL): sum of measurements divided by number of measurements available | 7, 15, and 24 months |
| Mean alpha-1-acid glycoprotein concentration | Average retinol concentration (mg/dL): sum of measurements divided by number of measurements available | 7, 15, and 24 months |
| Mean WAMI | Average WAMI: the sum of WAMI scores from each assessment divided by number of assessments | 6, 12, 18, and 24 months |
| Myeloperoxidase concentration | ng/mL | Months 1-12, 15, 18, and 24 months, but after month 12 due to limited collections some systematic forward-filling occurs |
| Neopterin concentration | nmol/L | Months 1-12, 15, 18, and 24, but after month 12 due to limited collections some systematic forward-filling occurs |
| Pathogen score | Moving average for number of pathogens detected: number of pathogens detected, divided by the number of surveillance stools tested | 0 to 24 months |
| Percentage of days with ALRI | Cumulative percentage of maternally reported  ALRI | 0 to 24 months |
| Percentage of days with antibiotics | Cumulative percentage of maternally reported days of antibiotic use | 0 to 24 months |
| Percentage of days with breastfeeding | Cumulative percentage of reported days of any breastfeeding | 0 to 24 months |
| Percentage of days with diarrhea | Cumulative percentage of maternally reported diarrhea | 0 to 24 months |
| Percentage of days with fever | Cumulative percentage of maternally reported fever | 0 to 24 months |
| Sex | Female or male | Enrolment |
| Site | Site ID |  |
| Energy intake | Cumulative count of kilocalories consumed | 9 to 24 |
| Percent energy from protein | Running average of percent energy from protein | 9 to 24 |
| Weight-for-age Z-score at enrolment | Z-score calculated using WHO program | Enrolment (<17 days after birth) |
