# Supplementary material for: Childhood stunting in relation to the pre- and postnatal environment during the first 2 years of life: The MAL-ED longitudinal birth cohort study
Source: PLoS Med. 2017 Oct 25;14(10):e1002408. doi: 10.1371/journal.pmed.1002408 (PMC5656304; doi:10.1371/journal.pmed.1002408)
Supplement: S4 Table — (DOCX) [file pmed.1002408.s011.docx]

**S4 Table. Adjusted cumulative interquartile odds ratios for the WAMI index and food in security score when using average and time varying values.** There were small variations in either the WAMI index (Figure S4) or food insecurity scores (Figure S5) within individuals over time. Moreover, there were no differences in our analysis when WAMI or food insecurity were included as averaged or when they were included as time varying. See differences below in outcomes below:

|  | Adjusted cumulative interquartile OR (95% CI) | |
| --- | --- | --- |
|  | Averaged WAMI score | Time varying WAMI score |
| 0 months | 1.27 (1.09-1.48) | 1.23 (1.05- 1.44) |
| 12 months | 2.96 (2.55 -3.44) | 2.92 (2.51-3.40) |
| 24 months | 3.26 (2.78 – 3.82) | 3.28 ( 2.78 – 3.87) |

|  | Adjusted cumulative interquartile OR (95% CI) | |
| --- | --- | --- |
|  | Averaged food insecurity score | Time varying food insecurity score |
| 0 months | 0.95 (0.84-1.06) | 1.00 (0.90-1.10) |
| 12 months | 1.16 (1.02-1.31) | 1.14 (1.01-1.28) |
| 24 months | 1.42 (1.21-1.67) | 1.19 (1.06-1.33) |
