# Supplementary material for: Childhood stunting in relation to the pre- and postnatal environment during the first 2 years of life: The MAL-ED longitudinal birth cohort study
Source: PLoS Med. 2017 Oct 25;14(10):e1002408. doi: 10.1371/journal.pmed.1002408 (PMC5656304; doi:10.1371/journal.pmed.1002408)
Supplement: S5 Table — (DOCX) [file pmed.1002408.s012.docx]

**Table S5. Adjusted cumulative odds ratios and 95% confidence interval estimated from the single multivariable analysis of the age by factor interactions adjusted for sex and site and accounting for child clusters at three different ages.** In the table below, we provide a summary of results (adjusted cumulative odds ratios [OR] and 95% confidence interval) estimated from the single multivariable analysis of the age by factor interactions adjusted for sex and site and accounting for child clusters at three different ages (0, 12, and 24 months of age):

| **Variable** | **Age (months)** | **Reference value (best quartile or lowest number)** | **Test value (worst quartile or lowest number)** | **OR** | **95% CI** | |
| --- | --- | --- | --- | --- | --- | --- |
| Enrollment weight-for-age Z-score (25^th^ vs. 75^th^ percentile) | 0 | -0.02 | -1.38 | 11.33 | 8.73 | 14.70 |
|  | 12 | -0.01 | -1.38 | 2.66 | 2.19 | 3.22 |
|  | 24 | -0.01 | -1.41 | 1.82 | 1.49 | 2.23 |
| Maternal height (25^th^ vs. 75^th^ percentile) | 0 | 157.0 | 147.6 | 1.65 | 1.28 | 2.14 |
|  | 12 | 157.0 | 148.0 | 2.00 | 1.63 | 2.47 |
|  | 24 | 156.8 | 147.8 | 2.38 | 1.89 | 3.01 |
| Enteropathogen detection (75^th^ vs. 25^th^ percentile) | 12 | 0.58 | 1.17 | 1.21 | 0.99 | 1.49 |
|  | 24 | 0.69 | 1.25 | 1.36 | 1.07 | 1.73 |
| Mean WAMI Index (25^th^ vs. 75^th^ percentile) | 0 | 0.73 | 0.40 | 1.19 | 0.76 | 1.85 |
|  | 12 | 0.73 | 0.41 | 1.43 | 1.00 | 2.05 |
|  | 24 | 0.75 | 0.45 | 1.75 | 1.21 | 2.55 |
| Hemoglobin (25^th^ vs. 75^th^ percentile) | 0 | 11.70 | 10.30 | 1.05 | 0.84 | 1.32 |
|  | 12 | 11.67 | 10.27 | 1.05 | 0.88 | 1.25 |
|  | 24 | 11.70 | 10.30 | 1.01 | 0.83 | 1.22 |
| Vitamin A (25^th^ vs. 75^th^ percentile) | 0 | 28.17 | 18.80 | 0.97 | 0.77 | 1.23 |
|  | 12 | 28.05 | 18.93 | 0.91 | 0.74 | 1.12 |
|  | 24 | 28.22 | 19.31 | 1.14 | 0.93 | 1.41 |
| Plasma Ferritin (25^th^ vs. 75^th^ percentile) | 0 | 31.26 | 11.05 | 1.08 | 0.97 | 1.19 |
|  | 12 | 31.05 | 11.33 | 0.88 | 0.75 | 1.03 |
|  | 24 | 31.67 | 11.80 | 0.91 | 0.79 | 1.04 |
| Food insecurity score (25^th^ vs. 75^th^ percentile) | 0 | 5.20 | 0.00 | 1.01 | 0.76 | 1.35 |
|  | 12 | 5.25 | 0.00 | 0.93 | 0.71 | 1.22 |
|  | 24 | 6.20 | 0.00 | 1.20 | 0.87 | 1.65 |
| Lactulose:Mannitol Z-score (75^th^ vs. 25^th^ percentile) | 0 | -0.42 | 0.57 | 1.14 | 0.96 | 1.36 |
|  | 12 | 0.05 | 1.14 | 1.17 | 1.01 | 1.36 |
|  | 24 | -0.16 | 1.13 | 1.08 | 0.91 | 1.29 |
| Square root of stool MPO (75^th^ vs. 25^th^ percentile) | 0 | 49.03 | 130.02 | 1.00 | 0.80 | 1.25 |
|  | 12 | 46.63 | 109.80 | 0.94 | 0.83 | 1.05 |
|  | 24 | 30.38 | 69.55 | 1.03 | 0.89 | 1.19 |
| Square root of stool NEO (75^th^ vs. 25^th^ percentile) | 0 | 26.65 | 51.09 | 0.86 | 0.73 | 1.02 |
|  | 12 | 31.02 | 59.85 | 0.84 | 0.73 | 0.97 |
|  | 24 | 18.50 | 41.33 | 1.01 | 0.88 | 1.16 |
| Square root of stool AAT (75^th^ vs. 25^th^ percentile) | 0 | 0.44 | 0.90 | 0.92 | 0.74 | 1.14 |
|  | 12 | 0.41 | 0.80 | 0.96 | 0.84 | 1.11 |
|  | 24 | 0.32 | 0.69 | 1.14 | 0.96 | 1.35 |
| Alpha-1-acid glycoprotein (75^th^ vs. 25^th^ percentile) | 0 | 87.00 | 130.17 | 0.72 | 0.57 | 0.91 |
|  | 12 | 87.00 | 128.00 | 1.01 | 0.83 | 1.23 |
|  | 24 | 86.00 | 127.33 | 1.18 | 0.96 | 1.45 |
| Longitudinal diarrhea prevalence (75^th^ vs. 25^th^ percentile) | 12 | 0.24 | 3.68 | 1.00 | 0.86 | 1.16 |
|  | 24 | 0.40 | 3.36 | 0.89 | 0.75 | 1.07 |
| Longitudinal ALRI prevalence (75^th^ vs. 25^th^ percentile) | 12 | 0.00 | 0.26 | 1.00 | 0.94 | 1.06 |
|  | 24 | 0.00 | 0.27 | 1.02 | 0.95 | 1.09 |
| Longitudinal fever prevalence (75^th^ vs. 25^th^ percentile) | 12 | 1.05 | 5.53 | 1.21 | 0.97 | 1.51 |
|  | 24 | 1.07 | 5.37 | 1.11 | 0.84 | 1.46 |
| Number of ALRI diagnoses (0 vs. 1) | 12 | 0.00 | 1.00 | 1.03 | 0.98 | 1.08 |
|  | 24 | 0.00 | 1.00 | 1.04 | 0.98 | 1.10 |
| Number of diarrhea diagnoses (0 vs. 1) | 12 | 0.00 | 1.00 | 1.00 | 0.83 | 1.08 |
|  | 24 | 0.00 | 1.00 | 1.00 | 0.98 | 1.46 |
| Longitudinal antibiotic use (75^th^ vs. 25^th^ percentile) | 12 | 1.58 | 9.21 | 0.96 | 0.77 | 1.20 |
|  | 24 | 1.88 | 8.86 | 0.81 | 0.62 | 1.06 |
| Days with partial breastfeeding (75^th^ vs. 25^th^ percentile) | 12 | 100.00 | 50.00 | 1.00 | 1.00 | 1.00 |
|  | 24 | 100.00 | 68.19 | 1.10 | 0.82 | 1.46 |
| Cumulative energy intake (25^th^ vs. 75^th^ percentile) | 12 | 2.54 | 0.97 | 0.97 | 0.82 | 1.15 |
|  | 24 | 13.92 | 7.31 | 1.54 | 1.10 | 2.16 |
| % Energy from protein (25^th^ vs. 75^th^ percentile) | 12 | 13.35 | 9.78 | 1.22 | 1.06 | 1.41 |
|  | 24 | 12.80 | 10.39 | 1.39 | 1.13 | 1.72 |
