# Supplementary material for: Childhood stunting in relation to the pre- and postnatal environment during the first 2 years of life: The MAL-ED longitudinal birth cohort study
Source: PLoS Med. 2017 Oct 25;14(10):e1002408. doi: 10.1371/journal.pmed.1002408 (PMC5656304; doi:10.1371/journal.pmed.1002408)
Supplement: S1 Text — (DOCX) [file pmed.1002408.s013.docx]

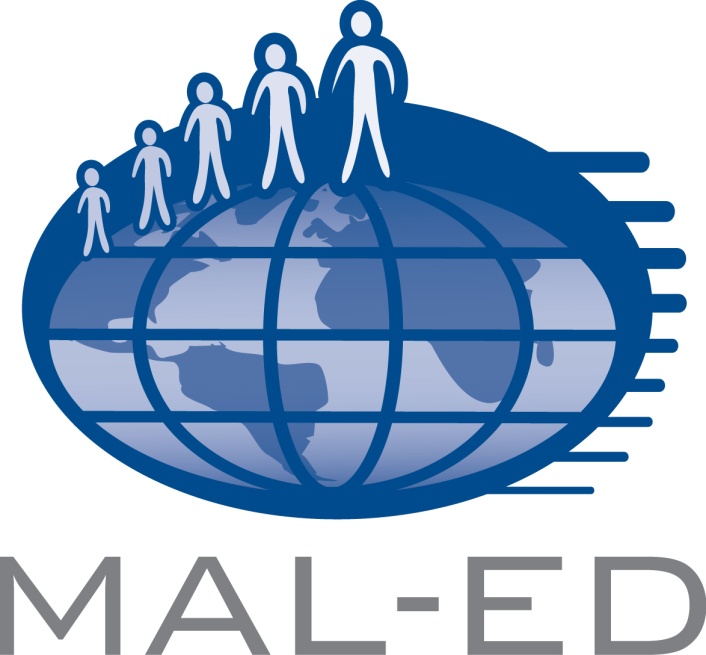


**Etiology, Risk Factors and Interactions of Enteric Infections and Malnutrition and the Consequences for Child Health and Development**

Birth Cohort Studies

Manual of Procedures

November, 2014

**Table of Contents**

[Introduction to the Manual of Procedures iii](#_Toc403633611)

[Background and Rationale iv](#_Toc403633612)

[Abbreviations vii](#_Toc403633613)

[**Recruit, Consent and Enroll** 1](#_Toc403633614)

[SCR—Screening Process 2](#_Toc403633615)

[PID—Participant ID Log SOP 4](#_Toc403633616)

[NPF—Non-Participant Form SOP 5](#_Toc403633617)

[Consenting Process 8](#_Toc403633618)

[CAF—Child Assesment Form SOP 10](#_Toc403633619)

[**Demographic, SES and Food Security Assessments** 12](#_Toc403633620)

[DAF—Baseline Demographic Questionnaire 13](#_Toc403633621)

[FSQ—Food Security Questionnaire 15](#_Toc403633622)

[FSE—Followup SES Questionnaire 17](#_Toc403633623)

[**Household Surveillance** 25](#_Toc403633624)

[SAF—Surveillance Assessment Form 26](#_Toc403633625)

[XAF—Surveillance Check Form 34](#_Toc403633626)

[NUR—Nursing Notes Form 36](#_Toc403633627)

[REF—Child Referral Form SOP 37](#_Toc403633628)

[MOA—Monthly Form A 40](#_Toc403633629)

[MAF—Maternal Assessment Form 45](#_Toc403633630)

[MOB—Monthly Form B 47](#_Toc403633631)

[MOC—Monthly Form C 50](#_Toc403633632)

[ANT – Anthropometry Check Form 55](#_Toc403633633)

[NCF—Non-Continuation Form SOP 57](#_Toc403633634)

[QCS—Surveillance Quality Monitoring: Guidelines for Field Supervisors 59](#_Toc403633635)

[Surveillance Assessment Form – Quality Control Plan 63](#_Toc403633636)

[Anthropometry Quality Control Plan 65](#_Toc403633637)

[Supervisor checklist 66](#_Toc403633638)

[VIF—Vaccine Information Form SOP 68](#_Toc403633639)

[**Collection and Processing of Clinical SampleS** 70](#_Toc403633640)

[SIL—Sample ID Log SOP 71](#_Toc403633641)

[**Stool** 72](#_Toc403633642)

[SCT/SFC—Stool Collection, Processing and Transport to the Laboratory 73](#_Toc403633643)

[RSS/SRF—Receiving and Storage of Stool Samples SOP 79](#_Toc403633644)

[Stool Specimen Test Quantities and Storage 87](#_Toc403633645)

[QNS—Processing and Testing Quantity Not Sufficient Stool 88](#_Toc403633646)

[Microbiology Stool Aliquoting Flow Chart 92](#_Toc403633647)

[ELISA thawed specimen stability & work flow 93](#_Toc403633648)

[BDF—Stool Bacteriology Culture and Identification: Bacteriology Data Form SOP 94](#_Toc403633649)

[ECP—Identification of Diarrheagenic E.coli by Multiplex PCR SOP 110](#_Toc403633650)

[FVF—Fecal Viral ELISA Tests 116](#_Toc403633651)

[NRV—Detection of Norovirus by RT-PCR 120](#_Toc403633652)

[MDF—Microscopy for the Detection of Ova and Parasites in Stool: Microscopy Data Form SOP 128](#_Toc403633653)

[PEF—Detection of Protozoa By ELISA 135](#_Toc403633654)

[ALA—Alpha -1- Antitrypsin Assay 140](#_Toc403633655)

[MPO—Myeloperoxidase ELISA: In Vitro Determination of Myeloperoxidase in Stool 146](#_Toc403633656)

[NEO—Neopterin ELISA: Quantitative Determination of Neopterin in Stool 152](#_Toc403633657)

[QCB—Quality Control: Bacterial Growth Media 158](#_Toc403633658)

[QCM—Quality Control/Quality Assurance: Microbiology 160](#_Toc403633659)

[**Urine** 161](#_Toc403633660)

[UCF—Urine Collection, Processing, and Transport 162](#_Toc403633661)

[USV—Small Volume Urine Collection, Processing and Transport SOP 168](#_Toc403633662)

[ULM—Lactulose: Mannitol Assay SOP 171](#_Toc403633663)

[UIL—Urinary Iodine Assay SOP 179](#_Toc403633664)

[**Blood** 182](#_Toc403633665)

[BCH/DCF—Blood Collection SOP 183](#_Toc403633666)

[Appendix A: Blood Collection & Hemoglobin (BCH) SOP Flow Chart 191](#_Toc403633667)

[HEM—HemoCue Assay for Determining Hemoglobin Levels in Blood 191](#_Toc403633668)

[BPT—Blood Processing, Transport and Storage 196](#_Toc403633669)

[Micronutrient Assays 201](#_Toc403633670)

[AGP—Alpha-1-Acid Glycoprotein SOP 202](#_Toc403633671)

[HTR—Plasma Human Transferrin Receptor SOP 207](#_Toc403633672)

[FAR—Plasma Ferritin Assay SOP 218](#_Toc403633673)

[ZAR—Plasma Zinc Assay SOP 221](#_Toc403633674)

[RAR—Plasma Retinol Assay SOP (Needs proof reading by Technical subcommittee) 223](#_Toc403633675)

[PGA—Plasma Glutamine and Arginine Assay SOP 226](#_Toc403633676)

[BLL—Blood Lead SOP 229](#_Toc403633677)

[Vaccine Response Assays 230](#_Toc403633678)

[VRT—Detection of Tetanus*-*Specific IgG in human sera/plasma by ELISA 231](#_Toc403633679)

[VRM—Detection of Measles*-*Specific IgG by ELISA 239](#_Toc403633680)

[VRP—Detection of Anti-Bordetella Pertussis Toxin Specific IgG by ELISA 247](#_Toc403633681)

[VRR—Detection of anti-Rotavirus Specific IgA By ELISA 254](#_Toc403633682)

[VRF - Form Guidance for Vaccine Response forms 300](#_Toc403633683)

[**Collection of OTHER Clinical Specimens** 302](#_Toc403633684)

[AXT—Axillary Temperature Recording and Thermometer Calibration 303](#_Toc403633685)

[SAL—Saliva Collection SOP 305](#_Toc403633686)

[BCM – Breast Milk Collection 310](#_Toc403633687)

[BCT—Breast Milk Sample Transport and Processing SOP 313](#_Toc403633688)

[MSC—Maternal Stool Collection, Processing and Transport to the Laboratory 315](#_Toc403633689)

[MSR—Receiving and Storage of Maternal Stool Samples SOP 319](#_Toc403633690)

[**Growth and Nutrition** 325](#_Toc403633691)

[Dietary Assessment Overview 326](#_Toc403633692)

[FRQ/FRS—24 Hour Food Recall SOP 329](#_Toc403633693)

[ANM—Anthropometry Measurements of Children and Mothers 344](#_Toc403633694)

[DBE─Detection of Bipedal Edema SOP 351](#_Toc403633695)

[**Cognitive Development** 353](#_Toc403633696)

[Cognitive Development Overview 354](#_Toc403633697)

[HIT—HOME Inventory (Infant/Toddler Version) 357](#_Toc403633698)

[RCM—Raven’s Combined Progressive Matrices 364](#_Toc403633699)

[SRQ—Self-Reporting Questionnaire 20 369](#_Toc403633700)

[BSD—Bayley Scales of Infant Development 371](#_Toc403633701)

[ITS—Infant Temperament Scale 374](#_Toc403633702)

[MWG—MacArthur Adapted Communicative Development Inventory (CDI): Words and Gestures 379](#_Toc403633703)

[VID—Video Collection SOP 381](#_Toc403633704)

[VFT—Video File Transfer SOP 383](#_Toc403633705)

[**OTHER** 396](#_Toc403633707)

[PDF—Protocol Deviation Form SOP 397](#_Toc403633708)

[AEF—Adverse Event Recording and Reporting SOP 399](#_Toc403633709)

[GPS - Geographic Positioning System SOP (GPS) 402](#_Toc403633710)

[**Double Data Entry and Barcode Sample Tracking** 406](#_Toc403633711)

[BST—Barcode Sample Tracking Database SOP 407](#_Toc403633712)

Introduction to the Manual of Procedures

The success of the MAL-ED Network Project depends, to a very large extent, not only on the collaboration and collegial working relationships of all the scientists, administrators and support staff involved in the project, but also on the use of harmonized protocols and methods. Following common protocols will allow us to compare and contrast study results across sites. The implementation of these common protocols is the real strength of the Network and that which makes the MAL-ED Network unique. Indeed, the usefulness of the data and the samples that we gather and archive depends on this collaborative approach and will be the legacy of this project. This Manual of Procedures (MOP) is intended to serve as a field reference guide on how to conduct our studies and as a source of the most current version of the various forms and procedures in use by all sites in the Network.

It is very important that we strive to make this a living document, one that reflects changes in the technologies available to us in the pursuit of our goals, as well as one that is informed by the experience of the researchers and Study Researcher / Nurse / Fieldworkers who have to translate these protocols and data forms into real work and output. We want to hear your comments on what does and does not work and what needs to be changed. We will make collective decisions based on that information and will ensure that all participants are made aware of changes to the procedures that will affect the way investigators do their work.

We realize that any document as large and diverse in its content as this one, which has been assembled by committees, will not be perfect when it is released to the sites for implementation. It is important for you to send your comments on content or ambiguities that you note to MAL-ED administrative staff at The Foundation for the National Institutes of Health (FNIH) or at the Fogarty International Center, NIH (FIC) to coordinate changes and updates.

Background and Rationale

Malnutrition is considered one of the most prevalent risk factors for morbidity and mortality in children under five. An estimated 20% of children in the developing world are malnourished [1] and poor nutrition is linked to more than half of all child deaths worldwide [2]. Malnutrition in early childhood may lead to cognitive and physical deficits and may cause similar deficits in future generations as malnourished mothers give birth to low birth weight children [3]. In addition, malnutrition increases susceptibility and incidence of infections and is associated with diminished response to vaccines.

The root of malnutrition in early childhood is complex and has a variety of direct and underlying contributors: lack of food, insufficient breastfeeding, inadequate complementary foods, catabolic states due to infection, and inadequate response of the host and the host’s gut microbiome to caloric deficit. But pathogenic bacteria, viruses, and parasites in the gut also impact nutritional status. Firstly, enteric pathogens impair nutrient absorption by damaging the absorptive capacity of the intestine, causing protein-energy and micronutrient malnutrition. Secondly, enteric infections can compromise the intestinal barrier, causing increased intestinal permeability to pathogens, endotoxins, and other macromolecules that can result in the chronic stimulation of the immune system. Importantly, both micronutrient deficiencies and chronic immune stimulation have been found to impair growth and increase susceptibility to infectious diseases [4]. Additionally, altered gut flora and pathogens may also influence the effectiveness of orally-delivered vaccines. Understanding the complex and synergistic relationships between enteric infections and malnutrition, therefore, is fundamental to the design of better intervention strategies.

While it is likely that enteric infections can lead to malnutrition, existing data on enteropathogen etiologies are limited by small sample sizes, limited geographic locales, and robustness of diagnostic tests. Additionally, there has been relatively little study of morbidity and mortality due to chronic and recurrent enteric infectious microorganisms and parasites, their contribution to the global burden of disease in children under five, and the consequential long-term effects in adulthood. To date, there have also been no systematic studies elucidating the relationship between growth/development and specific micro-organisms, incidence rates at specific ages in early childhood, or mixed infections. Furthermore, there have not been demonstrative studies on associations of these factors with intermediary indicators, such as gut dysfunction, which would presumably link enteric infections with growth and development. Finally, there are also limited studies looking at vaccine response associated with mixed infection, and the role of micronutrient supplementation in developing countries.

**Hypotheses, Study Aims, and Methods**

**We hypothesize that infection with certain enteropathogens leads to malnutrition by (1) causing intestinal inflammation and/or by altering intestinal barrier and adsorptive function and by (2) failure of normal growth and development.** In addition to quantifying the associations between enteric infection and malnutrition, our study also aims to shed light on relevant questions such as: (1) which micro-organisms or mixed infections are most relevant to growth faltering and poor development and (2) at what age in early life do specific infections cause the most disruption to growth and development?

In order to investigate these hypotheses, we have established a coordinated network of sites in populations with a high prevalence of malnutrition and enteric infections for a comprehensive study, using shared and harmonized protocols, to determine factors that impact early childhood health and development. Intensive surveillance of children during the first two years of life at the proposed network of sites will allow us to elucidate some of these complex relationships and hopfeully lead to more targeted and cost-effective interventions in the future.

We will undertake prospective longitudinal and case control epidemiological studies to identify the factors associated with a child’s risk of enteric infection, chronic diarrhea, malnutrition, as well as with impaired gut function, vaccine response and development.

**Longitudinal Cohort Studies** – At each site, project community health workers will identify, consent and enroll at least 200 infants at birth. The infants will be followed at regular intervals from birth up to 24 months of age and up to 36 months of age when feasible and as budget and protocols allows. Active surveillance for infectious diseases and general child health will include twice weekly household visits. Using harmonized protocols, each site will perform a number of tests and assessments including those for gut function, anthropometry and cognitive development, micronutrient status, and vaccine responses. The standardized procedures for the longitudinal cohort studies are detailed in this document.

**Case Control Studies–** For the case control studies (in Brazil and Bangladesh), moderate to severely underweight (weight for age Z score (WAZ) <-2) children 6-24 months attending nutrition clinics or from the community will be identified and enrolled. Age, sex, and neighborhood matched controls will also be enrolled over the first 4 years (to reach at least 500 cases and 500 controls at each of the two sites) with quarterly follow-up to be conducted for at least one year thereafter. Please note that the standardized procedures for the case-control studies will be outlined in a companion document.

**It is important that all sites follow the standardized procedures detailed below to ensure that data collected for the longitudinal cohort studies at each site will be comparable with data collected at other sites.**

**References:**

1. Black, R.E., et al., *Maternal and child undernutrition: global and regional exposures and health consequences.* Lancet, 2008. **371**(9608): p. 243-60.

2. Caulfield, L.E., et al., *Undernutrition as an underlying cause of child deaths associated with diarrhea, pneumonia, malaria, and measles.* Am J Clin Nutr, 2004. **80**(1): p. 193-8.

3. Victora, C.G., et al., *Maternal and child undernutrition: consequences for adult health and human capital.* Lancet, 2008.

4. Campbell, D.I., M. Elia, and P.G. Lunn, *Growth faltering in rural Gambian infants is associated with impaired small intestinal barrier function, leading to endotoxemia and systemic inflammation.* J Nutr, 2003. **133**(5): p. 1332-8.

### Abbreviations

| **ID** | **Form/SOP Name** | **Associated Form/SOP ID** |
| --- | --- | --- |
| AEF | Adverse Event Recording and Reporting SOP |  |
| AGP | Alpha 1-acid Glycoprotein |  |
| ALA | Alpha-1 Antitrypsin |  |
| ANM | Anthropometry Measurements SOP | MOA, MOB, ANM, ANT |
| ANT | Anthropometry Check Form SOP | MOA, MOB, ANM, ANT |
| AXT | Axillary Temperature SOP | MOA, MOB |
| BCH | Blood Collection SOP | HEM, BPT |
| BCM | Breast Milk Collection SOP | BCT |
| BCT | Breast Milk Transport and Processing | BCM |
| BDF | Bacteriology Data Form |  |
| BLL | Blood Lead Assay | SOP is not final |
| BPT | Blood Processing, Transport and Storage | BCH, BRF |
| BSD | Bayley Scales of Infant and Toddler Development |  |
| BST | Barcode Sample Tracking Database SOP |  |
| CAF | Baseline Child Assessment Form |  |
| CBQ | Child Behavior Questionnaire | dependent on future funding |
| DAF | Baseline Demographic Form |  |
| DCF | DNA Collection Form |  |
| DPF | DNA Processing Form |  |
| ECP | E. coli PCR form |  |
| FAR | Ferritin Assay Results Form |  |
| FRQ/FRS | 24 Hour Food Recall SOP |  |
| FSE | Follow-up SES Questionnaire |  |
| FSQ | Food Security Questionnaire |  |
| FVF | Fecal Viral Form |  |
| HEM | HemoCue Assay for Determining Hemoglobin Levels in Blood | BCH |
| HIT | HOME: Infant Toddler Version |  |
| HTR | Plasma Human Transferrin Receptor SOP |  |
| IAR | Urinary Iodine Assay Results Form | UIL |
| ITS | Infant Temperament Scale |  |
| MAF | Maternal Assessment Form | BCH, SAL |
| MDF | Microscopy Data Form |  |
| MEP | MPO ELISA Plate Form | MPO |
| MOA | Monthly Combined Form A | ANM, ANT, AXT |
| MOB | Monthly Combined Form B | ANM, ANT, AXT |
| MOC | Monthly Combined Form C | ANM, ANT, AXT |
| MPO | Myeloperoxidase ELISA Form | MEP |
| MSC | Maternal Stool Collection Form | MSR |
| MSR | Receiving and Storage of Maternal Stool Samples SOP | MSC |
| MWG | MacArthur Words and Gestures |  |
| MWS | MacArthur Words and Sentences | dependent on future funding |
| NCF | Non-Continuation Form |  |
| NEO | Neopterin ELISA Form | NEP |
| NEP | NEO ELISA Plate Form | NEO |
| NPF | Non-Participant Form |  |
| NRV | Detection of Noroviruses |  |
| NUR | Nursing Notes |  |
| PDF | Protocol Deviation Form SOP | PDF |
| PEF | Protozoa ELISA form |  |
| PGA | Plasma Glutamine & Arginine Assay Form |  |
| PID | Participant ID Log |  |
| QCB | Quality Control: Bacterial Growth Media |  |
| QCM | Quality Control/Quality Assurance: Microbiology |  |
| QCS | Surveillance Quality Monitoring | MOA, MOB, ANT, XAF |
| QNS | Quantity Not Sufficient Form |  |
| RAR | Retinol Assay Results Form |  |
| RCM | Raven's Combined Matrices |  |
| REF | Referral Form |  |
| RSS | Receiving and Storage of Stool Samples | SRF, SIL, SFC, QNS |
| SAF | Surveillance Assessment Form | XAF |
| SAL | Saliva Collection SOP | MAF |
| SCR | Screening Form |  |
| SCT | Stool Collection, Processing and Transport to Laboratory | SFC, MOA, MOB, SAF |
| SFC | Stool Field Collection Form | SCT |
| SIL | Sample ID Log |  |
| SRF | Stool Receiving Form | RSS |
| SRQ | SRQ-20 |  |
| STS | Specimen Transport and International Shipping SOP |  |
| UCF | Urine Collection & Receiving Form |  |
| UIL | Urinary Iodine Assay SOP | IAR |
| ULM | Urinary Lactulose-Mannitol Lab Form |  |
| USV | Small Volume Urine Collection Form |  |
| VID | Video Collection SOP | BSD, RCM |
| VIF | Vaccine Information Form SOP | VIF |
| VFT | Video File Transfer SOP | VID |
| VR1 | Vaccine Response Laboratory Report Form, 7 Months | VRM, VRT, VRO, VRP, VRT |
| VR2 | Vaccine Response Laboratory Report Form, 15 Months | VRM, VRT, VRO, VRP, VRT |
| VRM | Vaccine Response ELISA measles SOP | VR1, VR2 |
| VRO | Vaccine Response Polio Plaque Assay SOP | VR1, VR2 |
| VRP | Vaccine Response ELISA Pertussis SOP | VR1, VR2 |
| VRR | Vaccine Response ELISA Rotavirus SOP | VR1, VR2 |
| VRT | Vaccine Response ELISA Tetanus SOP | VR1, VR2 |
| XAF | Surveillance Check Form SOP | SAF |
| ZAR | Zinc Assay Results Form |  |

**Recruit, Consent and Enroll**

SCR—Screening Process

I. Purpose

To screen potential participants for entry into cohort.

II. Material

Screening form, Participant ID log, pen, clipboard.

III. Methods

1. Study Researcher / Nurse / Fieldworker will visit households within 17 days of childbirth in order to screen mothers and children for entry into study. Potential participants will be identified through site-specific methods (e.g. census, antenatal clinics) and informal follow up with the pregnant women will occur in order to ensure screening within 17 days of birth.
2. Participant ID (PID) will be assigned at the start of the visit by writing the name and address of the child on the PID log. This PID will be transferred to the top of the screening form.
3. Question guidance

| # | Question | Guidance |
| --- | --- | --- |
|  | Participant ID | Write the participant ID in the space provided at the upper left corner. |
| 01 | Study Researcher / Nurse / Fieldworker ID | The Study Researcher / Nurse / Fieldworker’s unique ID number should be written here. |
| 02 | Today’s date | Today’s date – DD/MMM/YY |
| 03 | Plans to move outside community? | This question asks whether the family plans to move outside the community in the next six months. This includes planned absence from the study area of greater than 30 days if the Study Researcher / Nurse / Fieldworker will be unable to make contact with the caregiver during that time. If the family will move but the Study Researcher / Nurse / Fieldworker can retain contact with the family and collect morbidity information and other data, the child can be enrolled, but if the family plans to be out of the study area for more than 30 days, they should not be enrolled in the study. |
| 04 | Mother <16 years of age? | This question asks the age of the mother. The Study Researcher / Nurse / Fieldworker may already have the age of the mother from the census; in that case, this question will serve to verify the age of the mother. |
| 05 | Mother has another child in MAL-ED study? | This question asks if the mother has another child in the MAL-ED study. The field site should not send a Study Researcher / Nurse / Fieldworker to enroll women who already have a child enrolled in the cohort, but this question serves to verify this information. |
| 06 | Multiple pregnancy? | This question asks if the birth resulted in a single child or multiples (twins, triplets, etc.). |
| 07 | Is the child healthy? | This question asks if the child is healthy. Healthy, for this study, is defined as **not** having:  - Severe disease requiring hospitalization for something other than typical healthy birth  - Severe or chronic condition diagnosed by medical doctor (e.g. neonatal disease, renal disease, chronic heart failure, liver disease, cystic fibrosis, congenital conditions)  - Enteropathies diagnosed by medical doctor  - Weight at birth or enrollment was <1500 g |
| 08 | Is the mother able to give informed consent? | This question asks if the mother is living and available to provide informed consent. |

The child is eligible to participate in the study if:

1. The answer to questions 3, 4, 5, and 6 is No, &
2. The answer to questions 7 and 8 is Yes.

If the child is eligible to participate, continue on to the consent form, or if consent has already been obtained, continue to the Child Assessment Form (CAF).

If the child is **not** eligible to participate (answer to question 3, 4, 5, or 6 is Yes, or answer to question 7 or 8 is No) or if the caregiver is unwilling to answer any of the screening questions, the child **cannot** be enrolled in the study and the Non-Participation Form (NPF) should be filled out with the caregiver.

IV. Notes

- Women do not need to have delivered in order to give consent; however, sites may wish to delay the consenting process until after screening is complete (after birth) in order to avoid consenting women who ultimately will not be enrolled in the study.
- The mother needs to be living and able to give consent in order for the child to be enrolled in this study.

V. General QC instructions

- 100% of each Study Researcher / Nurse / Fieldworker’s forms should be reviewed by the supervisor on at least a weekly basis, ideally, at the end of each working day. Supervisors should ensure the forms are complete (no missing fields), that the data appear to be correct, and that visits planned for the day were made.

- Corrections to the forms should be minimized (data should be correct the first time), but if needed, the Study Researcher / Nurse / Fieldworker should follow proper correction procedures. If form corrections are necessary:

- - 1. Cross through the incorrect information once,
    2. Write the correct information,
    3. Write the date the correction was made, and
    4. Write their initials.

- Make sure forms are kept confidential and protected (in a locked file drawer, for example) when they are returned from the field.

- Data center transmission of forms

- - Once forms have been completely filled out by the Study Researchers / Nurses / Fieldworkers and reviewed by the supervisor, they must be delivered within 48 hours to the local data center for data entry. Data entry of the forms should occur within one month of delivery to the data center (preferably sooner).

PID—Participant ID Log SOP

I. Purpose

To assign unique confidential participant IDs (PIDs) to all screened families.

II. Material

Participant ID log, pen, clipboard

III. Methods

1. Study Researcher / Nurse / Fieldworker will visit households within 17 days of childbirth in order to screen mothers and children for entry into study and obtain consent.
2. Participant ID (PID) will be assigned at the start of the visit by writing the name (last, first) and address of the child on the PID log. This PID will be transferred to the top of all forms filled out for the child.
3. The PID log is the link between the PID and the participant’s names and addresses. As such, it should be guarded carefully for the participant’s privacy and for the study’s ability to identify participants.
4. The PID log information will be stored locally in a secure and locked place and will **not** be uploaded to the central database.

IV. Notes

- What is the PID?

Digits 1 & 2 – Indicate country (PE-Peru, BR-Brazil, Bangladesh-BG, Nepal-NP, Pakistan-PK, India-IN, Tanzania-TZ, South Africa-SA)

Digit 3 – Indicates site # (most countries have only one study site at this time)

Digit 4 – Indicates family member – C=Child, M=Mother

Digits 5-8 – Indicates the household number 0001-9999

- In the cohort, the PID Log is the only data collection form that will include names and addresses. The PID will be used to identify participants on all other study forms.

NPF—Non-Participant Form SOP

I. Purpose

To collect some basic information on the mothers / children who were not eligible for the study or refused to participate.

II. Material

Non-Participant form, Participant ID log, pen, clipboard

III. Methods

If the child was not enrolled in the study, either because the family refused to participate or because they did not pass the screening process, this form must be filled out. Start by asking for verbal consent to ask some questions – if consent is given, you may continue with the form. If no consent is given, fill in only the first three questions. If no Participant ID has been assigned (mother refused to go through screening process), document the child’s name (or, if the woman is still pregnant, the mother’s name) and address on the Participant ID log and transfer the PID to the top of the NPF. If the mother does not want to answer any of the questions, fill in ‘NA’.

Some sites may want to collect additional information about why the individual refused to participate. In addition, some sites may want to have supervisors perform follow up visits to families that refuse to participate so that the supervisor can provide additional information about the study.

IV. General QC instructions

- 100% of each Study Researcher / Nurse / Fieldworker’s forms should be reviewed by the supervisor on at least a weekly basis, ideally, at the end of each working day. Supervisors should ensure the forms are complete (no missing fields), that the data appear to be correct, and that visits planned for the day were made.

- Corrections to the forms should be minimized (data should be correct the first time), but if needed, the Study Researcher / Nurse / Fieldworker should follow proper correction procedures. If form corrections are necessary:

- - 1. Cross through the incorrect information once,
    2. Write the correct information,
    3. Write the date the correction was made, and
    4. Write their initials.

- Make sure forms are kept confidential and protected (in a locked file drawer, for example) when they are returned from the field.

- Data center transmission of forms

- - Once forms have been completely filled out by the Study Researchers / Nurses / Fieldworkers and reviewed by the supervisor, they must be delivered within 48 hours to the local data center for data entry. Data entry of the forms should occur within one month of delivery to the data center (preferably sooner).

| # | Question | Guidance |
| --- | --- | --- |
|  | Participant ID | Write the child’s participant ID in the space provided at the upper left corner. |
| 01 | Study Researcher / Nurse/Fieldworker ID | Enter the Study Researcher / Nurse / Fieldworker’s unique ID number here. |
| 02 | Today’s date | Format DD/MMM/YY |
| 03 | Reason for not participating | Enter the reason for not participating here. Additional information can be entered into the notes section on the bottom of the form. |
| 04 | Mother’s age | If verbal consent is given, continue with these questions on the form. This is the age of the child’s mother. If she does not know her exact age ask for an estimate, and enter that number. |
| 05 | Number of children | This question asks about the number of live births a woman has had, not including the most recent one. Include only live births, rather than all pregnancies. |
| 06 | Drinking water | The purpose of this question is to assess the cleanliness of the household drinking water by asking about the household’s main source of water. If drinking water is obtained from several sources, probe to determine the source from which the household obtains the majority of its drinking water. If the source varies by season, record the main source used at the time of interview. Below are explanations of each of the response categories.  **Piped into dwelling:** Pipe connected with in-house plumbing to one or more taps, e.g. in the kitchen and bathroom. Sometimes called a house connection.  **Piped to yard/plot:** Pipe connected to a tap outside the house in the yard or plot. Sometimes called a yard connection.  **Public tap or standpipe:** Public water point from which community members may collect water. A standpipe may also be known as a public fountain or public tap. Public standpipes can have one or more taps and are typically made of brickwork, masonry or concrete.  **Tubewell or borehole:** A deep hole that has been driven, bored or drilled with the purpose of reaching ground water supplies. Water is delivered from a tubewell or borehole through a pump which may be human, animal, wind, electric, diesel or solar-powered.  **Protected well:** A well that is (1) protected from runoff water through a well lining or casing that is raised above ground level and a platform that diverts spilled water away from the well and (2) covered so that bird droppings and animals cannot fall down the hole. Both conditions must be observed for a dug well to be considered as protected.  **Unprotected well:** A well which is unprotected from runoff water; 2) unprotected from bird droppings and animals; or (3) both.  **Protected spring:** A spring protected from runoff, bird droppings, and animals by a “spring box” which is typically constructed of brick, masonry, or concrete and is built around the spring so that water flows directly out of the box into a pipe without being exposed to outside pollution.  **Unprotected spring:** A spring that is subject to runoff and/ or bird droppings or animals. Unprotected springs typically do not have a “spring box”.  **Rainwater:** Rain that is collected or harvested from surfaces by roof or ground catchment and stored in a container, tank or cistern.  **Tanker truck:** Water is obtained from a provider who uses a truck to transport water into the community. Typically the provider sells the water to households.  **Cart with small tank:** Water is obtained from a provider who transports water into a community using a cart and then sells the water. The means for pulling the cart may be motorized or non-motorized (e.g., a donkey).  **Surface water:** Water located above ground and includes rivers, dams,  lakes, ponds, streams, canals, and irrigation channels  **Bottled water:** Water that is bottled and sold to the household in bottles. |
| 07 | Toilet facility | The purpose of this question is to obtain a measure of the sanitation level of the household, since toilet facilities are important for disease control and health improvement. Below are some definitions for the terms used in the codes:  **Flush/pour flush toilet:** A flush toilet uses a cistern or holding tank for flushing water and has a water seal, which is a U-shaped pipe, below the seat or squatting pan that prevents the passage of flies and odors. A pour flush toilet uses a water seal, but unlike a flush toilet, a pour flush toilet uses water poured by hand for flushing (no cistern is used).  - **Flush** **to piped sewer system:** A system of sewer pipes (also called sewerage), that is designed to collect human excreta (feces and urine) and wastewater and remove them from the household environment. Sewerage systems consist of facilities for collection, pumping, treating and disposing of human excreta and wastewater.  - **Flush** **to septic tank:** An excreta collection device consisting of a watertight settling tank normally located underground, away from the house or toilet.  - **Flush** **to pit latrine**: A system that flushes excreta to a hole in the ground.  - **Flush** **to somewhere else:** A system in which the excreta is deposited in or nearby the household environment in a location other than a sewer, septic tank, or pit, e.g., excreta may be flushed to the street, yard/plot, drainage ditch or other location.  **- Flush, don’t know where:** The toilet flushes but the participant does not know where the waste goes.  **Pit latrine:** Excreta is deposited without flushing directly into a hole in the ground  **- Ventilated improved pit latrine (VIP):** A latrine ventilated by a pipe extending above the latrine roof. The open end of the vent pipe is covered with gauze mesh or fly-proof netting and the inside of the superstructure is kept dark.  - **Pit latrine with slab**: A latrine with a squatting slab, platform or seat firmly supported on all sides which is raised above the surrounding ground level to prevent surface water from entering the pit and for ease of cleaning.  - **Pit latrine without slab/open pit:** A latrine without a squatting slab, platform or seat. An open pit is a rudimentary hole in the ground where excreta is collected.  **Composting toilet:** A toilet into which excreta and carbon-rich material are combined (vegetable wastes, straw, grass, sawdust, ash) and special conditions maintained to produce inoffensive compost.  **Bucket toilet:** Involves the use of a bucket or other container for the retention of feces (and sometimes urine and anal cleaning material), which is periodically removed for treatment or disposal.  **No facility/bush/field:** The household has no facility and/or uses the bush or a field near the household.  **Public toilet:** Household members use a public toilet.  **Other:** Any other option that does not fall into one of these categories. |
| 08 | Primary way to distinguish population | This question asks about the *primary* method for distinguishing between members of the population. While more than one of these categories might apply, emphasize that you would like to know which of the categories is used most often. For questions 9-13, only ask the question that corresponds to the response to question 8. For example, if the respondent states that *language spoken (04)* is the primary way of distinguishing members of her/his population, then only ask question 12 and write ‘NA’ for questions 9, 10, 11 and 13. |
| 09 | Caste | Only ask this question if the response to question 8 was *caste (01).* |
| 10 | Race | Only ask this question if the response to question 8 was *race (02).* |
| 11 | Tribe | Only ask this question if the response to question 8 was *tribe (03).* |
| 12 | Language spoken | Only ask this question if the response to question 8 was *language spoken (04).* |
| 13 | Religion | Only ask this question if the response to question 8 was *religion (05).* |

Consenting Process

1. Purpose

To obtain informed consent from the parent/guardian in accordance with the guidelines of human study research.

1. Material

IRB-Approved Informed Consent Form in local language, Pen Clipboard,

1. Methods
2. The Study Researcher/Nurse/Fieldworker (FW) uses the Screening Form (SCR) to visit the homes with newborn babies (or when the mother is in the end of her 3^rd^ trimester). If the mother is eligible, the FW obtains signed consent from the parent or guardian using the site-specific Informed Consent Form. Consent is obtained as close to birth as possible.
3. The consent form is of utmost importance and must be signed or, in the case of illiterate persons, a thumbprint must be obtained before any study procedure is performed. The FW explains who the study team is and why we are there, maintaining a good rapport with the parent/guardian and observing whether the words are being understood well.
4. The FW carefully explains the details of the study to the parents or guardians of the participant, who will be asked to read, understand and sign an informed consent form approved by a local institutional review board (IRB) prior to any study-related evaluations being performed. Any questions will be clarified prior to obtaining consent. In the case of illiterate persons, the consent form will be read to him/her in the presence of a witness and a digital impression (thumbprint) will be obtained in place of a signature. The witness cannot be associated with the study and is not needed for literate persons.
5. Information obtained will always be repeated in order to verify that the person understood. It will be made clear that the person is autonomous and free to leave the study at his or her wish at any time.
6. The FW will explain the importance of the accuracy of the information obtained to the subject’s parent or guardian, reinforcing the need to closely follow and accurately report their subject’s symptoms.
7. The FW will inform the parent or guardian of the subject of the discomforts that may occur, and the benefits anticipated, and assure them of our respect for their privacy and confidentiality.
8. After the FW is confident that the parent/guardian understands the study, they are asked to sign the consent form. The signatures and signature dates of the responsible parent /guardian, witness (if needed) and the FW are hand-written on the consent form. A copy of the consent form is given to the parent/guardian.
9. Completed consent forms are placed in the subject’s folder, which is kept locked and is accessible only to the members of the study team.
10. Documentation

All signatures and dates are made on the consent form. These are to be hand-written, original signatures and dates. If the parent/guardian is not able to write the date or their name, the FW obtaining the consent will write the following statements:

Parent/guardian of the child is not able to write her name so a thumbprint is made for her, the FW prints name.

1. Verification

Signatures and dates written on the Consent Forms are checked for completion and verification with PID# recorded on the Screening Form (SCR).

CAF—Child Assesment Form SOP

I. Purpose

To collect baseline demographic, feeding, and anthropometric information.

II. Material

Child Assessment Form (CAF), Anthropometry SOP and equipment, pen

III. Methods

1. A Study Researcher / Nurse / Fieldworker trained in measuring anthropometry will administer this form:
   1. After screening has occurred and consent has been obtained
   2. Within 17 days of birth
2. Before starting this form, request hospital or doctor’s record of the birth, if available.

IV. General QC instructions

- 100% of each Study Researcher / Nurse / Fieldworker’s forms should be reviewed by the supervisor on at least a weekly basis, ideally, at the end of each working day. Supervisors should ensure the forms are complete (no missing fields), that the data appear to be correct, and that visits planned for the day were made.

- Corrections to the forms should be minimized (data should be correct the first time), but if needed, the Study Researcher / Nurse / Fieldworker should follow proper correction procedures. If form corrections are necessary:

- - 1. Cross through the incorrect information once,
    2. Write the correct information,
    3. Write the date the correction was made, and
    4. Write their initials.

- Make sure forms are kept confidential and protected (in a locked file drawer, for example) when they are returned from the field.

- Data center transmission of forms

- - Once forms have been completely filled out by the Study Researchers / Nurses / Fieldworkers and reviewed by the supervisor, they must be delivered within 48 hours to the local data center for data entry. Data entry of the forms should occur within one month of delivery to the data center (preferably sooner).

- Standard rounding techniques are recommended: Round down if last digit is <5 and round up if the last digit is >5).

| # | Question | Guidance |
| --- | --- | --- |
|  | Participant ID | Write the child’s participant ID in the space provided at the upper left corner. |
| 01 | Study Researcher / Nurse / Fieldworker ID | Enter the Study Researcher / Nurse / Fieldworker’s unique ID number here. |
| 02 | Today’s date | Format DD/MMM/YY |
| 03 | Screen completed and consent obtained | Prior to beginning this form, the Child Screening Form (SCR) should be completed and Consent should be obtained. |
| 04 | Date of birth | This question asks about the child’s date of birth. If there are hospital or doctor’s records, they can be used to verify the mother’s information. If the mother is uncertain about the date, the Study Researcher / Nurse / Fieldworker can ask locally relevant ways to determine how many days have passed since the birth. |
| 05 | Gender of child | Enter here whether the enrolled child is male or female. |
| 06 | Birth weight | If birth weight is documented in available medical records, write the birth weight (in kilograms) on the form. If records are not available or the birth weight is not recorded, ask the mother if she knows the birth weight. If so, write it on the form. If there are no hospital / doctor records and the mother does not know the birth weight, write NA. |
| 07 | How long after childbirth did the mother begin breastfeeding the child? | This question asks about how much time passed after birth before the child was first breastfed. If the mother says the child was first breastfed immediately after birth or sometime within one hour, write 00 on the form. If the child was not breastfed in the first hour, but was first breastfed between 1 and 24 hours after birth, write 01 on the form. If the child was first breastfed 1-3 days after birth, write 02. If the child was first breastfed 4 or more days after birth, write 03. If the child has not yet been breastfed at all, write NA. |
| 08 | Was the child fed the first milk (colostrum)? | The first milk is the milk that is produced immediately after childbirth. |
| 09 | Was there pre-lacteal feeding of the child? | This question asks whether the child was given anything before receiving the first breast milk. |
| 10 | Current weight | Enter here the value obtained by weighing the child in kilograms to two decimal places. *Refer to SOP on anthropometry*. |
| 11 | Current length | Enter here the value obtained by measuring the child’s height in centimeters to one decimal place. *Refer to SOP on anthropometry*. |
| 12 | Current head circumference | Enter here the value obtained by measuring the child’s head circumference in centimeters to one decimal place. *Refer to SOP on anthropometry*. |

**Demographic, SES and Food Security Assessments**

DAF—Baseline Demographic Questionnaire

I. Purpose

The Baseline Demographic Questionnaire (DAF) form will be implemented at each household at 0 months. The mother of the child enrolled in the MAL-ED study must be present and willing to respond in order to complete this survey for the household.

II. Material

Baseline Demographic Questionnaire Form (DAF), pen and clipboard.

III. Methods

1. The first 8 questions should be answered by the head of household, if this person is different from the child’s mother. The remaining questions should be answered by the child’s mother. Mother is defined as the biological mother of the child, or the woman who gave birth to the child, not someone acting as a caretaker who may consider herself the child’s mother. If the mother is the head of household, skip questions 1-10 and begin with question 11. If the head of household is unavailable, skip to question 11 and begin with the mother.
2. Question Guidance

| # | Question | Guidance |
| --- | --- | --- |
|  | Participant ID | Write the child’s participant ID in the space provided at the upper left corner. |
| 1 | Study Researcher / Nurse / Fieldworker ID | Enter the Study Researcher / Nurse / Fieldworker’s unique ID number here. |
| 2 | Today’s date | Format DD/MMM/YY |
| 3 | What is your age? | This is the age of the head of household in years. If the head of household does not know her or his exact age ask for an estimate, and enter that number. |
| 4 | *(Record sex)* | Record the sex of the head of household. You can answer this question using observation rather than asking it. |
| 5 | What is your relationship to [CHILD’S NAME]? | This is the head of household’s relationship to the study child, whose height and weight you will be measuring. Refer to the child by name when asking this question. |
| 6 | Are you currently married, divorced, widowed, or never married? *If never married, skip to question 8.* | For this question, married is defined as either legally married or cohabiting with a partner. Similarly, divorced includes legally divorced or no longer cohabiting with a partner. |
| 7 | How old were you when you got married for the first time? | This question asks the age of the head of household when she or he was first married. Again, marriage includes cohabiting with a partner as well as legal marriage. |
| 8 | Have you ever attended school?  *If no, skip to question 11.* | This question refers to formal school only, and includes any formal schooling beginning with kindergarten. |
| 9 | How many years of schooling have you completed? | This question is asking about years of schooling completed, beginning with kindergarten and through any university or post-university education. If the respondent began a year of school but did not complete it, do not include that year. If the respondent repeated any years of schooling, only include that grade level once. For example, if the respondent repeated 5^th^ grade, include that as 1 year only, rather than 2 years. |
| 10 | *If younger than 25 years*  *old:* Are you currently attending school or college? | This question refers to formal school only, but might include technical schools as well as university programs. |
| 11 | How old are you? | This is the age of the child’s mother. If she does not know her exact age ask for an estimate, and enter that number. |
| 12 | Are you currently married, divorced, widowed, or never married? | For this question married is defined as either legally married or cohabiting with a partner. Similarly, divorced includes legally divorced or no longer cohabiting with a partner. |
| 13 | How old were you when you got married for the first time? | This question asks the age of the mother when she was first married. Marriage includes cohabiting with a partner as well as legal marriage. |
| 14 | What is your relationship to [NAME OF HEAD OF HOUSEHOLD]? | This question asks about the mother’s relationship to the head of the household, who answered questions 1-10. If the mother is the head of household, enter “self” (11) as the response. Otherwise, the answer should specify the mother’s role in the relationship. For example, if she is the daughter of the head of household enter ‘02’. |
| 15 | Have you ever attended school?  *If no, skip to question 18.* | This question refers to formal school only, and includes any formal schooling beginning with kindergarten. |
| 16 | How many years of schooling have you completed? | This question is asking about years of schooling completed, beginning with kindergarten and through any university or post-university education. If the respondent began a year of school but did not complete it, do not include that year. If the respondent repeated any years of schooling, only include that grade level once. For example, if the respondent repeated 5^th^ grade, include that as 1 year only, rather than 2 years. |
| 17 | *If younger than 25 years old:* Are you currently attending school or college? | This question refers to formal school only, but might include technical schools as well as university programs. |
| 18 | What is the last name of the father of your child? | This question asks about the surname of the child’s father. If the mother does not know this information, enter NA. If she is not sure of the spelling, or is illiterate, the FW should ask her to state the name, and attempt to spell it as closely as possible. |
| 19 | Does your household pay any domestic workers? | This question is asking about hired domestic workers that the household pays regularly and who sleep in the household. It does not include family members who do unpaid domestic work. |
| 20 | How old were you when you first became pregnant? | This question asks about the mother’s age when she became pregnant for the first time. In this case, include all pregnancies, whether or not they resulted in live births. |
| 21 | How many pregnancies have you had in your lifetime? | This question asks about the number of pregnancies a woman has had in her lifetime. Include all pregnancies, whether or not they resulted in live births. |
| 22 | How many live births have you had in your lifetime? | This question asks about the number of live births a woman has had in her lifetime. Include only live births, rather than all pregnancies. |
| 23 | Are all of these children still alive? *If yes, skip the next question.* | This question asks about whether all children born alive to the mother, given in response to question 22, are still living. If the answer is yes, the questionnaire is complete. |
| 24 | How many children have died? | This question asks the mother about the number of children who were born alive (question 22), but who later died. |

FSQ—Food Security Questionnaire

I. Purpose

The Food Security Questionnaire (FSQ) will be implemented at each household at the 0, 6, 12, 18, 24, 30 and 36 month study visits—30 & 36 month visits if resources and protocols allow. At the 0 month study visit, the FSQ will be administered with the Baseline Demographic Questionnaire (DAF); at the follow-up visits, the FSQ will accompany the SES Assessment Form (FSE). These questions can be answered by the mother of the child, the child’s primary caregiver, or the head of household. However, it is important that the same person respond each time this questionnaire is administered.

II. Material

Food Security Questionnaire Form (FSQ), pen and clipboard.

III. Methods

1. Each of the questions in the Food Security section is asked with a recall period of four weeks (30 days). The respondent is first asked a frequency-of-occurrence to determine whether the condition happened rarely (once or twice), sometimes (three to ten times) or often (more than ten times) in the past four weeks.
2. Some of the nine questions inquire about the respondents’ perceptions of food vulnerability or stress (e.g., did you worry that your household would not have enough food?) and others ask about the respondents’ behavioral responses to insecurity (e.g., did you or any household member have to eat fewer meals in a day because there was not enough food?). The questions address the situation of all household members and do not distinguish adults from children or adolescents. All of the questions ask whether the respondent or other household members either felt a certain way or performed a particular behavior over the previous four weeks.
3. The questionnaire should be asked in its entirety. Project staff should avoid picking and choosing only certain questions. Research has shown that the complete set of questions does a better job of distinguishing the household food insecurity (access) level than any question on its own.
4. Question Guidance

| # | Question | Response |
| --- | --- | --- |
|  | Participant ID | Write the child’s participant ID in the space provided at the upper left corner. |
| 1 | Study Researcher / Nurse / Fieldworker ID | Enter the Study Researcher / Nurse / Fieldworker’s unique ID number here. |
| 2 | Today’s date | Format DD/MMM/YY |
| 3 | In the past four weeks, did you worry that your household would not have enough food? | This question asks the respondent to report their personal experience with uncertainty and anxiety about acquiring food during the previous month. The interviewer should explain that household is defined as the people who usually sleep in the household. Mention that this definition of household applies to all the questions with that term. |
| 4 | In the past four weeks, were you or any household member not able to eat the kinds of foods you preferred because of a lack of resources? | One domain of food insecurity (access) is having limited choices in the type of food that a household eats. This question asks whether any household member was not able to eat according to their preference due to a lack of resources. Preference can refer to the form of a particular food (i.e., whole rice vs. broken rice), type of staple (i.e., millet vs. corn) or a high quality food (i.e., a piece of meat or fish). Preferred foods may or may not be nutritionally high quality. The interviewer should also read the definition of a “lack of resources.” Mention that this definition of household applies to all the questions with that term. The respondent needs to answer on behalf of all household members |
| 5 | In the past four weeks, did you or any household member have to eat a limited variety of foods due to a lack of resources? | This question asks about dietary choices related to variety – i.e., whether the household had to eat an undesired monotonous diet (little diversity in the different types of foods consumed). The respondent needs to answer on behalf of all household members. |
| 6 | In the past four weeks, did you or any household member have to eat some foods that you really did not want to eat because of a lack of resources to obtain other types of food? | This question, which also captures the dimension of limited choices, asks whether any household member had to eat food that they found socially or personally undesirable due to a lack of resources. Often these are foods or food preparations that are consumed only under hardship. Different people may consider different foods to be undesirable, so it is best not to provide examples here at first. The respondent needs to answer on behalf of all household members, according to his or her own perception of the types of food household members ate during the previous four weeks. For all questions, it is important to remind respondents that the examples are not an exhaustive list. |
| 7 | In the past four weeks, did you or any household member have to eat a smaller meal than you felt you needed because there was not enough food? | This question asks whether the respondent felt that the amount of food (any kind of food, not just the staple food) that any household member ate in any meal during the past four weeks was smaller than they felt they needed due to a lack of resources. The respondent should answer according to his or her perception of what constitutes enough food for the needs of the household members. The respondent needs to answer on behalf of all household members. |
| 8 | In the past four weeks, did you or any other household member have to eat fewer meals in a day because there was not enough food? | This question asks whether any household member, due to lack of food, had to eat fewer meals than the number typically eaten in the food secure households in their area. The respondent needs to answer on behalf of all household members. |
| 9 | In the past four weeks, was there ever no food to eat of any kind in your household because of lack of resources to get food? | This question asks about a situation in which the household has no food to eat of any kind in the home. This describes a situation where food was not available to household members through the households’ usual means (e.g., through purchase, from the garden or field, from storage, etc.). |
| 10 | In the past four weeks, did you or any household member go to sleep at night hungry because there was not enough food? | This question asks whether the respondent felt hungry at bedtime because of lack of food or whether the respondent was aware of other household members who were hungry at bedtime because of lack of food. The respondent needs to answer on behalf of all household members. |
| 11 | In the past four weeks, did you or any household member go a whole day and night without eating anything because there was not enough food? | This question asks whether any household member did not eat from the time they awoke in the morning to the time they awoke the next morning due to lack of food. The respondent needs to answer on behalf of all household members. |

FSE—Followup SES Questionnaire

I. Purpose

The Followup Socioeconomic Status Form (FSE) will be administered in each MAL-ED household every six months starting when the infant is six months old (i.e. 6, 12, 18, 24, 30, 36 month visits—30 & 36 month visits if resources and protocols allow).

II. Material

Followup Socioeconomic Status Form (FSE), pen and clipboard.

III. Methods

The following people should respond to the questions on this form: head of household, mother of the child, and primary caregiver (if different from the mother).

- **Head of household** is defined as the person who contributes most financially to the household.
- **Mother** is defined as the biological mother of the child, or the woman who gave birth to the child, not someone acting as a caregiver who may consider herself the child’s mother.
- **Primary caregiver** is the person who most often cares for the child, including taking care of the child when he/she is sick, feeding the child, etc.

The first 8 questions should be answered by the head of household, if this person is different from the child’s mother or primary caregiver. Questions 9-20 are for the mother of the child. If the mother is deceased skip to question 21. If the mother is unavailable, return to the household at another time to complete these questions. If the mother is alive, it is important that she respond to questions 9-20, even if she is not the child’s primary caregiver. Questions 21-28 should be addressed to the child’s primary caregiver. *Complete these questions only if the mother is not the primary caregiver.*  If the mother is the primary caretaker skip to question 29.The remaining questions should be answered by the child’s primary caregiver. Questions 29-88 should be answered by the child’s primary caregiver.

**Header information**

The first section, including date, fieldworker ID and child ID should be completed by the fieldworker once the mother has consented to participate.

| # | Question | Guidance |
| --- | --- | --- |
| **DEMOGRAPHIC QUESTIONS** | | |
| *Questions for head of household (if mother/primary caregiver of child is the head of household skip to question 9)* | | |
| 1 | What is your age? | This is the age of the head of household in years. If the head of household does not know her or his exact age ask for an estimate, and enter that number. |
| 2 | *(Record sex)* | Record the sex of the head of household. You can answer this question using observation rather than asking it. |
| 3 | What is your relationship to [CHILD’S NAME]? | This is the head of household’s relationship to the infant/child. Refer to the infant/child by name when asking this question. |
| 4 | Are you currently married, divorced, widowed, or never married?  *If never married, skip to question 6.* | For this question, married is defined as either legally married or cohabiting with a partner. Similarly, divorced includes legally divorced or no longer cohabiting with a partner. |
| 5 | How old were you when you got married for the first time? | This question asks the age of the head of household when she or he was first married. Again, marriage includes cohabiting with a partner as well as legal marriage. |
| 6 | Have you ever attended school?  *If no, skip to question 9.* | This question refers to formal school only, and includes any formal schooling beginning with kindergarten. |
| 7 | How many years of schooling have you completed? | This question is asking about years of schooling completed, beginning with kindergarten and through any university or post-university education. If the respondent began a year of school but did not complete it, do not include that year. If the respondent repeated any years of schooling, only include that grade level once. For example, if the respondent repeated 5^th^ grade, include that as 1 year only, rather than 2 years. |
| 8 | *If younger than 25 years old:* Are you currently attending school or college? | This question refers to formal school only, but might include technical schools as well as university programs. |
| *Questions 9-20 are for the mother of the child. If the mother is deceased skip to question 21. If the mother is unavailable, return to the household at another time to complete these questions. Complete these questions even if the mother is not the primary caregiver for the enrolled child.* | | |
| 9 | What is your age? | This is the age of the child’s mother. If she does not know her exact age ask for an estimate, and enter that number. |
| 10 | Are you currently married, divorced, widowed, or never married?  *If never married, skip to question 12.* | For this question married is defined as either legally married or cohabiting with a partner. Similarly, divorced includes legally divorced or no longer cohabiting with a partner. |
| 11 | How old were you when you got married for the first time? | This question asks the age of the mother when she was first married. Marriage includes cohabiting with a partner as well as legal marriage. |
| 12 | Have you ever attended school?  *If no, skip to question 15.* | This question refers to formal school only, and includes any formal schooling beginning with kindergarten. |
| 13 | How many years of schooling have you completed? | This question is asking about years of schooling completed, beginning with kindergarten and through any university or post-university education. If the respondent began a year of school but did not complete it, do not include that year. If the respondent repeated any years of schooling, only include that grade level once. For example, if the respondent repeated 5^th^ grade, include that as 1 year only, rather than 2 years. |
| 14 | *If younger than 25 years old:* Are you currently attending school or college? | This question refers to formal school only, but might include technical schools as well as university programs. |
| 15 | How old were you when you first became pregnant? | This question asks about the mother’s age when she became pregnant for the first time. In this case, include all pregnancies, whether or not they resulted in live births. |
| 16 | How many pregnancies have you had in your lifetime? | This question asks about the number of pregnancies a woman has had in her lifetime. Include all pregnancies, whether or not they resulted in live births. |
| 17 | How many live births have you had in your lifetime? | This question asks about the number of live births a woman has had in her lifetime. Include only live births. This number should be less than or equal to the number of pregnancies. |
| 18 | Are all of these children still alive?  *If yes, skip to question 20.* | This question asks about the number of children who are still alive, out of the number of live births. This number should be less than equal to the number of live births. |
| 19 | How many children have died? | This question asks about the number of children, out of the live births, who have died. For many women the answer might be 0. Otherwise this number should be less than or equal to the number of live births from question 17. |
| 20 | Are you the primary caregiver for [CHILD’S NAME]? | The primary caregiver is the person who most often cares for the child, including taking care of the child when he/she is sick, feeding the child, etc. If the answer is no, ask questions 21-28 to the child’s primary caregiver. Is the answer is yes, skip questions 21-28 and continue with question 29. |
| *Questions 21-28 should be addressed to the child’s primary caregiver. Complete these questions only if the mother is not the primary caregiver. If the mother is the primary caregiver skip to question 29.* | | |
| 21 | What is your relationship to [CHILD’S NAME]? | This is the primary caregiver’s relationship to the infant/child. Refer to the infant/child by name when asking this question. |
| 22 | How old are you? | This is the age of the child’s primary caregiver. If he or she does not know his/her exact age ask for an estimate, and enter that number. |
| 23 | Are you currently married, divorced, widowed, or never married?  *If never married, skip to question 25.* | For this question married is defined as either legally married or cohabiting with a partner. Similarly, divorced includes legally divorced or no longer cohabiting with a partner. |
| 24 | How old were you when you got married for the first time? | This question asks the age of the primary caregiver when she/he was first married. Marriage includes cohabiting with a partner as well as legal marriage. |
| 25 | What is your relationship to [NAME OF HEAD OF HOUSEHOLD]? | This question asks about the relationship of the caregiver to the head of household. |
| 26 | Have you ever attended school?  *If no, skip to question 29.* | This question refers to formal school only, and includes any formal schooling beginning with kindergarten. |
| 27 | How many years of schooling have you completed? | This question is asking about years of schooling completed, beginning with kindergarten and through any university or post-university education. If the respondent began a year of school but did not complete it, do not include that year. If the respondent repeated any years of schooling, only include that grade level once. For example, if the respondent repeated 5^th^ grade, include that as 1 year only, rather than 2 years. |
| 28 | *If younger than 25 years old:* Are you currently attending school or college? | This question refers to formal school only, but might include technical schools as well as university programs. |
| **SOCIO-ECONOMIC STATUS QUESTIONS** | | |
| 29 | How long has your family lived in this house? | This question asks about the length of time that the child’s family has lived in their current home. If the head of household’s family lived in the home prior to the child’s mother living there, record the length of time that the head of households family has lived in the home. The goal is to understand how long the extended family of the child and lived in this home, not the child or mother specifically. |
| 30 | How many rooms are there in your house? | The total number of rooms in the house includes the veranda, the living and sleeping rooms, as well as attached kitchens used only by this household. It excludes storerooms and separate kitchens that are shared with other families/households. |
| 31 | How many rooms in this household are used for sleeping? | This question is seeking information on which rooms in the house are usually used for people to sleep. For example, this would not include the kitchen or toilet facilities. |
| 32 | How many people usually sleep in this household? | This question asks about the number of people who usually sleep in this household on an average night. |
| 33 | Does your household pay any domestic workers? | Domestic workers are defined as non-family members who work in the household for pay. |
| 34 | What is the main source of drinking water for members of your household?  *If answered 04, 05, 06, 07, or 08 skip to question 37.* | The purpose of this question is to assess the cleanliness of the household drinking water by asking about the household’s main source of water. If drinking water is obtained from several sources, probe to determine the source from which the household obtains the majority of its drinking water. If the source varies by season, record the main source used at the time of interview. Below are explanations of each of the response categories.  **01= Piped into dwelling:** Pipe connected with in-house plumbing to one or more taps, e.g. in the kitchen and bathroom. Sometimes called a house connection.  **02= Piped to yard/plot:** Pipe connected to a tap outside the house in the yard or plot. Sometimes called a yard connection.  **03= Public tap or standpipe:** Public water point from which community members may collect water. A standpipe may also be known as a public fountain or public tap. Public standpipes can have one or more taps and are typically made of brickwork, masonry or concrete.  **04= Tubewell or borehole:** A deep hole that has been driven, bored or drilled with the purpose of reaching ground water supplies. Water is delivered from a tubewell or borehole through a pump which may be human, animal, wind, electric, diesel or  solar-powered.  **05= Protected well:** A well that is (1) protected from runoff water through a well lining or casing that is raised above ground level and a platform that diverts spilled water away from the well and (2) covered so that bird droppings and animals cannot fall down the hole. Both conditions must be observed for a dug well to be considered as protected.  **06= Unprotected well:** A well which is (1) unprotected from runoff water; (2) unprotected from bird droppings and animals; or (3) both.  **07= Surface water:** Water located above ground and includes rivers, dams, lakes, ponds, streams, canals, and irrigation channels  **08= Other** |
| 35 | Is your piped water supply continuous or is it sometimes interrupted? | This question is for subjects who responded either “piped into dwelling”, “piped to yard/plot”, or “public tap or standpipe” to question 34. If continuous, skip to question 37. |
| 36 | How often do these interruptions last? | If the subject responded that their water is sometimes interrupted to question 35, ask how often these interruptions usually last for. If the interruptions are seasonal, enter “05” for “more than 24 hours”. |
| 37 | What is the main source of water used by your household for other purposes such as cooking and hand-washing? | This question asks about the main source of water used for purposes other than drinking. In many cases it will be the same response as that given to question 34. |
| 38 | Do you pay or barter for water? | This question asks about whether the household ever pays or barters for any source of water, either formally or informally. |
| 39 | Where is the water source located? | This question asks about the location of the household’s drinking water source. |
| 40 | How long does it take to go there, get water and come back in one trip? *If water is located on the premises, response is 000.* | If the source of drinking is located within the dwelling or yard/plot, or if the household relies on rainwater, enter “000” as the response to this question. Otherwise, include the time it takes to get to the source, wait to get water (if necessary), and get back to the house. Record the time it takes to get water by whatever means of transportation the person generally uses, whether the person walks or rides a bicycle or motor vehicle. Convert answers given in hours to minutes. Put zeroes in front of the response if necessary; for example, “30 minutes” would be ‘030,’ and “one hour and a half” would be ‘090.’ |
| 41 | Who is the main person in the household who goes to fetch water from this source? | The purpose if this question is to know which family member usually performs the task of fetching water. Knowing which member of the household usually hauls the water gives us an idea of whether gender and generational disparities exist with respect to water hauling responsibilities. If the respondent answers that there are several members from the household who perform this chore, emphasize that you are interested in the person who usually fetches the water (i.e., most of the time). |
| 42 | Do you treat your water in any way to make it safer to drink? | The purpose of questions 42 and 43 is to know whether the household drinking water is treated within the household and if so, what type of treatment is used. The type of treatment used at the household level provides an indication of the safety of the drinking water used in the household. |
| 43 | What do you usually do to the water to make it safer to drink?  *Note that the order of response categories has changed since the PSE for this question.* | The meaning of response categories for this question are:  **01= Let it stand and settle:** Holding or storing water undisturbed and without mixing long enough for larger particles to settle out or sediment by gravity  **02= Solar disinfection:** Exposing water, which is stored in buckets, containers, or vessels, to sunlight.  **03= Using a water filter (ceramic/sand/composite/etc.):** The water flows through a media to remove particles and at least some microbes from water. Media used in filtering systems usually include ceramic, sand and composite.  **04= Strain it through a cloth:** Pouring water through a cloth which acts as a filter for collecting particulates from the water  **05= Add bleach/chlorine:** Use of free chlorine to treat drinking water. Free chlorine may be in the form of liquid sodium hypochlorite, solid calcium hypochlorite, or bleaching powder  **06= Boil:** Boiling or heating of water with fuel  **07= Other** |
| 44 | Do you wash your hands after helping your child defecate? | Questions 44-46 ask about the respondent’s hand-washing practices. This question asks about whether the respondent washes her hands after helping her child defecate, which includes changing diapers. In all cases hand-washing must include use of soap. |
| 45 | Do you wash your hands before preparing food? | This question asks about whether the respondent washes her hands with soap directly before preparing food. |
| 46 | Do you wash your hands after using the toilet? | This question asks about whether the respondent washes her hands with soap after using the toilet herself. |
| 47 | Do you use toilet paper? | This question refers to whether the respondent uses toilet paper after urinating or defecating herself (as opposed to helping her child). |
| 48 | What kind of toilet facility do members of your household usually use?  *Note that the order of response categories has changed since the PSE for this question.* | The purpose of this question is to obtain a measure of the sanitation level of the household, since toilet facilities are important for disease control and health improvement. Below are some definitions for the terms used in the codes:  **01= No facility/bush/field or bucket toilet:** The household has no facility and/or uses the bush, field, or a bucket toilet near the household.  **02= Pit latrine without flush:** Excreta is deposited without flushing directly into a hole in the ground. Pit latrines without flush include:  - Ventilated improved pit latrine (VIP): A latrine ventilated by a pipe extending above the latrine roof. The open end of the vent pipe is covered with gauze mesh or fly-proof netting and the inside of the superstructure is kept dark.  - Pit latrine with slab: A latrine with a squatting slab, platform or seat firmly supported on all sides which is raised above the surrounding ground level to prevent surface water from entering the pit and for ease of cleaning.  - Pit latrine without slab/open pit: A latrine without a squatting slab, platform or seat.  An open pit is a rudimentary hole in the ground where excreta is collected.  **03= Flush** **to piped sewer system:** A system of sewer pipes (also called sewerage), that is designed to collect human excreta (feces and urine) and wastewater and remove them from the household environment. Sewerage systems consist of facilities for collection, pumping, treating and disposing of human excreta and wastewater.  **04= Flush** **to septic tank:** An excreta collection device consisting of a watertight settling tank normally located underground, away from the house or toilet.  **05= Flush** **to pit latrine**: A system that flushes excreta to a hole in the ground.  **06= Flush** **to somewhere else** A system in which the excreta is deposited in or nearby the household environment in a location other than a sewer, septic tank, or pit, e.g., excreta  may be flushed to the street, yard/plot, drainage ditch or other location.  **07= Other** |
| 49 | Do you share this toilet facility with other households? | Question 49 asks about whether the toilet facilities are shared with one or more other households. In question 50 we want to find out how many households use the same facility. This is an important measure of the level of hygiene in the household. |
| 50 | How many households use this toilet facility? | This question asks how many households regularly use the same toilet facility as the respondent household. If 10 or more households use this toilet facility enter “10”. |
| 51 | Does your household ever have electricity?  *If no, skip to question 55.* | This question asks whether the household ever has access to electricity. Even if the electricity access is irregular or unreliable, respond yes “01” to this question. |
| 52 | Is your electricity supply continuous year-round, or is it sometimes interrupted?  *If continuous, skip to question 55.* | This question asks whether the household’s electricity supply is ever interrupted. |
| 53 | *If sometimes interrupted:* How long do these interruptions usually last? | This question asks, when household electricity supply is interrupted, how long do these interruptions usually last. The respondent should give an approximate response that captures what happens most often when interruptions take place. |
| 54 | In case of discontinued power supply what source does this household usually use? | If there is no alternate electricity supply in cases of discontinued power, enter “NA”. |
| *The answers to these questions on ownership of certain items will be used to form a rough measure of the socioeconomic status of the household. Read out each item and enter the answer given for each item. Do not leave any item(s) blank. If the respondent reports that a household item such as a radio is broken, try to find out how long it has been broken and whether it will be fixed. If the item appears to be out of use only temporarily, enter ‘01’ for Yes. Otherwise, enter ‘00’ for No. If household assets were donated or given to the household, either as gifts or as part of a poverty alleviation program, they should still be included as long as they are in working form.* | | |
| 55 | Does your household have an iron (either charcoal or electric)? |  |
| 56 | Does your household have a mattress? |  |
| 57 | Does your household have a chair or bench? |  |
| 58 | Does your household have a sofa? |  |
| 59 | Does your household have a cupboard? | This includes includes cupboards with shutters and open shelves. |
| 60 | Does your household have a table? |  |
| 61 | Does your household have an electric fan? |  |
| 62 | Does your household have a radio or transistor? |  |
| 63 | Does your household have a computer? | This includes all computers that can be used for word processing and are in working form. |
| 64 | Does your household have a television? | This includes both black and white and color TVs. |
| 65 | Does your household have a mobile telephone? | This refers to whether any member of the household owns a working mobile phone. |
| 66 | Does your household have a refrigerator? |  |
| 67 | Does your household have a watch or clock? | This refers to any functioning watch or clock, regardless of quality. |
| 68 | Does your household have a bicycle? | A small child’s bicycle is primarily a toy and should not be recorded here. |
| 69 | Does any member of your household own a bank account? | This refers to formal bank accounts only, and does not include microfinance structures that operate outside of the formal banking system. |
| 70 | Does this household own any agricultural land?  *If no, skip to question 72.* | Ownership of agriculture land is another important indicator of the socioeconomic status of the household. First ask question 69 to find out whether any member of the household owns any land that can be used for agriculture. If the answer is YES, ask question 70 on the number of acres owned altogether by the members of the household. Record the answer in the respective boxes. |
| 71 | How much agricultural land does this household own? |  |
| 72 | Does your household own cows, bulls, or buffaloes? | For questions 72-73, answer “yes” if the household owns any of the animals listed, regardless of the number. |
| 73 | Does your household own chickens or ducks? |  |
| 74 | Do you ever heat your house?  *If no, skip to question 76.* |  |
| 75 | What is the primary source of fuel used for heating in your household? | If the household uses more than one fuel for heating, find out the fuel used most often and enter the code for that fuel. |
| 76 | What type of cooking stove is mainly used in your house? | In these questions we want to find out whether the food in the household is cooked on a stove or an open fire and the approach used if any to channel the smoke, i.e. a chimney or a hood or both. You may need to probe for an exact description of the type of stove. |
| 77 | Is cooking done inside the house, outside the house, or both? | The purpose of this question is to collect information on the location where food is prepared in the household: in the household, outside the household, or both. This information is important in providing an indicator of the air quality inside and around the dwelling. |
| 78 | *Main material of the floor (observation)* | This is an observation not a question since you will usually be able to see for yourself what kind of floor the house has. However, ask if you are not sure. If there is more than one kind of flooring material, record the main type of material (the material that covers the largest amount of floor space). |
| 79 | *Main material of the roof (observation)* | As with the floor material, you will usually be able to see for yourself what kind of roof material the house has. However, observing the roof material may not always be easy or you may be able to observe part but not the whole roof. Ask the respondent if you are not sure or if you can not observe the roof properly. If the household lives in an apartment building, look at the roof from a reasonable distance and ask the respondent if necessary. If there is more than one kind of roofing material, record the main type of material (the material that covers the largest amount of roof space). |
| 80 | *Main material of the exterior walls (observation)* | This is not a question but an observation. You will usually be able to see for yourself what kind of material the exterior walls are made of. However, ask the respondent if you are not sure. Again, if there is more than one kind of wall material, record the main type of material (the material that covers the largest amount of wall space). |
| 81 | Do you have a separate room which is used as a kitchen? | This question seeks information on whether the household has a separate room used as a kitchen in order to provide additional information on the hygiene status of the household. |
| 82 | Which of the following is the primary way you distinguish members of your population? | This question asks about the *primary* method for distinguishing between members of the population. While more than one of these categories might apply, emphasize that you would like to know which of the categories is used most often. For questions 83-87, only ask the question that corresponds to the response on question 82. For example, if the respondent states that *language spoken (04)* is the primary way of distinguishing members of her/his population, then only ask question 86. Do not ask questions 83, 84, 85, or 87. |
| 83 | *If caste (01) is the response for question 82:* What caste are you a member of? | Only ask this question if the response to question 82 was *caste (01).* |
| 84 | *If race (02) is the response for question 82:* Which racial group are you a member of? | Only ask this question if the response to question 82 was *race (02).* |
| 85 | *If tribe (03) is the response for question 82:* Which tribal group are you a member of? | Only ask this question if the response to question 82 was *tribe (03).* |
| 86 | *If language spoken (04) is the response for question 82:* What language do you speak in your household? | Only ask this question if the response to question 82 was *language spoken (04).* |
| 87 | *If religion (05) is the response for question 82:* What religion do you practice in your household? | Only ask this question if the response to question 82 was *religion (05).* |
| 88 | What is the average monthly income for the entire household? | This question asks about the average monthly income in the household. In most cases this amount will be an estimate as most households may not receive a regular income. Encourage the respondent to include monetary income from all household members. Enter the amount in the top row given for responses, and the letter associated with the local currency in the box next to *(currency)* on the bottom row. |

**Household Surveillance**

SAF—Surveillance Assessment Form

I. Purpose

To collect twice weekly information on child illness and intake of specific foods throughout two to three years of participation.

II. Material

Surveillance Assessment Form (SAF), Child Referral Form (REF), thermometer, timer (or watch with second hand), materials for diarrhea stool specimen collection, pen, clipboard.

III. Methods

1. A Study Researcher / Nurse / Fieldworker trained in measuring respiratory rate / temperature will administer this form:
   1. After screening has occurred and consent has been obtained
   2. Within 17 days of birth
2. The form is set up to collect data on a monthly basis – At the beginning of the month (or beginning of surveillance), write the child’s Participant ID and the month/year at the top of the form.
3. The Study Researcher / Nurse / Fieldworker will begin collecting surveillance information on the same day as the child is enrolled in the study. If that is not possible, the surveillance form can be completed on the day after enrollment. On the first day of surveillance, the Study Researcher / Nurse / Fieldworker will ask the mother if the child has been sick at all since birth (giving examples of diarrhea, cough, fever, etc.) and if illness is reported, ask all of the questions under that day. Please enter NA for the days prior to the birth of the child.
4. The Study Researcher / Nurse / Fieldworker will visit the houses of enrolled children twice a week in order to collect data on illness and diet using the SAF. Study Researcher / Nurse / Fieldworkers will visit houses on regularly scheduled days (e.g., Tuesdays and Fridays, Mondays and Thursdays) and ask about illness and food intake on the days since the last visit.
5. The Study Researcher / Nurse / Fieldworker will generally ask about symptoms on the 3 or 4 days since the last visit; e.g., if today is May 8^th^ and the last visit was on May 5^th^, the Study Researcher / Nurse / Fieldworker will fill in information for May 5^th^-7^th^ (columns 5, 6, 7). Information on today (May 8^th^) will be collected during the next visit. Days begin at 08:00 am and end at 07:59 am the following morning. For food intake, refer only to intake on the day before the visit (ending that morning).
6. For those questions that require measurement/collection on the day of visit, enter the data in the column for the day of visit. For example, in the course of asking about yesterday (May 7^th^), if a diarrhea episode is identified, then for question 17, diarrhea sample collected, the study researcher / nurse / fieldworker will collect a stool sample and fill in Yes in the column corresponding to the day the sample was collected (May 8^th^). Another example relates to the ALRI information. If the mother reports cough or shortness of breath yesterday (May 7^th^), the study researcher / nurse / fieldworker will collect respiratory rate information today – these respiratory rates will be recorded under today’s date (May 8^th^). For the diet questions, you should enter the information in the column corresponding to yesterday’s date, since the data collected is yesterday’s dietary intake.
7. The form should be started at the beginning of the month (or at the beginning of surveillance). When the form is complete (at the end of the month), it should be given to the Data Management team for entry into the database by the 7^th^ of the following month. There will be some months when the Study Researcher / Nurse / Fieldworker needs to carry two months’ forms to the field (e.g., if the date of the visit is May 2^nd^, the Study Researcher / Nurse / Fieldworker should have the April and May forms to collect data from April 29^th^, 30^th^, and May 1^st^).
8. It is very important that the Study Researcher / Nurse / Fieldworker contacts the mother/caregiver and fills out the forms on the assigned days; however, in some cases the mother/caregiver will not be available. If the mother/caregiver is not available when the Study Researcher / Nurse / Fieldworker visits the home, the Study Researcher / Nurse / Fieldworker will return to the home at least three times on that day, and then at least once a day on all following days until the Study Researcher / Nurse / Fieldworker contacts the mother/caregiver and fills out the form. The Study Researcher / Nurse / Fieldworker will fill out the form for all days since the last contact, up to 7 days. If the last contact was more than 7 days ago, the days since the last contact up to 7 days ago will be missing data. If the Study Researcher / Nurse / Fieldworker cannot make contact with the mother/caregiver for 60 consecutive days within the first two years of follow up, the child will be dropped from the study. Within the third year (months 25-36), children should be retained in the study whenever possible, even if gaps in follow up occur.If more than two weeks of SAF data are missing, fill out a single PDF for each gap..
9. Phone collection of data – In some cases, study researchers may be able to contact the family using the phone if they have left the area for a short time. While this is okay for data collection in the short-term, this should not be done over a long period (>4 weeks). If the family is gone from the study area for more than 60 days in the first two years of the study, they will be dropped from the study.
10. On some days, the child may be ill on the day of the field worker visit. In order to standardize the timing of the data collection, we are **not** generally asking about today’s illnesses, only about illnesses on previous days. Information about diarrheal illness **today** will be collected on the next visit. There are some data that will be collected today: if the child had cough or shortness of breath yesterday, the Study Researcher / Nurse / Fieldworker will look for indrawing and measure respiratory rate, and those data will be collected under today’s date. In the same way, if the caregiver reports fever in the past 24 hours, the temperature will be taken today and documented under today’s date. The data collector is very welcome to make a note of the symptoms not documented today (e.g. diarrhea) on another piece of paper so that they are sure to not miss collection of those data on the next visit.)
11. If the child is found to have a serious or life-threatening condition (e.g., seizure, high fever, dyspnea, more than 8 episodes daily of liquid diarrhea, coma, paralysis, hemorrhage), the child will be referred to the local emergency hospital.
12. If the child is found to have a less severe condition (ALRI, dehydration, fever, blood in stool), the child will be referred to the nearest health center for assessment and treatment.
13. All referrals will be noted on the Child Referral Form (REF).

IV. General QC instructions

- 10% of study participants will receive a QC visit by the supervisor each month at which SAF data will be collected on the XAF form for comparison with the Study Researcher / Nurse / Fieldworker. Detailed instructions can be found in the XAF SOP.

- 100% of each Study Researcher / Nurse / Fieldworker’s forms should be reviewed by the supervisor on at least a weekly basis, ideally, at the end of each working day. Supervisors should ensure the forms are complete (no missing fields), that the data appear to be correct, and that visits planned for the day were made.

- Corrections to the forms should be minimized (data should be correct the first time), but if needed, the Study Researcher / Nurse / Fieldworker should follow proper correction procedures. If form corrections are necessary:

- - 1. Cross through the incorrect information once,
    2. Write the correct information,
    3. Write the date the correction was made, and
    4. Write their initials.

- Make sure forms are kept confidential and protected (in a locked file drawer, for example) when they are returned from the field.

- Data center transmission of forms

- - Once forms have been completely filled out by the Study Researchers / Nurses / Fieldworkers and reviewed by the supervisor, they must be delivered within 48 hours to the local data center for data entry. Data entry of the forms should occur within one month of delivery to the data center (preferably sooner).

- Standard rounding techniques are recommended: Round down if last digit is <5 and round up if the last digit is >5).

V. Question Guidance

| # | Question | Guidance | |
| --- | --- | --- | --- |
| At the beginning of the month (or start of surveillance), write the month and year in the upper right hand corner in spaces provided. Always ask if the child has been taken to see a health care provider since the last visit, and if so, get out a REF to fill in with the information about the visit. | | | |
| 01 | Date | The days in the Date row represent days of the month. | |
| 02 | Successful visit today? | Enter ‘0’ if you did not successfully collect any information or if you did not make a visit to the household. Enter ‘1’ under today’s day of the month (e.g., if today is May 15^th^, enter 1 in the box below 15) if you successfully make contact with the mother / caregiver to ask questions about the child’s morbidity, as this field is an indicator of when data are collected. If the mother / caregiver is not available, enter ‘0’, fill in the number of times you visit the household for question number 3 and return the following day. | |
| 03 | # times visits were made to the household | If you make contact with the mother / caregiver on the first visit and successfully complete the survey, enter ‘1’. If the mother / caregiver is not available, enter the number of visits made to the house each day. Additional visits should be made until the data are collected. | |
| 04 | Field Researcher ID | Enter the field researcher’s unique ID number here. | |
| For the following questions, answers should be recorded for all days since last visit. If this visit is at the beginning of surveillance, ask about any illnesses since birth (give examples of diarrhea, cough, fever, etc.) and if the mother reports any illness, fill in the column under the date the illness was experienced. If the last contact was more than 7 days ago, the days since the last contact up to 7 days ago will be missing data. For all questions up through question 19, you will never record illesses today after 0800 – you are only asking about illnesses in the past, starting with yesterday, 0800 through 0759 this morning. | | | |
| 05 | Illness | Was <CHILD> sick yesterday? If so, write 1 in the appropriate column. If the caregiver responds that the child was not sick, probe and ask if the child had any diarrhea, cough, fever yesterday. If the answer is still no, write 0. Continue with all of the following questions and then ask about the other days since the last visit. When finished with all days since the last visit, continue to second page. | |
| 06 | Activity level | What was <CHILD>’s activity level? Possible answers: 0=normal, 1=sleepy, and 2=difficult to awaken. | |
| 07 | Oral intake / Appetite | How was <CHILD>’s appetite? Possible answers: 0=normal or more than normal, 1=less than normal. | |
| 08 | Vomiting | Did <CHILD> vomit? Possible answers: 1=yes or 0=no. | |
| 09 | Ear pain / pulling | Did <CHILD> have ear pain / pulling? Possible answers: 1=yes or 0=no. | |
| 10 | Antibiotic use | Did <CHILD> take oral or injected antibiotics? Possible answers: 1=yes or 0=no. If yes, ask to see package, other documentation of antibiotics / medication for question 11. If no, write ‘NA’ for question 11 and go on to question 12. If antibiotics were used on the eye or skin, answer 0 to this question and document the antibiotics used on the NUR form. | |
| 11 | Antibiotic type | What kind of antibiotics did <CHILD> take? Refer to package or rely on maternal report – antibiotic options on form. If there is no package and the mother does not know what kind of antibiotics were given, ask if the caretaker has any paperwork from the healthcare provider that might list the recommended medications, or if there are pills left over that the Study Researcher / Nurse / Fieldworker can look at. If there is no documentation and the Study Researcher / Nurse / Fieldworker cannot identify the antibiotic by looking at the pills, enter 7 (unknown) in this box. All available information (color of the pills, field researcher’s best guess of what medication names are written) should be entered into the Nursing Notes Form for discussion with the supervisor. If the name of the antibiotic is known, but the data collector does not know what class of antibiotic it is, they should write down the name on the Nursing Notes Form and ask the supervisor. Ideally, the supervisor will generate a list of the most common antibiotics in the study site and the corresponding class of antibiotics for entry into the form.  If a child is using antibiotics from 2 different classes (e.g. penicillin and sulfonamides), please record one antibiotic on one day and the other antibiotic on the next day. In the NUR (Nursing Notes form) you should record that both antibiotics were being given to the child on the same day of visit. | |
| 12 | Diarrhea | Did <CHILD> have diarrhea? Possible answers: 1=yes or 0=no. If a respondent is not sure what we mean by diarrhea, tell her it means “loose or runny stools That take the shape of their container.” This question is supposed to assess whether or not the mother thinks the child had diarrhea (ie, it does not matter if the diarrhea met the study definition – it is the mother’s perception of illness in the form of diarrhea). Continue to ask the following questions, regardless of whether the mother reports that the child had loose stools or not. | |
| 13 | # loose stools | How many loose stools did <CHILD> pass? (Loose stools take the shape of their container). If the child passed 8 or more loose stools in a 24 hour period, refer to the nearest health center for assessment / treatment (and fill out the Child Referral Form (REF)). This question is to assess whether the child had any loose stools and if the child met the definition for diarrhea yesterday and may therefore require a sample collection (if no sample has been collected for this particular episode, see Q17). | |
| 14 | Blood in stool | Was there any blood in <CHILD>’s stools? Possible answers: 1=yes or 0=no. If the answer to this question is yes, refer to the nearest health center for assessment / treatment (and fill out the Child Referral Form (REF)). | |
| 15 | Dehydrated | Was <CHILD> dehydrated? If the mother answers no, enter 0 (none). If the mother answers yes, ask whether the dehydration was mild to moderate (some) (1) or severe (2). Dehydration is defined as:  None: non-sunken eyes, no lethargy, if irritable, easily consoled, drinks or breast feeds well, normal abdominal skin pinch  Some: irritable/difficult to console, delay in skin pinch return, eyes sunken, when offered liquid drinks eagerly/demonstrates greater than normal thirst  Severe: lethargic child, sunken eyes, listless, difficulty in mother/child interaction  If the mother reports severe dehydration, refer to the nearest health center for assessment / treatment (and fill out the Child Referral Form (REF)). | |
| 16 | ORT administered | Was <CHILD> given oral rehydration salts (ORS) such as [LOCAL NAME FOR ORS PACKET], a pre-packaged ORS liquid, or a government-recommended homemade fluid? Possible answers: 1=yes or 0=no. | |
| 17 | Sample collected | Possible answers: 1=yes, 0=no, NA.  Enter this information in the column for the day of the visit, for example, if diarrhea is reported yesterday, one would collect the stool sample during today’s visit and note the sample collection under the column for today’s date. See Diarrhea Specimen Collection section of the Stool Collection SOP for detailed sample collection instructions.  Diarrhea sample should be collected if :   1. the child had diarrhea yesterday (>3 loose stools) AND 2. if no diarrhea sample has been collected for this episode of diarrhea within the past two weeks.   Only one diarrhea sample should be collected for each diarrhea episode or, in the case of persistent diarrhea, samples should be taken every two weeks during the extended episode. Diarrhea episodes are separated by at least two diarrhea-free days. For example, if the child had diarrhea yesterday, but no diarrhea on the two previous days, you would collect a diarrhea sample. On the next visit in 3 or 4 days, if the child continued to have diarrhea and the episode did not end (the child did not have two or more consecutive diarrhea-free days), you would not take another diarrhea sample. If, however, the child experienced two or more diarrhea-free days and then started a new diarrhea episode, you would collect another sample.  When the sample is collected, enter Yes in the column corresponding to the day of collection. The answer to this question should be ‘NA’ if no stool sample collection is indicated (because of no diarrhea or because a sample has already been collected during this episode) and No if the sample should have been collected but collection was not successful (and additional attempts should be made to collect this sample). If a child goes two days without diarrhea and no sample was collected for that episode, the Study Researcher / Nurse / Fieldworker has missed collection for that episode and a Protocol Deviation Form should be filled out. | |
| 18 | Cough | Did <CHILD> have an illness with a cough? Possible answers: 1=yes or 0=no. | |
| 19 | Short of breath | Was <CHILD> short of breath? Short, rapid breathing or difficulty breathing are signs of pneumonia or other acute respiratory infection, which are a principal cause of death among children. Possible answers: 1=yes or 0=no. | |
| If the answer to Question 18 or 19 was YES yesterday, continue to Question 20. Questions 20-23 and 25-27 should be recorded in the column for the day of the visit (since you are measuring respiratory rate, taking temperature, and making referrals today). If the caregiver does not report coughing or shortness of breath in the child yesterday, fill in ‘NA’ for questions 20-23 and go to Question 24. | | | |
| 20 | Indrawing | Observe the child’s chest for indrawing. Possible answers: 1=yes or 0=no. If the answer to this question is YES, refer the child to the nearest health center for assessment / treatment (and fill out the Child Referral Form (REF)). | |
| 21 | Respiratory rate 1 | Take the child’s respiratory rate (breaths per minute) twice. Enter the first respiratory rate here. | |
| 22 | Respiratory rate 2 | Enter the second respiratory rate here. | |
| 23 | Acute lower respiratory infection (ALRI) | Possible answers: 1=yes or 0=no. If the child has cough or shortness of breath PLUS rapid respiratory rate (defined as: >60 breaths per minute in children aged 0 to <2 months, >50 breaths per minute in children aged 2 to <12 months and >40 breaths per minute in children aged 12 months to three years), then answer yes (1) to this question and refer the child to the nearest health center for assessment / treatment (and fill out the Child Referral Form (REF)). | |
| 24 | Fever | Has <CHILD> been ill with a fever? Possible answers: 1=yes or 0=no. If the answer is ‘no’, enter ‘NA’ in question 25 and go on to question 26. If the answer is ‘yes’, take the child’s temperature and record in question 25. | |
| 25 | Temperature | If the mother reports a fever yesterday, take the child’s temperature and record here XX.X^o^C. If the temperature is above 39^o^C, refer the child to the nearest health center for assessment / treatment (and fill out the Child Referral Form (REF)). | |
| 26 | Referral made | If the child was referred to a health center for any reason, enter 1 in this space in the column that corresponds to today’s date and fill out the Child Referral Form (REF). If the child was not referred for any reason, enter ‘0’. | |
| 27 | Nursing notes | The Nursing Notes Form (NUR) can be used to collect any other information about the child’s health status. This form is not required by the study, but can be used at the discretion of the local site. If the NUR form was used, enter 1 here. If no nursing notes were written, enter ‘0’. | |
| The following questions should be asked about the diet yesterday (from yesterday sunrise until this morning sunrise). This first section asks about all liquids the child has consumed during the past 24 hours. Fill in the column corresponding to yesterday’s date since you are asking about yesterday’s dietary intake. | | | |
| 28 | Breast milk | Are you breastfeeding <CHILD>? Possible answers: 1=yes or 0=no. If NO, enter ‘NA’ for questions 29 and 30, and then skip to Q.31 | |
| 29 | Breast milk during night | Last night, how many times did you breastfeed <CHILD> from sunset to sunrise? Enter the number here. If answer is 'as often as the baby wanted' or 'I don't know' then probe with 'how often did the baby want to nurse, 2 times or 5 times or 10 times?' | |
| 30 | Breast milk during day | Yesterday, during the day, how many times did you breastfeed <CHILD>? Enter the number here. If answer is 'as often as the baby wanted' or 'I don't know' then probe with 'how often did the baby want to nurse, 2 times or 5 times or 10 times?' | |
| 31 | Infant formula | Do you give <CHILD> infant formula? Possible answers: 1=yes or 0=no. If NO, enter ‘NA’ for questions 32 and 33 and then skip to Q.34 | |
| 32 | Infant formula during night | Last night, how many times did you feed <CHILD> formula from sunset to sunrise? Enter the number here. If answer is 'as often as the baby wanted' or 'I don't know' then probe with 'how often did the baby want to eat, 2 times or 5 times or 10 times?' | |
| 33 | Infant formula during day | Yesterday, during the day, how many times did you feed <CHILD> formula? Enter the number here. If answer is 'as often as the baby wanted' or 'I don't know' then probe with 'how often did the baby want to eat, 2 times or 5 times or 10 times?' | |
| 34 | Animal milk | Do you give <CHILD> other milks, such as canned, powdered or fresh animal milk? Possible answers: 1=yes or 0=no. If NO, enter ‘NA’ for questions 35 and 36, and then skip to Q.37 | |
| 35 | Animal milk during night | Last night, how many times did you feed <CHILD> animal milks from sunset to sunrise? Enter the number here. If answer is 'as often as the baby wanted' or 'I don't know' then probe with 'how often did the baby want to eat, 2 times or 5 times or 10 times?' | |
| 36 | Animal milk during day | Yesterday, during the day, how many times did you feed <CHILD> animal milk? Enter the number here. If answer is 'as often as the baby wanted' or 'I don't know' then probe with 'how often did the baby want to eat, 2 times or 5 times or 10 times?' | |
| 37 | Plain water | Yesterday, during the day or last night, did <CHILD> have plain water? Possible answers: 1=yes or 0=no. | |
| 38 | Tea, coffee, local examples | Yesterday, during the day or last night, did <CHILD> have tea or coffee or <local examples>? Possible answers: 1=yes or 0=no. | |
| 39 | Fruit juices | Yesterday, during the day or last night, did <CHILD> have any fruit juices? Possible answers: 1=yes or 0=no. | |
| 40 | Other liquids | Yesterday, during the day or last night, did <CHILD> have any other liquids, such as sugar water, thin soup or broth, carbonated drinks, <local examples>? Possible answers: 1=yes or 0=no. | |
| The following questions are about solid foods. This is not meant to be a comprehensive look at the child’s diet; rather, these questions ask about specific foods that are thought to have beneficial effects on the child’s digestive system. | | | |
| 41 | Semi-solid foods | Is <CHILD> eating any semi-solid, mashed or solid foods? Possible answers: 1=yes or 0=no. If NO, stop and draw a line through questions 42 through 50. If yes, continue to Q.42. | |
| Thinking about yesterday, during the day and at night, did <CHILD> have any of the following foods (even if they were in combination with other foods)? Fill in the column corresponding to yesterday’s date since you are asking about yesterday’s dietary intake. | | | |
| 42 | Porridges, breads, other foods/drinks made from wheat, oat, barley, soy? | | Possible answers: 1=yes or 0=no. |
| 43 | Rice, millet? | | Possible answers: 1=yes or 0=no. |
| 44 | White potatoes, white yams, manioc, other roots? | | Possible answers: 1=yes or 0=no. |
| 45 | Peanuts, spreads with peanuts? | | Possible answers: 1=yes or 0=no. |
| 46 | Beans, peas, lentils? | | Possible answers: 1=yes or 0=no. |
| 47 | Bananas? | | Possible answers: 1=yes or 0=no. |
| 48 | Yogurt, keifer? | | Possible answers: 1=yes or 0=no. |
| 49 | Onion, leeks, shallots, garlic? | | Possible answers: 1=yes or 0=no. |
| 50 | Fermented foods or drinks? | | Possible answers: 1=yes or 0=no. |
| 51 | Supervisor check | | The supervisor should review every surveillance form after every completed visit and write his or her initials in this space to demonstrate that he or she has reviewed that day’s data collection and found it to be complete. The supervisor only needs to initial on the days that the review is performed (does not need to initial all days). |

XAF—Surveillance Check Form

I. Purpose

To allow supervisors to ensure that Study Researcher / Nurse / Fieldworkers are making their scheduled visits and obtaining accurate data.

II. Summary

The supervisor goes to the household and collects Surveillance Assessment Form data (XAF – the supervisor’s version of the SAF) for comparison with regular Study Researcher / Nurse / Fieldworker’s data collection.

III. Number

10% of participants – The supervisor must visit 10% of the participants one time each month. At the beginning of each month, the supervisor will calculate the total number of participants enrolled in the study and make plans to visit at least 10% of those households one time to administer the XAF. In order to standardize the process, the supervisor will ask about illnesses on the last three days.

IV. Process options

One suggested process is that the supervisor (or their designee) chooses a random day of the month to visit all households (or a random sample) that are scheduled to receive a Study Researcher / Nurse / Fieldworker visit on that day. The supervisor should not tell the Study Researcher / Nurse / Fieldworker which day the check will happen, nor should they be at the house at the exact same time. The supervisor may need to choose additional days for data collection checks if the total visits possible on one day do not total at least 10% of the participants currently enrolled in the study. Some sites may want to make SAF QC visits evenly over the month and that is fine as well. The 10% sample should be random and representative of the participants’ ages, locations, and all other variables.

V. Additional Notes

If the supervisor would like to check a higher percent of the total participants, particularly in the beginning or when a new Study Researcher / Nurse / Fieldworker is hired, they are encouraged to do so.

The supervisor should review the XAF and SAF forms daily (or at least weekly, if daily is untenable) and discuss discrepancies found with the Study Researcher / Nurse / Fieldworker and provide additional training if necessary. Do not change the data that the Study Researcher / Nurse / Fieldworker collected!

VI. Inconsistencies

The original data collected by the Study Researcher / Nurse / Fieldworker will be entered into the database. There is no need to ‘correct’ the data based on the supervisor’s responses. This comparison is done simply to guarantee that the Study Researcher / Nurse / Fieldworker is administering the form correctly and consistently in the field.

1. Minor inconsistencies

The supervisor should discuss the different responses with the Study Researcher / Nurse / Fieldworker. In many cases, the different answers may be due entirely to the respondent’s mood or ability to recall information at that moment. For instance, the first time the mother answers the question about liquid stools, she might answer 2, but the second time, she might have thought more about it and remembered another liquid stool on that day, so she answered 3 the second time. This is normal and does not need to cause major concern.

1. Major inconsistencies:

If, for example, the Study Researcher / Nurse / Fieldworker records no illness on a certain day but the supervisor records that the mother reported diarrhea, antibiotics, fever and a cough, the supervisor should attempt to verify that the Study Researcher / Nurse / Fieldworker is making their visits as scheduled and that they are asking the questions in a way that encourages complete answers. Major inconsistencies can be defined as more than 15% of the total answers that are discrepant. If a Study Researcher / Nurse / Fieldworker consistently returns data that poorly correlates with the supervisor’s data, and does not improve after additional training, that Study Researcher / Nurse / Fieldworker should be replaced.

VII. Example

In month 6, as of July 1st, there are 72 children enrolled in the study. In July, the supervisor will visit seven of the households and collect XAF data.

The supervisor (or designee) can initially choose one day and one Study Researcher / Nurse / Fieldworker, and will visit every household on that Study Researcher / Nurse / Fieldworker’s schedule for the day.

The supervisor only needs to ask the questions on the form and does not need to actually collect diarrheal stool samples; however if a stool sample was indicated due to presence of diarrhea, the supervisor should answer question 17 on the XAF yes (01) and ensure that the Study Researcher / Nurse / Fieldworker actually collected a sample.

If the supervisor only visited 4 households on the initial QC visiting day, he or she can choose another day during the month to visit additional households until he or she has visited at least 10%. In this case, 10% is 7.2 – it is allowable to round down to 7 households. If the number were 7.6, 8 households would be required.

If the supervisor completed a visit, but the Study Researcher / Nurse / Fieldworker was unable to complete the visit on that day, the supervisor’s data should be discarded and not used for quality control. Visits must occur on the same day for a valid comparison.

In this example, if the supervisor chooses to schedule visits throughout the month, they would visit approximately two households each week to complete the XAF.

NUR—Nursing Notes Form

I. Purpose

To allow the Study Researcher / Nurse / Fieldworker to document additional information about the child during twice weekly surveillance throughout two years of participation.

II. Material

Nursing Notes Form (NUR), pen, clipboard

III. Methods

A Study Researcher / Nurse / Fieldworker will utilize this form as dictated by the particular site. If nursing notes are written on a particular day, this should be noted on the Surveillance Assessment Form (SAF), Q27. Nursing notes should not be entered into the central database, but can be entered into the local database. The NUR form should be returned to the MAL-ED study center when the page is full or when the Surveillance Assessment Form (SAF) is returned, whichever comes first.

IV. General QC instructions

- 100% of each Study Researcher / Nurse / Fieldworker’s forms should be reviewed by the supervisor on at least a weekly basis, ideally, at the end of each working day. Supervisors should ensure the forms are complete (no missing fields), that the data appear to be correct, and that visits planned for the day were made.

- Corrections to the forms should be minimized (data should be correct the first time), but if needed, the Study Researcher / Nurse / Fieldworker should follow proper correction procedures. If form corrections are necessary:

- - 1. Cross through the incorrect information once,
    2. Write the correct information,
    3. Write the date the correction was made, and
    4. Write their initials.

- Make sure forms are kept confidential and protected (in a locked file drawer, for example) when they are returned from the field.

- Data center transmission of forms

- - Once forms have been completely filled out by the Study Researchers / Nurses / Fieldworkers and reviewed by the supervisor, they must be delivered within 48 hours to the local data center for data entry. Data entry of the forms should occur within one month of delivery to the data center (preferably sooner).

| # | Question | Guidance |
| --- | --- | --- |
|  | Participant ID | Write the participant ID in the space provided at the upper left corner. |
| 01 | Study Researcher / Nurse / Fieldworker ID | Enter the Study Researcher/Nurse/Fieldworker’s unique ID number here. |
| 02 | Today’s date | Format DD/MMM/YY |
| 03 | Nursing notes | This space can be used for any notes the fieldworker would like to make about the child’s health, information about the family (e.g., future travel plans), or any other information that the fieldworker would like to document (such as other medications used, injections given i.e. malaria vaccine or local folk medication or healing practices i.e. gripe water). This form can also be used to document adverse events. If adverse events occur, notify the site PI within 24 hours of the event. |

REF—Child Referral Form SOP

I. Purpose

To document child illness referrals and outcome of those referrals.

II. Material

Child Referral Form (REF), pen, clipboard

III. Methods

1. This form is filled out as a follow up to referrals made by the Study Researcher / Nurse / Fieldworker, to document self-referral to medical care, and to document medication taken without contact with the medical system. It is important for the study to document medical care and treatment received by the child in order to have a good picture of the health of the child.
2. If the child is hospitalized or the Study Researcher / Nurse / Fieldworker is unable to get information about a diagnosis, the Study Researcher / Nurse / Fieldworker should wait until the child is discharged or until the information is available to fill out questions 9-14.
3. If the child becomes seriously ill or is hospitalized, the Principal Investigator must be notified.
4. When filling out this form, the Study Researcher / Nurse / Fieldworker should request to see the documentation from the healthcare provider. This can help the Study Researcher / Nurse / Fieldworker identify the diagnosis.

IV. General QC instructions

- 100% of each Study Researcher / Nurse / Fieldworker’s forms should be reviewed by the supervisor on at least a weekly basis, ideally, at the end of each working day. Supervisors should ensure the forms are complete (no missing fields), that the data appear to be correct, and that visits planned for the day were made.

- Corrections to the forms should be minimized (data should be correct the first time), but if needed, the Study Researcher / Nurse / Fieldworker should follow proper correction procedures. If form corrections are necessary:

- - 1. Cross through the incorrect information once,
    2. Write the correct information,
    3. Write the date the correction was made, and
    4. Write their initials.

- Make sure forms are kept confidential and protected (in a locked file drawer, for example) when they are returned from the field.

- Data center transmission of forms

- - Once forms have been completely filled out by the Study Researchers / Nurses / Fieldworkers and reviewed by the supervisor, they must be delivered within 48 hours to the local data center for data entry. Data entry of the forms should occur within one month of delivery to the data center (preferably sooner).

| # | Question | Guidance |
| --- | --- | --- |
|  | Child ID | Write the child ID in the space provided at the upper left corner. |
| 01 | Study Researcher / Nurse / Fieldworker ID | Enter the Study Researcher / Nurse / Fieldworker’s unique ID number here. |
| 02 | Referral source | Enter here whether the child was referred for medical care by a Study Researcher/Nurse/Fieldworker (00) or if they sought care on their own (01). If no medical care was sought, but nutritional supplementation or medical treatment was given to the child, enter NA. |
| If the Study Researcher / Nurse / Fieldworker referred the child for medical care, fill in questions 3 and 5. | | |
| 03 | Referral date | Enter the date the child was referred for care. Format DD/MMM/YY |
| 04 | Today’s date | Enter today’s date. Format DD/MMM/YY |
| 05 | Visit healthcare provider | If the caregiver followed through with the referral and visited a health center based on the Study Researcher / Nurse / Fieldworker’s referral, enter 01 (Yes) and continue with the questions. If the caregiver did not seek out medical care, enter 00 (No). If the caregiver did not seek out medical care and the medical problem continues, the fieldworker should refer the child again for medical care (and fill out another Referral Form (REF)). |
| The following questions should be filled in for all reported interactions with health care providers (both referred by study staff and self-referred). Also, if the caregiver reports giving medication or nutritional supplementation to the child without consulting with the health care system, document this in questions 16-19. | | |
| 06 | Reason for seeking health care | Enter here the primary reason for seeking healthcare – if the primary reason for referral was diarrhea related illness, enter 01. If the primary reason for referral was ALRI related illness, enter 02. If the primary reason for referral was malaria, enter 03. If the primary reason for referral was poor growth, enter 05. If the primary reason for referral was anemia, enter 06. If the primary reason for referral was another reason (not 1, 2, 3, 5, or 6), enter 04 and write the reason in Q7.  If there is more than one reason for seeking care, enter the primary option in question 6 and write the other (secondary) option(s) in question 7 (other). |
| 07 | Other | If the reason for referral was not listed above (or if there was more than one reason), write the (secondary) reason here. |
| 08 | Date treatment was sought | Enter the date treatment was first sought. Format DD/MMM/YY |
| 09 | Source of health care | Enter here the source of healthcare – If the caregiver sought health care at a pharmacy, enter 01. If the caregiver sought health care at a formal health center or hospital, enter 02. If the caregiver sought care at another location, enter 03. |
| 10 | Diagnosis - dehydrating diarrhea | Enter here if the child was diagnosed with dehydrating diarrhea by a medical professional (doctor or nurse). Possible answers yes=01, no=00. |
| 11 | Diagnosis - ALRI | Enter here if the child was diagnosed with ALRI by a medical professional (doctor or nurse). Possible answers yes=01, no=00. |
| 12 | Diagnosis - Malaria | Enter here if the child was diagnosed with malaria by a medical professional (doctor or nurse). Possible answers yes=01, no=00. |
| 13 | Diagnosis – Other | Write the diagnosis here if the child was diagnosed with another condition by a medical professional (doctor or nurse). |
| 14 | Hospitalized? | Enter here whether the child was hospitalized as a result of this health center visit. Possible answers yes=01, no=00. |
| 15 | Hospitalized - # days | If the child was hospitalized, enter the number of days hospitalized here. |
| 16 | Nutritional supplementation | Enter 01 here if nutritional supplementation (e.g., pushti packet, vitamins, plumpynut, etc.) was given and write in supplement type in question 17. Enter 00 if no supplementation was given and go to question 18. |
| 17 | Supplement description | Write in type of nutritional supplementation that was given to the child. |
| 18 | Medication | Enter 01 here if medication (western or traditional) was given and write in medication type in question 19. Enter 00 if no medication was given. |
| 19 | Medication name | Write in type of medication that was given to the child. |

MOA—Monthly Form A

I. Purpose

To document dietary diversity, vaccine history, and anthropometry on a monthly basis up to and including 8 months of age.

II. Material

Monthly Form A, pen, clipboard, equipment to measure weight, height and head circumference (see Anthropometry SOP), materials for monthly stool specimen collection (see Monthly Stool Specimen Collection SOP)

III. Methods

The Study Researcher / Nurse / Fieldworker trained in measuring anthropometry and dietary assessment will administer this form monthly when the child is one to eight months of age. Ideally, the fieldworker will visit the home on the anniversary of the child’s date of birth (for a child born on May 17^th^, the fieldworker would visit on June 17^th^, July 17^th^, etc.) but it may not always be possible to visit on the exact day. At times when the exact anniversary date visit is not possible, the form can be filled out within two days before or after (for a child born on May 17^th^, the fieldworker can go between June 15^th^ and June 19^th^ for the one month visit, between July 15^th^ and July 19^th^ for the two month visit, etc.). It is very important that the form be completed within the given +/- two day window; however, if the visit is missed within that window, the Study Researcher / Nurse / Fieldworker may visit the household to collect the MOA / MOB information up to 7 days beyond the birthdate anniversary. If data collection occurs outside the +/- 2 day window, a PDF form must be completed. If the visit is performed, but no anthropometry is collected, the reason for the missed anthropometry collection should be documented in a PDF.

The site should follow site-specific guidelines when poor growth is identified.

IV. General QC instructions

- QC of the anthropometry measurements will be done using the ANT form – In brief, 5% of the participants will have their anthropometric measurements taken a second time by a different Study Researcher / Nurse / Fieldworker and these measurements will be compared (see ANT SOP for further details).

- 100% of each Study Researcher / Nurse / Fieldworker’s forms should be reviewed by the supervisor on at least a weekly basis, ideally, at the end of each working day. Supervisors should ensure the forms are complete (no missing fields), that the data appear to be correct, and that visits planned for the day were made.

- Corrections to the forms should be minimized (data should be correct the first time), but if needed, the Study Researcher / Nurse / Fieldworker should follow proper correction procedures. If form corrections are necessary:

- - 1. Cross through the incorrect information once,
    2. Write the correct information,
    3. Write the date the correction was made, and
    4. Write their initials.

- Make sure forms are kept confidential and protected (in a locked file drawer, for example) when they are returned from the field.

- Data center transmission of forms

- - Once forms have been completely filled out by the Study Researchers / Nurses / Fieldworkers and reviewed by the supervisor, they must be delivered within 48 hours to the local data center for data entry. Data entry of the forms should occur within one month of delivery to the data center (preferably sooner).

- Standard rounding techniques are recommended: Round down if last digit is <5 and round up if the last digit is >5).

| # | Question | Guidance |
| --- | --- | --- |
|  | Participant ID | Write the child’s participant ID in the space provided at the upper left corner. |
| 01 | Study Researcher / Nurse / Fieldworker ID | Enter the Study Researcher / Nurse / Fieldworker’s unique ID number here. |
| 02 | Today’s date | Format DD/MMM/YY |
| The following questions should be asked about the diet yesterday (from yesterday sunrise until this morning sunrise). This first section asks only about the liquids the child has consumed. | | |
| 03 | Breast milk | Are you breastfeeding <CHILD>? Possible answers: 1=yes or 0=no. If NO, enter ‘NA’ for questions 4 and 5, and then skip to Q.6 |
| 04 | Breast milk during night | Last night, how many times did you breastfeed <CHILD> from sunset to sunrise? Enter the number here. If answer is 'as often as the baby wanted' or 'I don't know' then probe with 'how often did the baby want to nurse, 2 times or 5 times or 10 times?' |
| 05 | Breast milk during day | Yesterday, during the day, how many times did you breastfeed <CHILD>? Enter the number here. If answer is 'as often as the baby wanted' or 'I don't know' then probe with 'how often did the baby want to nurse, 2 times or 5 times or 10 times?' |
| 06 | Infant formula | Do you give <CHILD> infant formula? Possible answers: 1=yes or 0=no. If NO, enter ‘NA’ for questions 7 and 8, and then skip to Q.9 |
| 07 | Infant formula during night | Last night, how many times did you feed <CHILD> formula from sunset to sunrise? Enter the number here. If answer is 'as often as the baby wanted' or 'I don't know' then probe with 'how often did the baby want to eat, 2 times or 5 times or 10 times?' |
| 08 | Infant formula during day | Yesterday, during the day, how many times did you feed <CHILD> formula? Enter the number here. If answer is 'as often as the baby wanted' or 'I don't know' then probe with 'how often did the baby want to eat, 2 times or 5 times or 10 times?' |
| 09 | Animal milk | Do you give <CHILD> other milks, such as canned, powdered or fresh animal milk? Possible answers: 1=yes or 0=no. If NO, enter ‘NA’ for questions 10 and 11, and then skip to Q.12 |
| 10 | Animal milk during night | Last night, how many times did you feed <CHILD> animal milks from sunset to sunrise? Enter the number here. If answer is 'as often as the baby wanted' or 'I don't know' then probe with 'how often did the baby want to eat, 2 times or 5 times or 10 times?' |
| 11 | Animal milk during day | Yesterday, during the day, how many times did you feed <CHILD> animal milk? Enter the number here. If answer is 'as often as the baby wanted' or 'I don't know' then probe with 'how often did the baby want to eat, 2 times or 5 times or 10 times?' |
| 12 | Plain water | Yesterday, during the day or last night, did <CHILD> have plain water? Possible answers: 1=yes or 0=no. |
| 13 | Tea, coffee, local examples | Yesterday, during the day or last night, did <CHILD> have tea or coffee or <local examples>? Possible answers: 1=yes or 0=no. |
| 14 | Fruit juices | Yesterday, during the day or last night, did <CHILD> have any fruit juices? Possible answers: 1=yes or 0=no. |
| 15 | Other liquids | Yesterday, during the day or last night, did <CHILD> have any other liquids, such as sugar water, thin soup or broth, carbonated drinks, <local examples>? Possible answers: 1=yes or 0=no. |
| The following questions are about solid foods that were consumed yesterday (from yesterday sunrise until this morning sunrise). | | |
| 16 | Semi-solid foods | Is <CHILD> eating any semi-solid, mashed or solid foods? If NO, enter ‘NA’ for questions 17 through 31 (can draw a line through the boxes), and skip to Q. 32. If yes, continue to Q.17. |
| Thinking about yesterday, during the day and at night, did <CHILD> have any of the following foods (even if they were in combination with other foods)? | | |
| 17 | Rice, porridge, bread, noodles or other foods/drinks made from grains? | Possible answers: 1=yes or 0=no. |
| 18 | White potatoes, white yams, manioc, or other roots? | Possible answers: 1=yes or 0=no. |
| 19 | Carrots, squash, or sweet potatoes that are yellow or orange inside? | Possible answers: 1=yes or 0=no. |
| 20 | Any dark green leafy vegetables such as spinach? | Possible answers: 1=yes or 0=no. |
| 21 | Foods made with beans, lentils, peas, corn, ground nuts? | Possible answers: 1=yes or 0=no. |
| 22 | Ripe mangoes, papayas, or other sweet yellow, orange, or red fruit? | Possible answers: 1=yes or 0=no. |
| 23 | Any other fruits or vegetables such as banana, apple, oranges, tomatoes, avocado? | Possible answers: 1=yes or 0=no. |
| 24 | Liver, kidney, heart or other organ meats? | Possible answers: 1=yes or 0=no. |
| 25 | Any meat, such as chicken, beef, lamb, goat, duck (others)? | Possible answers: 1=yes or 0=no. |
| 26 | Eggs? | Possible answers: 1=yes or 0=no. |
| 27 | Fresh or dried fish or shellfish? | Possible answers: 1=yes or 0=no. |
| 28 | Cheese, yogurt or other dairy products? | Possible answers: 1=yes or 0=no. |
| 29 | Any sugary foods such as pastries, cakes or biscuits? | Possible answers: 1=yes or 0=no. |
| 30 | Any commercially available foods for infants or young children? | Possible answers: 1=yes or 0=no. |
| 31 | # times child was fed | Enter here the number of times the child was fed yesterday (yesterday sunrise until this morning sunrise) |
| 32 | Child’s appetite | 4='very good' (likes to eat, interested in food, willing to try new foods); 3='good' (shows interest in food, eats well); 2='fair' (sometimes interested in food, doesn't like some foods); 1='poor' (not interested in new foods, likes a few foods, prefers breast milk or milks) |
| 33 | Vitamin A | Possible answers: 1=yes or 0=no. |
| 34 | Vitamin syrups | ‘Regular' is 2+ times/week. Possible answers: 1=yes or 0=no. |
| 35 | Iodized salt | Possible answers: 1=yes or 0=no. |
| 36 | Washed salt | Possible answers: 1=yes or 0=no. |
| 37 | Non-food items | Possible answers: 1=yes or 0=no. |
| Vaccines administered since last visit – Ask to see vaccine records. If vaccine records are available, check to see if any vaccines have been given in the past month. If vaccines have been given since the last monthly visit, document the date the vaccine was given here. If no vaccine records are available, ask the caregiver if the child has received any vaccines since the last monthly visit, and if so, document the type and date of vaccination. If more than one dose was given of a particular vaccine since the last visit (because a monthly visit was missed, for example), use the ‘Other‘ field (questions 50-57) to document the additional dose(s). If you only know the name of the vaccine but you do not know which diseases the vaccine protects against, write down the name of the vaccine on the Nursing Notes Form and discuss with your supervisor. For example, if the mother tells you the child received Pentaxim, you will enter the date for DPT, HiB, and IPV since pentaxim is a combination of all of those vaccines. If you don’t know what vaccines are included in a combination vaccine, ask your supervisor. You will notice there is a Measles option and there is an MMR option – If the child received measles vaccine, you should fill out either the Measles date (Q42) or MMR date (Q45), not both. If the child received only measles vaccine (not in combination with mumps and rubella vaccines), fill out the date for measles. If the child received the combination vaccine with measles, mumps and rubella (MMR), fill out the date for MMR (do not fill out the date for measles alone). | | |
| 38 | BCG date (DD/MMM/YY) | Format DD/MMM/YY |
| 39 | DPT date (DD/MMM/YY) | Format DD/MMM/YY |
| 40 | Hepatitis B date (DD/MMM/YY) | Format DD/MMM/YY |
| 41 | OPV date (DD/MMM/YY) | Format DD/MMM/YY |
| 42 | Measles date (DD/MMM/YY) | Format DD/MMM/YY |
| 43 | Rotavirus date (DD/MMM/YY) | Format DD/MMM/YY |
| 44 | Hib date (DD/MMM/YY) | Format DD/MMM/YY |
| 45 | Measles, Mumps, and Rubella (MMR) date (DD/MMM/YY) | Format DD/MMM/YY |
| 46 | Japanese Encephalitis date (DD/MMM/YY) | Format DD/MMM/YY |
| 47 | Pneumococcal conjugate vaccine (PCV) date | Format DD/MMM/YY |
| 48 | Inactivated polio vaccine (IPV) date | Format DD/MMM/YY |
| 49 | Yellow fever vaccine date | Format DD/MMM/YY |
| 50 | Other (1) | Write in the type of vaccine if not listed above |
| 51 | Date of other (1) | Format DD/MMM/YY |
| 52 | Other (2) | Write in the type of vaccine if not listed above |
| 53 | Date of other (2) | Format DD/MMM/YY |
| 54 | Other (3) | Write in the type of vaccine if not listed above |
| 55 | Date of other (3) | Format DD/MMM/YY |
| 56 | Other (4) | Write in the type of vaccine if not listed above |
| 57 | Date of other (4) | Format DD/MMM/YY |
| 58 | Weight (kg) | Enter here the value obtained by weighing the child in kilograms to at least two decimal places. *Refer to SOP on anthropometry*. |
| 59 | Length (cm) | Enter here the value obtained by measuring the child’s length in centimeters to one decimal place. *Refer to SOP on anthropometry*. |
| 60 | Head circumference (cm) | Enter here the value obtained by measuring the child’s head circumference in centimeters to one decimal place. *Refer to SOP on anthropometry*. |
| 61 | Stool specimen collected? | For this question, enter whether or not the monthly (diarrheal or non-diarrheal) stool sample was collected within the birth date +/- 2 day window prescribed for the monthly visit. If the sample is collected within that window, answer this question 1 (Yes), if it is not collected within the window, answer 0 (No). *Refer to SOP on Monthly Stool Collection for details on how to collect the sample*. **Do not collect monthly stool samples within 48 hours after the administration of the Lactulose Mannitol solution because LM interferes with some of the gut function biomarker assays done with stool samples.** In the event that a stool specimen is not collected within the +/- 2 day window, you should still collect it as soon as possible up to 7 days after the birth date anniversary and treat it as a monthly specimen, but it should be recorded as a protocol deviation using the PDF form. Be sure to also complete a Stool Field Collection (SFC) form for all samples collected. If a diarrheal stool sample was collected from the child within the window for collection for the monthly sample (+/- 2 days of monthly birth date anniversary), it is not necessary to collect a separate monthly stool sample. If the child has diarrhea during the monthly visit and a sample has not been collected for this diarrheal episode in the past two weeks, a stool sample should be collected. If the person conducting the Monthly form data collection does not know whether a diarrheal sample has been collected, they should collect the sample and a decision to process the sample can be made at the laboratory/study center. |
| 62 | Does the child have bipedal edema? | Enter if child has bipedal edema (YES/NO). Refer to SOP on Detection of Bipedal Edema. Format Yes = 01; No= 00. |

MAF—Maternal Assessment Form

I. Purpose

To collect maternal anthropometric information and pregnancy characteristics at the two month visit.

II. Material

Maternal Assessment Form (MAF), Anthropometry SOP and related equipment, pen, clipboard

III. Methods

A Study Researcher / Nurse / Fieldworker trained in measuring anthropometry will administer this form at the two month visit (+/-two days of child turning two months of age). If the window period is missed, collect the data and fill out a PDF.

IV. General QC instructions

- 100% of each Study Researcher / Nurse / Fieldworker’s forms should be reviewed by the supervisor on at least a weekly basis, ideally, at the end of each working day. Supervisors should ensure the forms are complete (no missing fields), that the data appear to be correct, and that visits planned for the day were made.

- Corrections to the forms should be minimized (data should be correct the first time), but if needed, the Study Researcher / Nurse / Fieldworker should follow proper correction procedures. If form corrections are necessary:

- - 1. Cross through the incorrect information once,
    2. Write the correct information,
    3. Write the date the correction was made, and
    4. Write their initials.

- Make sure forms are kept confidential and protected (in a locked file drawer, for example) when they are returned from the field.

- Data center transmission of forms

- - Once forms have been completely filled out by the Study Researchers / Nurses / Fieldworkers and reviewed by the supervisor, they must be delivered within 48 hours to the local data center for data entry. Data entry of the forms should occur within one month of delivery to the data center (preferably sooner).

- Standard rounding techniques are recommended: Round down if last digit is <5 and round up if the last digit is >5).

| # | Question | Guidance |
| --- | --- | --- |
|  | Participant ID | Write the mother’s participant ID in the space provided at the upper left corner. (The mother’s PID is the same as the child’s PID, only with an ‘M’ instead of a ‘C’. For example, if BG1C0001 is the child’s PID, BG1M0001 is the mother’s PID.) |
| 01 | Study Researcher / Nurse / Fieldworker ID | Enter the Study Researcher / Nurse / Fieldworker’s unique ID number here. |
| 02 | Today’s date | Format DD/MMM/YY |
| 03 | Do you have any problems seeing at night or in dim light? | Enter here whether the woman suffered from diminished vision at night (night blindness) while pregnant. This condition should resolve soon after birth. Possible answers: 1=yes or 0=no. |
| 04 | Difficulty with vision during daylight? | Enter here whether the woman had poor vision during daylight. Possible answers: 1=yes or 0=no. |
| 05 | Smoke tobacco while pregnant? | Enter here whether the woman smoked any tobacco while pregnant. Possible answers: 1=yes or 0=no. |
| 06 | Number of cigarettes smoked per day? | Enter here the average number of cigarettes (or other tobacco sources) smoked per day while pregnant. |
| 07 | Drink alcohol while pregnant? | Enter here whether the woman drank any alcohol while pregnant. Possible answers: 1=yes or 0=no. |
| 08 | Number of alcoholic drinks per week? | Enter here the average number of alcoholic drinks the woman had per week while pregnant. |
| 09 | Current weight | Enter here the value obtained by measuring the mother’s weight in kilograms to at least one decimal place. *Refer to SOP on anthropometry*. |
| 10 | Current height | Enter here the value obtained by measuring the mother’s height in centimeters to one decimal place. *Refer to SOP on anthropometry*. |
| Collection of blood and saliva samples from the mothers is a site-specific decision. Some sites may wish to collect samples whereas other sites may not. Please ask the field supervisor if you are expected to collect maternal samples, and if not, simply answer no to questions 11 and 12. | | |
| 11 | Blood sample | Enter here if a blood sample was collected. Possible answers: 1=yes or 0=no. If the answer is yes, please complete the DNA collection form (DCF). |
| 12 | Saliva sample | Enter here if a saliva sample was collected. Possible answers: 1=yes or 0=no. If the answer is yes, please complete the DNA collection form (DCF). |

MOB—Monthly Form B

I. Purpose

To document vaccine history and anthropometry on a monthly basis from nine months to two years of age.

II. Material

Monthly Form B, pen, clipboard, equipment to measure weight, height and head circumference (see Anthropometry SOP), materials for monthly stool specimen collection (see Monthly Stool Specimen Collection SOP)

III. Methods

A Study Researcher / Nurse / Fieldworker trained in measuring anthropometry and dietary assessment will administer this form monthly when the child is nine to twenty-four months of age. Ideally, the fieldworker will visit the home on the anniversary of the child’s date of birth (for a child born on May 17^th^, the fieldworker would visit on June 17^th^, July 17^th^, etc.) but it may not always be possible to visit on the exact day. At times when the exact anniversary date visit is not possible, the form can be filled out within two days before or after (for a child born on May 17^th^, the fieldworker can go between June 15^th^ and June 19^th^ for the one month visit, between July 15^th^ and July 19^th^ for the two month visit, etc.). It is very important that the MOB form be completed within the given +/- two day window; however, if the visit is missed within that window, the Study Researcher / Nurse / Fieldworker may visit the household to collect the MOA / MOB information up to 7 days beyond the birthdate anniversary. If data collection occurs outside the +/- 2-day window, a PDF must be completed.For the FRQ dietary assessment form, there is an allowed window period of upto 15 days to capture a typical day of food intake. If the visit is performed, but no anthropometry is collected, the reason for the missed anthropometry collection should be documented in a PDF.

The site should follow site-specific guidelines when poor growth is identified.

IV. General QC instructions

- QC of the anthropometry measurements will be done using the ANT form – In brief, 5% of the participants will have their anthropometric measurements taken a second time by a different Study Researcher / Nurse / Fieldworker and these measurements will be compared. (see ANT SOP for further details)

- 100% of each Study Researcher / Nurse / Fieldworker’s forms should be reviewed by the supervisor on at least a weekly basis, ideally, at the end of each working day. Supervisors should ensure the forms are complete (no missing fields), that the data appear to be correct, and that visits planned for the day were made.

- Corrections to the forms should be minimized (data should be correct the first time), but if needed, the Study Researcher / Nurse / Fieldworker should follow proper correction procedures. If form corrections are necessary:

- - 1. Cross through the incorrect information once,
    2. Write the correct information,
    3. Write the date the correction was made, and
    4. Write their initials.

- Make sure forms are kept confidential and protected (in a locked file drawer, for example) when they are returned from the field.

- Data center transmission of forms

- - Once forms have been completely filled out by the Study Researchers / Nurses / Fieldworkers and reviewed by the supervisor, they must be delivered within 48 hours to the local data center for data entry. Data entry of the forms should occur within one month of delivery to the data center (preferably sooner).

- Standard rounding techniques are recommended: Round down if last digit is <5 and round up if the last digit is >5).

| # | Question | Guidance |
| --- | --- | --- |
|  | Participant ID | Write the child’s participant ID in the space provided at the upper left corner. |
| 01 | Study Researcher / Nurse / Fieldworker ID | Enter the Study Researcher / Nurse / Fieldworker’s unique ID number here. |
| 02 | Today’s date | Format DD/MMM/YY |
| Vaccines administered since last visit – Ask to see vaccine records. If vaccine records are available, check to see if any vaccines have been given in the past month. If vaccines have been given since the last visit, document the date the vaccine was given here. If no vaccine records are available, ask the caregiver if the child has received any vaccines since the last monthly visit, and if so, document the type and date of vaccination. If more than one dose was given of a particular vaccine since the last visit (because a monthly visit was missed, for example), use the ‘Other‘ field (questions 50-57) to document the additional dose(s). If you only know the name of the vaccine but you do not know which diseases the vaccine protects against, write down the name of the vaccine on the Nursing Notes Form and discuss with your supervisor. For example, if the mother tells you the child received Pentaxim, you will enter the date for DPT, HiB, and IPV since pentaxim is a combination of all of those vaccines. If you don’t know what vaccines are included in a combination vaccine, ask your supervisor. You will notice there is a Measles option and there is an MMR option – If the child received measles vaccine, you should fill out either the Measles date (Q7) or MMR date (Q10), not both. If the child received only measles vaccine (not in combination with mumps and rubella vaccines), fill out the date for measles. If the child received the combination vaccine with measles, mumps and rubella (MMR), fill out the date for MMR (do not fill out the date for measles alone). | | |
| 03 | BCG date (DD/MMM/YY) | Format DD/MMM/YY |
| 04 | DPT date (DD/MMM/YY) | Format DD/MMM/YY |
| 05 | Hepatitis B date (DD/MMM/YY) | Format DD/MMM/YY |
| 06 | OPV date (DD/MMM/YY) | Format DD/MMM/YY |
| 07 | Measles date (DD/MMM/YY) | Format DD/MMM/YY |
| 08 | Rotavirus date (DD/MMM/YY) | Format DD/MMM/YY |
| 09 | Hib date (DD/MMM/YY) | Format DD/MMM/YY |
| 10 | Measles, Mumps, Rubella (MMR) date (DD/MMM/YY) | Format DD/MMM/YY |
| 11 | Japanese Encephalitis date (DD/MMM/YY) | Format DD/MMM/YY |
| 12 | Pneumococcal conjugate vaccine (PCV) date | Format DD/MMM/YY |
| 13 | Inactivated polio vaccine (IPV) date | Format DD/MMM/YY |
| 14 | Yellow fever vaccine date | Format DD/MMM/YY |
| 15 | Other (1) | Write in the type of vaccine if not listed above |
| 16 | Date of other (1) | Format DD/MMM/YY |
| 17 | Other (2) | Write in the type of vaccine if not listed above |
| 18 | Date of other (2) | Format DD/MMM/YY |
| 19 | Other (3) | Write in the type of vaccine if not listed above |
| 20 | Date of other (3) | Format DD/MMM/YY |
| 21 | Other (4) | Write in the type of vaccine if not listed above |
| 22 | Date of other (4) | Format DD/MMM/YY |
| 23 | Weight (kg) | Enter here the value obtained by weighing the child in kilograms to at least one decimal place. *Refer to SOP on anthropometry*. |
| 24 | Length (cm) | Enter here the value obtained by measuring the child’s length in centimeters to one decimal place. *Refer to SOP on anthropometry*. |
| 25 | Head circumference (cm) | Enter here the value obtained by measuring the child’s head circumference in centimeters to one decimal place. *Refer to SOP on anthropometry*. |
| 26 | Stool specimen collected? | For this question, enter whether or not the monthly (diarrheal or non-diarrheal) stool sample was collected within the birth date +/- 2 day window prescribed for the monthly visit. If the sample is collected within that window, answer this question 1 (Yes), if it is not collected within the window, answer 0 (No). *Refer to SOP on Monthly Stool Collection for details on how to collect the sample*. **Do not collect monthly stool samples within 48 hours after the administration of the Lactulose Mannitol solution because LM interferes with some of the gut function biomarker assays done with stool samples.** In the event that a stool specimen is not collected within the +/- 2 day window, you should still collect it as soon as possible up to 7 days after the birth date anniversary and treat it as a monthly specimen, but it should be recorded as a protocol deviation using the PDF form. Be sure to also complete a Stool Field Collection (SFC) form for all samples collected. If a diarrheal stool sample was collected from the child within the window for collection for the monthly sample (+/- 2 days of monthly birth date anniversary), it is not necessary to collect a separate monthly stool sample. If the child has diarrhea during the monthly visit and a sample has not been collected for this diarrheal episode in the past two weeks, a stool sample should be collected. If the person conducting the Monthly form data collection does not know whether a diarrheal sample has been collected, they should collect the sample and a decision to process the sample can be made at the laboratory/study center. |
| 27 | Does the child have bipedal edema? | Enter if child has bipedal edema (YES/NO). Refer to SOP on Detection of Bipedal Edema. Format Yes = 01; No= 00. |

MOC—Monthly Form C

I. Purpose

To document dietary pattern, vaccine history, and anthropometry on a monthly basis from 25 to 36 months of age.

II. Material

Monthly Form C, pen, clipboard, equipment to measure weight, height and head circumference (see Anthropometry SOP)

III. Methods

The Study Researcher / Nurse / Fieldworker trained in measuring anthropometry and dietary assessment will administer this form monthly when the child is 25 to 36 months of age. Ideally, the fieldworker will visit the home on the anniversary of the child’s date of birth (for a child born on May 17^th^, the fieldworker would visit on June 17^th^, July 17^th^, etc.) but it may not always be possible to visit on the exact day. At times when the exact anniversary date visit is not possible, the form can be filled out within two days before or after (for a child born on May 17^th^, the fieldworker can go between June 15^th^ and June 19^th^ for the 25 month visit, between July 15^th^ and July 19^th^ for the 26 month visit, etc.). It is very important that the form be completed within the given +/- two day window; however, if the visit is missed within that window, the Study Researcher / Nurse / Fieldworker may visit the household to collect the MOC information up to 7 days beyond the birthdate anniversary. If data collection occurs outside the +/- 2 day window, a PDF form must be completed. If the visit is performed, but no anthropometry is collected, the reason for the missed anthropometry collection should be documented in a PDF.

The site should follow site-specific guidelines when poor growth is identified.

The 24 hour recall should not be conducted on the same day as the MOC.

IV. General QC instructions

QC of the anthropometry measurements will be done using the ANT form – In brief, 5% of the participants will have their anthropometric measurements taken a second time by a supervisor or someone with a high level of expertise in the collection of anthropometric data and these measurements will be compared (see ANT SOP for further details). Second measurements will be considered to be the gold standard. For the three months when both height and length will be collected, only the height and weight need to be collected. In other words, you do not need to collect both the length and the height for the ANT in the 25-36 month period – only the height (and the weight) need to be collected.

- 100% of each Study Researcher / Nurse / Fieldworker’s forms should be reviewed by the supervisor on at least a weekly basis, ideally, at the end of each working day. Supervisors should ensure the forms are complete (no missing fields), that the data appear to be correct, and that visits planned for the day were made.

- Corrections to the forms should be minimized (data should be correct the first time), but if needed, the Study Researcher / Nurse / Fieldworker should follow proper correction procedures. If form corrections are necessary:

i. Cross through the incorrect information once,

ii. Write the correct information,

iii. Write the date the correction was made, and

iv. Write their initials.

- Make sure forms are kept confidential and protected (in a locked file drawer, for example) when they are returned from the field.

- Data center transmission of forms

- - Once forms have been completely filled out by the Study Researchers / Nurses / Fieldworkers and reviewed by the supervisor, they must be delivered within 48 hours to the local data center for data entry. Data entry of the forms should occur within one month of delivery to the data center (preferably sooner).

- Standard rounding techniques are recommended: Round down if last digit is <5 and round up if the last digit is >5).

| # | Question | Guidance |
| --- | --- | --- |
|  | Participant ID | Write the child’s participant ID in the space provided at the upper left corner. |
| 01 | Study Researcher / Nurse / Fieldworker ID | Enter the Study Researcher / Nurse / Fieldworker’s unique ID number here. |
| 02 | Today’s date | Format DD/MMM/YY |
| The following questions should be asked about the diet yesterday (from yesterday sunrise until this morning sunrise). This first section asks only about the liquids the child has consumed. | | |
| Yesterday, during the day or last night, did <CHILD> have any of the following liquids? | | |
| 03 | Breast milk | Possible answers: 1=yes or 0=no. |
| 04 | Animal milk (canned, powdered or fresh) | Possible answers: 1=yes or 0=no. |
| 05 | Plain water | Possible answers: 1=yes or 0=no. |
| 06 | Tea, coffee, local examples | Possible answers: 1=yes or 0=no. |
| 07 | Fruit or vegetable juices | Possible answers: 1=yes or 0=no. |
| 08 | Carbonated beverages (Cola, Fanta, local examples)? | Possible answers: 1=yes or 0=no. |
| 09 | Thin soup or broth? | Possible answers: 1=yes or 0=no. |
| The following questions are about solid foods that were consumed yesterday (from yesterday sunrise until this morning sunrise). | | |
| Thinking about yesterday, during the day and at night, did <CHILD> have any of the following foods (even if they were in combination with other foods)? | | |
| 10 | Rice, porridge, bread, noodles or other foods made from grains? | Possible answers: 1=yes or 0=no. |
| 11 | White potatoes, white yams, manioc, or other roots? | Possible answers: 1=yes or 0=no. |
| 12 | Carrots, squash, or sweet potatoes that are yellow or orange inside? | Possible answers: 1=yes or 0=no. |
| 13 | Any dark green leafy vegetables such as spinach? | Possible answers: 1=yes or 0=no. |
| 14 | Foods made with beans, lentils, peas, corn, ground nuts? | Possible answers: 1=yes or 0=no. |
| 15 | Ripe mangoes, papayas, or other sweet yellow, orange, or red fruit? | Possible answers: 1=yes or 0=no. |
| 16 | Any other fruits or vegetables such as banana, apple, orange, tomato, avocado? | Possible answers: 1=yes or 0=no. |
| 17 | Liver, kidney, heart or other organ meats? | Possible answers: 1=yes or 0=no. |
| 18 | Any meat, such as chicken, beef, lamb, goat, duck (others)? | Possible answers: 1=yes or 0=no. |
| 19 | Eggs? | Possible answers: 1=yes or 0=no. |
| 20 | Fish or shellfish? (fresh, canned or dried) | Possible answers: 1=yes or 0=no. |
| 21 | Cheese, yogurt or other dairy products? | Possible answers: 1=yes or 0=no. |
| 22 | Any sugary foods such as pastries, cakes, biscuits or jam? | Possible answers: 1=yes or 0=no. |
| 23 | Margarine, butter or ghee? (as a spread) | Possible answers: 1=yes or 0=no. If ghee is not eaten in your site, do not ask about it. This question refers to ‘oil’ (ie, butter, margarine, ghee) as a spread (eg, on bread). |
| 24 | # times child was fed | Enter here the number of times the child was fed yesterday (yesterday sunrise until this morning sunrise) |
| 25 | After cooking, was oil (or butter or ghee) added to child’s food? | Possible answers: 1=yes or 0=no. This question refers to ‘oil’ (ie, butter, margarine, ghee) as a condiment or addition to cooked food. |
| 26 | Was yesterday a typical day? | Possible answers: 1=yes, 2=no, the child was sick, 3=no, yesterday was a holiday/celebration, 4=no, other  This question is meant to determine whether the diet described in the previous questions was typical for the child, or if yesterday was unusual. |
| 27 | Other text | If the answer to question 26 was 4=no, other, write in what made yesterday different here. |
| 28 | Child’s appetite | 4='very good' (likes to eat, interested in food, willing to try new foods); 3='good' (shows interest in food, eats well); 2='fair' (sometimes interested in food, doesn't like some foods); 1='poor' (not interested in new foods, likes only a few foods) |
| 29 | Vitamin A | Possible answers: 1=yes or 0=no. |
| 30 | Vitamin syrups | ‘Regular' is 2+ times/week. Possible answers: 1=yes or 0=no. |
| 31 | Iodized salt | Possible answers: 1=yes or 0=no. |
| 32 | Washed salt | Possible answers: 1=yes or 0=no. |
| 33 | Non-food items | Possible answers: 1=yes or 0=no. |
| 34 | Caregivers | This question is meant to assess the different people who took care of the child yesterday. Individuals should have cared for the child for more than 15 minutes to be listed here. Fill in as many caregivers as are named by the respondent. Options: 01= Mother; 02=Sibling; 03=Other relative in household; 04=Relative outside the household; 05=Unpaid non-relative; 06=Paid childcare (nanny, babysitter); 07=Household staff (not childcare specific, like a housekeeper or cook); 08=Preschool; 09=Other. |
| 35 | Provided information about food yesterday | This question is meant to assess the different people who reported food consumption of the child yesterday. Fill in as many people as are named by the respondent. Options: 01= Mother; 02=Sibling; 03=Other relative in household; 04=Relative outside the household; 05=Unpaid non-relative; 06=Paid childcare (nanny, babysitter); 07=Household staff (not childcare specific, like a housekeeper or cook); 08=Preschool; 09=Other. |
| Vaccines administered since last visit – Ask to see vaccine records. If vaccine records are available, check to see if any vaccines have been given in the past month. If vaccines have been given since the last monthly visit, document the date the vaccine was given here. If no vaccine records are available, ask the caregiver if the child has received any vaccines since the last monthly visit, and if so, document the type and date of vaccination. If more than one dose was given of a particular vaccine since the last visit (because a monthly visit was missed, for example), use the ‘Other‘ field (questions 50-57) to document the additional dose(s). If you only know the name of the vaccine but you do not know which diseases the vaccine protects against, write down the name of the vaccine on the Nursing Notes Form and discuss with your supervisor. For example, if the mother tells you the child received Pentaxim, you will enter the date for DPT, HiB, and IPV since pentaxim is a combination of all of those vaccines. If you don’t know what vaccines are included in a combination vaccine, ask your supervisor. You will notice there is a Measles option and there is an MMR option – If the child received measles vaccine, you should fill out either the Measles date (Q42) or MMR date (Q45), not both. If the child received only measles vaccine (not in combination with mumps and rubella vaccines), fill out the date for measles. If the child received the combination vaccine with measles, mumps and rubella (MMR), fill out the date for MMR (do not fill out the date for measles alone). | | |
| 36 | BCG date (DD/MMM/YY) | Format DD/MMM/YY |
| 37 | DPT date (DD/MMM/YY) | Format DD/MMM/YY |
| 38 | Hepatitis B date (DD/MMM/YY) | Format DD/MMM/YY |
| 39 | OPV date (DD/MMM/YY) | Format DD/MMM/YY |
| 40 | Measles date (DD/MMM/YY) | Format DD/MMM/YY |
| 41 | Rotavirus date (DD/MMM/YY) | Format DD/MMM/YY |
| 42 | Hib date (DD/MMM/YY) | Format DD/MMM/YY |
| 43 | Measles, Mumps, and Rubella (MMR) date (DD/MMM/YY) | Format DD/MMM/YY |
| 44 | Japanese Encephalitis date (DD/MMM/YY) | Format DD/MMM/YY |
| 45 | Pneumococcal conjugate vaccine (PCV) date | Format DD/MMM/YY |
| 46 | Inactivated polio vaccine (IPV) date | Format DD/MMM/YY |
| 47 | Yellow fever vaccine date | Format DD/MMM/YY |
| 48 | Other (1) | Write in the type of vaccine if not listed above |
| 49 | Date of other (1) | Format DD/MMM/YY |
| 50 | Other (2) | Write in the type of vaccine if not listed above |
| 51 | Date of other (2) | Format DD/MMM/YY |
| 52 | Other (3) | Write in the type of vaccine if not listed above |
| 53 | Date of other (3) | Format DD/MMM/YY |
| 54 | Other (4) | Write in the type of vaccine if not listed above |
| 55 | Date of other (4) | Format DD/MMM/YY |
| Anthropometry | | |
| 56 | Weight (kg) | Enter here the value obtained by weighing the child in kilograms to at least two decimal places. *Refer to SOP on anthropometry*. |
| Note about length and height: Due to the different values obtained when measuring length and height, both need to be measured for at least the first three months of MOC for each child. Three months of both height and length measurements are needed – preferably in months 25-27, but if those months are missed, any three months are okay (earlier preferred). This will allow us to generate a participant-specific correction to the measurement and will allow us to model linear growth continuously throughout the 36-month period. After three months of measuring both length and height, only height is required (although sites are welcome to continue measuring both height and length beyond that time). | | |
| 57 | Length (cm) | Enter here the value obtained by measuring the child’s length in centimeters to one decimal place. *Refer to SOP on anthropometry*. |
| 58 | Height (cm) | Enter here the value obtained by measuring the child’s height in centimeters to one decimal place. *Refer to SOP on anthropometry*. |
| 59 | Head circumference (cm) | Enter here the value obtained by measuring the child’s head circumference in centimeters to one decimal place. *Refer to SOP on anthropometry*. |
| 60 | Does the child have bipedal edema? | Enter if child has bipedal edema (YES/NO). Refer to SOP on Detection of Bipedal Edema. Format Yes = 01; No= 00. |

ANT – Anthropometry Check Form

I. Purpose

Assess measurement error and identify training needs

II. Summary

Each month, a senior study nurse or field worker well-trained in anthropometry and who will be considered the gold standard should visit 5% of the households within 24 hours of the Monthly form data collection (MOA, MOB, MOC) in order to collect duplicate anthropometric measurements for quality control purposes. The 5% sample should be random and representative of the participants’ ages, locations, and all other variables.

III. Methods

Every month, the weight, length (height in 25-36m time period), and head circumference of 5% of the children will be collected using the ANT form and entered into the database. These anthropometric measurements will be used to assess measurement error and identify the need for retraining of staff. At the beginning of each month, the supervisor will calculate the number of participants in the study and then schedule quality control visits for a random sample of 5% of the participants to occur within 24 hours of regular administration of the MOA, MOB, or MOC. Only length should be measured in 0-24 month time period, and only height will be measured in the ANT in the 25-36 month time period (length need not be measured for the ANT in 25-36 months, even if both length and height may be measured in the MOC).

IV. Additional notes

The supervisor should review the ANT and MOA/MOB/MOC forms at the end of the day (or on a weekly basis) and discuss discrepancies found with the Study Researcher / Nurse / Fieldworker and provide additional training if necessary. Do not change the data that the Study Researcher / Nurse / Fieldworker collected!

V. Inconsistencies

The original data collected by the Study Researcher / Nurse / Fieldworker will be entered into the database. There is no need to ‘correct’ the data based on the second data collector’s responses. This comparison is done simply to guarantee that the Study Researcher / Nurse / Fieldworker is making the visits and measuring the children, and for us to calculate measurement error and identify opportunities for additional training.

1. Minor inconsistencies

The supervisor should discuss the different measures with the Study Researcher / Nurse / Fieldworker. In many cases, the different measures may be due to the child’s state of mind, whether they were fidgeting or hungry or energetic and not wanting to be still for measurement. A small degree of variation is normal and does not need to cause concern.

1. Major inconsistencies

If there are large differences between the measurements (greater than 3 cm in length, 1 cm in head circumference, or 500 g in weight), the supervisor should ensure that a refresher training is completed by both Study Researcher / Nurse / Fieldworkers if both measurements were taken by Study Researcher / Nurse / Fieldworkers, or if the QC measurement was taken by the trainer (gold standard), the trainer should conduct the refresher training with the Study Researcher / Nurse / Fieldworker. If a Study Researcher / Nurse / Fieldworker consistently returns data that poorly measures anthropometry, and does not improve after additional training, that Study Researcher / Nurse / Fieldworker should be replaced.

VI. General QC instructions

- 100% of each Study Researcher / Nurse / Fieldworker’s forms should be reviewed by the supervisor on at least a weekly basis, ideally, at the end of each working day. Supervisors should ensure the forms are complete (no missing fields), that the data appear to be correct, and that visits planned for the day were made.

- Corrections to the forms should be minimized (data should be correct the first time), but if needed, the Study Researcher / Nurse / Fieldworker should follow proper correction procedures. If form corrections are necessary:

- - 1. Cross through the incorrect information once,
    2. Write the correct information,
    3. Write the date the correction was made, and
    4. Write their initials.

- Make sure forms are kept confidential and protected (in a locked file drawer, for example) when they are returned from the field.

- Data center transmission of forms

- - Once forms have been completely filled out by the Study Researchers / Nurses / Fieldworkers and reviewed by the supervisor, they must be delivered within 48 hours to the local data center for data entry. Data entry of the forms should occur within one month of delivery to the data center (preferably sooner).

- Standard rounding techniques are recommended: Round down if last digit is <5 and round up if the last digit is >5).

| # | Question | Guidance |
| --- | --- | --- |
|  | Participant ID | Write the child’s participant ID in the space provided at the upper left corner. |
| 01 | Study Researcher / Nurse / Fieldworker ID | Enter the Study Researcher / Nurse / Fieldworker’s unique ID number here. |
| 02 | Today’s date | Format DD/MMM/YY |
| 03 | Weight (kg) | Enter here the value obtained by weighing the child in kilograms to at least one decimal place. *Refer to SOP on anthropometry*. |
| 04 | Length (cm) in 0-24 months, height (cm) in 25-36 months | Enter here the value obtained by measuring the child’s length (0-24 months) or height (25-36 months) in centimeters to one decimal place. *Refer to SOP on anthropometry*. |
| 05 | Head circumference (cm) | Enter here the value obtained by measuring the child’s head circumference in centimeters to one decimal place. *Refer to SOP on anthropometry*. |

NCF—Non-Continuation Form SOP

I. Purpose

To collect information about the reason a study participant is lost to follow up.

II. Material

Non-Continuation form, pen, clipboard

III. Methods

The Study Researcher / Nurse / Fieldworker will fill out this forms if:

- the primary caretaker would like to withdraw the child from the study, or

- the child’s family decides to move out of the surveillance area, or

- the child dies during follow up, or

- the child is less than 24 months of age and the primary caretaker is not available to answer questions for more than 60 days and their whereabouts are unknown.

- the mother is enrolled in the BMMI study and decides to discontinue.

The supervisor is encouraged to identify specific reasons for dropping out of the study and to put into place measures to minimize additional drop outs.

IV. General QC instructions

- 100% of each Study Researcher / Nurse / Fieldworker’s forms should be reviewed by the supervisor on at least a weekly basis, ideally, at the end of each working day. Supervisors should ensure the forms are complete (no missing fields), that the data appear to be correct, and that visits planned for the day were made.

- Corrections to the forms should be minimized (data should be correct the first time), but if needed, the Study Researcher / Nurse / Fieldworker should follow proper correction procedures. If form corrections are necessary:

- - 1. Cross through the incorrect information once,
    2. Write the correct information,
    3. Write the date the correction was made, and
    4. Write their initials.

- Make sure forms are kept confidential and protected (in a locked file drawer, for example) when they are returned from the field.

- Data center transmission of forms

Once forms have been completely filled out by the Study Researchers / Nurses / Fieldworkers and reviewed by the supervisor, they must be delivered within 48 hours to the local data center for data entry. Data entry of the forms should occur within one month of delivery to the data center (preferably sooner).

| # | Question | Guidance |
| --- | --- | --- |
|  | Participant ID | Write the participant ID in the space provided at the upper left corner.  - If the child is only enrolled in the MAL-ED study use the **child’s** PID.  - If the child is enrolled in both the MAL-ED study and the BMMI study, and the mother discontinues the BMMI study but the child remains in the MAL-ED cohort, use the **mother’s** PID.  -If the child is enrolled in both the MAL-ED study and the BMMI study, and the child drops out of both the MAL-ED and BMMI studies, use the **child’s** PID. |
| 01 | Study Researcher / Nurse / Fieldworker ID | Enter the Study Researcher / Nurse / Fieldworker ID number here. |
| 02 | Today’s date | Format DD/MMM/YY |
| 03 | Reason for leaving the study | Document the reason for leaving the study here.  Dropped out=00: If the subject is being withdrawn from the study, enter 00 and probe for reasons why they do not want to participate. Write any reasons they give for dropping out in the notes section of the form.  Moved=01 - If they are moving, determine if they are moving out of the study area. If they move within the study area, we can continue to follow them. If they are moving out of the study area, enter 01.  Dead=02 If the subject dies, enter 02.  Unknown=03 - If the child is less than 24 months of age and both the child and the primary caretaker have not been available for 60 days or more and you cannot get information about where they are, enter 03. |
| 04 | Date of last contact | Enter the date of last contact with the study subject. Format DD/MMM/YY. |
| 05 | Dropping out from which study? | MALED Cohort = Enter “01” if the participant is dropping out of cohort study and never consented for BMMI study or enrolled before BMMI study started.  BMMI = Enter “02” If the participant is only dropping out of BMMI study but will remain part of the cohort study  BMMI & MALED Cohort = Enter “03” If the participant is dropping out of both BMMI and the Cohort study. |
|  | Notes | Enter notes here, if relevant. Notes can include: reasons for leaving the study, new address if available, cause of death, any additional information. |

QCS—Surveillance Quality Monitoring: Guidelines for Field Supervisors

I. Purpose

To provide general guidelines and minimum standards for surveillance data collection quality control activities by the field supervisor.

II. Methods

The field supervisor will supervise a team of Study Researchers / Nurses / Fieldworkers / Data Collectors who will administer all of the surveillance forms at the appropriate times. The field supervisor should use locally appropriate management techniques in order to ensure complete, accurate and timely data collection.

The field supervisor should monitor:

1. Training
   - The supervisor should ensure that all staff is appropriately trained to collect the data.
   - The supervisor should be aware of changes to forms and procedures and ensure timely retraining of staff (within one week of announcement of changes).
   - The supervisor should review all forms collected by staff and identify inconsistencies or mistakes that indicate retraining is necessary.
   - The supervisor should hold periodic (at least quarterly) refresher training sessions with a focus on data collection topics identified as problem areas. For example, if retention is becoming a problem, the supervisor should organize a training session on how to keep participants in the study. During that training session, staff can brainstorm about retention methods or (no- or very low-cost) incentives that might be offered in order to increase retention, or the supervisor could bring in an expert from the local university to discuss these issues.
2. Recruitment
   - The supervisor should ensure adequate and representative recruitment occurs throughout the study (on average between 9 and 18 recruited per month, representative of larger population). It is important to recruit approximately equal numbers throughout the year so that we do not have large numbers of children born in one season and few born in another. Based on a final number of 200 children at the end of two years, we think 9-18 children per month is a good goal.

c) Enrollment

- When enrolling a mother/child, the following forms are needed:

PID log

SCR

Consent

CAF

DAF

FSQ

SAF

(REF, NUR, NPF may be needed as well)

If the mother does not want to participate or if the child is ineligible, fill out the NPF and do not fill out any other forms.


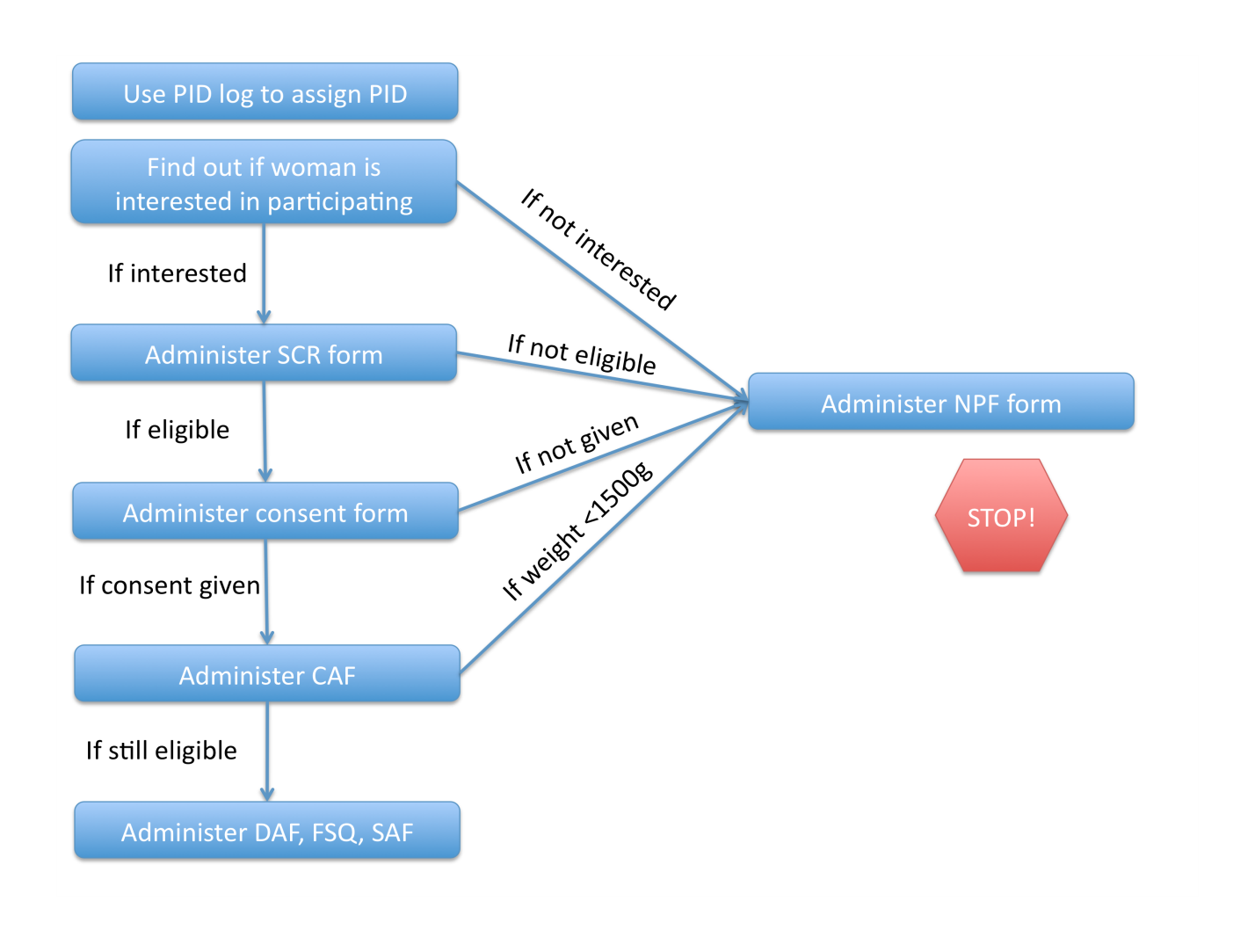


d) Data collection

Ideal collection windows for key forms/samples

| Forms | Form name | Month(s) | Visit range |
| --- | --- | --- | --- |
| BCH | Blood collection | 7, 15 | DOB - 2/+12 DAYS |
| BSD | Bayley Scales | 6, 15, 24, 36 | DOB (+/-) 15 DAYS |
| FRQ | 24 hour food recall | 9 TO 24 (25-36 optional) | DOB -2 /+15 DAYS |
| FSE | Follow-up SES | 6, 12, 18, 24, 30, 36 | DOB (+/-) 15 DAYS |
| FSQ | Food security questionnaire | 6, 12,18,24, 30, 36 | DOB (+/-) 15 DAYS |
| HIT | HOME | 6, 24 | DOB (+/-) 15 DAYS |
| ITS | Infant temperament scale | 6 | DOB (+/-) 15 DAYS |
| MAF | Maternal assessment form | 2 | DOB (+/-) 2 DAYS |
| MOA | Monthly form A | 1 TO 8 | DOB (+/-) 2 DAYS |
| MOB | Monthly form B | 9 TO 24 | DOB (+/-) 2 DAYS |
| MOC | Monthly form C | 25 to 36 | DOB (+/-) 2 DAYS |
| MWG | MacArthur words and gestures | 8, 15 | DOB (+/-) 15 DAYS |
| RCM | Raven's combined matrices | 6-8 | DOB (+/-) 15 DAYS |
| SFC | Stool field collection | 1 TO 24 | DOB (+/-) 2 DAYS |
| SRQ | SRQ-20 | 1,6,15, 24, 36 | DOB (+/-) 15 DAYS |
| UCF | Urine collection form | 3,6,9,15 | DOB -2/+7 DAYS |

- - Organize schedules for Study Researchers / Nurses / Fieldworkers in order to ensure all data are collected on each participant in a timely manner (see schedule of forms/sample collection above). Data should be collected within the collection windows identified for each form, and if data are collected outside the window, a PDF should be filled out.
  - Review 100% of each Study Researcher / Nurse / Fieldworker’s forms at least on a weekly basis, ideally, at the end of each working day. Supervisors should ensure the forms are complete (no missing fields), that the data appear correct, and that visits planned for the day / week were made.
  - Corrections to the forms should be minimized (data should be correct the first time), but if needed, the supervisor should ensure that the Study Researcher / Nurse / Fieldworker follows proper correction procedures. If form corrections are necessary, the data collector should:
    1. Cross through the incorrect information with one line only,
    2. Write the correct information,
    3. Write the date the correction was made, and
    4. Write their initials.
  - Make sure forms are kept confidential and protected (in a locked file drawer, for example) when they are returned from the field.
  - Specific quality control plans have been developed for the SAF and for anthropometry (as part of the forms: MOA and MOB). Apart from the general quality control methods listed above (supervisor review of 100% of forms at the end of each day) there are no specific quality control plans for other forms.
  - The QC for the SAF and MOA/MOB forms can be done during a single visit as long as the SAF data are collected on that day and the MOA/MOB data are collected within 24 hours of the ANT data
  - Supervisors should keep up-to-date on changes in the SOPs and CRFs and retrain staff as needed. Frequent meetings, preferably weekly, should be held with the data collection staff in order to provide form or procedural updates to the staff, talk about any issues that have been observed, and in general, touch base with the staff and identify any questions or training needs.
  - Real-time check of anthropometry – In order to correct errors in anthropometry measurement in a timely manner, supervisors (or field workers) are encouraged to print out growth charts and draw points on the chart for each measurement. If the point is wildly inconsistent with the previous points (decrease of more than 2 centimeters in height, gain/loss of approximately more than 1 z-score of height or weight), prompt remeasurement (within 3 days of original measurement) is recommended. Growth charts can be found in the forms appendix and here: <http://www.who.int/childgrowth/standards/en/>. If remeasurement occurs and the original value was approximately correct, it should remain as originally written. If the original value was grossly inaccurate (>10% difference), replace with the more accurate value as long as the remeasurement occurs within one week (preferably sooner) of the original measurement. Follow standard cross-out procedures, and add a note with the date that remeasurement occurred to the form. If the child is very underweight or stunted, please follow your site-specific guidelines for managing/referring these children.

Data center transmission of forms - Once forms have been completely filled out by the Study Researchers / Nurses / Fieldworkers and reviewed / initialed by the supervisor, they must be delivered within five days to the local data center for data entry. Data entry of the forms should occur within one month of delivery to the data center (preferably sooner).

1. Database review
   1. After the data are entered, reports will be generated by the DCC so that supervisors can review the collected data for inconsistencies and errors. (e.g., With your knowledge of the expected diarrhea rates in the area, are the numbers what you expect them to be? Are the diarrhea rates similar across Study Researchers / Nurses / Fieldworkers? Etc.) Quality control reports can be generated more frequently at the local level if desired.

#### Surveillance Assessment Form – Quality Control Plan

I. Purpose

To allow supervisors to ensure that Study Researcher / Nurse / Fieldworkers are making their scheduled visits and obtaining accurate data.

II. Summary

The supervisor (or designee) visits the household and collects Surveillance Assessment Form data (XAF – the supervisor’s version of the SAF) for comparison with regular Study Researcher / Nurse / Fieldworker’s data collection.

III. Number: 10% of participants

The supervisor must visit 10% of the participants one time each month. At the beginning of each month, the supervisor (or supervisor’s designee) will calculate the total number of participants enrolled in the study and make plans to visit at least 10% of those households one time to administer the XAF.

IV. Methods

One suggested process is that the supervisor (or their designee) chooses a random day of the month to visit all households (or a random sample) that are scheduled to receive a Study Researcher / Nurse / Fieldworker visit on that day. The supervisor should not tell the Study Researcher / Nurse / Fieldworker which day the check will happen, nor should they be at the house at the exact same time. The supervisor may need to choose additional days for data collection checks if the total visits possible on one day do not total at least 10% of the participants currently enrolled in the study. In order to standardize the process, the supervisor will ask about illnesses on the last three days. Some sites may want to make SAF QC visits evenly over the month and that is fine as well. The 10% sample should be random and representative of the participants’ ages, locations, and all other variables.

V. Additional Notes

If the supervisor would like to check a higher percent of the total participants, particularly in the beginning or when a new Study Researcher / Nurse / Fieldworker is hired, they are encouraged to do so.

The supervisor should review the XAF and SAF forms, discuss discrepancies found with the Study Researcher / Nurse / Fieldworker, and provide additional training if necessary. Do not change the data that the Study Researcher / Nurse / Fieldworker collected!

VI. Inconsistencies

The original data collected by the Study Researcher / Nurse / Fieldworker will be entered into the database. There is no need to ‘correct’ the data based on the supervisor’s responses. This comparison is done simply to guarantee that the Study Researcher / Nurse / Fieldworker is administering the form correctly and consistently in the field.

1. Minor inconsistencies

The supervisor should discuss the different responses with the Study Researcher / Nurse / Fieldworker. In many cases, the different answers may be due entirely to the respondent’s mood or ability to recall information at that moment. For instance, the first time the mother answers the question about liquid stools, she might answer 2, but the second time, she might have thought more about it and remembered another liquid stool on that day, so she answered 3 the second time. This is normal and does not need to cause concern.

1. Major inconsistencies

If, for example, the Study Researcher / Nurse / Fieldworker records no illness on a certain day but the supervisor records that the mother reported diarrhea, antibiotics, fever and a cough, the supervisor should attempt to verify that the Study Researcher / Nurse / Fieldworker is making their visits as scheduled and that they are asking the questions in a way that encourages complete answers. Major inconsistencies can be defined as more than 15% of the total answers that are discrepant. If a Study Researcher / Nurse / Fieldworker consistently returns data that poorly correlates with the supervisor’s data, and does not improve after additional training, that Study Researcher / Nurse / Fieldworker should be replaced.

Example: In month 6, as of July 1st, there are 72 children enrolled in the study. In July, the supervisor will visit seven of the households and collect XAF data.

The supervisor (or designee) can initially choose one day and one Study Researcher / Nurse / Fieldworker, and will visit every household on that Study Researcher / Nurse / Fieldworker’s schedule for the day.

The supervisor only needs to ask the questions on the form and does not need to actually collect diarrheal stool samples; however if a stool sample was indicated due to presence of diarrhea, the supervisor should answer question 17 on the XAF yes (01) and ensure that the Study Researcher / Nurse / Fieldworker actually collected a sample.

If the supervisor only visited 4 households on the initial QC visiting day, he or she can choose another day during the month to visit additional households until he or she has visited at least 10%. In this case, 10% is 7.2 – it is allowable to round down to 7 households. If the number were 7.6, 8 households would be required.

If the supervisor completed a visit, but the Study Researcher / Nurse / Fieldworker was unable to complete the visit on that day, the supervisor’s data should be discarded and not used for quality control. Visits must occur on the same day for a valid comparison.

If the supervisor chooses to schedule visits throughout the month, they would visit approximately two households each week to complete the XAF.

#### Anthropometry Quality Control Plan

I. Purpose

Assess measurement error and identify training needs

II. Summary

Each month, a study nurse or field worker trained in anthropometry should visit 5% of the households within 24 hours of the Monthly form data collection (MOA, MOB, MOC) in order to collect duplicate anthropometric measurements for quality control purposes. The 5% sample should be random and representative of the participants’ ages, locations, and all other variables.

III. Methods

Every month, the weight, length (0-24m) or height (25-36m), and head circumference of 5% of the children will be collected using the ANT form and entered into the database. These anthropometric measurements will be used to assess measurement error and identify the need for retraining of staff. At the beginning of each month, the supervisor will calculate the number of participants in the study and then schedule quality control visits for a random sample of 5% of the participants to occur within 24 hours of regular administration of the MOA/MOB/MOC.

IV. Notes

The supervisor should review the ANT and MOA/MOB/MOC forms at the end of the day (or on a weekly basis) and discuss discrepancies found with the Study Researcher / Nurse / Fieldworker and provide additional training if necessary. Do not change the data that the Study Researcher / Nurse / Fieldworker collected!

V. Inconsistencies

The original data collected by the Study Researcher / Nurse / Fieldworker will be entered into the database. There is no need to ‘correct’ the data based on the second data collector’s responses. This comparison is done simply to guarantee that the Study Researcher / Nurse / Fieldworker is making the visits and measuring the children, and for us to calculate measurement error and identify opportunities for additional training.

1. Minor inconsistencies

The supervisor should discuss the different measures with the Study Researcher / Nurse / Fieldworker. In many cases, the different measures may be due to the child’s state of mind, whether they were fidgeting or hungry or energetic and not wanting to be still for measurement. A small degree of variation is normal and does not need to cause concern.

1. Major inconsistencies

If there are large differences between the measurements (greater than 3 cm in length, 1 cm in head circumference, or 500 g in weight), the supervisor should ensure that a refresher training is completed by both Study Researcher / Nurse / Fieldworkers if both measurements were taken by Study Researcher / Nurse / Fieldworkers, or if the QC measurement was taken by the trainer (gold standard), the trainer should conduct the refresher training with the Study Researcher / Nurse / Fieldworker. If a Study Researcher / Nurse / Fieldworker consistently returns data that poorly measures anthropometry, and does not improve after additional training, that Study Researcher / Nurse / Fieldworker should be replaced.

#### Supervisor checklist

The supervisor is responsible for supervising timely and accurate data collection by staff. In order to assist the supervisor in his/her efforts with the surveillance forms, the following checklist is provided. The supervisor has the option to train and assign these tasks to other study staff members.

On a daily basis, the supervisor should:

- Be available for questions by field worker/data collection and data entry staff.
- Ensure that data collection staff knows what visits must be made.
- Monitor that the forms are kept confidential and safe in a locked cabinet in the office when they are not being used in the field.
- Be notified of any adverse events that occur in the study.
- Report all Adverse Events related to the study to the site-PI within 24 hours of notification of these events.

On a weekly basis, the supervisor should:

- Plan out weekly schedules and responsibilities for field workers (using database scheduler or local equivalent) and communicate expectations to field workers.
- Review 100% of data collection forms filled out in the past week (prefer daily, but should be within 7 days) to see that:
  - All forms are filled out completely and correctly. Supervisor/designee initials and dates each reviewed form.
  - All scheduled visits were made by study staff. Each site is responsible for monitoring missed visits and enacting corrective action.
  - PDFs were completed for all visits made outside the allowable window.
  - Recruitment targets are being met (goal is 9-18 recruited per month)
  - Participants are being retained in the study (not dropping out/missing)
- Deliver all completed forms to the data entry staff.
- Convene a weekly meeting with all field staff to discuss past issues, future plans, and any updates to forms or procedures. It is preferable that the on-site PI attends these weekly meetings or at least is available for consult as needed.
- Meet with individual field workers to discuss the results of any QC forms that were collected (XAF or ANT).

On a monthly basis, the supervisor should:

- Determine if overall recruitment targets were met for the previous month (9-18 per month) (and if not, troubleshoot why not) and make plans to meet recruitment targets for upcoming month.
- Determine how well data collectors performed in the previous month (visits completed within windows), and make schedule for field worker visits during the upcoming month.
- Determine if quality control visits planned for the previous month were made, and schedule quality control visits (ANT (5%) and XAF (10%)) for the upcoming month.
- Review DCC quality control report (and perform any local QC checks)
- Meet with site PI to discuss progress (enrollment, participant retention rates), review QC reports, and troubleshoot problems.
- Check if any study participants are missing the maximum of 60 consecutive days of data collection.
- Share “lessons learned” for issues that have come up in the field or in lab with other site’s staff and MALED admin.

On a quarterly basis (or more often if needed), the supervisor should:

- Hold a formal training session on a locally relevant topic to increase quality and/or timeliness of surveillance data collected (can be refresher training or an outside expert in retention methods, for example).

VIF—Vaccine Information Form SOP

I. Purpose

To collect information regarding childhood vaccinations source for each study participant. Information on the names of the vaccines, the dates of administration, and the sources of the information will be collected. This will be used as a validation tool for source of vaccine questions adminstred in the MOA form.

II. Material

Vaccine Information Form (VIF), Pen

III. Methods

The Vaccine Information Form (VIF) is to be used at months 3, 6, 9, 12, 24,27, 30 33 and 36 (or the end of the study). It will be used concurrently with the Monthly Form A (MOA) at months 3 and 6, with the Monthly Form B (MOB) at months 9, 12 and 24, and with the Monthly Form C (MOC) at month 27, 30, 33 and 36 (or the end of the study).

As with the Monthly Forms A, B and C, FWs should ideally visit a study participant on the monthly anniversary of his/her birthday. Sometimes this may not be possible. However, it is important the forms be completed within the given +/- two day window. If data collection occurs outside the +/- 2 day window, a PDF form must be completed. Please see the MOA SOP for more detailed information regarding the window of data collection.

IV. Further Instructions

Record information regarding the *Vaccine Name*, *Date of Administration*, and *Source* of information, for each vaccine received by the study participant since the last VIF was filled.

Vaccination information from children’s vaccination cards or clinic records is STRONGLY preferred.

When visiting the study participant’s household, ask to see vaccine records. If vaccine records are available, check to see if any vaccines have been given since the last VIF was completed. If vaccines have been given since the last VIF was filled, document them here. In the *Vaccine Name* column, write down the name of the vaccine exactly as it appears on the vaccination card (e.g. if the child received “Pentaxim”, write “Pentaxim” in *Vaccine Name* column). In the *Date of Administration* column, write down the date the study participant received the corresponding vaccine exactly as it appears on the vaccination card. For the *Source* column, write “1”, since this information was obtained from the study participant’s vaccination history card.

If vaccination history records for the study participant are unavailable, obtain information regarding childhood vaccinations from clinic records. As above, enter information on the *Vaccine Name* and *Date of Administration* exactly as they appears on the clinic record. For the *Source* column, write “2”, since this information was obtained from the study participant’s clinic records.

If the study participant lacks both vaccination history records and clinic records, ask the mother, caregiver or other guardian for a verbal history of the child’s vaccinations and the approximate dates that the vaccines were administered. Record this information in the *Vaccine Name* and *Date of Administration* columns. For *Source*, write “3”, since this information was obtained from a verbal source.

If information on a study participant’s vaccination history was obtained by some other means, record this information as above, and in the *Source* column, write “4”, for other.

To summarize, options for the *Source* column are:

1. From vaccination card
2. From clinic record
3. From verbal source (e.g. mother, guardian etc.)
4. Other

If information on vaccines given to the study participant comes from a variety of different sources, mark the source of information regarding each vaccine in the corresponding *Source* column appropriately.

If no vaccines have been given during the period covered by the VIF, fill out the participant ID, date, and fieldworker ID, and leave the rest blank. If this is not done, the form will be consider ‘missed’.

**Collection and Processing of Clinical SampleS**

SIL—Sample ID Log SOP

I. Purpose

To assign unique Sample IDs (SIDs) to all biological specimens collected.

II. Material

Sample ID logbook, pen

III. Methods

1. After a biological specimen (e.g. child or maternal stool, blood, urine, saliva, breastmilk) has been received in the laboratory, the laboratory technician will immediately assign a unique Sample ID to this biological specimen.
2. The laboratory technician will use the Sample ID logbook provided to each site to assign the Sample ID to this specimen. The laboratory technician will enter information on PID, specimen type (B, U, M, D, S), and date (DD/MMM/YY) and time of collection (HH:MM; 24 hour time scale) adjacent to the first blank entry next to the pre-printed column of Sample IDs.
3. The SID will be hand-written by the laboratory technician on the labels for all of the specimen collection containers for a specific study subject on a certain date and time of specimen collection.
4. The unique SID attached to the specimen will remain with the specimen and its aliquots throughout the testing and evaluation process.
5. The SID log is the link between the PID, SID, and time and date of collection.
6. The SID log information will be stored locally and will be entered to the central MAL-ED database on a weekly basis.

IV. Notes

- What is the SID?

Digits 1 & 2 – Indicate country (PE-Peru, BR-Brazil, Bangladesh-BG, Nepal-NP, Pakistan-PK, India-IN, Tanzania-TZ, South Africa-SA)

Digit 3 – Indicates site # (most countries have only one study site at this time)

Digit 4-9 – Indicates the ascension number for the specimen

- The SID will be used to identify biological specimens on all other study forms.

**Collection and Processing of Clinical Samples**

**Stool**

SCT/SFC—Stool Collection, Processing and Transport to the Laboratory

**This SOP has been read and understood by:**

| **Name** | **Date** |
| --- | --- |
|  |  | |
|  |  | |
|  |  | |
|  |  | |
|  |  | |
|  |  | |
|  |  | |
|  |  | |
|  |  | |
|  |  | |

**Document History:**

| **Version Number** | **Reason for Changes** | **Date** |
| --- | --- | --- |
|  |  |  |
|  |  |  |
|  |  |  |
|  |  |  |

1.0 **Purpose**

This protocol describes the procedure for stool specimen collection from MAL-ED research subjects enrolled in the cohort and timely transport to the laboratory for further processing.

2.0 **Responsibility**

Field coordinator is responsible for ensuring that standard methodology described here is followed in stool collection and transport.

3.0 **Materials**

3.1 Stool specimen (4 – 10 grams or ml)

3.2 Pre-printed PID labels (4 plus 1 extra)

3.3 Cary-Blair media transport tube

3.4 Plastic disposable transfer pipette for liquid stools

3.5 Cotton-tipped wooden stick

3.6 Wide-mouthed plastic container suitable for collecting stools

3.7 Wooden spatula

3.8 Vial with 10 ml of 10% buffered formalin

3.9 Frozen ice packs

3.10 Cold box

3.10 Tube rack

3.11 Disposable latex gloves

3.12 Disposable diaper

3.13 Sealable plastic bags

3.14 Plastic spoon

3.15 Pen

3.16 Surveillance Assessment Form (SAF) or Monthly Combined Form A (MOA) or Monthly Combined Form B (MOB)

3.17 Stool Field Collection Form (SFC)

4.0 **Collection Procedure for Monthly Stool**

4.1 Monthly stool sample will be collected on the same day as the scheduled day for completion of the monthly surveillance form. This visit shall occur on the monthly anniversary of the child’s birth (+/- 2 days). So, if a child is born on February 17^th^,for example, the monthly visits will occur every month from the 15^th^ to the 19^th^ of that month. It is very important that the surveillance form and the stool sample are completed/collected within the +/- two day window. If you are unable, even with the best of efforts, to visit the child within the 5 day window (birth date anniversary +/- 2 days), the field worker may visit the home up to 5 days beyond the +2 timeframe (the birth date anniversary + 7 days). However, if the visit takes place beyond 2 days after the birth date anniversary, a PDF must be filled out.  **Do not collect monthly stool samples within 48 hours after the administration of the Lactulose Mannitol solution because LM interferes with some of the gut function biomarker assays done with stool samples.**

4.2 Monthly stool samples will be collected every month for two years (24 months) as described above. Samples collected on month 1-12, 15, 18, 21, and 24 will be worked up for the detection of enteric pathogens as described in subsequent protocols in this document. Samples collected on months 13, 14, 16, 17, 19, 20, 22, 23, (and if extension funded, 27, 30, 33 and 36) will be frozen at -80C as archival samples. Samples collected on months 13, 14, 16, 17, 19, 20, 22, 23, (and if extension funded, 27, 30, 33 and 36) do NOT require recollection if greater than 2 grams but less than 4 grams. If samples collected on months 13, 14, 16, 17, 19, 20, 22, 23, (and if extension funded, 27, 30, 33 and 36) are less than 2 grams, recollection should be requested. Additionally, we encourage sites to obtain a stool sample as soon as possible after enrollment for freezing in the archive. This “0” time sample will not be characterized for pathogens, but will be valuable for future studies to determine the earliest gut microbiome in children enrolled in this cohort. The time “0” stool does NOT require recollection.

If the MAL-ED extension project is funded, sites should collect at a minimum quarterly (at 27, 30, 33, 36 months) stool samples for archiving only. These quarterly samples should be collected on the anniversary of the child’s birth (+/- 2 days). If you are unable, even with the best of efforts, to visit the child within the 5 day window (birth date anniversary +/- 2 days), the field worker may visit the home AT ANYTIME to collect the stool sample. However, if the visit takes place beyond 2 days after the birth date anniversary, a PDF must be filled out. Sites may choose to collect stools more frequently and/or work up these samples for some or all of the microbiology assays however these activities will not be covered by MAL-ED funds.

4.3 For collection of Monthly Stool, inform child’s primary caretaker one day before planned stool collection and request caretaker to collect the first available fresh stool sample from the child on the morning of the planned visit.

4.4 The mother should be provided with the labeled stool container, diaper for infants, cold box, ice packs, gloves, plastic spoon, and 2 plastic bags the evening before planned stool collection. There should be enough ice packs in the cold box to keep it cold for up to 16 hours.

4.5 Instruct the caretaker to use the plastic spoon to collect 3-4 spoons of stool and place it in the stool container, close the lid tightly, and place the container in the plastic bag. If the child is also enrolled in the BMMI project, collect all of the stool sample.

5.0 **Collection Procedure for Diarrheal Stool**

Collection of diarrheal stool poses a challenge to the Study Researcher / Nurse / Fieldworker. We recommend that sites develop collection methods that are consistent with their local situation. We suggest some general methodology here for consideration. Remember that the definition of diarrhea is 3 loose stools in a single day. When that definition is met, every attempt should be made to visit the home as soon as possible to collect a sample before the episode ends. Caregivers should be instructed about the urgency of collection of a fresh sample and getting that sample into transport medium as soon as possible to increase the likelihood of identifying causative bacterial pathogens.

5.1 Caretakers of infants with liquid stools should be instructed to use an inverted diaper so that the stool doesn’t get absorbed in the lining.

5.2 We suggest leaving extra stool containers (without Cary Blair transport media) with the family with instructions to collect stool samples when a child has diarrhea. If the first sample is watery and the definition of diarrhea is not met, then the sample can be discarded. Sometimes, when an episode is of short duration, it does happen that the mother fails to collect any of the 3 or more stools, in which case that episode will have no sample associated with it. It should still be recorded as a diarrheal episode, but with no sample collected.

5.3 When a Study Researcher / Nurse / Fieldworker arrives at the home and a sample has already been collected by the caregiver, ask the caregiver what time the sample was produced and record that time (on SFC, question #4). In most cases this will only be an estimate. However it may be useful to estimate the time prior to storage in Cary Blair transport medium. Every attempt should be made to get the sample into Cary Blair within 2 hours of production. The time that the sample was preserved in Cary Blair should recorded on the SFC, question #5. The time that the Study Researcher / Nurse / Fieldworker arrives at the home and collects the specimen should also be recorded on the stool field collection form (SFC) (question #2, Time of visit).

5.4 If a diarrheal stool sample is not available, the Study Researcher / Nurse / Fieldworker may be able to wait at the home for a sample to be produced. The Study Researcher / Nurse / Fieldworker should assess the likelihood that they can collect a sample while waiting in light of other visits they need to make that day. If they cannot wait, they should leave collection material (specimen receptacle and cold box with ice packs) with the caregiver if they have not done so previously and provide instructions on collection procedure and plan to return to the home every 2 hours until a sample is collected. The arrival time of the visit should be documented on the SFC, question #2.

5.5 As soon as it is available to the Study Researcher / Nurse / Fieldworker, a portion of the diarrheal stool sample should be placed in Cary Blair (within 2 hours of production if possible) and refrigerated in cold packs for transport to the laboratory and processing (see below). Follow the temporary storage and transport procedures listed directly below.

6.0 **Temporary Storage and Transport Procedures**

6.1 Instruct the caretaker to place the plastic bag with the stool in the cold box immediately after collection (maximum time: 1 hour).

6.2 Collect the stool specimen early in the morning (maximum time from placement of stool in cold box to collection: 5 hours).

6.3 Document if the sample was in a cold environment on the requisition CRF. Also document if specimen is acceptable (estimated quantity, lid closed, and no leakage)

7.0 **Processing**

7.1 Label the original stool container, the Cary Blair tube and formalin vial with pre-printed PID labels. Circle the type of specimen received on the label (M=monthly stool collection, D=diarrheal stool recollection), and write the date of collection (DD/MMM/YY) and time of collection (hh:mm; 24 hour time scale) on the label.

7.2. Immediately upon collecting the stool sample, the Study Researcher / Nurse / Fieldworker must dip two cotton-tipped wooden sticks in the fecal specimen and place in Cary Blair transport media, breaking off the tip. This is the specimen that will be used for bacteriological culture.

7.3. Place 1 gram of stool using the wooden spatula, or 1 ml of liquid stool using the transfer pipette in 10% formalin vial. The preserved stool will be used for microscopic examination for ova and parasites. The preferred Formalin to stool ratio is 3:1.

7.4 Place the original stool container with the remaining stool, and the stool in formalin vial in a fresh plastic bag, and the Cary Blair tube in the tube rack, and transport in the cold box with fresh ice packs to the laboratory for further processing.

7.5 Maximum time from placement of stool swab in Cary Blair to receipt of all the specimens in the laboratory should be 18 hours.

1. **Reporting**

8.1 Record information on Stool Field Collection Form (SFC). The SFC form is completed for every diarrheal episode, each recollection attempt, and for each time the fieldworker travels to the home to complete the monthly surveillance form and to collect the monthly stool sample. Please see the table below for assistance. There is space provided at the end of the form to provide additional information, if necessary.

| # | Question | Guidance |
| --- | --- | --- |
|  | Participant ID | Write the child’s participant ID in the space provided at the upper left corner. |
| 01 | Date of visit | Enter the date of the visit (DD/MMM/YY) by the Study Researcher / Nurse / Fieldworker. |
| 02 | Time of visit | Enter the time that the Study Researcher / Nurse / Fieldworker visited the home to collect and pick-up the stool sample. If no sample was collected, this is the arrival time of the visit. |
| 03 | Was a stool sample collected? | Indicate whether or not a stool sample was collected. If yes, go to question #4. If no, go to question #8. |
| 04 | Time stool sample was produced | Enter the time (24 hour time scale, HH:MM) that the stool sample was produced. If this time is not available, estimate if time cannot be confirmed by care giver. |
| 05 | Time stool sample was preserved in Cary Blair | Enter the time that the stool sample was preserved in Cary Blair. |
| 06 | Type of stool collected | Indicate whether the stool sample was a monthly, diarrheal, monthly recollection, or diarrheal recollection. If the stool sample is a time zero stool sample, record as a monthly stool sample (M1). All initial collections of monthly stools are recorded as M1. There is no sequential numbering for the number of monthly stools collected. |
| 07 | Month of stool collected | Enter the number (#) of months that the child has been followed and in which this monthly stool sample was collected for this particular child. For example, if a child has been followed for 14 months and the stool is being collected for his 14^th^ monthly stool sample, enter 14. If the stool sample is a diarrheal stool sample, enter NA. |
| 08 | Recollection necessary? | Indicate if recollection is necessary. Every effort must be made to collect 4 to 10 grams of stool- the quantity stated in the protocol. It is recognized that some specimens received in the laboratory will be QNS- (Quantity Not Sufficient) -less than 4 grams, and require a recollection attempt. Recollection of the specimen may ONLY continue for up to 48 hours. If the stool sample is a time zero stool sample, no recollection is necessary, and the response should be NA. Samples collected on months 13, 14, 16, 17, 19, 20, 22, 23, 27, 30, 33, and 36 do NOT require recollection if greater than 2 grams but less than 4 grams. If samples collected on months 13, 14, 16, 17, 19, 20, 22, 23, 27, 30, 33, and 36 are less than 2 grams, recollection should be requested. |
| 09 | Fieldworker ID | Enter the fieldworker’s unique ID number who picked up the stool sample and/or visited the home. |

### RSS/SRF—Receiving and Storage of Stool Samples SOP

**This SOP has been read and understood by:**

| **Name** | **Date** |
| --- | --- |
|  |  | |
|  |  | |
|  |  | |
|  |  | |
|  |  | |
|  |  | |
|  |  | |
|  |  | |
|  |  | |
|  |  | |

**Document History:**

| **Version Number** | **Reason for Changes** | **Date** |
| --- | --- | --- |
|  |  |  |
|  |  |  |
|  |  |  |
|  |  |  |

1. Purpose

This document describes the procedures for receiving stool samples collected from study subjects, and the assignment of Sample ID to the stool samples. The stool specimens are collected for assessment of enteric infections and gut function. The procedures are designed to maintain the integrity of clinical specimens by proper handling and storage and the management of associated records. Personnel handling the samples and data are trained to receive and handle clinical specimens and records.

1. Materials

Refrigerator

-70°C freezer

-20°C freezer

Wooden spatula

Sterile cryovials

Cryovial labels

Cryovial racks

Storage boxes

Pipettes (20-200 µl and 100-1000 µl)

Scale

Stool receiving form (SRF)

1. Methods

**Safety:**

1. Universal precautions must be followed while handling samples (http://www.cdc.gov/ncidod/dhqp/gl_isolation_standard.html).
2. Technicians must wear appropriate proper personal protective equipment (PPE) (e.g. gloves, masks, etc.) when handling samples as well as when handling ultra cold freezer items.
3. Disinfect work areas before and after performing these procedures.
4. Avoid using techniques that are likely to generate aerosols.

Additional information can be found at <http://www.cdc.gov/OD/ohs/biosfty/bmbl5/bmbl5toc.htm>.

**Transfer of samples to the laboratory:**

Proper transfer of samples from field to laboratory will require coordination between field workers and laboratory technicians. Collection and transport of samples should be carried out in clean, labeled, dry, leak-proof containers. Transport the samples to the laboratory as soon as possible after collection. Samples should be transported in an ice chest or insulated carrier containing adequate amount of cold packs as well as cushioning material to protect the samples. Do not use wet ice for transport of clinical specimens. There will be three sets of stool samples for each individual. Each of the sets will be transferred from the field to the laboratory with pre-printed PID labels, and information regarding time and date of stool collection and type of stool collected (monthly or diarrheal stool).

***Three*** sets of stool samples will be received from each study subject –

1. Whole stool sample or unpreserved stool sample (UP)
2. Stool in Cary-Blair medium (CB)
3. Stool in 10% formol-saline (FS)

Samples in CB will be used for bacterial culture while samples in FS will be used for microscopic examination for ova and parasites. These samples have to be processed as soon as received in the laboratory. The unpreserved sample will be used for gut function assessment, viral and parasite ELISAs, RNA / DNA extractions and PCRs. These samples can be batched and stored at -70°C before testing. If the child is also enrolled in the BMMI project, an additional aliquot/s of unpreserved stool sample (total of 2 grams) should also be frozen and stored for later shipment.

**Receipt of specimens:**

When the stool sample is received at the laboratory, the Sample ID is immediately assigned using the Sample ID logbook provided to each individual site. Please see Sample ID log (SIL) SOP for detailed instructions. After the Sample ID has been successfully assigned, the Sample ID is written on the existing label on all sets of stool samples made from this stool collection. All stool sample sets collected at that specific time will have the SAME Sample ID for each individual study subject. Any future aliquots of this stool sample collected at that specific time will also have the same Sample ID. Future stool samples collected a different time from the same individual will have different Sample IDs. Recollection samples have a different Sample ID than the initial sample’s Sample ID.

**Reporting:**

Record information on Stool Receiving Form (SRF). Please see the table below for assistance. Information for questions #02, 03, 04, 05, 09, 10, 11, can be found on the Stool Field Collection (SFC) form completed by the fieldworker.

| # | Question | Guidance |
| --- | --- | --- |
|  | Participant ID | Write the child’s participant ID in the space provided at the upper left corner. |
| 01 | Sample ID | Write the 9-digit unique Sample ID. |
| 02 | Date of collection | Enter the date of collection (DD/MMM/YY) of the stool sample. Information located on SFC, question #1. |
| 03 | Time stool specimen was produced | Enter the time that the stool specimen was produced. If this time is not available, estimate if time cannot be confirmed by care giver. Information located on SFC, question #4. |
| 04 | Time stool specimen was picked up by Study Researcher / Nurse / Fieldworker | Enter the time that the stool specimen was picked up by the Study Researcher / Nurse / Fieldworker. Information located on SFC, question #2, time of visit. |
| 05 | Time stool specimen was preserved in Cary Blair | Enter the time that the stool specimen was preserved in Cary Blair. Information located on SFC, question #5. |
| 06 | Time stool specimen was received at field site/ laboratory | Enter the time that the stool specimen was received at the main laboratory or the intermediate field site, if appropriate. |
| 07 | Technician code | Enter the technician’s unique ID number who processed the specimen. |
| 08 | Unpreserved specimen? | Indicate whether or not an unpreserved specimen was received from the field. If no unpreserved specimen was received, complete QNS form and go to question #9. If yes, go to question #9. |
| 09 | Type of specimen | Indicate whether the stool specimen was a monthly, diarrheal, monthly recollection, and diarrheal recollection. Information located on SFC, question #6 and on sample container. |
| 10 | Month of stool collected | Enter the number (#) of months that the child has been followed and in which this monthly stool sample was collected for this particular child. For example, if a child has been followed for 14 months and the stool is being collected for his 14^th^ monthly stool sample, enter 14. If the stool sample is a diarrheal stool sample, enter NA. |
| 11 | If recollection, what is Sample ID of initial sample? | If the stool sample is a recollection sample, enter the Sample ID of the initial sample. If the stool sample is not a recollection sample, enter NA. **Recollection samples have a different Sample ID than the initial sample’s Sample ID.** All sample IDs are unique values. |
| 12 | Amount of stool | Enter the number of grams of stool received. |
| 13 | Recollection? | Indicate if recollection was requested, and what type of recollection (monthly vs. diarrheal) is requested. Information located on SFC, question #8. If the stool sample is a time zero stool sample, no recollection is necessary, and the response should be NA. Recollection of the specimen may ONLY continue for up to 48 hours. If samples collected on months 13, 14, 16, 17, 19, 20, 22, 23, 27, 30, 33, and 36 are less than 2 grams, recollection should be requested. |
| 14 | Consistency | Indicate the consistency of the stool sample, WATERY (very loose, watery stool with very little solid material) = 01, LIQUID (loose stool taking the shape of the container) = 02, SOFT (semi-solid, smooth stool) = 03, FORMED (solid, firm stool) = 04 |
| 15 | Bloody? | Indicate if the stool specimen was bloody or not bloody. **If bloody, refer the child for medical treatment of suspected dysentery.** |
| 16 | Mucus? | Indicate if the stool specimen had mucus or did not have mucus. |
| 17 | Was Cary Blair used in the field? | Indicate yes or no if Cary Blair was used in the field. If yes, go to question #19. If no, go to question #18. |
| 18 | Time Cary Blair used in the laboratory | Indicate what time Cary Blair was used in the laboratory. |
| 19 | Was formalin used in the field? | Indicate yes or no if formalin was used in the field. If yes, go to question #21. If no, go to question #20. |
| 20 | Time formalin used in the laboratory. | Indicate what time formalin was used in the laboratory. |
| 21 | Time stool left field site laboratory | For SELECT SITES ONLY, indicate what time the stool sample left field site laboratory. If your site does NOT have an intermediate field site processing laboratory, please respond NA. |
| 22 | Time stool received at main testing laboratory | For SELECT SITES ONLY, indicate what time the stool sample was received at the main testing laboratory. If your site does not have an intermediate field site processing laboratory, please respond NA. |
| 23 | QNS set up? | Indicate yes or no if this stool specimen requires a QNS setup. If there is less than 4 grams, the stool specimen requires a QNS setup. If yes, complete the QNS form and refer to SOP number QNS “Processing and Testing of QNS Stool Samples” for detailed instructions on how to proceed. If the stool sample is a time zero stool sample, no QNS set-up is necessary, and the response should be NA. If samples collected on months 13, 14, 16, 17, 19, 20, 22, 23, 27, 30, 33, and 36 for archiving purposes are less than 2 grams, these samples require a QNS set-up. A recollection sample does not need to be worked up as a QNS sample. If sample is a recollection sample, enter NA. |

**Internal Log-in of Specimens:**

All specimens must be held at 2 – 8°C until ready to process.

We recommend that sites develop tracking methods for sample testing, short-term sample storing, and general laboratory procedures that are consistent with their local situation. We suggest some general methodology here for consideration. Determine that all specimen information is available on the laboratory’s specimen receipt form (including PID and sample ID). Each laboratory will responsible for creating a logbook to track the specimens internally. The MAL-ED storage database can be used to assist in storing and tracking of samples.

*A sample stool specimen information form is given below.*

*
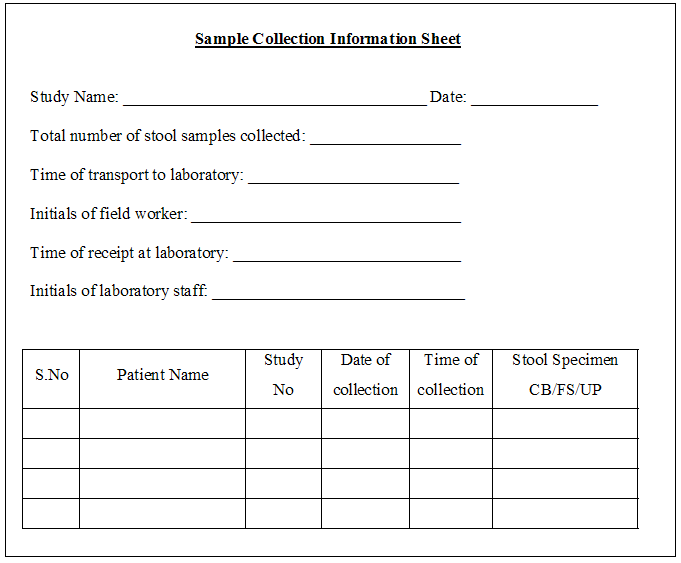
*

The details on the specimen form must agree with the information recorded on the specimen label (include PID and Sample ID on labels).

*An example of a stool specimen label is given below.*


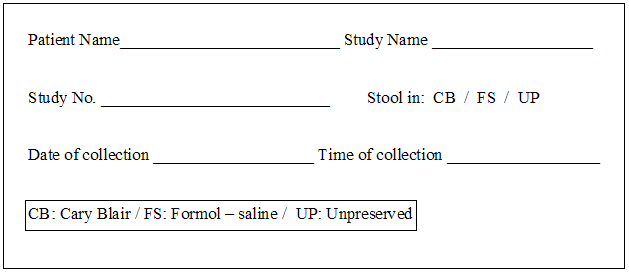


Log in the specimen details (including PID and sample ID) in the designated database or spreadsheet as well as any internal laboratory logbooks for the study. **Recollection samples have a different Sample ID than the initial sample’s Sample ID.**

There should be individual spreadsheets, log sheets and/or laboratory log books for each type of sample (blood, urine, stool, saliva) in the study.

Ensure that all details are entered including participant ID, sample ID, type of sample, volume of sample collected and date and time of sample collection. Enter the initials of the person handling the specimen in the appropriate column.

Approximately 4 – 10 g or ml of stool is collected from each study subject. When Cary Blair and Formalin are received, there should be a minimum of 2.5 grams unpreserved specimen available for ELISAs, PCR and archive. If there is less than 4 grams, refer to SOP number QNS “Processing and Testing of QNS Stool Samples” for detailed instructions on how to proceed. For a monthly stool collection, recollection should be requested if there is less than 4 grams of stool, and QNS should be followed. For a diarrhea stool sample, recollection should be requested is there is less than 2 grams of stool, and QNS should be followed. For a diarrhea stool sample between 2-4 grams, no recollection is necessary but QNS should be followed, If samples collected on months 13, 14, 16, 17, 19, 20, 22, and 23 for archiving purposes are less than 2 grams, recollection should be requested, and QNS form should be completed. Determine if recollection was requested and/or received. Lab technicians need to communicate to field workers as soon as possible to indicate if recollection is required. Methods of communication may include telephone, email, text message, personal communication. It is essential that field workers are aware that recollection has been requested. If the stool sample is a time zero stool sample, no recollection or QNS set-up are necessary.

*A sample laboratory log book and MS-Excel worksheet are given below:*
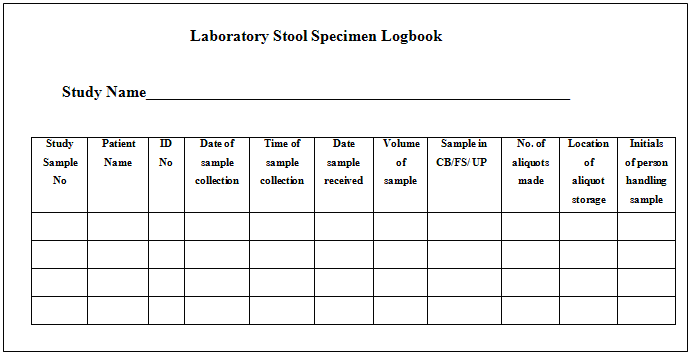


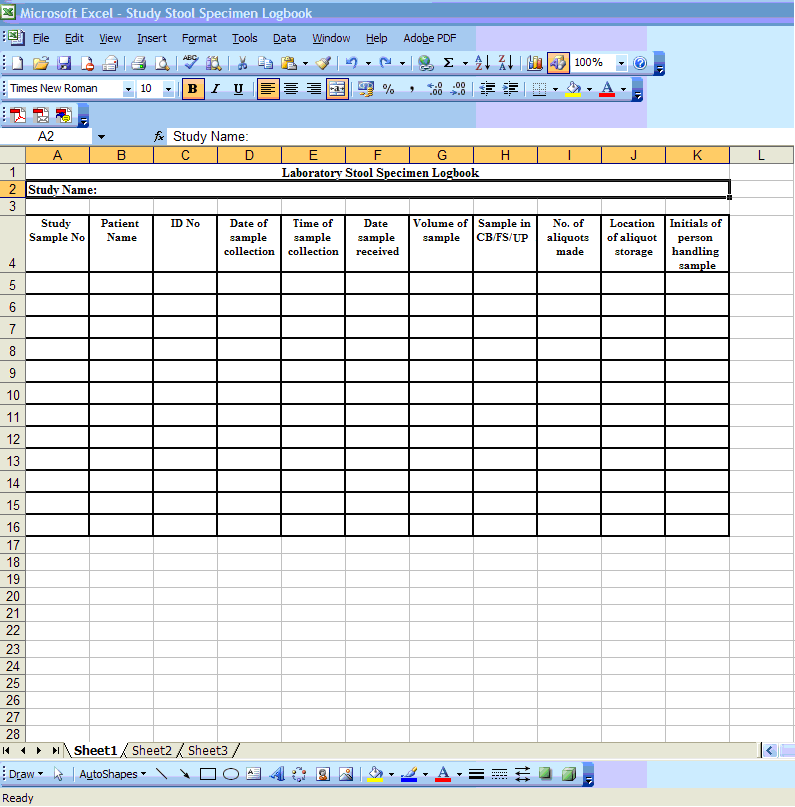


**Procedure for Aliquoting and Storage:**

1. For each sample, 4 aliquots will be made, depending on the volume of original sample available. 4 vials with 0.5 grams each vial is the standard protocol. If less is available for archive, refer to SOP QNS “Processing and Testing of QNS Stool Samples” for detailed instructions. If the stool sample is a time zero stool sample, no QNS set-up is necessary. Samples collected on months 13, 14, 16, 17, 19, 20, 22, 23, 27, 30, 33, and 36 do NOT require a QNS set-up if less than 4 grams but greater than 2 grams. Samples collected on months 13, 14, 16, 17, 19, 20, 22, 23, 27, 30, 33, and 36 DO require a QNS set-up if less than 2 grams.
2. Prepare 4 barcode labels per sample. Place each of these labels on individual cryo vials. Refer to SOP “Barcode Sample Tracking” instructions for assistance in creating label for archiving/storage.
3. Sample aliquots are done on ice.
4. Store aliquots at -70˚C.
5. Log-in the information of where the aliquots are stored with details of freezer number and rack both in the barcode database for sample storage as well as in the laboratory specimen storage logbook.
6. Bacterial isolates will also be frozen and stored at -70˚C. Refer to the Stool Bacteriology Culture and Identification: BDF SOP for additional information as well as the SOP for Barcode Sample Tracking.
7. If the child is also enrolled in the BMMI project, an additional 2 grams of unpreserved stool should also be frozen. These 2 grams can be left in one tube or placed into aliquots; this decision is at the discretion of the site. This sample will be later shipped to the BMMI project.

Stool Specimen Test Quantities and Storage

| **Test** | **Specimen or Derivative** | **Qty** | **4 ^o^C**  **storage** | **-20 ^o^C storage** | **-70 ^o^C storage** | **Room Temp storage** | **Storage after initial processing** | **QNS Alternate Specimen** |
| --- | --- | --- | --- | --- | --- | --- | --- | --- |
| Primary Specimen | Stool | 4-10 grams | 2 hours | NA | NA | 2 hours | NA | None |
| O&P microscopy (inclusive concentrate Microscopy & M. acid fast ) | Formalin | 1 gram | NA | NA | NA | Long term-indefinitely | after concentration RT Long term /indefinitely | Unpreserved -direct microscopy |
| Bacteriology Culture | Cary-Blair | 0.2g | 18-48 hours | NA | NA | 2 hours | NA | Unpreserved 4 ^o^C  4 hours |
| Campy ELISA | Unpreserved | 0.1 g or 100 ul | 72 hours | Long term | Long term | NA | In kit diluent 4^o^C  72 hours | Formalin  (not concentrated) |
| Viral ELISA (inclusive Adeno, Astro Rota) | Unpreserved | 0.1 g or  100 ul | 8 days | Long term | Long term | NA | In kit diluent 4^o^C  8 days | Adeno only-Formalin (not concentrated) |
| Giardia & Crypto ELISA | Unpreserved | 0.1g or  100 ul | 24 hours | Long term | Long term | NA | NA | Formalin  (not concentrated) |
| E.hist ELISA | Unpreserved | 0.2g or  200 ul | 48 hours | 2 months- Long term | 2 months-Long term | NA | NA | None |
| Norovirus RT PCR | Unpreserved | 0.1 g or 100 ul | 48 hours | NA | NA | Long term | Extracted RNA  4 ^o^C 4 hours,  -70 ^o^C long term | None |
| Archive | Unpreserved | 2 g  (4 x0.5 g) | NA | NA | Long term | NA | NA | None |
| E.coli PCR | Colonies from Culture | 5 colonies | NA | NA | Long term | Slant 2 months | Not Specified | None |
| Lactoferrin Techlab IBD Check | Unpreserved | 50 ul | 2 weeks | Long term | Long term | 2 weeks | In kit diluent 4^o^C  48 hours | None |
| a1 antitrypsin?? | Unpreserved | 0.1 g | ? | ? | ? | ? | ? | ? |

QNS—Processing and Testing Quantity Not Sufficient Stool

**This SOP has been read and understood by:**

| **Name** | **Date** |
| --- | --- |
|  |  | |
|  |  | |
|  |  | |
|  |  | |
|  |  | |
|  |  | |
|  |  | |
|  |  | |
|  |  | |
|  |  | |

**Document History:**

| **Version Number** | **Reason for Changes** | **Date** |
| --- | --- | --- |
|  |  |  |
|  |  |  |
|  |  |  |
|  |  |  |

1. Purpose

A complete set of laboratory test results on every stool specimen is of critical importance in the analysis of MAL-ED data. Every effort must be made to collect 4 to 10 grams of stool- the quantity stated in the protocol. It is recognized that some specimens received in the laboratory will be QNS- (Quantity Not Sufficient) -less than 4 grams. The purpose of this procedure is to describe the processing and list the prioritization of tests when the laboratory receives less than 4 grams of stool specimen. Samples collected on months 13, 14, 16, 17, 19, 20, 22, 23, 27, 30, 33, and 36 for archiving purposes do NOT require a QNS set-up if less than 4 grams but greater than 2 grams.

1. Materials

No materials necessary.

1. Methods

Refer to SOP SSC, “Stool Collection and Transport,” and SOP RSS, “Receipt and Storage of Stool Samples in the Laboratory”. Specimen Quantities covered in this procedure: Standard testing 4-10 g.; QNS Monthly: <4 g.; QNS Diarrheal: <2 g.; Diarrheal 2g to <4g.

1. Notify Field Worker and Field Supervisor of all QNS Specimens and request recollection. Requests should be made by the lab technician to the appropriate field worker as soon as possible. Methods of communication to convey that recollection is necessary may include telephone, email, and/or text message. Document request on the Stool Receiving Form (SRF) and complete QNS form.

2. Proceed with specimen processing based upon the quantity and type of specimen received.

**4-10 grams** – **Follow Standard Testing Procedures**

| *Standard Procedure Testing* | *Specimen* | *Qty* |
| --- | --- | --- |
| Primary Specimen | Stool | 4-10 grams |
| O&P microscopy (concentrate Microscopy & Mod AF ) | Formalin | 1g |
| Bacteriology Culture | Cary-Blair | 0.2g |
| Campy ELISA | Unpreserved | 0.1g or 100ul |
| Viral ELISA (inclusive Adeno, Astro, and Rotavirus) | Unpreserved | 0.1g or 100ul |
| Giardia and Crypto ELISA | Unpreserved | 0.1g or 100ul |
| E. hist ELISA | Unpreserved | 0.2g or 400ul |
| Norovirus RT-PCR | Unpreserved | 0.1g or 100ul |
| Archive sample | Unpreserved | 2g (4 x 0.5 g) |
| Lactoferrin | Unpreserved | 50 ul |
| Alpha-1- antitrypsin | Unpreserved | 0.1g |

**Monthly Specimen Less Than 4 grams**

1. Request Recollection

2. Proceed with testing in the order below.

3. As additional specimen is received, continue testing- picking up where the previous specimen left off. Complete as many tests as the quantity will allow.

4. Continue testing the recollected specimens for up to 48 hours. Recollection samples have a different Sample ID than the initial sample’s Sample ID.

5. After 48 hours from when the first monthly specimen was collected, stop testing for that monthly specimen.

6. Complete the QNS Form.

| *“Monthly” Specimen QNS Testing* | *Specimen* | *Qty* |
| --- | --- | --- |
| Primary Specimen | Stool | Less the 4g |
| Bacteriology Culture | Cary-Blair | 0.2g |
| Campy ELISA | Unpreserved | 0.1g or 100ul |
| Viral ELISA (inclusive Adeno, Astro, and Rotavirus) | Unpreserved | 0.1g or 100ul |
| Norovirus RT-PCR | Unpreserved | 0.1g or 100ul |
| Giardia and Crypto ELISA | Unpreserved | 0.1g or 100ul |
| E. hist ELISA | Unpreserved | 0.2g or 400ul |
| Archive sample | Unpreserved | 2 g (4 x 0.5 g) |
| O&P microscopy (concentrate Microscopy & Mod AF ) | Formalin | 1g |
| Lactoferrin | Unpreserved | 50 ul |
| Alpha-1-antitrypsin | Unpreserved | 0.1g |

**Diarrheal Specimen Less Than 2 grams**

1. Request Recollection

2. Proceed with testing in the order below.

3. As additional specimen is received, continue testing- picking up where the previous specimen left off. Complete as many tests as the quantity will allow. Recollection samples have a different Sample ID than the initial sample’s Sample ID.

4. Continue testing the recollected specimens for up to 48 hours.

5. 48 hours after the first diarrheal specimen was collected, stop testing for that diarrheal episode and complete the QNS Form.

| *“Diarrheal” Specimen QNS Testing* | *Specimen* | *Qty* |
| --- | --- | --- |
| Primary Specimen | Stool | Less than 2g |
| Bacteriology Culture | Cary-Blair | 0.2g |
| Campy ELISA | Unpreserved | 0.1g or 100ul |
| Viral ELISA (inclusive Adeno, Astro, and Rotavirus) | Unpreserved | 0.1g or 100ul |
| Norovirus RT-PCR | Unpreserved | 0.1g or 100ul |
| Giardia & Crypto ELISA | Unpreserved | 0.1g or 100ul |
| E. hist ELISA | Unpreserved | 0.2g or 400ul |
| Archive sample | Unpreserved | 2g (4 x 0.5 g) |

**Diarrheal Specimen 2g to <4g**

1. Proceed with testing in the order below.

2. Complete the QNS Form.

| *“Diarrheal” Specimen QNS Testing* | *Specimen* | *Qty* |
| --- | --- | --- |
| Primary Specimen | Stool | 2g to <4 g |
| Bacteriology Culture | Cary-Blair | 0.2g |
| Campy ELISA | Unpreserved | 0.1g or 100ul |
| Viral ELISA (inclusive Adeno, Astro, and Rotavirus) | Unpreserved | 0.1g or 100ul |
| Norovirus RT-PCR | Unpreserved | 0.1g or 100ul |
| Giardia and Crypto ELISA | Unpreserved | 0.1g or 100ul |
| E. hist ELISA | Unpreserved | 0.2g or 400ul |
| Archive sample | Unpreserved | 2 g (4 x 0.5 g) |
| Lactoferrin | Unpreserved | 50 ul |
| Alpha-1- antitrypsin | Unpreserved | 0.1g |

**Reporting:**

1. Specimen logs should be reviewed monthly to determine rates and patterns of QNS specimen collection.
2. Record information and results on the Quantity not Sufficient form (QNS).

| # | Question | Guidance |
| --- | --- | --- |
|  | Participant ID | Write the participant ID in the space provided at the upper left corner. |
| 01 | Sample ID | Write the 9-digit Sample ID. |
| 02 | O&P concentrate? | Indicate whether or not ova and parasite concentrates will be performed. |
| 03 | Lactoferrin? | Indicate whether or not a lactoferrin test will be conducted. |
| 04 | E. histolytica ELISA be performed? | Indicate whether or not E. histolytica ELISA tests will be performed. |
| 05 | Cryptosporidium and Giardia ELISAs be performed? | Indicate whether or not Cryptosporidium and Giardia ELISA tests will be performed. |
| 06 | Campylobacter ELISA be performed? | Indicate whether or not Campylobacter ELISA tests will be performed. |
| 07 | Specimen archived? | Indicate whether or not a specimen will be archived. |
| 08 | Alpha-1-antitrypsin? | Indicate whether or not an alpha-1-antitrypsin test will be performed. |
| 09 | Viral tests? | Indicate whether or not viral tests will be conducted. |
| 10 | Norovirus RT-PCR be performed? | Indicate whether or not Norovirus RT-PCR will be performed. |
| 11 | Culture set up? | Indicate whether or not a culture will be set up. |
| 12 | Technician code | Enter the technician’s unique ID number who completed the QNS form. |
| 13 | Date | Enter the date that the QNS form was completed. |

**Safety:**

Specimens should be handled, processed and disposed of using standard guidelines for biohazardous materials. Spills should be immediately disinfected.

Microbiology Stool Aliquoting Flow Chart

Field

Stool Specimen Primary

Field Worker retrieves

Aliquot in the Field by FW Aliquot in the Field by FW

Formalin

“FS” 1g

Unpreserved

“UP” 3g

Transport

Data Entry

Lab

Lab “UP”

Archive 2g

-70^0^ C

Lab “FS”

Concentrate

Lab “UP”

ELISA 0.7g

-70^0^ C

Lab “UP”

Noro 0.1g

4^0^ C

*or may store in smaller aliquots*

Mod AF

O&P

Wet prep

LacF

0.05g

Viral

0.1g

Giard &Cryp

0.1g

Camp

0.1g

E.hist

0.4g

#1

0.5g

#2

0.5g

#3

0.5g

#4

0.5g

ELISA thawed specimen stability & work flow
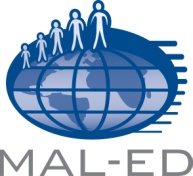


Unpreserved 0.7g stool

for ELISA

-20 to -70 ^0^ C Storage

ELISA workflow is dependent upon the number of stored aliquots designated for ELISAs and specimen stability after thawing.

Repeated freeze-thaw should be avoided.

If only one 0.7g aliquot is stored specifically for ELISAs, then after thawing, testing must occur on Day One through Day 8 (assay dependent).

Storing multiple smaller aliquots for ELISAs permits a more flexible testing schedule but uses more specimen and vials

**DAY 1: Thaw 0.7g stool vial**

**Run assays = “Same Day” ELISA (E hist, Giardia, Crypto)**

Crypto

Test

Same Day

Giardia

Test

Same Day

E.hist

Test

Same Day

Same

Diluent

**DAYs 1-2: “48 hours” ELISA (Lactoferrin)**

Lactoferrin Suspend in

Kit Diluent

Test within

48 hours

**DAYs 1-3: “72 hours” ELISA (Campylobacter)**

Campy

Suspend in

Kit Diluent

Test within

72 hours

**DAYs 1-8: “8 days” ELISA (Adenovirus, Astrovirus, Rotavirus)**

Adeno Suspend in

Kit Diluent

Test within

8 days

Rota Suspend in

Kit Diluent

Test within

8 days

Astro Suspend in

Kit Diluent

Test within

8 days

Same Same

Diluent Diluent

BDF—Stool Bacteriology Culture and Identification: Bacteriology Data Form SOP

**This SOP has been read and understood by:**

| **Name** | **Date** |
| --- | --- |
|  |  | |
|  |  | |
|  |  | |
|  |  | |
|  |  | |
|  |  | |
|  |  | |
|  |  | |
|  |  | |
|  |  | |

**Document History:**

| **Version Number** | **Reason for Changes** | **Date** |
| --- | --- | --- |
|  |  |  |
|  |  |  |
|  |  |  |
|  |  |  |

I. Purpose

To identify *Salmonella* and *Shigella,* *Vibrio, Yersinia, Aeromonas* and *Plesiomonas* in stool specimens.

Stool cultures can detect common causes of bacterial gastroenteritis. Selective and differential media are used to facilitate the isolation of organisms from the complex microbial flora in feces.

Stool specimens will be cultured for *Salmonella* and *Shigella,* *Vibrio, Yersinia, Aeromonas* and *Plesiomonas*. Biochemical tests are used to screen suspicious colonies and identify bacterial species. All presumptive *Salmonella, Shigella* and *Vibrio,* are confirmed by serotyping. *Yersinia, Aeromonas* and *Plesiomonas* will be confirmed by supplemental biochemical tests. The diagnosis of intestinal Campylobacter infection historically relied upon culture using selective media incubated at elevated temperatures in a microaerophilic atmosphere. Studies comparing the ProSpecT Campylobacter microplate antigen assay to standard culture show functional equivalence of the two methods Thus, the identification of *Campylobacter* in stool specimens is achieved by using either the ProSpecT microplate assay or culture; both methods are in Appendix 2.

II. Material

Stored in the refrigerator at 2-8°C:

1. MacConkey agar (MAC)
2. Xylose-Lysine-Deoxycholate (XLD) agar
3. Thiosulphate Citrate Bile Salt Sucrose Agar (TCBS) for Vibrio
4. Campy-BAP
5. Kligler Iron Agar Slants (KIA)
6. Lysine Decarboxylase (LDC) tubes
7. Motility Indole Ornithine Medium (MIO) tubes
8. Urea Slants
9. API 20E identification system or MicroScan Identification System
10. Difco or Denka- Seiken antisera kit

Stored at room temperature:

1. Oxidase reagent
2. Gram’s stain kit
3. Freezer vials

Other supplies and equipment:

1. Inoculating loops/needles
2. Applicator swabs
3. Pasteur pipettes
4. Microscope slides
5. 35 C hot air incubator

III. Methods

**Safety:**

Specimens should be handled, processed and disposed of using standard guidelines for biohazardous materials. Spills should be immediately disinfected.

**Quality control:**

QC requirements vary by test, from once per lot to weekly to each day of use. Refer to “Quality Control Requirements for Media and Reagents” for guidance.

**External quality assurance:**

Five unknown bacteria isolates will be sent to participating site laboratories two times per year. Bacterial identification will be performed by the bench microbiologist and results compared to peer labs.

**Specimen:**

Preserved stool in Cary-Blair transport medium (Two swabs Preferred). May be refrigerated up to 2 days.

Unpreserved stool should be received within 2 hours of passage.

**Procedure:**

1. **Media inoculation and incubation**

Cary-Blair preserved stool

1. Roll the stool-containing swab from the Cary-Blair medium over a one inch area in zone 1 of the MAC, XLD, TCBS, or other selective media. Use a second swab for the Campy-BAP or Columbia Blood Agar (if used for Campylobacter Culture refer to Appendix 2).
2. Streak for isolation.
3. Incubate plates at 35°C (For Campylobacter Culture refer to Appendix 2).

Zone 1

Zone 2

Zone 3

**2. Culture observation**

Examine all cultures after 18-24 hours incubation.

| **Medium** | Pathogens | Suspect colonies |
| --- | --- | --- |
| MacConkey Agar (MAC) | *Salmonella, Shigella, Aeromonas, Plesiomonas Yersinia, Vibrio, E.coli* | Non-Lactose Fermenter, colorless or transparent, E.coli- PCR archive |
| XLD | *Salmonella, Shigella, Aeromonas, Plesiomonas, Yersinia, Vibrio* | Transparent , red with or without H2S |
| TCBS | *Vibrio, Aeromonas* | Yellow, green, transparent blue-green (any growth) |

1. MAC: Examine plate for non-lactose fermenting (clear) colonies. If all colonies are lactose fermenters (pink), reincubate
2. XLD: Examine plate for clear, red or clear colonies with black centers (H_2_S production). If all colonies are yellow or yellow with black centers, reincubate.
3. TCBS: Examine plate for yellow colonies (sucrose fermenting) or green colonies (non -fermenting). Subculture any colonies, regardless of color, to Tryptic soy agar (TSA) or blood agar (BAP) for oxidase test. Reincubate and reexamine TCBS for 3 days.
4. If no suspicious colonies are observed after 18-24 hours incubation, reincubate overnight and reexamine MAC, XLD and BAP as above. Reexamine TCBS as above for 3 days.

**3. Screen Suspect Colonies**

MAC& XLD

1. Screening biochemical tubes can be inoculated directly from the MAC or XLD if the suspect colony is well isolated. Otherwise, reisolate suspect colonies. At the discretion of the microbiologist, an API20E or MicroScan may be used in lieu of tubes.

2. Select up to 5 suspect colony morphologies, and inoculate a set of biochemicals for each colony: KIA, LDC, MIO and Urea.

3. Incubate at 35^o^C for 18-24 hours.

4. Read and record results.

5. Discard all tubes sets that are K/K, or A/A, H_2_S +/-, gas+/-

6. Perform oxidase from KIA.

**Table 1 Interpretation of Screen Biochemicals**

| KIA  Slant/Butt gas H_2_S | LDC | Motility | Indole | Ornithine | Urea | Oxidase | Suspected  Organism |
| --- | --- | --- | --- | --- | --- | --- | --- |
| K/A gas- H_2_S +/- | + | + | - | - | - | - | *Salmonella* Typhi |
| K/A gas+ H_2_S - | - | + | - | + | - | *-* | *Salmonella* Paratyphi A |
| K/A gas+ H_2_S - | + | + | - | + | - | *-* | *Salmonella* Cholerasuis |
| K/A gas+/- H_2_S- | + | - | - | - | - | *-* | *Salmonella* Gallinarum |
| K/A gas+ H_2_S +/- | + | +/- | - | +/- | - | - | Other *Salmonella* |
| K/A gas- H_2_S- | - | - | +/- | - | - | *-* | *Shigella dysenteriae* |
| K/A gas+/- H_2_S - | - | - | +/- | - | - | *-* | *Shigella flexerni* |
| K/A gas- H_2_S- | - | - | +/- | - | - | *-* | *Shigella boydii* |
| K/A gas- H_2_S- | - | - | - | + | - | *-* | *Shigella sonnei* |
| K/A gas- H_2_S- | +/- | + | + | - | - | + | *Aeromonas spp* |
| K/A gas- H_2_S- | + | + | + | + | - | + | *Plesiomonas shigelloides* |
| K/A gas- H_2_S- | + | + | + | + | - | + | *Vibrio spp* |
| K/A gas- H_2_S- | - | - at 35°C  + at 25°C | +/- | + | + | *-* | *Yersinia spp* |

**A. Urea negative and oxidase negative: Possible *Salmonella* or *Shigella* Refer to Table 1.**

If screening biochemical results indicate *Salmonella* or *Shigella,* test the isolate with the appropriate Denka- Seiken or Difco antisera kits to confirm identification. Refer to Serotype Procedures in Appendix 1.

1. Resolution of Inconclusive or Suspect Serotype Results: If screening biochemical results indicate *Salmonella* or *Shigella,* but the isolate does not agglutinate with any antisera, then attempt to fully identify biochemically using additional tube biochemicals or API 20E. If the supplemental biochemical testing identifies an isolate as *Salmonella* or *Shigella*, then attempt again to confirm with antisera agglutination kits. If the isolate will still not agglutinate, then archive for further testing.
2. Resolution of Inconclusive or Suspect Biochemical Results: If *Salmonella* or *Shigella* are suspected but the biochemical screen results do not completely fit the expected profile, then attempt to resolve by setting up supplemental tube biochemicals or use API20E. If the biochemical testing identifies an isolate as *Salmonella* or *Shigella*, confirm with antisera agglutination kits.

**B. Urea Positive and oxidase negative: Possible *Yersinia* Refer to Table 1.**

1. If screening biochemical results indicate *Yersinia,* set up room temperature motility (25°C).
2. If motile at 25°C and non-motile at 35°C, then perform API20E to confirm *Yersinia* identification.

**C. Urea Negative and oxidase positive: Possible *Plesiomonas shigelloides or Aeromonas spp or Vibrio spp.* Refer to Table 1.**

***Aeromonas and Plesiomonas*** Refer to Table 1 if screening biochemical tube results set up from MAC indicate possible *Plesiomonas shigelloides* or *Aeromonas.* Correlate with BAP growth.

*Aeromonas* appear as large, flat colonies that are oxidase positive. Most *Aeromonas* isolates are beta-hemolytic.on BAP subculture.

*Plesiomonas shigelloides* colonies are shiny, opaque, smooth, non-hemolytic and oxidase-positive

1. Test suspicious colonies for oxidase
2. If all colonies tested are oxidase negative, no further action is required
3. If a colony is oxidase positive and is not obviously *Pseudomonas*, set up API 20E

***Vibrio spp*** Refer to Table 1 and Table 2 if screening biochemical tubes set up from MAC indicate possible *Vibrio* spp (Table 1), correlate with TCBS plate growth.

**TCBS** for *Vibrio:* Examine plate, pay close attention to yellow colonies (sucrose fermenting, possible *V.cholerae*) or translucent, green colonies (non fermenting, possible *V.parahaemolyticus*). Subculture any colonies, regardless of color, to a TSA or BAP. Note: *Aeromonas* is inhibited on TCBS but can grow (green/ translucent)

1. On **BAP subculture**  *Vibrio* appears as large, flat colonies, non-hemolytic and will have a greenish hue.
2. Perform oxidase test from TSA or BAP , *Vibrio* are oxidase positive, except for *V. metschnikovii*
3. If TCBS colonies or tube screened colonies from the MAC or XLD are consistent with *V.cholerae,* confirm identification by testing with antisera O1, Inaba, Ogawa, 0139. Refer to Serotype Procedures in Appendix 1.
4. If screening indicates *V.cholerae* but the isolate does not agglutinate with any antisera, identify biochemically with NaCl growth tubes or API 20E (Table 2).
5. If biochemically identified as *V.cholerae,* attempt again to confirm with antisera. If the isolate will still not agglutinate, archive for further testing.
6. If isolates are suspicious for *V.parahaemolyticus*, set up API20E. If biochemically confirmed as *V.parahaemolyticus or Vibrio spp*, archive for further testing.

**Table 2**

|  | TCBS colony | Oxidase | 0% NaCl | 3% NaCl | 6% NaCl | 8% NaCl | Serogroup |
| --- | --- | --- | --- | --- | --- | --- | --- |
| *Vibrio cholerae* | Yellow | + | + | + | +/- | - | 01 ,Inaba Ogawa;  0139. |
| *Vibrio parahaemolyticus* | Green, translucent | + | - | - | + | + | Archive for reference lab |
| *Aeromonas spp* | inhibited, green | + | + | - | - | - | None |

**D. Archive E.coli for PCR:**

1. After Day 2 incubation, examine MAC for flat, lactose fermenting (pink) colonies. Select 5 colonies that morphologically resemble E. coli to be archived for PCR testing. When there are multiple E. coli-like colony morphologies, choose a variety of morphotypes (up to 5).
2. Reisolate possible E.coli # 1-5 to a TSA. Incubate 35 C for 18-24 hours
3. After incubation, perform an indole test on each of the morphotypes If indole positive,isolates will be archived for PCR If indole negative, but E. coli is suspected, perform additional biochemicals for presumptive E. coli identification. If E.coli is identified, archive for PCR.
4. Refer to SOP “**IDENTIFICATION OF DIARRHEAGENIC E. COLI BY MULTUPLEX PCR** ” for detailed archive instructions.
5. If five presumptive E.coli colony types are not found, return to original plate and pick additional colonies for screening and archive.

**4. Interpretation**

Positive cultures: Isolation of any quantity of *Salmonella* and *Shigella,* *Vibrio, Yersinia, Aeromonas* and *Plesiomonas.*

Negative cultures: The isolation of only normal fecal flora.

**5. Archive**

Archive all pathogens and other isolates that may require additional testing.

*Archiving Isolates for Long Term Storage*

1. Use a 1.8-2.0ml cryovial containing cryopreservation fluid such as TSB with 15% glycerol, skimmed milk media or a commercially prepared product such as “Cryocare” vials by Key Scientific.

2. Inoculate the cryopreservative fluid with a pure culture of 24-48 hour growth to approximately a 3 McFarland standard.

3. Close vial tightly and invert 4-5 times to emulsify the organism. Do not vortex.

4. Store the inoculated cryovial at -70°C for long term archive. Below is the list of codes for each bacterial isolate. Please refer to the SOP for Barcode Sample Tracking for additional information regarding archiving of isolates. The bacterial isolate codes will be printed on the barcode label for each inoculated cryovial.

**Codes for Archived Bacterial Isolates**

| **Organism** | **Code** |
| --- | --- |
| Salmonella Typhi | SALTI |
| Salmonella Paratyphi A | SALPA |
| Salmonella Cholerasuis | SALCH |
| Salmonella Gallinarum | SALGL |
| Salmonella spp | SALSP |
| Salmonella GpB | SALGB |
| Salmonella GpC | SALGC |
| Salmonella GpD | SALGD |
| Other Salmonella | SALOT |
|  |  |
| Shigella dysenteriae GpA | SHIDY |
| Shigella flexneri GpB | SHIFL |
| Shigella boydii GpC | SHIBO |
| Shigella sonnei GpD | SHISO |
| Shigella sp non-typable | SHNTY |
| Aeromonas spp | AERSP |
| Aeromonas hydrophila | AERHY |
| Plesiomonas shigilloides | PLESH |
| Yersinia enterocolitica | YEREN |
|  |  |
| Vibrio cholera (all others) | VIBCH |
| Vibrio Parahaemolyticus | VIBPA |
| Vibrio cholera 01, Inaba | VIBCI |
| Vibrio cholera 01, Ogawa | VIBCO |
| Vibrio cholera 0139 | VIBCN |
|  |  |
| Campylobacter jejuni | CAMJE |
| Campylobacter spp | CAMSP |
|  |  |
| Isolate archived for further testing | IAFFT |

**6. Reporting**

1. Record results on Bacteriology Data Form (BDF) (See below for guidance).
2. Report all pathogens isolated.

| # | Question | Guidance |
| --- | --- | --- |
|  | Participant ID | Write the participant ID in the space provided at the upper left corner. |
| 01 | Sample ID | Write the 9-digit Sample ID. |
| 02 | Date specimen was received | Enter the date that the specimen was received. |
| 03 | Technician code | Enter the technician’s unique ID number who processed the sample. |
| 04 | Was a culture performed? | Indicate whether or not a culture was performed. If no, go to question #12. If yes, continue completing the form (go to question #5). |
| 05 | Was Cary Blair used for the culture? | Indicate whether or not Cary Blair was used for the culture. |
| 06 | Was Aeromonas detected? | Indicate whether or not Aeromonas was detected and, if yes, what type of Aeromonas. |
| 07 | Was Salmonella detected? | Indicate whetheror not Salmonella was detected and, if yes, what type of Salmonella. |
| 08 | Was Plesiomonas detected? | Indicate whether or not Plesiomonas was detected |
| 09 | Was Shigella detected? | Indicate whether or not Shigella was detected and, if yes, what type of Shigella. |
| 10 | Was Vibrio detected? | Indicate whether or not Vibrio was detected and, if yes, what type of Vibrio. |
| 11 | Was Yersinia enterocolitica detected? | Indicate whether or not Yersinia enterocolitica was detected. |
| 12 | What method was used to test for Campylobacter? | Enter the test method for Campylobacter, either ELISA or culture. If ELISA, go to question #13. If culture, go to question #14. |
| 13 | Campylobacter ELISA Result | Record the result of the Campylobacter ELISA test. Refer to the ELISA worksheet, if appropriate. |
| 14 | Was Campylobacter isolated? | Indicate whether or not Campylobacter was isolated and what type of Campylobacter. |
| 15 | Technician code | Enter the technician’s unique ID number who performed the Campylobacter test. |
| 16 | Date of Campylobacter test | Enter the date that the Campylobacter test was performed. |
| 17 | Was E. coli detected? | Indicate whether or not E. coli was observed |
| 18 | Was E. coli archived for PCR? | Indicate whether E. coli was archived for PCR assay. |

7. References

1. Clinical Microbiology Procedures Handbook 2nd Edition, Volume I 3.8.1
2. Manual of Clinical Microbiology, 9th Ed. ASM Press, Washington, DC
3. Color Atlas and Textbook of Diagnostic Microbiology, 6th ed., Chapter 6.
4. Bailey and Scott’s Diagnostic Microbiology 12th Edition
5. Manual for the Laboratory Identification and Antimicrobial Susceptibility Testing of Bacterial Pathogens of Public Health Importance in the Developing, World. World Health Organization 2003.

**APPENDIX 1**

**SALMONELLA, SHIGELLA AND VIBRIO SEROLOGICAL TESTING**

**Purpose:**

Serological tests are based on the fact that antibodies in serum, produced in response to exposure to bacterial antigens, will agglutinate with bacteria carrying homologous antigens. Identification procedures include cultural and biochemical examination in addition to serology. Thus, agglutinating sera is used in confirmatory tests but may also be used with appropriate caution in screening tests.

**Quality control:**

Antisera are checked for reactivity upon receipt and should produce 3+ -4+ agglutination when tested against its complimentary antigen. No reactivity should be observed when with tested with non-complimentary antigen. QC antisera monthly or with each isolate tested if used less frequently, to ensure that no deterioration has occurred.

**Materials:**

Glass slides

Bacteriology loop

Test Tube 13x75

Sterile saline

Appropriate Salmonella, Shigella Vibrio antisera (Store refrigerated) Denka- Seiken or Difco

**References:**

1.Manufactures Package Insert- Denka- Seiken or Difco

2. Manual of Clinical Microbiology ASM Press, 9th Ed 2007

**A. Serological Confirmation of Salmonella spp.**

The majority of Salmonella isolates encountered will belong to O Serogroup A-E. Serotype all isolates biochemically suspect or identified as Salmonella spp. with polyvalent O Group and if necessary, Vi. Isolates that have a biochemical reaction typical of S. Typhi should first be tested with Vi and D antisera. Other suspected Salmonella species will be tested first with the polyvalent O Group. If polyvalent O is positive, then proceed with other O Group mono antisera. Refer to Table 1.

**Procedure:**

1. Prepare bacteria suspension: From the KIA or other non selective media create a heavy suspension of bacteria in 0.5 mL saline, approximately equivalent to a 3 McFarland standard. Mix thoroughly.

2. Divide slides into two sections using a china marker.

3. Place a drop (10 μl) of each antiserum and saline control in separate sections of the slides.

4. Add 10 μl of the bacterial suspension to each antiserum and the saline control.

5. Rock the slide gently and observe for agglutination using indirect lighting over a dark background. Agglutination should be strong and clearly visible within one minute.

6. Examine the saline control suspension carefully to ensure that it is even and does not show clumping resulting from auto agglutination. If auto agglutination occurs, the culture is termed “rough” and cannot be serotyped.

**Note:**

Isolates that have a biochemical reaction typical of S. Typhi should first be tested with Vi and D antisera. If Vi is positive and D is negative, place the organism suspension in boiling water for 15-30 minutes, cool and retest with D antiserum.

**Reporting:**

Report findings in BDF, Bacterial Data Form.

Vi positive and D positive report “Yes, SalmonellaTyphi.”

Positive in Poly A-D and positive in mono A: report “Salmonella Paratyphi A”, (can be biochemically identified; and it is the only commonly encountered Serogroup A)

Positive in Poly A-D and Positive in Mono B. “Salmonella Gp B isolated”

Positive in Poly A-D and Positive in Mono C. “Salmonella Gp C isolated”

Positive in Poly A-D and Positive in Mono D, Negative in Vi. “Salmonella Gp D isolated”

Negative in Poly A-D and highly suspicious for Salmonella: do API and archive for further testing.

**B. Serological Confirmation of Shigella spp.**

Serological confirmation of Shigella isolates is based only on O antigen testing. If the isolate is biochemically a Shigella species, test suspect colonies with grouping antisera: A-D, Shigella dysenteriae GpA; Shigella flexneri GpB; Shigella boydii; GpC; Shigella sonnei GpD.

**Procedure:**

1. Prepare Bacterial Suspension: From KIA or other non-selective media, suspend bacterial growth from a pure culture of biochemically identified Shigella spp. in 0.5 mL saline to the turbidity of a 1 McFarland standard. Mix thoroughly.

2. Partition glass slides using the glass marker pencil. Each specimen requires five sections (A-D antiserum and one saline control).

3. Place a drop (10 µl) of each antiserum (A – D) and saline control in separate sections of the slides.

4. Add 10 μl of the bacterial suspension to each antiserum and the corresponding saline on the slide.

5. Rock the slide gently and observe for agglutination using indirect lighting over a dark background. Agglutination should be strong and clearly visible within one minute.

6. Examine the saline suspension carefully to ensure that it is even and does not show clumping resulting from auto agglutination. If auto agglutination occurs, the culture is termed “rough” and cannot be serotyped.

7. Confirm Serogroup and biochemical identification correlate.

**Reporting:**

Report findings on Bacteriology Data Form (BDF).

Group A positive =Shigella dysenteriae GpA

Group B positive= Shigella flexneri GpB

Group C positive= Shigella boydii; GpC

Group D positive =Shigella sonnei GpD

Negative in A-D and highly suspicious for Shigella, do API and repeat agglutination if necessary.

**C. Serological Confirmation of Vibrio cholerae**

Identification of Vibrio cholerae can be confirmed by slide agglutination testing with polyvalent O1 or O139 antisera. Fresh growth of suspected V. cholerae from a nonselective agar medium should be used. Using growth from TCBS agar may result in false-negative reactions

**Procedure:**

1. Suspend bacterial growth from a pure culture of biochemically suspected Vibrio cholerae in 0.5 mL saline. Mix thoroughly to create a moderately milky suspension.

2. Partition glass slides using the glass marker pencil. One section for each antiserum and one saline control.

3. Place a drop (10 μl) of each antiserum and saline control in separate sections of the slides.

4. Add 10 μl of the bacterial suspension to each antiserum and the saline control on the slide.

5. Rock the slide gently and observe for agglutination using indirect lighting over a dark background. Agglutination should be strong and clearly visible within one minute.

6. Examine the saline suspension carefully to ensure that it is even and does not show clumping resulting from auto agglutination. If auto agglutination occurs, the culture is termed “rough” and cannot be serotyped.

**Note:** If an isolate is positive in the polyvalent O1 or in the O139 antiserum, it may be reported as V. cholerae O1 or O139. Presumptive V. cholerae O1 isolates may then be tested in monovalent Ogawa and Inaba antisera. Inaba and Ogawa antisera should never be used with strains that are negative with polyvalent O1 antiserum. Strains of one serotype frequently produce slow or weak agglutination in antiserum to the other serotype. For this reason, agglutination reactions with Inaba and Ogawa antisera should be examined simultaneously, and the strongest and most rapid reaction should be used to identify the serotype.

**Reporting:**

Report findings on Bacteriology Data Form (BDF).

Positive in 01 only report “Vibrio Cholerae 01”

Positive in 01 and Ogawa report “Vibrio cholerae 01 Ogawa”

Positive in 01 and Inaba report “Vibrio cholerae 01 Inaba”

Positive in 0139 report “Vibrio cholerae 0139”

Negative in 01 and Negative in 0139, confirm identification biochemically and archive for further testing. Report “Presumptive Vibrio cholerae non-01”

**APPENDIX 2 IDENTIFICATION OF CAMPYLOBACTER IN STOOL**

Purpose:

Campylobacter species, especially Campylobacter jejuni, are major causes of acute diarrhea in humans. Campylobacteriosis is characterized by fever, abdominal pain, nausea, muscle pain, and watery diarrhea. The ProSpecT Campylobacter Microplate Assay is a solid-phase immunoassay for the detection of Campylobacter Specific Antigen (CSA). Diluted stool is added to plastic microtiter wells containing antibody to CSA. Any antigen present in the sample will be bound by this antibody and is subsequently detected by an anti-CSA conjugate. The diagnosis of intestinal Campylobacter infection has historically relied upon culture using selective media incubated at elevated temperatures in a microaerophilic atmosphere. Studies comparing the ProSpecT Campylobacter microplate antigen assay to standard culture show functional equivalence of the two methods Thus, the identification of *Campylobacter* in stool specimens can be done using either the ProSpecT microplate assay or culture.

**A. ProSpecT *Campylobacter* Microplate Assay**

**Specimen:**

1. Fresh, unpreserved stool specimens may be stored at 2-8°C and tested within 72 hours. Store stools at -20°C or lower if testing is to be performed later than 72 hours. Avoid repeated freeze-thawing.

2. Specimens in the Bacterial Specimen Diluent and stored refrigerated at 2-8°C for up to 72 hours prior to testing. (See Specimen Preparation below)

3. Stool specimens collected in Cary-Blair should be refrigerated at 2-8°C and tested within 1 week after collection.

**Materials:**

Kit Components:

1. Microwell strips–coated with rabbit polyclonal anti-CSA antibody
2. Enzyme conjugate–HRP-labeled rabbit polycolonal anti-CSA antibody with 0.01% thimerosal
3. Positive control–*C. jejuni* culture supernatant suspended in Negative Control with fetal bovine serum and 0.02% thimerosal
4. Negative control–human fecal material with rabbit serum and 0.02% thimerosal
5. Bacterial specimen diluent–buffered solution with rabbit serum and 0.02% thimerosal
6. Wash buffer–10X concentrate with 0.1% thimerosal, diluted before use (see below)
7. Color substrate–TMB in buffer
8. Stop solution–0.5N HCl

Other supplies

1. transfer pipettes
2. microplate cover
3. glass tubes
4. wooden sticks
5. distilled or deionized water
6. laboratory timer
7. Preparation of reagents:
8. Bring all reagents to room temperature before use. Mix gently.
9. Dilute 10X wash buffer concentrate to 1X by adding 1 part concentrate to 9 parts distilled or deionized water. Diluted wash buffer is stable for 1 month at 4°C.

**Quality control:**

1. Positive and Negative Controls must be included each time the test is performed.

2. The Positive and Negative Controls serve as both reagent and procedural controls.

3. The optical density (O.D.) of the Negative Control should be <0.100 at 450 nm or <0.070 at 450/630 nm. The Negative Control should be colorless when read visually.

4, The O.D. of the Positive Control should be >0.500 at 450nm or 450/630nm. Visually the intensity of color in the Positive Control should be equal to or greater than the 2+ reaction on the procedure card.

**Procedure:**

*Specimen preparation*

1. Specimens in Cary-Blair may be tested directly.

2. For unpreserved stool, add 0.6 ml Bacterial Specimen Diluent to a clean glass or plastic tube. Mix liquid or loose stool as thoroughly as possible. Using a kit-provided transfer pipette, transfer 0.3 ml of sample (third mark from tip opening) to the tube with diluent. Mix by drawing the sample up and down ONCE. Leave pipette in tube.

3. For formed stools: Using a wooden stick, transfer 0.3 gram (size of small pea) into the tube with diluent. Emulsify the stool using the applicator stick. Place a kit-provided transfer pipette into the tube and mix by drawing the sample up and down ONCE. Leave pipette in tube.

*Test Procedure*

Complete a ProSpecT Campylobacter Microplate Assay worksheet (internal lab worksheet in kit). Record the lab specimen number in the box corresponding to the well location in the actual microtiter plate. NOTE: Skip a well location between each control and patient sample.

Remove enough microplate strip wells from the foil pouch for each sample and control to be tested. Be sure to re-seal the pouch after opening to exclude moisture and return to the refrigerator.

1. Add 4 drops of Negative Control to the Neg Con well.

2. Add 4 drops of Positive Control to the Pos Con well.

3. Using a transfer pipette, add 4 drops of diluted specimen to the corresponding wells. Take care not to splash sample between wells.

4. Cover the microplate with the provided microplate cover and incubate at room temperature (20-25°C) for 60 minutes. Begin timing after the addition of the last patient sample.

5. Wash

First Wash: Shake out the contents of the wells over a sink. Wash by completely filling each well with diluted Wash Buffer. Shake out all fluid from the wells after each wash. **Wash a total of 3 times.**  After the last wash, remove all fluid from the wells by dumping the contents and striking the plate on clean paper towels. Remove as much buffer as possible but do not allow the wells to dry out before proceeding to the next step.

6. Add 4 drops of Enzyme Conjugate to each well. Cover the microplate and incubate at room temperature for 30 minutes.

Second wash – shake out the contents of the wells and **wash 5 times using the same technique described** as in step 5 above. After the last wash, remove all fluid from the wells by dumping the contents and striking the plate on clean paper towels.

Add 4 drops of Color Substrate to each well. Cover the microplate and incubate at room temperature for 10 minutes.

Add 1 drop of Stop Solution to each well. Gently tap the microplate until the yellow color is uniform. Read reactions within 10 minutes after adding the Stop Solution.

**Interpretation:**

A. Reading plates visually (refer to Procedure Card)

1. Read the controls:

*Positive control* – should be equal to or greater than the 2+ reaction on the Procedure Card.

*Negative control* – colorless. NOTE: if a yellow color develops that is equal to or greater than 1+ on the Procedure Card, the test should be repeated with careful attention to the wash procedure.

1. Patient Samples

Negative: Colorless

Indeterminate: Faint yellow color, less than the 1+ reaction

Positive: Yellow color of at least 1+ intensity

B. Reading Spectrophotometrically at 450nm (single wavelength) and/or 450/630-650nm (dual wavelength).

Single Wavelength 450nm  **Fresh Stool Transport Media**

**Negative:** OD <0.130 <0.100

**Indeterminate:** OD 0.130-0.170 0.100 – 0.130

**Positive:** OD >0.170 >0.130

Dual Wavelength 450/630-650 **Fresh Stool Transport Media**

**Negative:** OD <0.100 <0.070

**Indeterminate:** OD 0.100-0.140 0.070 – 0.100

**Positive:** OD >0.140 >0.100

**Interpretation of results:**

1. Negative – no *Campylobacter* SA or an undetectable level of *Campylobacter* SA is present in the sample.
2. Indeterminate:

a) Indeterminate samples should be repeated.

b) If the repeat test is positive, the specimen is positive.

c) If the repeat test is negative, the specimen is negative.

d) If the repeat test is again indeterminate, a new sample should be obtained and tested.

1. Positive – the sample contains *Campylobacter* SA.

**Reporting:**

Report findings on Bacteriology data form, BDF.

Test negative – report “Campylobacter antigen test negative.”

Test indeterminate – report “Campylobacter antigen test indeterminate

Test positive – report “Campylobacter antigen test positive.”

**Procedure notes:**

Other than the Wash Buffer, all provided reagents are provided at working strength; do not dilute.

Microwell strips must be stored in the resealable foil pouch to protect microwells from moisture.

Add reagents to the test wells in the same order throughout the procedure.

**References:**

Package Insert ProSpecT Campylobacter Microplate Assay.**B. CAMPYLOBACTER CULTURE AND IDENTIFICATION**

**Inoculation and incubation:**

Use a swab from Cary-Blair to inoculate a Campy-BAP. If using unpreserved stool inoculation, this must be done immediately after receiving the sample (no longer than 2 hours after passage). Place in a microaerophilic atmosphere (example Campy-Gen) and incubate at 42 C for up to 3 days.

**Culture observation:**

After 48-72 hours incubation, observe plate for colonies resembling Campylobacter: nonhemolytic with irregular edges, colonies may spread or swarm on moist surface, if no suspect colonies are seen at 48 hours, reincubate overnight and reexamine plate.

**Screen suspect colonies:**

The following tests are performed on suspected colonies:

Campylobacter spp

Gram Stain: Gram-Negative curved bacilli

Oxidase: Positive

Catalase: Positive

Sodium Hipurate Hydrolysis: Positive (purple ring) C.jejuni or Negative (yellow) Campylobacter spp.

OXIDASE - use sterile wooden sticks, never metal. The isolations have to be in a non-selective medium with neutral pH, since acid mediums tend to neutralize oxidase activity. Place an aliquot of the culture medium on filter paper previously dampened with a drop of the oxidase reagent and wait 30 seconds. A purple tint is read as POSITIVE.

CATALASE - use sterile wooden sticks, never metal. Place an aliquot of the culture medium in a drop of 3% hydrogen peroxide reagent (or in the spot catalase test) placed on a slide. The presence of bubbles after a few seconds indicates oxygen formation and is read as POSITIVE.

HIPURATE HYDROLISIS - Take a sample with inoculating loop and place it in a tube with 400 uL of 1% sodium hipurate, incubate for 2 hours at 37°C in a water bath. Add 200 uL of 3.5% Ninhidrine reagent (avoid removing sample from water bath) allow to incubate for 10 minutes more. A purple ring is read as POSITIVE. A yellow ring is NEGATIVE.

**Reporting:**

Record findings on Bacteriology data form (BDF).
Report “C.jejuni” or “Campylobacter species” if positive. Report “No” if no Campylobacter was isolated.

**References:**

1. DIFCO LABORATORIES. 1998. "Manual Difco". Division of Becton Dickinson and Company. Sparks, Maryland 21152-USA

2. MAC FADDIN, j. 1980. "Biochemical Tests for Identification of Medical Bacteria", Second edition. Edit. Willams & Wilkins. Baltimore-USA.

3. MURRAY, P.; BARON, E.; PFALLER, M.; TENOVER, F.; YOLKEN, R. 1999. Manual of Clinical Microbiology". 7th Edition. American Society for Microbiology. Washington D.C-USA

4. Nachamkin Irvin, Blaser Martin. 2000 “Campylobacter” 2^nd^ edition. Edit ASM Press. Washington DC –USA.

ECP—Identification of Diarrheagenic E.coli by Multiplex PCR SOP

**This SOP has been read and understood by:**

| **Name** | **Date** |
| --- | --- |
|  | | |
|  |  | |
|  |  | |
|  |  | |
|  |  | |
|  |  | |
|  |  | |
|  |  | |
|  |  | |
|  |  | |
|  |  | |

**Document History:**

| **Version Number** | **Reason for Changes** | **Date** |
| --- | --- | --- |
|  |  |  |
|  |  |  |
|  |  |  |
|  |  |  |

Protocol for the identification of 5 strains of diarrheagenic E. coli using a 9-plex PCR.

**PRINCIPLE**

E.coli can be isolated from nearly all human fecal cultures. Some strains of E.coli are known to be diarrheagenic and are an important cause of diarrhea in children, particularly in the developing world. Pathogenic strains of E.coli are generally categorized according to the toxins produced or virulence associated characteristics. This procedure describes the identification of diarrheagenic E.coli using a 9-plex PCR assay. DNA from E.coli colonies cultured from stool is extracted and a multiplex PCR assay is performed. The PCR products are separated using agarose gel electrophoresis; bands are visualized under UV illumination and photographed. 9 associated virulence genes tested in this multiplex PCR assay are:

Stx1 348 bp

Stx2 584 bp
LT 508 bp

ST 147 bp
eae 881 bp

bfpA 300 bp
ipaH 423 bp
aatA 650 bp

aaiC 215 bp
**SAFETY**

Specimens should be handled, processed and disposed of using standard guidelines for biohazardous materials. Spills should be immediately disinfected.

**QUALITY CONTROL**

A positive control and a negative control must be included in each run. The positive control is made up of a combination of characterized E.coli strains that includes every virulence gene tested.

The negative control is E.coli ATCC 25922 or another E.coli strain negative for all virulence genes.

**EXTERNAL QUALITY ASSURANCE**

Five E.coli isolates will be sent to participating site laboratories two times per year. Identification will be performed and results compared to peer labs.

**SPECIMEN**

Refer to SOP Stool Bacteriology Culture and Identification for instructions on initial colony selection. Subculture the 5 morphotypes onto non selective media i.e. TSA or BAP, incubate at 35-37°C for 24-48 hours to obtain fresh growth for testing. The five E.coli morphotypes will be tested in a pool.

**SUPPLIES AND EQUIPMENT**

1. Qiagen multiplex PCR kit
2. microcentrifuge tubes and racks
3. nuclease free water (nfw)
4. 20uM primer forwards ETEC508F, ETEC147F, STEC (EHEC) 348F, STEC (EHEC)584F, EPEC881F, EPEC300F, EIEC423F, EAEC650F, EAEC215F
5. 20uM primer reverses ETEC508R, ETEC147R, STEC (EHEC) 348R,STEC (EHEC) 584R, EPEC881R, EPEC300R, EIEC423R, EAEC650R, EAEC215R
6. P200 pipette and tips
7. P20 pipette and tips
8. P10 pipette and tips
9. vortex
10. microcentrifuge
11. Thermocycler
12. PCR plates, seals
13. Gel electrophoresis set up with 100bp ladder and camera.

**PROCEDURE**

1. **Isolate Preparation:**

Use 24-48 hour colony growth from non selective media

1. **DNA Extraction:**

DNA from E.coli colonies can be extracted using a 0.5% Triton boil method:

1. Add 1ml nuclease free water and 5ul of Triton X-100 to a microcentrifuge tube.
2. A. Test isolates: Add 1-2 colonies of each of the 5 E.coli morphotypes to the single tube, (try to add an equal amount of each type). Vortex 5 seconds

B. Postive Controls: Combine 2 colonies of each of the control strains into a single tube Vortex 5 seconds

1. Boil or incubate in a heat block at 100C for 20min
2. Centrifuge at 10,000 rpm for 10 minutes
3. Use supernatant directly for PCR.
4. **PCR:**

Determine the number of reactions to set up per run. Prepare the master mix in a clean DNA free area. Reaction master mix is made and dispensed into the 96-well plates. Extracted DNA is then added. A negative control and positive control is included in each run.

Prepare master mix and PCR plate

1. Thaw Qiagen multiplex MM, Q-solution, and primers in a DNA free room

2. Vortex Qiagen multiplex MM, Q-solution, and primers

3. Prepare master mix in a 1.5ml microcentrifuge tube:

**9-Plex Master Mix for E. coli Assay**

Per Well:

1. Qiagen multiplex MM 25.00 µl
2. 20µM ETEC508F 0.5 µl
3. 20µM ETEC508R 0.5 µl
4. 20µM ETEC147F 0.5 µl
5. 20µM ETEC147R 0.5 µl
6. 20µM STEC (EHEC)348F 0.5 µl
7. 20µM STEC (EHEC)348R 0.5 µl
8. 20µM STEC (EHEC)584F 0.5 µl
9. 20µM STEC (EHEC)584R 0.5 µl
10. 20µM EPEC881F 0.5 µl
11. 20µM EPEC881R 0.5 µl
12. 20µM EPEC300F 0.5 µl
13. 20µM EPEC300R 0.5 µl
14. 20µM EIEC423F 0.5 µl
15. 20µM EIEC423R 0.5 µl
16. 20µM EAEC650F 0.5 µl
17. 20µM EAEC650R 0.5 µl
18. 20µM EAEC215F 0.5 µl
19. 20µM EAEC215R 0.5 µl
20. Q-solution 5.00 µl
21. nfw 6.00 µl

4. Aliquot 45ul of master mix into each well

5. Place lid on the PCR plate and take the plate to another room for the DNA addition

6. Vortex extracted DNA

7. Add 5ul of DNA to sample or control wells

8. Seal plate

9. Run PCR

PCR cycling conditions

1× 15 min 95°C

40× 30 sec 94°C

90 sec 58°C

90 sec 72°C

1× 10 min 72°C

1× ∞ 4°C

**D.** **Post PCR**

A. Egel: When using the Egel apparatus from Invitrogen, use a 2% gel cartridge with either Ethidium Bromide (EtBr) or SYBR safe. For best band resolution use 2 ladders: 1) E-Gel® Low Range Quantitative DNA Ladder (100bp to 2000bp) and 2) TrackIt™ 50 bp DNA Ladder (50bp to 800bp). Use Mode 1 and run E-gel for 26 min. Image gel using either UV light for EtBr or safe imager for SYBR safe cartridges.

B. In house electrophoresis: Prepare 2% agarose gel and stain with EtBr. Use 2 ladders for best band resolution (100bp to 2000bp and 50bp to 800bp. Run the gel at 50V for 45 min.

**INTERPRETATION**

Positive: Presence of any virulence gene bands,

E. coli pathotypes were defined based on the presence of the following genes:

Shiga-toxin producing E. coli (STEC): stx1 or stx2,

ST-producing enterotoxigenic E. coli (ST-ETEC): ST and LT or ST alone,

LT-producing enterotoxigenic E. coli LT-ETEC: LT without ST,

typical enteropathogenic E.coli (EPEC): eae and bfpA,

atypical EPEC: (aEPEC): eae, (without bfpA, or stx1,or stx2),

enteroinvasive E.coli (EIEC): ipaH,

enteroaggregative E. coli: (EAEC) aatA or aaiC

Negative: No bands

**ARCHIVE**

E.coli pools positive for any of the virulence genes will be archived as a pool. To archive select 10 colonies of each morhoptype and combine all 5 morphotypes into one freezer vial. Store at -70 °C

**REPORTING**

1. Record information and results on the E. coli PCR (ECP) data form. Save the gel photograph for review.
2. If no bands are observed, report “No” for all nine genes.

| # | Question | Guidance |
| --- | --- | --- |
|  | Participant ID | Write the child’s participant ID in the space provided at the upper left corner. |
| 01 | Sample ID | Write the 9-digit Sample ID |
| 02 | Was E. coli PCR performed? | Record whether or not E. coli PCR was performed. |
| 03 | Technician code | Enter the technician’s unique ID number who performed the E. coli PCR. |
| 04 | Date of E. coli PCR | Enter the date (DD/MMM/YY) that the E. coli PCR was performed |
| 05-13 | Detection of the nine virulence genes | Record which of the nine virulence genes were detected. If no bands are observed, report “No” for all virulence genes. |

**REFERENCES**

1.Manual of Clinical Microbiology, 9^th^ Ed. ASM Press, Washington, DC

2.Taniuchi M, Walters CC, Gratz J, et al. Development of a multiplex polymerase chain reaction assay for diarrheagenic Escherichia coli and Shigella spp. and its evaluation on colonies, culture broths, and stool. Diagn Microbiol Infect Dis 2012; 73(2): 121-8

3.Nguyen et al. Detection and Characterization of diarrheagenic *E. coli* from young children in Hanoi, Vietnam. JCM 2005; 43(2):755-760.

4. Multiplex PCR for Diagnosis of Enteric Infections Associated with Diarrheagenic Escherichia coli Roberto Vidal, Maricel Vidal, Rossana Lagos, Myron Levine and Valeria Prado J. Clin. Microbiol. 2004, 42(4):1787. DOI: 10.1128/JCM.42.4.1787-1789.2004.

**5.** Luscher, D., and M. Athwegg. 1994. Detection of Shigellae, enteroinvasive and enterotoxigenic Escherichia coli using the polymerase chain reaction (PCR) in patients returning from tropical countries. Mol. Cell. 8:285-2

E. coli type Target Gene Nguyen et al Protocol Length

EAEC aaiC n/a 215bp

EAEC aatA 630bp 630bp (650 is more accurate)

EHEC Stx 1 130bp 348bp

EHEC Stx 2 298bp 584bp

EPEC bfpA 367bp 300bp

EPEC/EHEC eae 376bp 881bp

ETEC LT 322bp 508bp

ETEC ST 147bp 147bp

EIEC ipaH n/a 423bp

**PRIMER SEQUENCES**

**ETEC (LT)**

ETEC508F 5′-CACACGGAGCTCCTCAGTC-3′

ETEC508R 5′-CCCCCAGCCTAGCTTAGTTT-3′

**ETEC (ST)**

ETEC147F 5′-GCTAAACCAGTA^G^_A_GGTCTTCAAAA-3′

ETEC147R 5′-CCCGGTACA^G^_A_GCAGGATTACAACA-3′

**EHEC (Stx1)**

EHEC348F 5’- CAGTTAATGTGGTGGCGAAGG -3’

EHEC348R 5’- CACCAGACAATGTAACCGCTG -3’

**EHEC (Stx2)**

EHEC584F 5’- ATCCTATTCCCGGGAGTTTACG-3’

EHEC584R 5’- GCGTCATCGTATACACAGGAGC -3’

**EPEC (eae)**

EPEC881F 5′-CCCGAATTCGGCACAAGCATAAGC-3′

EPEC881R 5′-CCCGGATCCGTCTCGCCAGTATTCG-3′

**EPEC (bfpA)**

EPEC300F 5’-GGAAGTCAAATTCATGGGGGTAT-3’

EPEC300R 5’-GGAATCAGACGCAGACTGGTAGT-3’

**EIEC (ipaH)**

EIEC423F 5’-TGGAAAAACTCAGTGCCTCT -3’

EIEC423R 5’-CCAGTCCGTAAATTCATTCT -3’

**EAEC (aatA)**

EAEC650F 5’-CTGGCGAAAGACTGTATCAT-3’

EAEC650R 5’-CAATGTATAGAAATCCGCTGTT-3’

**EAEC (aaiC)**

EAEC215F 5’-ATTGTCCTCAGGCATTTCAC-3’

EAEC215R 5’-ACGACACCCCTGATAAACAA-3’

FVF—Fecal Viral ELISA Tests

**This SOP has been read and understood by:**

| **Name** | **Date** |
| --- | --- |
|  |  | |
|  |  | |
|  |  | |
|  |  | |
|  |  | |
|  |  | |
|  |  | |
|  |  | |
|  |  | |
|  |  | |

**Document History:**

| **Version Number** | **Reason for Changes** | **Date** |
| --- | --- | --- |
| 3.0 | Washer buffer modified | 11/JAN/10 |
|  |  |  |
|  |  |  |
|  |  |  |

**I. Purpose**

To determine the presence or absence of Adenovirus, Astrovirus, and Rotavirus antigens in stool specimens by enzyme immunoassay.

**II. Material**

Stored in the refrigerator at 2-8°C:

1. ProSpecT Rotavirus R240396 kit (usable 16 wks after opening)
2. ProSpecT Adenovirus R240096 kit
3. ProSpecT Astrovirus R240196 kit

Each kit contains:

1. One Microtiter plate

2. One bottle of Positive Control

3. One bottle of Conjugate

These are test-specific.

One bottle each of

1. Sample Diluent

2. Wash Buffer (X25)

3. Substrate TMB

4. Stop Solution

These are test-interchangeable.

Containers for collection of fecal specimens

Clean screw-capped disposable containers for preparing fecal suspensions

Clean vial for negative control

Clean absorbent paper

Waste container with disinfectant (bleach)

Automated plate washer (optional)

EIA plate reader capable of reading 96 well plate at absorbance of 450nm with reference 620-650nm

**III. Methods**

All tests involve addition of fecal suspensions to coated microwells, incubation, and detection with horseradish peroxidase conjugated detection antibody.

The ProSpecT Rotavirus test detects Group A rotavirus major inner capsid protein (VP6); capture and detection antibodies are polyclonal.

The ProSpecT Adenovirus test detects all human adenovirus serotypes via a genus-specific adenovirus hexon antigen; capture and detection antibodies are monoclonal.

The ProSpecT Astrovirus test detects all known strains of human astroviruses; capture antibody is a genus specific polyclonal antibody. Capture antibody is a dextran conjugated genus specific monoclonal antibody which incorporates multiple horseradish peroxidase molecules and thereby amplifies the signal.

**Quality control:**

A positive control and negative control (diluent) for each viral assay must be tested with every test run.

**Specimen:**

Refer to SOP SSC “Stool Specimen Collection and Transport” for details.

In setting of diarrhea, collect fecal specimens as soon as possible after onset. Peak excretion of rotavirus and astrovirus occurs 3-5 days and 3-13 days, respectively, after onset.

1. Specimens should be collected into empty containers with no additives (media, preservatives, sera, ions, oxidizers, detergents).

2. Rectal swabs may be used if sufficient material to obtain a 10% suspension for testing.

3. Storage at 2-8C is allowed for 8 days before testing.

4. Storage at -20C to -70C is allowed “long term” before testing.

5. ProSpecT Adenovirus sample also allows testing of fecal suspensions preserved in formalin however we will not use this specimen routinely/ Refer to SOP QNS, “Processing and Testing QNS Stool Samples” for guidance.

**Procedure:**

1. Open and prepare each kit.

Bring reagents to room temperature.

Return unused microwells to sealed pouch for storage at 2-8C.

Prepare working strength wash buffer (x10): 1 part concentrate: 9 parts deionized or distilled water. Unused working strength wash buffer can be stored by 2-8C for up to 30 days for subsequent use.

Protect substrate from light.

Return all materials to 2-8C upon completion.

2. Prepare fecal specimens.

Add approximately 0.1g feces (small pea sized portion), 100ul of liquid stool, or a rectal swab to 1ml of sample diluent (any of the 3 kit’s sample diluent is acceptable) in a vial. Mix and let settle for 10 minutes.

3. Test Procedure.

a. Count wells: count number of samples to test + 1 positive control + 1 negative control.

b. Add 2 drops of positive control to positive control well, add 2 drops (or 100ul) of sample to each sample well, and add 100ul of diluent to negative control well.

c. Add 2 drops of kit-specific conjugate to each microwell, mix gently for 20-30 sec.

d. Incubate at room temperature for 55-65 minutes.

e. Wash by instilling and removing approximately 350ul of working-strength wash buffer. Repeat 5 times. After the final wsh, tap inverted plate on absorben towels to remove the last traces of wash buffer. *Note:* washing is the most error-prone step for EIA testing.

f. Add 2 drops of substrate to each well.

g. Incubate at room temperature for 10 minutes.

h. Read and record visually.

i. Add 2 drops of stop solution.

j. Read in EIA reader set at 450 nm within 30 min of addition of stop solution.

**Interpretation:**

All positive control reactions must be visually positive and have OD > 0.500.

All negative control reactions should be colorless and have OD < 0.150.

Visual

Any sample that is more intensely blue than the negative control is positive.

Any specimen visually equal or less blue than negative control is negative.

If, after addition of substrate, a microwell turns dark blue and forms a blue black precipitate the sample is positive.

ELISA reader

Calculate cutoff value by adding 0.10 to the negative control.

Positive: Any sample above the cutoff (negative control + 0.10 OD is positive.

Negative: Any sample below the cutoff is negative.

Equivocal: Any sample within 0.010 of the cutoff. These samples should be retested.

**Reporting:**

1. Record and report the Visual Interpretation and the ODs and OD interpretation on the ELISA Worksheet and report results on the Fecal Viral Form (FVF).

| # | Question | Guidance |
| --- | --- | --- |
|  | Participant ID | Write the child’s participant ID in the space provided at the upper left corner. |
| 01 | Sample ID | Write the 9-digit Sample ID. |
| 02 | Date specimen was received | Enter the date that the specimen was received. |
| 03 | Technician code | Enter the technician’s unique ID number who processed the sample. |
| 04 | Was Adenovirus ELISA performed? | Enter yes or no if the Adenovirus ELISA was performed. If test was not performed, go to question #8. |
| 05 | Technician code | Enter the technician’s unique ID number who performed the Adenovirus ELISA test. |
| 06 | Date of Adenovirus ELISA test | Enter the date that the Adenovirus ELISA test was performed. |
| 07 | Result | Record the result of the ELISA test. Refer to the ELISA worksheet, if appropriate. |
| 08 | Was Astrovirus ELISA performed? | Enter yes or no if the Astrovirus ELISA was performed. If test was not performed, go to question #12. |
| 09 | Technician code | Enter the technician’s unique ID number who performed the Astrovirus ELISA test. |
| 10 | Date of Astrovirus ELISA test | Enter the date that the Astrovirus ELISA test was performed. |
| 11 | Result | Record the result of the ELISA test. Refer to the ELISA worksheet, if appropriate. |
| 12 | Was Rotavirus ELISA performed? | Enter yes or no if the Rotavirus ELISA was performed. If test was not performed, go to question #16. |
| 13 | Technician code | Enter the technician’s unique ID number who performed the Rotavirus ELISA test. |
| 14 | Date of Rotavirus ELISA test | Enter the date that the Rotavirus ELISA test was performed. |
| 15 | Result | Record the result of the ELISA test. Refer to the ELISA worksheet, if appropriate. |

NRV—Detection of Norovirus by RT-PCR

**This SOP has been read and understood by:**

| **Name** | **Date** |
| --- | --- |
| **1.** |  | |
| **2.** |  | |
| **3.** |  | |
| **4.** |  | |
| **5.** |  | |
| **6.** |  | |
| **7.** |  | |
| **8.** |  | |
| **9.** |  | |
| **10.** |  | |

**Document History:**

| **Version Number** | **Reason for Changes** | **Date** |
| --- | --- | --- |
| 2.1 | Changed RNA extraction to QIAamp Viral RNA Kit | 02Jan2010 |
| 3.1 | Inclusion of Qiagen extraction protocol and minor comments regarding site-specific adjustments for real-time PCR | 02Sep2010 |
|  |  |  |
|  |  |  |

**1. Purpose**

Human caliciviruses (HuCVs) have been recognized to be the leading cause of outbreaks of gastroenteritis in adults and an important cause of gastroenteritis in children in developed countries. HuCVs comprise noroviruses (NoVs), previously referred to as ‘‘Norwalk-like viruses’’ or ‘‘small round structured viruses’’ and Sapoviruses (SaVs), which are small (27–32 nm in diameter), single-stranded positive sense RNA viruses recently designated as separate genera in the Caliciviridae family. The genus Norovirus has extensive genetic diversity and is divided into at least two genogroups, I (GI) and II (GII) based on sequence analysis of genes encoding structural and nonstructural proteins This protocol describes the RNA extraction and real time RT-PCR procedures for the identification of Norovirus genogroup I and II from human fecal specimens

**2. Quality Assurance**

A. Quality Control

Positive and negative controls are included in each run.

Positive Control: A sample previously confirmed as positive for Norovirus

Negative Control: DEPC treated water as negative control.

Acceptance criteria are specified in the protocol. If the acceptance criteria are not met, further examination, documentation and review may be conducted.

B.Assay Validation Prior to assay implementation for study samples, each of the site laboratories performing Norovirus RT-PCR will receive a 5 (undiluted) stool specimens, which have been well characterized. The results of testing of these 10 samples must be returned to the microbiology sub-committee within 6 weeks of receipt of the samples. Laboratories with recurring problems in detection will be assisted with retraining.

C.EQA Twice a year, a panel of norovirus positive and negative samples will be used to assess the accuracy and reproducibility of results from all study sites. The panel will consist of 5 samples and will include both GI and GII positives. Each study site will test all samples independently from making fecal suspensions to real time PCR. This protocol describes the experiments and analysis required for characterization of noroviruses from human fecal samples.

**3. Materials**

The following are needed for sample preparation, storage, RNA extraction and real time RT-PCR.

1. Biological safety cabinet
2. Vortex
3. Microcentrifuge
4. Water bath
5. Dry baths
6. Pipettes (0.5-10 µl, 5-20 µl, 20-200 µl and 100-1000 µl)
7. Real Time Thermal cycler
8. -70°C freezer
9. -20°C freezer
10. QIAamp Viral RNA Kit ( Qiagen cat 52906 or 52904)
11. Ag-Path One-Step RT-PCR Kit (Ambion cat no. AM1005)
12. Nuclease Free Water
13. 96%-100% Ethanol
14. PCR Primers & Probes (Kageyama et al. J.Clin. Microbiol. 2003) refer to Table 2
15. 1.5 ml Microcentrifuge tubes
16. PCR plates or strips

**4. Sample**

Aliquot ~0.1 g of unpreserved fecal sample into a 2 ml cryovial and store at -70°C prior to processing.

**5. Preparation of Fecal Suspension**

1. Thaw specimen and prepare 10-20% fecal suspensions in 0.89% NaCl in 2ml screw cap tubes (0.1g fecal sample in 1 ml NaCl)

2. Centrifuge at 4000g for 20 minutes

4. Use 140 ul of the filtrate as starting material following the QIAamp Viral RNA extraction procedure

NOTE Fecal suspension may be stored at 4°C for up to 72 hours prior to extraction of viral RNA until processing. Once all investigations have been carried out the remaining suspension can be stored at -20ºC or 70°C.

Avoid freezing and thawing the specimens repeatedly.

**6. Extraction of viral RNA Using QIAamp Viral RNA Kit**

Refer to QIAamp Viral RNA Mini Handbook 12/2007 for additional details and guidance

Preparation of reagents

A. Addition of carrier RNA to Buffer AVL

NOTE: Buffer AVL–carrier RNA should be prepared fresh, and is stable at 2–8°C for up to

48 hours. This solution develops a precipitate when stored at 2–8°C that must be redissolved by warming at 80°C before use. Do not warm Buffer AVL–carrier RNA solution no more than 6 times. Do not incubate at 80°C for more than 5 minutes. Frequent warming and extended incubation will cause degradation of carrier RNA, leading to reduced recovery of viral RNA and eventually false negative RT-PCR results. This is particularly the case with low-titer samples.

Add 310 µl Buffer AVE to the tube containing 310 µg lyophilized carrier RNA to obtain

a solution of 1 µg/µl. Dissolve the carrier RNA thoroughly, divide it into conveniently sized aliquots, and store it at –20°C. Do not freeze–thaw the aliquots of carrier RNA more than 3 times. Check Buffer AVL for precipitate, and if necessary incubate at 80°C until the precipitate is dissolved. Calculate the volume of Buffer AVL–carrier RNA mix needed per batch of samples by selecting the number of samples to be simultaneously processed from Table 1.

For larger numbers of samples, volumes can be calculated using the following sample calculation: n x 0.56 ml = y ml y ml x 10 µl/ml = z µl


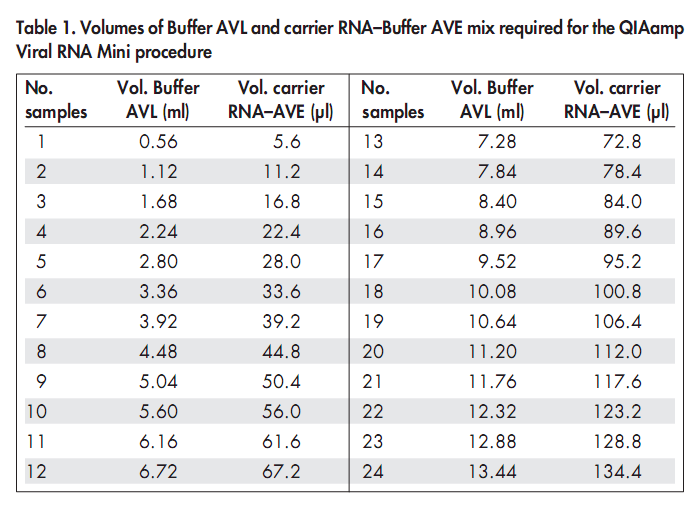


B.Prepare Buffer AW1:

Buffer AW1 is supplied as a concentrate. Before using for the first time, add the appropriate amount of ethanol (96–100%) as indicated on the bottle Buffer AW1 is stable for 1 year when stored closed at room temperature, but only until the kit expiration date.

C. Prepare Buffer AW2

Buffer AW2 is supplied as a concentrate. Before using for the first time, add the appropriate amount of ethanol (96–100%) to Buffer AW2 concentrate as indicated on the bottle.

Extraction Procedure

1. Pipet 560 µl of prepared Buffer AVL containing carrier RNA into a 1.5ml microcentrifuge tube.

2. Add 140 µl fecal suspension supernatant, to the Buffer AVL–carrier RNA in the microcentrifuge tube. Mix by pulse-vortexing for 15 s. To ensure efficient lysis, it is essential that the sample is mixed thoroughly with Buffer AVL to yield a homogeneous solution.

3. Incubate at room temperature (15–25°C) for 10 min.

4. Briefly centrifuge the tube to remove drops from the inside of the lid.

5. Add 560 µl of ethanol (96–100%) to the sample, and mix by pulse-vortexing for 15 s. After mixing, briefly centrifuge the tube to remove drops from inside the lid.

6. Carefully apply 630 µl of the solution from step 5 to the QIAamp Mini column

(in a 2ml collection tube) without wetting the rim. Close the cap, and centrifuge at 6000 x g (8000 rpm) for 1 min. Place the QIAamp Mini column into a clean 2ml collection tube, and discard the tube containing the filtrate. Optional vacuum step is excellent for processing multiple specimens (but need vacuum setup and Qiagen vacuum manifold (catalog No. 19413) is $238.).

NOTE: Close each spin column in order to avoid cross-contamination during centrifugation.

7. Carefully open the QIAamp Mini column, and repeat step 6.

8. Carefully open the QIAamp Mini column, and add 500 µl of Buffer AW1. Close the cap, and centrifuge at 6000 x g (8000 rpm) for 1 min. Place the QIAamp Mini column in a clean 2ml collection tube, and discard the tube containing the filtrate.

9. Carefully open the QIAamp Mini column, and add 500 µl of Buffer AW2. Close

The cap and centrifuge at full speed (20,000 x g; 14,000 rpm) for 3 min. Continue

Directly with step 11, or to eliminate any chance of possible Buffer AW2

Carryover, perform step 10, and then continue with step 11.

10. Recommended: Place the QIAamp Mini column in a new 2ml collection tube, and discard the old collection tube with the filtrate. Centrifuge at full speed for 1 min.

11. Place the QIAamp Mini column in a clean 1.5ml microcentrifuge tube. Discard the old collection tube containing the filtrate. Carefully open the QIAamp Mini column and add 60 µl of Buffer AVE equilibrated to room temperature. Close the cap, and incubate at room temperature for 1 min.

Centrifuge at 6000 x g (8000 rpm) for 1 min.

Extract storage

Viral RNA is stable for up to one year when stored at –20°C or –70°C. [Before storage, samples can be supplemented with equal volume of RNA storage solution (Ambion AM7000 or AM7001) to acquire greater RNA stability; this is recommendation from CDC Norovirus lab)

The extract may be stored at 4°C for 24h before RT-PCR.

The remaining sample and fecal extracts will be stored at -70°C for any further use.

**7. Real time RT PCR for detection of noroviruses**

The primers and probes are as described by Kageyama et al. J.Clin. Microbiol. 2003).

The protocol described in this SOP is for the use of Ag-Path One-Step RT-PCR Kit. This kit includes: 2X RT-PCR Buffer, 25X RT-PCR Mix, Detection Enhancer, and nuclease free water. Sites may make minor adjustments such as primer concentration to optimize this procedure for their site but the primers, probes and AgPath RT PCR kit specified in this SOP must be used by all sites.

Equipment preparation

1. Wipe and clean work surfaces, pipettes, and centrifuges to remove any potential RNase contamination.

2. Turn on the Real time PCR system to allow warm up for 15 min prior to use.

PCR Reagent preparation

1. Keep all reagents on ice during assay set up.

2. Mix the 2X Master Mix by inversion.

3. Vortex all primers for 5 sec.

4. Mix all probes by inversion.

5. Spin tubes with primers and probes for 5 sec and then place on ice.

Master Mix Preparation

Prepare the master mix in a PCR clean area. A No Template Control (NTC) and Positive Control (PC) for all primer and probe sets are included in each run. Determine the number of reactions (N) to set up per assay. Reaction master mixtures are made and dispensed into the 96-well reaction plate or 8-well strips as required. The reaction setup for GI and GII are as follows.

***Genogroup I Noroviruses***

2X RT-PCR Buffer 12.50 μl

25X RT-PCR Mix 1.00 μl

Forward primer – Cog1F (10 μM) 1.00 μl

Reverse primer - Cog1R (10 μM) 1.00 μl

Probe 1 – Ring1a (10 μM) 0.15 μl

Probe 2 – Ring 1b (10 μM) 0.15 μl

Detection Enhancer 1.67 μl

Nuclease-free water 5.53 μl

**Total volume 23.00 μl**

***Genogroup II Noroviruses***

2X RT-PCR Buffer 12.50 μl

25X RT-PCR Mix 1.00 μl

Forward primer – Cog2F (10 μM) 1.00 μl

Reverse primer - Cog2R (10 μM) 1.00 μl

Probe – Ring 2 (10 μM) 0.30 μl

Detection Enhancer 1.67 μl

Nuclease-free water 5.53 μl

**Total volume 23.00 μl**

RNA

Leave the clean area. Add 2 μl of the RNA sample, PC RNA or RNAase free water into wells labeled as sample, PC and NTC respectively. The final reaction volume is 25 μl, cover plate.

Centrifuge the plate at 500 x g for 1 min to remove bubbles or drops that may be present in the wells.

**Cycling Conditions Realtime RT-PCR**

Flourophore: FAM (530nm)

Step Temperature Time

RT 1 cycle 45C 10min

PCR 1 cycle 95C 10min

PCR 40 cycles 95C 15sec

60C 1min

The primer sequences, probes, cycling conditions and amplicon sizes for GI and GII real time PCRs are summarized in Table 2.

**Interpretation:**

1. The NTC reactions for primer/probe sets should not exhibit fluorescence growth curves that cross the threshold line. If, one or more of the primer/probe set NTCs is positive, invalidate the run and repeat the assay. Sample contamination may have occurred.
2. Positive control should result in reaction fluorescence curves (amplification plots) that cross the threshold with all primers and probes. If not, invalidate the run and repeat the assay.
3. A sample is deemed positive when a curve crosses the threshold before or at 40 CT and all controls worked properly.

**Reporting:**

1. Record and report results on Norovirus RT-PCR (NRV) Form

|  | Participant ID | Write the child’s participant ID in the space provided at the upper left corner. |
| --- | --- | --- |
| 01 | Sample ID | Write the 9-digit Sample ID. |
| 02 | Date specimen was received | Enter the date that the specimen was received. |
| 03 | Technician code processing sample | Enter the technician’s unique ID number who processed the sample. |
| 04 | Was Norovirus RT-PCR performed? | Enter yes or no if the Norovirus RT-PCR was performed. If RT-PCR was not performed, STOP completing form. |
| 05 | Technician code performing test | Enter the technician’s unique ID number who performed the Norovirus RT-PCR test. |
| 06 | Date of Norovirus RT-PCR test | Enter the date that the Norovirus RT-PCR test was performed. |
| 07 | Result | Record the result of the RT-PCR test: Negative=00,  Positive for Genogroup I =01, Positive for Genogroup II=02, Positive for Genogroup I and II = 03. |
| 08 | Cycle time to become positive for Genogroup I | Enter the number of cycles to become positive. |
| 09 | Cycle time to become positive for Genogroup II | Enter the number of cycles to become positive. |

**Limitations:**

The presence of excess DNA/RNA template in the specimen may result in false negative results. If a negative result is obtained and high levels of nucleic acid are suspected, the extracted sample may be tested at 2 or more dilutions (e.g., 1:10 and 1:100) to verify the result.

Table 2

**PCR primers, probes and cycling conditions used for GI and GII ORF1/2 region**

| **PCR** | **Cycling Conditions** | **Primer** | **Primer sequence** | **Position** | **Amplicon size** |
| --- | --- | --- | --- | --- | --- |
| GI | 45 ^o^C – 10min  95 ^o^C – 10 min  95 ^o^C – 15 sec  X 40  60 ^o^C – 1 min | COG1F COG1R  RING1 a  RING1 b | 5' cgY tgg atg cgN ttY cat ga 3'  5' CTT AGA CGC CAT CAT CAT TYA C 3'  FAM-AGA TYG CGA TCY CCT GTC CA-TAMRA  FAM-AGA TCG CGG TCT CCT GTC CA-TAMRA | 5291  5375  5340  5340 | 84 bp |
| GII |  | COG2F  COG2R  RING 2 | 5' CAR GAR BCN ATG TTY AGR TGG ATG AG3'  5' TCG ACG CCA TCT TCA TTC ACA3'  FAM-TGG GAG GGC GAT CGC AAT CT-TAMRA | 5003  5100  5048 | 97 bp |

MDF—Microscopy for the Detection of Ova and Parasites in Stool: Microscopy Data Form SOP

**This SOP has been read and understood by:**

| **Name** | **Date** |
| --- | --- |
| **1.** |  | |
| **2.** |  | |
| **3.** |  | |
| **4.** |  | |
| **5.** |  | |
| **6.** |  | |
| **7.** |  | |
| **8.** |  | |
| **9.** |  | |
| **10.** |  | |

**Document History:**

| **Version Number** | **Reason for Changes** | **Date** |
| --- | --- | --- |
|  |  |  |
|  |  |  |
|  |  |  |
|  |  |  |

1. **Purpose**

To detect ova and parasites in stool specimens.

Parasitic organisms can be detected in preparations made from stool samples using light microscopy. The organisms are identified morphologically by examining the shape, size, nuclear and cytoplasmic characteristics of the eggs, cysts, and parasites (trophozoites or worms). Oocysts can be identified using a modified acid fast stain.

Scope: Concentration and microscopic examination of formalin preserved stool by wet prep and modified acid fast stain.

**Quality control:**

1. Atlases, photographs and charts of parasites will be available and used by laboratory microscopists for the confirmation of positives.
2. Positive control slide containing Cryptosporidium oocysts will be stained with each batch of study modified acid fast slides.
3. Digital photographs may be taken of any questionable results and can be reviewed internally or sent to the Desktop for confirmation or resolution.

**External quality assurance:**

1. Five formalin stool concentrates for wet prep microscopy will be sent to site laboratories every 6 months Results will be compared to peer labs. A pass rate of 80% must be maintained.
2. Five methanol fixed slides for modified acid fast staining will be sent to site laboratories every 6 months after study opening. Routine staining and identification procedures will be performed by the bench technologist and results compared to peer labs. A pass rate of 80% must be maintained.
3. Positive modified acid fast slides will be archived and made available for review.

**Internal quality assurance:**

1. At least annually, the bench technologist will re-examine five stored, randomly selected formalin-fixed samples that have the original results blinded. The results of the re-examination will be compared to the original result.

**Safety:**

1. Specimens should be handled, processed and disposed of using standard guidelines for biohazardous materials.
2. Spills should be immediately disinfected with 6% hydrogen peroxide (preferred) or 10% bleach.
3. Lab bench should be disinfected at the completion of every work day

**Specimen**:

Refer to the Stool Specimen Collection and Transport SOP for details.

1. Fecal specimens (1-2 ml or grams) or rectal swabs are collected into sterile empty containers with no additives.
2. Specimens may be preserved in the field in 10% formalin at a ratio of one part stool to three parts formalin preservative if samples cannot be transported to the laboratory within 4 hours of passage.
3. Stool ova and parasites in formalin are preserved and may be tested in batches.
4. The specimens should not be frozen or placed in an incubator because of rapid parasitic degradation.
5. A portion of unpreserved stool will be archive for additional tests. Refer to SOP RSS, “Receipt and Storage of Stool Specimens”.

**Fecal Parasite Concentration**

1. **Purpose**

The concentration method is designed to separate protozoan cysts, oocysts and helminth eggs and larvae from fecal debris by centrifugation and differences in specific gravity. The suspension fluid (formalin and ethyl acetate) is less dense than cysts and eggs and thus they are found in the sediment in the bottom of the tube. Straining and defatting steps (using ethyl acetate to dissolve fecal fats and make the fecal debris float) remove fecal debris and clarify the specimen.

**Specimen:**

1. Stool preserved in 10% formalin at a ratio of one part stool to three parts formalin preservative.

2. Formalin preserved stools may be batch tested.

1. **Materials**

FPC Fecal Parasite Concentration kit (Evergreen Scientific, (800)421-6261)

10% buffered (or unbuffered) Formalin:

Combine and store in a tightly closed bottle (pH ~7.4):

100 ml formaldehyde (37% solution) + 900 ml water

For buffered, add 12g Na_2_HPO_4_ + 3g KH_2_PO_4_

Lugol’s Iodine:

Dissolve 10 g potassium iodide (KI) + 5 g powdered iodine crystals in 100 ml distilled water in a brown bottle (excess iodine crystals should remain on the bottom of the bottle). Store at room temperature for up to 1 year.

Working solution = dilute 1:5 with distilled water (prepare fresh every 10-14 days)

0.85% NaCl

Ethyl acetate

Centrifuge

Light microscope with 10x and 40x objectives

Disposable transfer pipets

Microscope slide & coverslip

**Quality Control:**

1. Check reagents daily, formalin should appear clear, without contamination.
2. Known positive control specimens should be examined quarterly.
3. Microscope should be cleaned and have the ocular micrometer calibrated annually.
4. Centrifuge speed calibration should be performed annually.
5. **Methods:**

Follow Concentration Procedure for manual sedimentation described below or use a commercial concentration kit such as, Evergreen Fecal Parasite Concentrator (FPC) kit.

**Option I – Manual Concentration:**

- - - 1. Line up test tubes in a rack; label tubes with laboratory specimen number.
      2. For specimens received in 10% formalin, resuspend stool/formalin mixture. To obtain the desired end product of 0.5 to 1 ml of sediment, processing 3 ml of the mixture should be sufficient. Strain approximately 3 ml of the formalinized stool mixture through wet gauze into a 15 ml conical tube.
      3. For unpreserved specimens, mix ~ 1 g of stool with 10 ml of 10% formalin, let mixture stand for a minimum of 30 minutes. Strain mixture through wet gauze into a 15 ml conical tube.
      4. Add ~7 ml of 0.85% NaCl (normal saline) to the tube and centrifuge for 10 minutes at 500 x g.
      5. Decant supernatant and resuspend sediment in saline (refill tube with ~14 ml saline, almost to the top of the tube, mix and centrifuge a second time for 10 minutes at 500 x g).
      6. Decant the supernatant and resuspend the sediment in ~10 ml saline and let mixture stand for 10 minutes.
      7. Add 4-5 ml of Ethyl acetate, cover tube and shake vigorously for 30 seconds.
      8. Centrifuge for 10 minutes at 500 x g.
      9. Observe for 4 layers = small sediment (containing parasites), layer of formalin, layer of fecal debris, topped by ethyl acetate.
      10. Ring the fecal plug using an applicator stick and decant all of the supernatant.
      11. While holding tube upside down, remove fluid from walls of the tube with a cotton-tipped swab.
      12. Add 1-2 drops of saline or formalin to resuspend the sediment and place a drop on a slide and cover with a 22x22 coverslip.

**Option II – Evergreen Fecal Parasite Concentrator (FPC) kit:** as per product insert

1. Line up the flat-bottom tubes in a rack; label tubes with laboratory accession number.

2. Place the conical centrifuge tubes (with the green filtration screen attached) in a rack directly behind the flat-bottom tubes.  Label tubes with laboratory accession number.

3. Add the following to the flat-bottom tube in the order specified:

A. 9 ml of 10% formalin

B. Two spoonfuls of formalinized feces (1.0 g)

C. Three drops of Triton X-100

D. 3 ml of ethyl acetate

4. Attach the conical centrifuge tube (with strainer attached with the aerator tube extended 3.5 cm above the top of the green mid-piece screen so that it extends above the fluid level when the tube is inverted.) to each flat bottom tube.

BE SURE TO MATCH THE CORRECT TUBE NUMBERS WITH EACH OTHER.  Tighten each connection to prevent leakage.

5. Shake vigorously for 30 seconds.

6. Flip the tubes over and allow the mixture to filter into the strainer into the conical tube.  If the sample stops flowing, tap the conical tube on the countertop to re-start the transfer.

7. Unscrew the centrifuge tube/strainer assembly and discard.

8. Screw a white plastic cap onto each conical centrifuge tube.

9. Centrifuge conical tubes at 500 x g for 10 minute. Ring the fecal plug using an applicator stick and decant all of the supernatant.

10. While holding tube upside down, remove fluid from walls of the tube with a cotton-tipped swab.

11. Add 1-2 drops of saline or formalin to resuspend the sediment and place a drop on a slide and cover with a 22x22 cover slip.

**Microscopic Examination of Sediment:**

Protozoan cysts and helminth eggs and larvae may be seen and identified. In a heavy infection with *Cryptosporidium*, oocysts may be seen in a direct smear; however, the modified acid-fast stain is normally used to detect these organisms. Oocysts of *Isospora belli* can also be seen in a direct smear. Spores of the microsporidia are too small, and the shape resembles other debris within the stool; therefore, they are not readily visible.

1. Examine the slides using a microscope at 10X and 40X:

a. Systematically scan the entire coverslip with the 10X objective (low power). If something suspicious is seen, turn to the 40X objective for more detailed exam.

- 1. Scan at least one-third of the coverslip with the 40X objective (high dry power), even if nothing suspicious has been seen at 10X.
  2. Consult photographs and refer to parasitology atlas for identification guidance. Study site may email problem or unknown parasite photographs to the microbiology subcommittee for consultation.

2. For enhanced morphological detail, Lugol’s Iodine stain (1:5 dilution) may be used.

**Reporting:**

1. Record results on Microscopy Data Form (MDF). See below for additional guidance.

| # | Question | Guidance |
| --- | --- | --- |
|  | Participant ID | Write the child’s participant ID in the space provided at the upper left corner. |
| 01 | Sample ID | Write the 9-digit Sample ID. |
| 02 | Date specimen was received | Enter the date that the specimen was received. |
| 03 | Technician code | Enter the technician’s unique ID number who processed the sample. |
| 04 | Was wet prep microscopy performed? | Indicate whether or not wet prep microscopy was performed. If no, STOP completing the form. |
| 05 | Were parasites observed? | Indicate yes if parasites were observed and no if parasites were not observed upon wet prep microscopy. If no, continue to question #42. If yes, continue to question #6. |
| 06-39 | Which parasite was observed? | For each parasite listed, indicate whether or not this parasite was observed using either concentrate wet prep and/or direct wet prep. |
| 40-41 | Other parasites observed? | Indicate whether additional parasites not listed were observed using either concentrate wet prep and/or direct wet prep. If additional parasites were observed, complete parasite reporting log. |
| 42 | Technician code | Enter the technician’s unique ID number who performed the wet preps. |
| 43 | Date specimen was tested for parasites | Enter the date that the specimen was tested for parasites. |
| 44-47 | See information contained within the Modified Acid Fast Stain section. | |

**Modified Acid Fast Stain (cold Ziehl-Neelson)**

1. **Purpose**

*Cryptosporidium*, *Cyclospora*, and *Isospora* cause severe diarrhea in immunocompromised persons. They can also cause diarrhea in immunocompetent hosts. The modified Ziehl-Neelsen acid fast staining technique is useful for the identification of oocysts, which may be difficult to detect with other stains such as trichrome.

**Specimen:**

Concentrated sediment of formalin-preserved stool

1. **Materials**

TB Stain Kit ZN (BD, (800)675-0908, cat. # 212520) – containing carbolfuchsin ZN, acid-alcohol and methylene blue solutions

Light Microscope (with 40X and 100X oil immersion objectives)

Microscope slide

**Quality Control:**

Frequency – stain a Control slide (prepared from a specimen containing *Cryptosporidium* oocysts) with each batch stained

Acceptable results - *Cryptosporidium* oocysts 4-6um staining red against a blue background.

1. **Methods**
2. Prepare a smear on a glass slide by placing 1-2 drops of fixed/concentrated stool sediment (note: Do not make the smears too thick, you should be able to see through the material while still wet.)
3. Allow the slide to air dry.
4. Fix the slide by flooding slide with 100% methanol for 1 minute.

(Note: fixed slides are valid indefinitely)

1. Stain the slide using a modified Ziehl-Neelson stain kit:

a. Flood slide with Carbolfuchsin ZN, stain for 1 minute

b. Rinse thoroughly with water.

c. Decolorize by rinsing briefly with acid-alcohol until no more color runs from the slide.

d. Rinse thoroughly with water.

e. Flood slide with methylene blue, stain for 1 minute.

f. Rinse thoroughly with water and air-dry.

(Note: Stained slides are valid indefinitely)

**Examination of Slide:**

Examine the stained sediment form concentrate using 40X (high dry power) and 100X oil immersion:

a. Systematically scan 200-300 fields with the 40X objective. If something suspicious is seen, turn to the 100X objective for more detailed exam.

b. Consult photographs and refer to atlas for identification guidance.

**Interpretation:**

*Cryptosporidium spp.* oocysts are oval or round oocysts, will stain red to pink, measuring 4-6 μm, with a clear internal structure (some of the 4 sporozoites may be visible).

*Isospora sp*. will stain pink to red to deep purple, oval in shape and measuring 10-20 μm (in some immature oocysts the entire oocyst may stain, in mature oocysts the two sporocysts within the oocyst wall usually stain pink to purple with a clear area between the stained sporocysts and the oocyst wall).

*Cyclospora cayetanensis* oocysts resemble *Cryptosporidium* but larger (8-10 μm) and have no definite internal morphology, the acid-fast staining more variable light pink to deep red, some will contain granules or have a bubbly appearance like “wrinkled cellophane” and some oocysts may appear clear or very pale.

Microsporidia spores are acid fast, but identification is very difficult because of their small size (1-2 um).

Study site may send questionable or unknown parasite photographs to the Study Desktop for identification.

**Documentation and Reporting:**

1. Parasitological test results are recorded on Microscopy Data Form (MDF).
2. Completed MDFs are submitted to the Data Management team for double entry into the database.
3. The Lab coordinator reviews lab reports before submission to the Data Management team.

| 44 | Was a modified acid-fast stain performed? | Indicate whether or not a modified acid-fast stain was performed. If the stain was not performed, STOP; form is complete. |
| --- | --- | --- |
| 45 | Did modified acid-fast stain detect an organism? | Indicate whether or not an organism was detected and what organism was detected. |
| 46 | Technician code | Enter the technician’s unique ID number who performed the modified acid-fast stain. |
| 47 | Date | Enter the date (DD/MMM/YY) that the modified acid-fast stain was performed. |

**Archive:**

1. Store the remaining formalized and concentrated stool specimens in labeled boxes at ambient temperature until completion of the study.
2. Store all slides in numbered slide boxes until completion of the study.

**References:**

1. Baily and Scott’s, Diagnostic Microbiology edition, C.V.Mosby Company
2. Laboratory Diagnosis of Cryptosporidiosis, D.P.Case et al, J.Clin.Pathol.38:1337-1341, 1985.
3. Sydney Finegold & William J. Martin: Diagnostic Microbiology. The C.V. Mosby Company, St. Louis
4. Clinical Laboratory Standards Institute CLSI M28A2 Procedure for the Recovery and Identification of Parasites from the Intestinal Tract. Approved Guideline
5. Diagnostic Medical Parasitology L.S Garcia 5^th^ ed 2007

PEF—Detection of Protozoa By ELISA

**This SOP has been read and understood by:**

| **Name** | **Date** |
| --- | --- |
|  |  | |
|  |  | |
|  |  | |
|  |  | |
|  |  | |
|  |  | |
|  |  | |
|  |  | |
|  |  | |
|  |  | |

**Document History:**

| **Version Number** | **Reason for Changes** | **Date** |
| --- | --- | --- |
|  |  |  |
|  |  |  |
|  |  |  |
|  |  |  |

1. **Purpose**

To detect Giardia, Cryptosporidium, and Entamoeba histolytica antigens by enzyme immunoassay.

1. **Materials**

Stored in the refrigerator at 2-8° C:

Entamoeba histolytica II kit (Techlab, Inc.)

Giardia II kit (Techlab, Inc.)

Cryptosporidium II kit (Techlab, Inc.)

Clean screw-capped disposable containers for preparing fecal suspensions

Clean vial for negative control

Clean absorbent paper

Waste container with disinfectant (bleach)

Automated plate washer (optional)

EIA plate reader capable of reading 96 well plate at absorbance of 450nm with reference 620-650nm

1. **Methods**

**Quality control:**

A single positive control and single negative control (diluent) must be tested with every test run.

**Specimen:**

Refer to the Stool Specimen Collection and Transport SOP for details.

1. Fresh or frozen unpreserved stool collected into sterile empty containers with no additives.

2. Samples can be stored at -20° to -70°C and batch tested. It is advisable to test within two months. Frozen fecal specimens are thawed. Repeated freeze-thaw should be avoided.

3. Although the Giardia and Cryptosporidium package insert allows for the testing of formalin preserved specimens, this option will not be routinely used.

4. In case of small specimen quantities refer to SOP QNS “Processing and Testing of QNS Stool Specimens”.

**Procedure:**

1. Open and prepare each kit.

2. Prepare fecal specimens.

**A. Entamoeba histolytica test**

**Specimen Preparation**

For liquid stools, transfer 400μl of specimen in 1.5 ml microcentrifuge.

400μl of diluent is added to the tube (containing stool).

For formed stool, transfer 0.150 to 0.200 g of stool in 1.5 ml microcentrifuge tube and mix thoroughly by vortexing.

**Test Procedure**

1. One drop of conjugate is added to a positive control well, negative control well, and patient sample well.
2. The positive control is shaken for several seconds, and then one drop is added to the positive control well.
3. 100μl of diluents is added to the negative control well.
4. 200μl of vortexed diluted specimen is transferred to a test well of micro assay plate.
5. Cover wells with a plastic adhesive sheet.
6. Incubate for 2 hours at room temperature.
7. Contents of assay wells are shaken out into a discard pan.
8. Each well is washed using a diluted wash solution. The wash solution is supplied as a 20x concentrate, and 50ml of this is added to 950ml of distilled water.
9. Washing steps are repeated for four times, or washing should be continued if any fecal material remains in any well.
10. After washing, the plate is inverted and tapped firmly onto dry paper towel to remove residual liquid in well.
11. 2 drops of substrate are added to each well.
12. Gently tap the wells initially and again at 5 min to mix the substrate.
13. Incubate for 10 minutes at room temperature.
14. Add one drop of stop solution to each well, gently tap to mix, and wait for 2 minutes before reading.
15. Blue color converts to yellow color after mixing of stop solution.
16. The absorbance of color is measured at 450nm wavelength.
17. Reading should be taken within 10 minutes after mixing in stop solution.

**Spectrophotometric Reading:**

- 1. Set the microplate ELISA reader to read at 450 nm. If a dual wavelength reader is used, set the ELISA reader to read at 450 nm and reference at 620 nm.
  2. Determine the absorbance value for the negative control. The negative control reading should be 0.150 or less. If not, the test is not valid and should be repeated, paying attention to the wash procedure.
  3. Subtract the reading of the negative control well from the readings of the positive control well and test wells before interpreting the results (the microplate ELISA reader may be set to blank on the negative control well so this step is performed automatically).
  4. The reading for the positive control should be 0.500 or higher after the negative control reading has been subtracted. If this value is not obtained, the test should be repeated.
  5. A specimen is considered positive for adhesin if the reading is 0.050 or higher after the negative control reading has been subtracted. A specimen is considered negative for the adhesin if the reading is <0.050.
  6. A positive test result indicates that E. histolytica adhesin is present in the fecal specimen. A specimen is considered negative for the adhesin if the reading is <0.050 after the negative control has been subtracted.
  7. Record on ELISA worksheet.

**Interpretation of results:**

| Absorbance (after subtracting Negative Control) | Visual Color | INTERPRETATION |
| --- | --- | --- |
| <0.050 | Clear | Negative - does not contain *E. histolytica* |
| ≥0.050 | Pale yellow to strong yellow | Positive - Specimen contains *E. histolytica* adhesin |

***Visual Interpretation*:**

**Negative:** Any sample that is colorless or resembles the negative control well in intensity of color.

**Positive:** Any sample that is obviously more yellow than the negative control well.

NOTE: The negative control, as well as some test wells, may show some slight yellow color. A sample well must be obviously more yellow than the negative control well to be called a positive result.

**B. Giardia and Cryptosporidium tests**

**Specimen Preparation:**

Frozen fecal specimens should be thawed. Add 400µL of *Diluent* to a microcentrifuge tube (one per sample), then add 100µL of sample to the tube and mix well. If the specimen cannot be pipetted, use an applicator stick to transfer approximately 0.1 gram of feces. This is about the size of a small pea (about 4mm in diameter).

**Methods:**

1. Positive control is shaken for several seconds, then added one drop to a positive control well.

2.100μl of diluents are added to the negative control well.

3.100μl of diluents are transferred to each test well, and then 50μl of diluted specimen is transferred to a test well of micro assay plate by using plastic pipettes.

4. The wells are gently tapped to mix well after sealing with a plastic adhesive sheet.

5. Incubation for 1 hour at room temperature.

6. Contents of assay wells are shaken out into a discard pan.

7. Each well is washed with 1x wash solution in a squirt bottle with a fine-tipped nozzle.

8. Washing steps are repeated for four times or washing should be continued if any fecal material remains in well.

9. After washing the plate is inverted and tapped firmly onto a dry paper towel to remove residual liquid in well.

10. One drop of conjugate is added to each well.

11. Plate is sealed with a plastic adhesive sheet, and gently tapped to mix well.

12. Incubation for 30 min. at room temperature.

13. Steps 8 and 9 are repeated for washing.

14. 2 drops of substrate are added to each well.

15. Gently tap to mix.

16. Incubation for 10 minutes at room temperature.

17. One drop of stop solution is added to each well, gently tap to mix and wait 2 min. before reading.

18. Blue color converts to yellow color after mixing of stop solution.

19. The absorbance of color is measured at 450nm and for dual reader blank against air at 620 nm and read at 450 nm.

20. Reading should be taken within 10 minutes after mixing stop solution.

**Interpretation:**

***Spectrophotometric Interpretation:***

1. Determine the absorbance value of the Negative Control. The Negative Control reading should be <0.150 OD _450_ or < 0.090 OD _450/620_. If not, the test is not valid and should be repeated, paying attention to the wash procedure.

2. The reading for the *Positive Control* should be ≥0.500 or higher.

3. Test results

Negative: < 0.150 (absorbance at 450 nm) or < 0.090 (absorbance at 450/620 nm)

Positive: ≥ 0.150 (absorbance at 450 nm) or ≥ 0.090 (absorbance at 450/620 nm)

| Absorbance (nm) | | Visual Color | Interpretation |
| --- | --- | --- | --- |
| 450 | 450/620 |
| < 0.150 | < 0.09 | Clear to slight yellow | Negative – Below detectable limits of assay |
| ≥ 0.150 | ≥ 0.090 | Pale yellow to strong yellow | Positive Specimen - contains the target antigen |

***Visual Interpretation*:**

**Negative:** Any sample that is colorless or resembles the negative control well in intensity of color.

**Positive:** Any sample that is obviously more yellow than the negative control well.

NOTE: The negative control, as well as some test wells, may show some slight yellow color. A sample well must be obviously more yellow than the negative control well to be called a positive result.

**Reporting:**

1. Record and report the Visual Interpretation and the ODs and OD interpretation on the ELISA Worksheet and report results on the Protozoa ELISA Form (PEF).

| # | Question | Guidance |
| --- | --- | --- |
|  | Participant ID | Write the child’s participant ID in the space provided at the upper left corner. |
| 01 | Sample ID | Write the 9-digit Sample ID. |
| 02 | Date specimen was received | Enter the date that the specimen was received. |
| 03 | Technician code | Enter the technician’s unique ID number who processed the sample. |
| 04 | Was Cryptosporidium ELISA performed? | Enter yes or no if the Cryptosporidium ELISA was performed. If test was not performed, go to question #8. |
| 05 | Technician code | Enter the technician’s unique ID number who performed the Cryptosporidium ELISA test. |
| 06 | Date of Cryptosporidium ELISA test | Enter the date that the Cryptosporidium ELISA test was performed. |
| 07 | Result | Record the result of the Cryptosporidium ELISA test. Refer to the ELISA worksheet, if appropriate. |
| 08 | Was Giardia ELISA performed? | Enter yes or no if the Giardia ELISA was performed. If test was not performed, go to question #12. |
| 09 | Technician code | Enter the technician’s unique ID number who performed the Giardia ELISA test. |
| 10 | Date of Giardia ELISA test | Enter the date that the Giardia ELISA test was performed. |
| 11 | Result | Record the result of the Giardia ELISA test. Refer to the ELISA worksheet, if appropriate. |
| 12 | Was E. histolytica ELISA performed? | Enter yes or no if the E. histolytica ELISA was performed. If test was not performed, form is complete. |
| 13 | Technician code | Enter the technician’s unique ID number who performed the E. histolytica ELISA test. |
| 14 | Date of E. histolytica ELISA test | Enter the date that the E. histolytica ELISA test was performed. |
| 15 | Result | Record the result of the E. histolytica ELISA test. Refer to the ELISA worksheet, if appropriate. |

**References:**

Entamoeba histolytica II kit (Techlab, Inc.)

Giardia II kit (Techlab, Inc.)

Cryptosporidium II kit (Techlab, Inc.)

ALA—Alpha -1- Antitrypsin Assay

**This SOP has been read and understood by:**

| **Name** | **Date** |
| --- | --- |
| **1.** |  | |
| **2.** |  | |
| **3.** |  | |
| **4.** |  | |
| **5.** |  | |
| **6.** |  | |
| **7.** |  | |
| **8.** |  | |
| **9.** |  | |
| **10.** |  | |

**Document History:**

| **Version Number** | **Reason for Changes** | **Date** |
| --- | --- | --- |
| 2 | Clarify dilutions to be run to consistently obtain values on the standard curve | 14-Aug-10 |
| 2 | Dilutions are to be run in duplicate; 2 wells of 1:250 and 2 wells of 1:500 are run | 14-Aug-10 |
| 2 | Use of standards clarified | 14-Aug-10 |
| 2 | AAT reporting form changed | 14-Aug-10 |
| 5 | Dilutions to be run in duplicate; 2 wells of 1:500 are run | 27-Oct-10 |
| 6 | Dilutions to be run singly; 1 well of 1:500 is run | 09-May-11 |
| 6.1 | Added option for 1:10000 dilution | 07-Jul-14 |

**I.** **Purpose**

For the quantitative determination of alpha-1-antitrypsin in stool specimen as an indication of gut inflammation.

**Intended use**

The ELISA Kit is intended for the quantitative determination of alpha-1-antitrypsin in serum and stool. For in vitro diagnostic use only.

**Introduction**

Alpha-1-Antitrypsin is a 52 kD glycoprotein, which is produced by the liver, intestinal macrophages, monocytes and mucous membrane cells of the gut. It belongs to the group of acute phase proteins and is one of the most important proteinase inhibitors. Alpha-1-antitrypsin inhibits, beside others, the proteinases trypsin and the elastase of neutrophils. A lack of α-1-AT leads to an enhanced proteolysis. Only a very small amount of alpha-1-antitrypsin is cleaved or resorbed in the gut. Therefore the measurement of α-1-AT in stool reflects the permeability of the gut during inflammatory processes.

This protocol has been modified significantly from the kit instructions based on MALED data showing that Alpha-anti-trypsin values in Peru and Pakistan are consistently too high to derive valid results from when run at the recommended 1:250 dilution. It is both more efficient and more likely to generate valid results if a further dilution is run instead of the 1:250 dilution on the initial plates (it saves the sample from freeze thaw process which effects the results of the assay).

**Safety**

All reagents of this kit are strictly intended for in vitro diagnostic use only.

This assay was produced and put on the market according to the IVD guidelines of 98/79/EC. Do not interchange kit components from different lots. The stop solution (STOP) contains acid and has to be handled carefully. It is corrosive and causes burns. It should be handled with gloves, eye protection, and appropriate protective clothing in a hood. Any spill should be wiped out immediately with copious quantities of water. Do not breathe vapor and avoid inhalation. In case of an accident or indisposition contact immediately a physician.

The substrate TMB (tetramethyl benzidine) is toxic by ingestion and contact with the skin. Any spill should be wiped out immediately with copious quantities of water. Wear disposable gloves while handling specimens or kit reagents and wash hands thoroughly afterwards. Do not pipette by mouth. Do not eat, drink, smoke or apply makeup in areas where specimens or kit reagents are handled. The reagents of the test kit contain bactericides to protect against bacterial growth. Avoid the contact with the skin or mucous membrane. Reagents should not be used beyond the expiration date shown on kit label. Observe the guidelines for performing quality control in medical laboratories by assaying controls and/or pooled sera. During handling of all kit reagents, controls and serum samples observe the existing legal regulations.

**II. Material**

1. Alpha -1- antitrypsin ELISA form (AEF)
2. BioVendor Research and Diagnostic Products cat # RIC6200R (phone: + 420–549 124 185
3. Other Materials:

• Laboratory balance

• Centrifuge, 3000xg

• Glass or plastic vials

• Various pipettes

• Foil to cover the microtiterplate

• Multichannel or multipipette

• ELISA reader with filter 450 nm (reference filter 620 or 690 nm)

• Microtiterplate shaker

• Vortex mixer

**III. Methods – also refer to Product insert**

Reagent Prep:

1. Microtiterplate (MTP): Take the needed strips out of the bag and mount them on the holder. Please take care that the package has reached room temperature before opening the bag. Strips that are not needed can be stored at 2-8°C. Please dispose of the holder when all strips are used.
2. Washbuffer (WASHBUF): Dilute the washbuffer concentrate 1:10 with deionized (DI) water (1 part buffer + 9 parts DI water.) The dilution is stable for 14 days at 2-8°C.

- Important: When storing the washbuffer concentrate at 2-8°C crystalization could occur. Before dilution, all crystals must be dissolved.
- Note: It is recommended to dilute only the amount of buffer which is used to process the given samples.
- All other test reagents are stable at 2-8 °C, up to the date of expiry stated on the label.

Sample Prep: Note- avoid multiple freeze thaw cycles

Alpha-1-antitrypsin is extracted by the sample dilution buffer out of the stool sample.

1. **100 mg** stool are mixed with **5 ml** WASHBUF on a vortex mixer until the mixture is homogenous.
2. **1 ml** of the mixture is transferred into an Eppendorf vial and centrifuged for **10 min** at 10,000xg.
3. Dilute the supernatant **1:250** with WASHBUF (4 µl + 996 µl WASHBUF) for dilution 1 (1:250).
4. Make dilution 2 (1:500) by taking 120 ul of dilution 1 and 120 ul of WASHBUF.
5. **100 µl** of the **dilution 2** are used per well.

Procedure

All reagents and samples should be at room temperature (18-26°C) and mixed well before use. The position of standards, controls and samples should be noted on a protocol sheet.

1. Take out the needed strips for the microtiter plate and wash **1x** with **250 µl** diluted WASHBUF. Remove residual buffer by tapping the plate on absorbent paper after the washing step.
2. Pipette **100 µl** of all five **STDs (0, 3.3, 10, 30, 90 ng/ml), CTRL** and **dilution 2 (1:500)** of **samples** in the microtiterplate. The dilution 2 of samples is to be runsingly, meaning only one well per sample will be run.
3. The strips are covered and incubated by shaking for **60 min** at room temperature (18-26 °C).
4. The reaction begins with pipetting into the antibody coated microwell. **Pipetting should be done as quickly as possible**. When processing many samples at once, the samples should be pipetted from a separate microtiterplate (150 µl) and transferred simultaneously using a multichannel pipette.
5. Discard the content of the microwells and wash **5x** with **250µl** of diluted WASHBUF. Remove residual buffer by tapping the plate on absorbent paper after the last washing step.
6. Pipette **100 µl** CONJ in each microwell.
7. The strips are covered and incubated by shaking for **60 min** at room temperature (18-26 °C).
8. Discard the content of the microwells and wash **5x** with **250 µl** diluted WASHBUF. Remove residual buffer by tapping the plate on absorbent paper after the last washing step.
9. Pipette **100 µl** SUB in each microwell. Incubate for **10-15 min** at room temperature (18-26 °C) in the dark.
10. Pipette **50 µl** STOP in each microwell, mix well.
11. Read the absorbance at 450 nm. If the microtiterplate reader allows use of a reference wavelength, select 620 or 690 nm as reference wavelength.

**Reading should be done within 5 min after stopping reaction.**

If the highest standard exceeds the range of the reader the reading should be done at 405 nm against 620 nm (690 nm).

**IV. Results**

For the α-1-antitrypsin concentration of fecal samples you must describe the relationship between absorbance at 405 (OD) and the concentration of Alpha-1-antitrypsin in ng/ml by generating a standard curve on each run. This is best done with the 4-parameter algorithm- if not available on your microplate reader a point to point or spline function can be used. Depending on your ELISA plate reader (many have a function that is programmable, ie SoftMaxPro from molecular diagnostics units) this can be done automatically. If not, this requires the generation of a graph like that displayed below in this SOP for each plate.
After generating the standard curve, the measured alpha-1-AT concentration (NOT THE OPTICAL DENSITY) is recorded on the results form along with the dilution (1:500) that yielded the result. In the case that the OD is above the value of the 90 ng/ml standard, report NA on the results form for questions 7,8 or 9 on the report form (do not report a value over 90 ng/ml per dilution). Questions on the report form have been added for the dilutions of 1:1000 and 1:10000. If you obtain a OD on the standard curve (ie below 90 ng/ml) at 1:500 there is no need to run the sample at 1:1000 or 1:10000.

*The curve given above is only for demonstration. It must not be used for calculation of your samples*

**V. Reporting:**

1. Record results on Alpha-1-antitrypsin (ALA) form.

| # | Question | Guidance |
| --- | --- | --- |
|  | Participant ID | Write the child’sparticipant ID in the space provided at the upper left corner. |
| 01 | Sample ID | Write the 9-digit Sample ID. |
| 02 | Date specimen was received | Enter the date that the stool specimen was received. |
| 03 | Technician code processing sample | Enter the technician’s unique ID number who processed the sample. |
| 04 | Was α-1- antitrypsin ELISA performed? | Enter yes or no if the α-1- antitrypsin ELISA was performed. If test was not performed, STOP, form is complete. |
| 05 | Technician code | Enter the technician’s unique ID number who performed the α-1- antitrypsin ELISA test. |
| 06 | Date of α-1- antitrypsin ELISA test | Enter the date that the α-1- antitrypsin ELISA test was performed. |
| 07 | Calculated α-1- antitrypsin concentration value at 1:250 | Enter the α-1- antitrypsin concentration in **ng/ml If the optical density of the sample is greater than the 90 ng/ml standard record NA**  **NOTE:** As of 27-Oct-10 1:250 dilution will no longer be run, so record **NA** |
| 08 | Calculated α-1- antitrypsin concentration value at 1:500 | Enter the α-1- antitrypsin concentration in **ng/ml If the optical density of the sample is greater than the 90 ng/ml standard record NA** |
| 09 | Calculated α-1- antitrypsin concentration value at 1:1000 | Enter the α-1- antitrypsin concentration in **ng/ml If the sample was not run at this concentration because values on the standard curve were obtained at lower dilutions, record NA** |
| 10 | Calculated α-1- antitrypsin concentration value at 1:10000 | Enter the α-1- antitrypsin concentration in **ng/ml If the sample was not run at this concentration because values on the standard curve were obtained at lower dilutions, record NA** |

**VI. Limitation -**

**Stool samples** with α-1-antitrypsin concentrations greater than the highest standard value at a dilution of 1:500 should be further diluted 1:1000 with wash buffer and assayed again. To do this, prepare 400 ul of dilution 2 (1:500) by combining 200 ul of dilution 1 and 200 ul WASHBUFF (as described in Methods, section IIIB step 4). In a separate tube dilute 110 ul of dilution 2 with 110 ul of WASHBUFF to yield dilution 3 (1:1000). To make dilution 4 (1:10000), dilute 110 ul of dilution 3 with 110 of WASHBUFF. Place 100 ul of dilution 3 (1:1000) or dilution 4 (1:10000) in a single well on the plate run. Again, the sample dilution 3 (1:1000) or dilution 4 (1:10000) should be run **singly**.

**VII. Quality Control -**

Control samples should be analyzed with each run. Results, generated from the analysis of control samples, should be analyzed by technicians on the same day of the run. Because of the sensitivity of the assay to temperature and time, it is expected that ODs will vary between laboratories that should develop their own normal range with special attention to the standard with 0 ng/ml and the CTRL sample.

If within the same assay one or more values of the quality control sample are outside of the acceptable limits, then the results for the samples may not be valid. If the duplicates of the same dilution have significantly disparate values, the assay should be rerun.

**Notes:** The local Principal Investigator is notified immediately when an event occurs outside of the standards of practice (this event is called a ***protocol deviation,* which is any unapproved change, deviation, or departure from the study design or procedures). A Protocol Deviation Form is completed by the individual having committed the deviation and the study coordinator/ sponsor/IRBs are notified.**

**VIII. References -**

1. G. Beckmann (Hrsg.). Mikroökologie des Darmes; ISBN 3-87706-521-X;

### MPO—Myeloperoxidase ELISA: In Vitro Determination of Myeloperoxidase in Stool

**This SOP has been read and understood by:**

| **Name** | **Date** |
| --- | --- |
| **1.** |  |
| **2.** |  |
| **3.** |  |
| **4.** |  |
| **5.** |  |
| **6.** |  |
| **7.** |  |
| **8.** |  |
| **9.** |  |
| **10.** |  |

**Document History:**

| **Version Number** | **Reason for Changes** | **Date** |
| --- | --- | --- |
| 2 | Added instructions for completing MPO and MEP forms | 9/APR/12 |
| **3** | Added question (6) about dilution factor to MPO form | 24/Apr/12 |
|  |  |  |
|  |  |  |

**I. Summary and Explanation of the Test**

The granules of neturophils (approx. 70% of the white blood cells) contain a large number of different enzymes. **Myeloperoxidase** (MPO) catalyzes the oxidation of substances through H_2_O_2_. The **MPO** H_2_O_2_-system has a toxic effect on many micro-organisms such as bacteria, fungi, viruses and mycoplasma. The efficiency of the bacteria-destructive Myeloperoxidase H_2_O_2_-system is increased by MN-Elastase. **MPO** determination in the stool reflects the inflammatory activity of Crohn’s disease or ulcerative colitis.

**II. Test Principle**

This Enzyme-Linked-Immuno-Sorbent-Assay (ELISA) is suitable for the quantitative determination of Myeloperoxidase in urine and stool. In a first incubation step, the Myeloperoxidase in the samples is bound to an available excess of antibodies against Myeloperoxidase, which are immobilized to the surface of the microtiter plates. To remove all unbound substances, a washing step is carried out. In a second incubation step, a Peroxidase-labeled antibody against MPO is added. After another washing step, to remove all unbound substances, the solid phase is incubated with the substrate Tetramethylbenzidine (TMB). An acidic stop solution is then added to stop the reaction. The color converts from blue to yellow. The intensity of the yellow color is directly proportional to the concentration of MPO in the sample. A dose response curve of the absorbance unit (optical density, OD) vs. concentration is generated, using results obtained from the calibrators. MPO, present in the patient samples, is determined directly from the curve.

**III. Materials Required but not Supplied**

1. Ultra pure water *
2. Laboratory balance
3. Precision pipettors and disposable tips to deliver 10-1000 µl
4. Foil to cover the microtiter plate
5. Horizontal microtiter plate shaker
6. A multi-channel dispenser or repeating dispenser
7. Centrifuge
8. Vortex-Mixer
9. Standard laboratory glass or plastic vials, cups, etc.
10. Microtiter plate reader at 450 or 405 nm (reference wavelength 620 or 690 nm)
11. MEP Form
12. MPO Form

* Immundiagnostik AG recommend the use of Ultra Pure Water (Water Type 1; ISO 3696), which is free of undissolved and colloidal ions and organic molecules (free of particles > 0.2 µm) with an electrical conductivity of 0.055 µS/cm at 25 °C (≤18.2 MΩ cm).

**IV. Preparation and Storage of Reagents**

1. To run the assay more than once, ensure that reagents are stored at conditions stated on the label. **Prepare only the appropriate amount necessary for each assay.** The kit can be used up to 4 times within the expiry date stated on the label.
2. Reagents with a volume less than **100 µl** should be centrifuged before to avoid loss of volume.
3. The **WASBUF** (wash buffer concentrate) should be diluted with ultra pure water **1:10** before use (100 ml WASHBUF + 900 ml ultra pure water), mix well. Crystals could occur due to high salt concentration in the stock solutions. The crystals must be redissolved at 37 °C in a water bath before dilution. The **WASHBUF** (wash buffer concentrate) is stable at **2-8 °C** until the expiry date stated on the label. Diluted **buffer solution** can be stored in a closed flask **2-8 °C for one month**.
4. The lyophilized **standards (STD) and controls (CTRL)** are stable at **2-8 °C** until the expiry date stated on the label. Reconstitution details are given in the data sheet.
5. The **AB** (detection antibody, biotinylated) must be diluted **1:101** in wash buffer (e.g. 100 µl AB + 10 ml wash buffer). The undiluted AB is stable at **2-8 °C** until the expiry date given on the label. **Diluted antibody solution is not stable and cannot be stored.**
6. The **CONJ** (conjugate, POD-antibody) must be diluted **1:101** in wash buffer (e.g. 100 µl CONJ + 10 ml wash buffer). The undiluted CONJ is stable at **2-8 °C** until the expiry date stated on the label. **Diluted conjugate is not stable and cannot be stored.**
7. All other test reagents are ready to use. Test reagents are stable until the expiry date (see label of test package) when stored at **2-8 °C**.

**V. Specimen Collection and Preparation**

For stool samples:

1. Weigh precisely **0.1g**  of the sample and add **5 ml** of the wash buffer and vortex until sample is mixed thoroughly.
2. Centrifuge the sample suspension for 10 min at 900g. Transfer 1 ml of the supernatant into an Eppendorf tube and centrifuge again at 16,000g for 5 min. The resulting supernatant can be stored at -20 °C for about 1 month.
3. Centrifuge the supernatant at 16,000g for 2 min before use. Dilute the supernatant **1:10** in wash buffer (100 µl supernatant + 900 µl wash buffer). Use **100 µl** of the end-dilution in the assay

**VI. Test Procedure**

Prior to use in the assay allow all reagents and sample to come to room temperature (22 °C) and mix well.

Wash the pre-coated microtiter plate 5x with 250 µl ELISA wash buffer.

1. Pipette **100 µl** of **STD** (standards), **CTRL** (controls) or **samples** into each well.
2. Incubate for **1 hour** at room temperature, shaking on a horizontal mixer.
3. Decant the contents of the plate and wash the wells **5x with 250 µl** of washing buffer solution.
4. Add **100 µl** of diluted **AB** (detection antibody solution).
5. Incubate for **1 hour** at room temperature, shaking on a horizontal mixer.
6. Decant the contents of the plate and wash the wells **5x with 250 µl** of washing buffer solution.
7. Add **100 µl** of diluted **CONJ** (conjugate solution).
8. Incubate for **1 hour** at room temperature, shaking on a horizontal mixer.
9. Decant the contents of the plate and wash the wells **5x with 250 µl** of washing buffer solution.
10. Add **100 µl** of **SUB** (TMB-substrate) solution.
11. Incubate approx. **10-20 minutes** at room temperature, shaking delicately (be sure not to agitate so vigorously that wells overflow or bubbles are formed ) once at the beginning middle and end of the incubation period (ie at least 3 times in total) until sufficient coloring is achieved.
12. Add **50 µl** of **STOP** (stop solution) and mix briefly.
13. Determine **absorption immediately** with an ELISA reader at **450 nm** against 620 nm (or 690 nm) as a reference. If no reference wavelength is available, read only at 450 nm. If the extinction of the highest standard exceeds the measurement range of the photometer, absorption must be measured immediately at 405 nm against 620 nm (or 690 nm) as reference.

**VII. Results**

A calibration curve is constructed from the calibrator values and the results of the samples are read from it. Commercially available software can be used as well as graph paper for evaluation.

The calibration curve **is not linear**, therefore a spline- or 4PL-algorithm is recommended.

**If results obtained are not on the standard curve, the sample should be diluted further and run again.**

For stool samples:

The concentrations of the samples must be read directly from the standard curve. Results are to be reported as concentrations at a given dilution, NOT optic densities.

*The concentration read from the calibration curve must be multiplied by* ***500 (or the final dilution factor used)*** *to obtain the MPO concentration of the sample. This value should be reported on the MPO form.* Sample calculations:

Step 1. 0.1g of stool+5mL wash buffer. (Dilution factor 1:50).

Step 3. 100ul supernatant + 900ul wash buffer (Dilution factor 1:10).

**Total dilution factor**: 1/50 *1/10 =1:500

**Final result**: Concentration obtained from standard curve * dilution factor

eg. 11.8703 * 500 = 5935.15ng/ml

Examples of other dilutions:

Step 1. 0.1g of stool+5mL wash buffer. (Dilution factor 1:50).

Step 3. 50ul supernatant + 950ul wash buffer (Dilution factor 1:20).

Total dilution factor: 1/50 *1/20 =1:1000

Step 1. 0.1g of stool+5mL wash buffer. (Dilution factor 1:50).

Step 3. 100ul supernatant + 400ul wash buffer (Dilution factor 1:5).

Total dilution factor: 1/50 *1/5 =1:250

**VIII. Reporting:**

1. Record ELISA plate run information on the MEP form—complete 1 MEP form per plate.

| **#** | **Question** | **Guidance** |
| --- | --- | --- |
| 01 | Plate ID | Write the 13-digit Plate ID. The ID consists of the plate run number (eg 1^st^ plate run of that day, 2^nd^ plate run that day etc.) + the type of plate (MPO) + the date (DDMMMYY) + the 2 letter Country_ID. |
| 02 | Date of MPO assay | Enter the date that the MPO assay was run. |
| 03 | Time of MPO assay | Enter the time of the MPO assay (24 hour scale HH:MM) |
| 04 | Technician code processing sample | Enter the technician’s unique ID number who processed the sample. |
| **Information for standards (On the page that derives standard curve)** | | |
| 05 | Kit lot # for Standards | Enter the lot number of the Standards included with the MPO assay kit |
| 06 | Vender reported OD for Standard 1 | Enter the OD for Standard 1 reported in the manufacturer’s materials with the MPO assay kit. Standard 1 has the lowest OD. |
| 07 | Vender reported OD for Standard 2 | Enter the OD for Standard 2 reported in the manufacturer’s materials with the MPO assay kit. Standard 2 has the 2^nd^ lowest OD. |
| 08 | Vender reported OD for Standard 3 | Enter the OD for Standard 3 reported in the manufacturer’s materials with the MPO assay kit. Standard 3 has the middle OD. |
| 09 | Vender reported OD for Standard 4 | Enter the OD for Standard 4 reported in the manufacturer’s materials with the MPO assay kit. Standard 4 has the 2^nd^ highest OD. |
| 10 | Vender reported OD for Standard 5 | Enter the OD for Standard 5 reported in the manufacturer’s materials with the MPO assay kit. Standard 5 has the highest OD. |
| 11 | Concentration for Standard 1 | Enter the Concentration for Standard 1 reported in the manufacturer’s materials with the MPO assay kit. Standard 1 has the lowest concentration. |
| 12 | Concentration for Standard 2 | Enter the Concentration for Standard 2 reported in the manufacturer’s materials with the MPO assay kit. Standard 2 has the 2^nd^ lowest concentration. |
| 13 | Concentration for Standard 3 | Enter the Concentration for Standard 3 reported in the manufacturer’s materials with the MPO assay kit. Standard 3 has the middle concentration. |
| 14 | Concentration for Standard 4 | Enter the Concentration for Standard 4 reported in the manufacturer’s materials with the MPO assay kit. Standard 4 has the 2^nd^ highest OD. |
| 15 | Concentration for Standard 5 | Enter the Concentration for Standard 5 reported in the manufacturer’s materials with the MPO assay kit. Standard 5 has the highest concentration. |
| **Information for controls (On the product specification page)** | | |
| 16 | Kit lot for Controls | Enter the lot number of the Controls included with the MPO assay kit |
| 17 | Concentration for  Control 1 | Enter the Concentration for Control 1 reported in the manufacturer’s materials with the MPO assay kit. |
| 18 | Concentration for  Control 2 | Enter the Concentration for Control 2 reported in the manufacturer’s materials with the MPO assay kit. |
| **OD values and results obtained for controls (generated by MAL-ED lab)** | | |
| 19 | OD for Control 1 | Enter the OD for Control 1 obtained in the lab. |
| 20 | Result for Control 1 | Enter the calculated result for Control 1 obtained in the lab. |
| 21 | OD for Control 2 | Enter the OD for Control 2 obtained in the lab. |
| 22 | Result for Control 2 | Enter the calculated result for Control 2 obtained in the lab. |
| **OD values for standards (generated by MAL-ED lab)** | | |
| 23 | OD for Standard 1 | Enter the OD for Standard 1 obtained in the lab. |
| 24 | OD for Standard 2 | Enter the OD for Standard 2 obtained in the lab. |
| 25 | OD for Standard 3 | Enter the OD for Standard 3 obtained in the lab. |
| 26 | OD for Standard 4 | Enter the OD for Standard 4 obtained in the lab. |
| 27 | OD for Standard 5 | Enter the OD for Standard 5 obtained in the lab. |
| 28 | Observations | Record any observations. |

1. Record MPO sample results on the MPO form--complete 1 MPO form per sample.

| **#** | **Question** | **Guidance** |
| --- | --- | --- |
|  | Participant ID | Write the child’sparticipant ID in the space provided at the upper left corner. |
| 01 | Sample ID | Write the 9-digit Sample ID. |
| 02 | Date specimen was received | Enter the date that the stool specimen was received. |
| 03 | Technician code processing sample | Enter the technician’s unique ID number who processed the sample. |
| 04 | Was Myeloperoxidase ELISA performed? | Enter yes or no if the myeloperoxidase ELISA was performed. If test was not performed, STOP, form is complete. |
| 05 | Date of Myeloperoxidase ELISA test | Enter the date that the myeloperoxidase ELISA test was performed. |
| 06 | Final dilution | Enter 01 for 1:250 dilution; 02 for 1:500 dilution; 03 for 1:1000 dilution; 04 for other (if other, report the actual dilution in the Observations section). |
| 07 | Optical density | Enter the OD value obtained for the specimen. |
| 08 | Results (ng/ml) | Enter the **final** myeloperoxidase concentration in ng/ml. (Multiply the concentration obtained from the standard curve by the final dilution) |
| 09 | Plate ID | Write the 13-digit Plate ID. The ID consists of the plate run number (eg 1^st^ plate run of that day, 2^nd^ plate run that day etc.) + the type of plate (MPO) + the date (DDMMMYY) + the 2 letter Country_ID. |
| 10 | Observations | Record any observations. |

**IX. Limitations**

Samples with Myeloperoxidase levels greater than the highest calibrator should be further diluted and re-assayed.

**X. Quality Control**

Control samples should be analyzed with each run. Results, generated from the analysis of control samples, should be evaluated for acceptability using appropriate statistical methods. The results from the patient samples may not be valid, if within the same assay one or more values of the quality control sample are outside the acceptable limits.

### NEO—Neopterin ELISA: Quantitative Determination of Neopterin in Stool

**This SOP has been read and understood by:**

| **Name** | **Date** |
| --- | --- |
| **1.** |  |
| **2.** |  |
| **3.** |  |
| **4.** |  |
| **5.** |  |
| **6.** |  |
| **7.** |  |
| **8.** |  |
| **9.** |  |
| **10.** |  |

**Document History:**

| **Version Number** | **Reason for Changes** | **Date** |
| --- | --- | --- |
| 2 | Low values at extreme of standard curve. Change final dilution from 1:1000 to 1:500 | 12-OCT-2011 |
| 3 | Added instructions for completing the NEP and NEO forms. | 9/APR/12 |
|  |  |  |
|  |  |  |
|  |  |  |

**I. Intended Use**

Enzyme immunoassay for the *in-vitro-diagnostic* quantitative determination of neopterin in human urine and sera. This protocol has been adapted for stool.

**II. Summary and Explanation**

Neopterin biosynthesis is closely associated with activation of the cellular immune system. Increased concentrations of neopterin were reported in patients with viral infections, suggesting that increased values may originate from the immune response of patients directed against virally infected cells. It was shown that antigenic stimulation of human peripheral blood mononuclear cells leads to neopterin release into cell culture medium and that human macrophages produce neopterin in vitro when stimulated by interferon gamma.

**III. Test Principle**

Solid phase enzyme-linked immunosorbent assay (ELISA) based on the basic principle of a competitive ELISA. An unknown amount of antigen in the sample and a fixed amount of enzyme labeled antigen compete for the antibody-binding sites (rabbit-anti-neopterin). Both antigen-antibody complexes bind to the wells of the microtiter strips coated with a goat-anti-rabbit antibody. Unbound antigen is removed by washing. The intensity of the color developed after the substrate incubation is inversely proportional to the amount of antigen in the sample. Results of samples can be determined directly using the standard curve.

**IV. Materials Required but not Supplied**

1. Micropipettes (Multipette Eppendorf or similar devices, < 3% CV). Volume: 10; 50; 100; 1000 µL
2. Vortex mixer with adapter for multiple samples (See <http://www.usascientific.com/productimages/74043010_300.jpg> for an example)
3. Orbital shaker (500 rpm)
4. 8-Channel Micropipettor with reagent reservoirs
5. Wash bottle, automated or semi-automated microtiter plate washing system
6. Microtiter plate reader capable of reading absorbance at 450 nm (reference wavelength 600-650 nm)
7. Bidistilled or deionised water
8. Paper towels, pipette tips and timer
9. NaCl 0.9%
10. NEO Form
11. NEP Form

**V. Procedure Notes**

1. Any improper handling of samples or modification may influence the results. The indicated pipetting volumes, incubation times, temperatures, and pretreatment steps have to be performed strictly according to the instructions. Use calibrated pipettes and devices only.
2. Once the test has been started, all steps should be completed without interruption. Make sure that required reagents, materials and devices are prepared ready at the appropriate time. Allow all reagents and specimens to reach room temperature (18-25 °C) and gently swirl each vial of liquid reagent and sample before use. Mix reagents without foaming.
3. Avoid contamination of reagents, pipettes and wells/tubes. Use new disposable plastic pipette tips for each component and specimen. Do not interchange caps. Always cap not used vials. Do not reuse wells/tubes or reagents.
4. Some components contain ≤ 250 µL solution. Take care that the solution is completely on the bottom of the vial before opening.
5. Incubation time affects results. All wells should be handled in the same order and time sequences. It is recommended to use an 8-channel Micropipettor for pipetting of solutions in all wells.
6. Microplate washing is important. Improperly washed wells will give erroneous results. It is recommended to use a multichannel pipette or an automatic microplate washing system. Do not allow the wells to dry between incubations. Do not scratch coated wells during rinsing and aspiration. Rinse and fill all reagents with care. While rinsing, check that all wells are filled precisely with Wash Buffer, and that there are no residues in the wells.
7. Humidity affects the coated wells/tubes. Do not open the pouch until it reaches room temperature. Unused wells/tubes should be returned immediately to the resealed pouch including the desiccant.

**VI. Procedure**

1. Weigh 0.1 g of stool and dilute sample in 0.5 ml NaCl (dilution 1:5).
2. Vortex 30 minutes (using an adapter for multiple samples).
3. Centrifuge at 1200g for 20 minutes.
4. Remove **10µL** of supernatant and dilute in **990 µL** of NaCl (dilution of 1:100 bringing final dilution to 1:500)-- protect from light by pre-wrapping tubes with aluminum foil or doing this dilution in an amber tube.
5. Vortex for one minute.
6. Pipette **10 µL** of each **Standard, Control, and diluted stool sample** into the respective wells of the Microtiter plate.
7. Pipette **100 µL** of freshly prepared **Enzyme Conjugate (1:201)** into each well.
8. Pipette **50 µL** of **Neopterin Antiserum** into each well.
9. Cover plate with black adhesive foil. **Incubate 90 minutes** at **RT (18-25 °C)** on an orbital shaker (500 rpm) in the dark.
10. Remove adhesive foil. Discard incubation solution. Wash plate **4 x** with **300 µL** diluted **Wash Buffer**. Remove excess solution by tapping the inverted plate on a paper towel.
11. For adding of Substrate and Stop Solution use, if available, an 8-channel Micropipettor. Pipetting should be carried out in the same time intervals for Substrate and Stop Solution. Use positive displacement and avoid formation of air bubbles.
12. Pipette **150 µL** of **TMB Substrate Solution** into each well.
13. **Incubate 10 minutes** at **RT (18-25 °C)**.
14. Stop the substrate reaction by adding **150 µL** of **TMB Stop Solution** into each well. Briefly mix contents by gently shaking the plate.
15. **Measure** optical density with a photometer at **450 nm** (Reference wavelength: 600-650 nm) within **15 minutes**.

**VII. Calculation of Results**

The obtained OD of the standards (y-axis, linear) are plotted against their concentration (x-axis, logarithmic) either on semi-logarithmic graph paper or using an automated method. A good fit is provided with cubic spline, 4 Parameter Logistics or Logit-Log.

For the calculation of the standard curve, apply each signal of the standard (one obvious outlier of duplicated might be omitted and the more plausible single value might be used).

The concentrations of the samples must be read directly from the standard curve. Results are to be reported as concentrations (in nmol/L) at a given dilution, NOT optic densities.

***Samples showing concentrations above the highest standard have to be diluted and re-assayed.***

*Note: Due to the dilution of stool samples the stool values obtained have to be multiplied by* ***500 (or the final dilution factor used)*** *to obtain the final Neopterin concentration of the sample. This value should be reported on the NEO form.*

Sample calculations

Step 1. 0.1g of stool+0.5mL NaCl. (Dilution factor 1:5).

Step 3. 10ul supernatant + 990ul NaCl (Dilution factor 1:100).

**Total dilution factor**: 1/5 *1/100 =1:500

**Final result**: Concentration obtained from standard curve * dilution factor

eg. 1.3433* 500 = 671.65nmol/L

Examples of other dilutions:

Step 1. 0.1g of stool+5mL NaCl. (Dilution factor 1:5).

Step 3. 5ul supernatant + 995ul NaCl (Dilution factor 1:200).

Total dilution factor: 1/5 *1/200 =1:1000

Step 1. 0.1g of stool+5mL NaCl. (Dilution factor 1:5).

Step 3. 10ul supernatant + 490ul NaCl (Dilution factor 1:50).

Total dilution factor: 1/5 *1/50 =1:250

For your own site analyses (**not for MAL-ED**)**,** in order to convert to ng/ml:

multiply the final MAL-ED result (in nmol/L) by 0.253 in order to get Neopterin in ng/ml. This is only for site use and is not to be entered in the MAL-ED database.

*Conversion:*

*Neopterin (nmol/L) x 0.253 = ng/mL*

**VIII. Reporting:**

1. Record ELISA plate run information on the NEP form—complete 1 NEP form per plate.

| **#** | **Question** | **Guidance** |
| --- | --- | --- |
| 01 | Plate ID | Write the 13-digit Plate ID. The ID consists of the plate run number (eg 1^st^ plate run of that day, 2^nd^ plate run that day etc.) + the type of plate (NEO) + the date (DDMMMYY) + the 2 letter Country_ID. |
| 02 | Date of NEO assay | Enter the date that the NEO assay was run. |
| 03 | Time of NEO assay | Enter the time of the NEO assay (24 hour scale HH:MM) |
| 04 | Technician code processing sample | Enter the technician’s unique ID number who processed the sample. |
| **Information for standards (obtained from Quality Control Certificate)** | | |
| 05 | Kit lot # for Standards | Enter the lot number of the Standards included with the NEO assay kit |
| 06 | Vender reported OD for Standard A | Enter the OD for Standard A reported in the manufacturer’s materials with the NEO assay kit. |
| 07 | Vender reported OD for Standard B | Enter the OD for Standard B reported in the manufacturer’s materials with the NEO assay kit. |
| 08 | Vender reported OD for Standard C | Enter the OD for Standard C reported in the manufacturer’s materials with the NEO assay kit. |
| 09 | Vender reported OD for Standard D | Enter the OD for Standard D reported in the manufacturer’s materials with the NEO assay kit. |
| 10 | Vender reported OD for Standard E | Enter the OD for Standard E reported in the manufacturer’s materials with the NEO assay kit. |
| 11 | Vender reported OD for Standard F | Enter the OD for Standard F reported in the manufacturer’s materials with the NEO assay kit. |
| 12 | Concentration for Standard A | Enter the Concentration for Standard A reported in the manufacturer’s materials with the NEO assay kit. |
| 13 | Concentration for Standard B | Enter the Concentration for Standard B reported in the manufacturer’s materials with the NEO assay kit |
| 14 | Concentration for Standard C | Enter the Concentration for Standard C reported in the manufacturer’s materials with the NEO assay kit. |
| 15 | Concentration for Standard D | Enter the Concentration for Standard D reported in the manufacturer’s materials with the NEO assay kit |
| 16 | Concentration for Standard E | Enter the Concentration for Standard E reported in the manufacturer’s materials with the NEO assay kit. |
| 17 | Concentration for Standard F | Enter the Concentration for Standard F reported in the manufacturer’s materials with the NEO assay kit. |
| **Information for controls (obtained from Quality Control Certificate)** | | |
| 18 | Concentration for  Control 1 | Enter the Concentration for Control 1 reported in the manufacturer’s materials with the NEO assay kit. |
| 19 | Concentration for  Control 2 | Enter the Concentration for Control 2 reported in the manufacturer’s materials with the NEO assay kit. |
| **OD values and results obtained for controls (generated by MAL-ED lab)** | | |
| 20 | OD for Control 1 | Enter the OD for Control 1 obtained in the lab. |
| 21 | Result for Control 1 | Enter the calculated result for Control 1 obtained in the lab. |
| 22 | OD for Control 2 | Enter the OD for Control 2 obtained in the lab. |
| 23 | Result for Control 2 | Enter the calculated result for Control 2 obtained in the lab. |
| **OD values for standards (generated by MAL-ED lab)** | | |
| 24 | OD for Standard A | Enter the OD for Standard A obtained in the lab. |
| 25 | OD for Standard B | Enter the OD for Standard B obtained in the lab. |
| 26 | OD for Standard C | Enter the OD for Standard C obtained in the lab. |
| 27 | OD for Standard D | Enter the OD for Standard D obtained in the lab. |
| 28 | OD for Standard E | Enter the OD for Standard E obtained in the lab. |
| 29 | OD for Standard F | Enter the OD for Standard F obtained in the lab. |
| 28 | Observations | Record any observations. |

1. Record Neopterin sample results on the NEO form—Complete 1 NEO form per sample.

| **#** | **Question** | **Guidance** |
| --- | --- | --- |
|  | Participant ID | Write the child’sparticipant ID in the space provided at the upper left corner. |
| 01 | Sample ID | Write the 9-digit Sample ID. |
| 02 | Date specimen was received | Enter the date that the stool specimen was received. |
| 03 | Technician code processing sample | Enter the technician’s unique ID number who processed the sample. |
| 04 | Was Neopterin ELISA performed? | Enter yes or no if the neopterin ELISA was performed. If test was not performed, STOP, form is complete. |
| 05 | Date of Neopterin ELISA test | Enter the date that the neopterin ELISA test was performed. |
| 06 | Final dilution | Enter 01 for 1:250 dilution; 02 for 1:500 dilution; 03 for 1:1000 dilution; 04 for 1:125 dilution; 05 for 1:200 dilution; 06 for Other (if other, report the actual dilution in the Observations section). |
| 07 | Optical density | Enter the OD value obtained for the specimen. |
| 08 | Results (nmol/L) | Enter the **final** neopterin concentration in nmol/L. (Multiply the concentration obtained from the standard curve by the final dilution) |
| 09 | Plate ID | Write the 13-digit Plate ID. The ID consists of the plate run number (eg 1^st^ plate run of that day, 2^nd^ plate run that day etc.) + the type of plate (NEO) + the date (DDMMMYY) + the 2 letter Country_ID. |
| 10 | Observations | Record any observations. |

### QCB—Quality Control: Bacterial Growth Media

**PURPOSE:** To assure the quality of prepared media and maintain its ability to support the growth of bacteria for which it is intended.

Media prepared in house is tested for:

1. Sterility, if it is autoclaved or filtered during preparation.
2. Ability to support growth, using at least one organism to demonstrate the ability of the media to support growth
3. Selectivity and/or inhibition, using at least one organism to confirm its selective characteristic, and at least one organism to confirm its inhibitory characteristic
4. Biochemical response, using at least one organism which will produce the expected reaction (positive control) and with at least one organism which will not produce the expected reaction (negative control). Commercially prepared media is performance tested by the manufacturer and requires only visual inspection and a sterility check.

All reagents are tested to make sure the expected reactions are obtained.

Antisera should be checked for reactivity upon receipt.

Document the date of preparation or receipt and quality control test results.

Refer to Table 1 for control strains and frequency requirements.

**A. Sterility Check**

1. Incubate one tube or plate from each autoclaved or filter-sterilized batch of media 35 C for 48 hours.
2. Document acceptable sterility on MEDIA LOG.
3. If any are contaminated, incubate 10% of the shipment.
4. Complete a QC DEVIATION FORM if any are not sterile.
5. Discard all contaminated media.
6. Arrange for replacement of contaminated media.

**B. Ability to support growth of the target organisms**

Use at least one strain to test for ability of the media to support growth of the target pathogen.

1. Inoculate the control strain to nonselective broth (e.g.TSB]) and incubate overnight.
2. Prepare a standardized inoculum for testing the medium by making a 1:100 dilution of the broth culture.
3. Inoculate test media, incubate, and examine growth.
4. Document whether the strain produces the appropriate reactions

**C. Confirmation of, selective growth and differential biochemical reactions**

For selective media use at least one organism that will grow on the medium and at least one organism that will not grow on the selective medium to test for the medium’s ability to differentiate target organisms from competitors. If the medium is both selective and differential, include two organisms that will grow on the medium and produce different reactions (e.g.,for MacConkey agar: a lactose-nonfermenting organism such as S. flexneri; a lactose-fermenting organism such as E. coli; and, S. aureus, which should not grow).

1. Inoculate the control strains to nonselective broth (e.g.TSB]) and incubate overnight.
2. Prepare a standardized inoculum for testing the medium by making a 1:10 dilution of the broth culture.
3. Inoculate test media, incubate, and examine growth and reactions.
4. Document whether the strains produce the appropriate reactions

**D. Biochemicals and reagents :**

Use at least one organism that will produce a positive reaction and at least one organism that will produce a negative reaction (e.g., or urea medium, a urease-positive organism such as Proteus and a urease- negative organism such as E. coli).

1. Pick 18-24 hour colony growth from control strains.
2. Inoculate test media, incubate, examine for biochemical reaction
3. Document whether the strains produce the appropriate reactions

**E. Antisera** QC antisera upon receipt to ensure that no deterioration has occurred.

TABLE 1 Acceptable Organisms for Performance Quality Control

| Media/Reagent | Positive Controls | Negative Control | Frequency |
| --- | --- | --- | --- |
| Cary Blair Transport | S.flexneri ,C.jejuni | N.A. | Each Batch |
| Catalase | S.aureus | Streptococcus sp | Each day of use |
| Gram Stain | E.coli | S.aureus | Weekly |
| Hipurate Hydrolysis | C.jejuni IV | C.coli I | Each Batch |
| Indole (spot) | E.coli | K.pneumoniae | Each day of use |
| Kligler Iron Agar Slants | Salmonella : K/A H2S | E.coli A/A, gas | Each Batch |
| Lysine Decarboxylase | K.pneumoniae | P.mirabilis | Each Batch |
| MacConkey agar | E. coli ,LF pink  Salmonella sp NLF Colorless | S.aureus inhibited | Each Batch |
| MIO Motility  Indole  Ornithine | E.coli  E.coli  E.coli | K..pneumoniae  K.pneumoniae  P.mirabilis | Each Batch |
| Nitrate | Campylobacter |  | Each Batch |
| Oxidase | Aeromonas sp | E.coli | Each day of use |
| TCBS | *Vibrio cholerae ,*yellow | E.coli ,inhibited | Each Batch |
| Toluidine Blue-DNA agar | C.jejuni IV | C.coli I | Each Batch |
| Urea | Proteus sp | E.coli | Each Batch |
| Xylose-Lysine-Deoxycholate | E. coli ,LF yellow  Salmonella sp , Black H2S | S.aureus ,inhibited | Each Batch |
| Rapid H2S | C.jejuni IV | C.coli I | Each Batch |
| Antisera | Each Target serotype, 3+ | No agglutination | Upon receipt, then monthly |
| API 20E | Refer to API SOP | Refer to API SOP | Upon receipt |

###

### QCM—Quality Control/Quality Assurance: Microbiology

**PURPOSE**

To address central questions of the MAL-ED project, we need accurate, comparable, and complete microbiology data. Also, we must ensure that all sites adhere to the harmonized protocols. The Microbiology QC/QA activities are designed to support the site labs and verify data quality but not impose an excessive financial or unnecessary time burden on the Mal-ED Consortium.

1. Quality Assurance Site Visit

The microbiology procedures are mostly routine and easily implemented with minimal training at the study sites. The goal of the QA site visit is to confirm and document the capacity of each site to adhere to the standardized protocols and deliver reliable microbiology data.

The QA site visit will include:

- Review of the microbiology SOPs and data forms
- Specimen Dry Run (collection or receipt to archive)
- Identification and resolution of any site specific obstacles to compliance
- Site specific training assistance if needed
- IATA specimen shipping certification

2. Laboratory Proficiency: QC/QA

QC- Quality Control

Each Microbiology SOP specifies QC measures to ensure that all kits and reagents are working to their design /operational specifications.

EQA- External Quality Assurance

Site labs will receive blinded samples for testing two times per year. The results will be compared with peer labs. An acceptable pass rate is 80%. However, all discrepant results will be investigated and corrective action taken. A lab experiencing reoccurring problems will receive retraining.

The following will be sent *twice a year*.

- Five formalin stool concentrates for wet prep microscopy
- Five methanol fixed slides for modified acid fast staining
- Five *E.coli* isolates or DNA extracts for Identification of Diarrheagenic *E. coli* by Multiplex PCR
- Five blinded bacteria isolates for identification
- Five undiluted stool specimens, (that have been well characterized for Norovirus) will be sent to participating site labs for Norovirus PCR testing

IQA -Internal Quality Assurance

IQA includes annual competency assessment of staff, continuing education, investigation of internal discrepant results, etc. Some MAL-ED Consortium sites have well developed Quality Management Plans in place. These sites may share tools with the other site labs on the Central Desktop. Sharing resources (e.g., training check sheets, annual competency tests, etc) will help build the capacity of the MAL-ED consortium.

**Collection and Processing of Clinical Specimens**

**Urine**

UCF—Urine Collection, Processing, and Transport

NOTE: This SOP describes urine collection when administering the Lactulose Mannitol Solution (3, 6, 9, 15 months). Sites conducting the lactulose mannitol assay at 24 months (SA & TZ) should also use this UCF SOP.

Instructions for collecting small urine volumes (without administering LM solution first) at months 24, 36 and BMMI-only timepoints (12, 18, 21m) can be found in the Small Urine Collection SOP that follows).

**Urine Collection Schedule**

|  |  | 3m | 6m | 9m | 12m | 15m | 18m | 21m | 24m | 36m |
| --- | --- | --- | --- | --- | --- | --- | --- | --- | --- | --- |
| MAL-ED only child | Urine Collected | Yes | Yes | Yes | No | Yes | No | No | Yes | Yes |
|  | LM administered? | Yes | Yes | Yes |  | Yes |  |  | TZ & SA only | No |
|  | Volume of chlorhexidine-free urine collected | 2ml | 2ml | 2ml |  | 2ml |  |  | 2ml | 2ml |
|  | Form used | UCF | UCF | UCF |  | UCF |  |  | USV | USV |
| MAL-ED + BMMI child | Urine Collected | Yes | Yes | Yes | Yes | Yes | Yes | Yes | Yes | (for MAL- ED only) |
|  | LM administered? | Yes | Yes | Yes | No | Yes | No | No | No | No |
|  | Volume of chlorhexidine-free urine collected | 4ml | 4ml | 4ml | 2ml | 4ml | 2ml | 2ml | 4ml | 2ml |
|  | Form used | UCF | UCF | UCF | USV | UCF | USV | USV | USV | USV |

1. **Purpose**

Urine specimens will be collected during the monthly surveillance visits at 3, 6, 9, and 15 months of age and will be used to assess the ratio of lactulose mannitol excretion and iodine concentration (only at 6 and 15 months). Iodine is measured as part of the micronutrient assessment; the lactulose:mannitol (LM) test is considered a consistent and sensitive method to measure small intestinal epithelial area, paracellular and transcellular transport, damage and permeability. Children will be fasted for **at least 2 hours**) before ingesting a solution containing lactulose (250 mg/ml) and mannitol (50 mg/ml) in a dose of 2 ml/Kg of weight or a maximum 20 ml. The children are requested to empty their bladders whenever possible while fasting and before ingesting the test sugar solution. The children are allowed to return to their regular diet **30 minutes** after ingestion of the lactulose:mannitol test solution and total urine is collected for five hours, during which time the caregiver should offer children liquids frequently in order to permit collection of an adequate volume of urine. Total urine volume is measured and recorded and a sample of five ml is stored in a -80 °C freezer until lactulose and mannitol concentration is determined by HPLC employing pulsed amperometric detection (PAD). Most sites will send these urine samples to either Dr. Aldo Lima, Fortaleza, Brazil or to Dr. Zulfi Bhutta, AKU, Karachi, Pakistan for centralized testing of L/M. A 2 ml sample of urine (without chlorhexidine) is placed in a cryotube and frozen at each time point (iodine testing will be conducted on the 6 and 15 month specimens, however the chlorhexidine-free specimen may be useful for future assays). Children in the BMMI cohort should have an **additional** 2ml chlorhexidine-free sample of urine collected.

1. **Material**

Oral L:M Solution (**see SOP for method of preparation and conduct of the LM test**)

2 Urine collection jars/cups (graduated 100 mL)

2 Pediatric urine collection bags (Fisher cat. #22275347 or Hollister U-bag #7501)

Chlorhexidine preservative solution in a dropper bottle (2.35% = 40 mg chlorohexidine / ml water) (Sigma Chemical Co., St. Louis, MO)

Pasteur pipets

5-15 ml Test tube

2 ml cryotube (for iodine urine sample)

**if applicable** cryotubes for BMMI (2ml of sample in 4 X 0.5ml aliquots)

2 Coolers with ice packs (1 dedicated food-only for L:M solution & 1 for urine samples)

Min/Max thermometers (Fisher cat. #S90201)

Pre-printed specimen labels

Bath soap and paper towels and cleansing towelettes

Urine Collection Form (UCF) NOTE this form has been updated to include questions about BMMI collection.

1. **Methods**

A. 1 week prior to L:M testing:

Data Management team notifies FWs and Pharmacy of subjects to be L:M tested. The FWs are given a list of children and the Pharmacy is given a list of PID numbers with their corresponding weights. The Pharmacy prepares and stores the L:M solution vials in a food-only refrigerator for the FWs to take on the morning of the testing.

B. Day before testing:

24 hours before the test is to be performed on the study child, the family is informed that the subject is not to eat or drink for a mimimum of 2 hours (ie. no eating or drinking after sunrise) before the test is to be performed.

C. Morning of testing:

NOTE**** As of May 20, 2011, we are asking sites to record the number of **loose** (not total) stools passed during the 5 hour urine collection period on the UCF because it has been observed that there is an association between higher zscores and loose stool production (ie well nourished children show increased development of loose stools during testing).

1. The L:M solution is kept refrigerated until the day of the study. The L:M solution is transported by FWs from the laboratory to the field in a cooler with a thermometer and ice pack (L:M solution vials may be stored refrigerated at a Field office, if one is available).
2. The L:M assay is performed on urine samples collected during the monthly surveillance visits conducted at 3, 6, 9, and 15 months of age. Fieldworkers should make every effort to collect the urine sample during the window allowed for that visit (DOB +/- 2 days). However, to allow for problems encountered in administration of the L:M solution or collection of the urine sample, repeat collection can be attempted during an extended window **for this assay only**. This window is defined as DOB -2/+7 days (a total of 10 days) at the 3, 6, 9, and 15 month visits. If a sample is obtained outside of this 10 day window, a protocol deviation form must be completed.
3. Early in the morning of testing (ie. 2 hours after sunrise), the FW places the L:M solution into a dedicated food-only cooler containing an ice pack and carries the cooler to each subject’s home.
4. FW describes the procedure to the mother/guardian and verifies that the child has not had any food or drink for at least 2 hours (ie. since sunrise).
5. FW attaches a pediatric urine collection bag to the infant/child (see following Urine Collection Bag SOP)
6. After bag is attached, the FW gives the L:M solution to the subject to drink by pouring the solution into a drinking or medicine cup. Dose is 2 ml/Kg of weight to a maximum 20 ml. Record time of completed ingestion on the Urine Collection Form in the Time of Injestion field.
7. Thirty (30) minutes after the complete ingestion of the LM solution the family is instructed to resume regular feedings (i.e. breastfeeding and/or breast milk alternated with other milk, water and other weaning foods).
8. For 5 hours, the FW stays near the child’s home and collects the urine in a urine collection bag. FW may move between 2-3 houses to perform testing of 2-3 children on the same morning.
9. After 2 hours or if the bag is filled, the bag is replaced with a new bag and the urine is poured into a labeled graduated jar/cup (jar placed into a ziplock baggie and kept cool in a BSL2 labeled cooler with ice). At the 6 and 15 month testing, a 2 ml sample is pipeted from the urine jar/cup and place into a labeled cryotube for iodine testing.

NOTE 1: Sites may wish to collect and archive additional 2ml chlorhexidine-free urine specimens at each time point to be used in future assays as determined by the Gut Function Committee.

NOTE 2: For those enrolled in the BMMI project, at 3, 6, 9, and 15 months, you MUST collect an additional 2 ml chlorhexidine-free sample by pipetting from the urine jar/cup and placing into 4 labeled cryotubes for freezing and subsequent shipment to Washington University. After performing the additional collection, complete the BMMI specific questions on the UCF form.

1. After removing the chlorhexidine-free sample for iodine and other testing , for every 50mL of urine remaining in the graduated jar/cup, 1-2 drops of 2.35% chlorhexidine is added as a preservative.
2. After the 5 hours +/-15 minutes, the urine collection is complete and the urine collection bag is removed and the urine added to the 2-hour sample. Additional drops of chlorohexidine are added as needed (1-2 drops for every 50 ml of additional urine).
3. The time of completion is recorded on the Urine Collection Form in the End Time field and the final volume of the collected urine is noted in the Total Volume field.

Note: 2 ml is added to the total volume to account for the removal of 2 ml for iodine and other testing. If the child is in the BMMI cohort, an additional 2ml should be added to account for the removal of the BMMI sample

1. Enter the date of collection (DD/MMM/YY) and time of collection (hh:mm; 24 hour time scale) On the pre-printed labels, circle the U indicating that the sample is a urine sample. Please also indicate by circling if the urine sample is for iodine testing (I), or BMMI (BM). or laculose:mannitol (L:M). The Sample ID will be entered once it is assigned in the laboratory.
2. The FW places all of the urine samples (iodine (chlorhexidine-free), BMMI if applicable (chlorhexidine-free), and L/M) into a ziplock bag containing absorbent toweling. All specimens are refrigerated (or kept cold in a cooler with a frozen ice pack) until transported to the research laboratory for analysis. For transport, urines are placed into a plastic cooler (labeled Biohazardous, BSL#2) containing a frozen ice pack. The cooler is securely sealed with tape and sent to the Laboratory, preferrably on the same day as collection.
3. At the time of arrival at the research laboratory, the Sample ID is assigned to the urine specimens. About 5-15 ml of urine with added chlorohexidine is transferred to another pre labeled (with subject’s PID, and Lactulose:Mannitol) sample mailing tube and sealed with parfilm. Enter the date and time of collection on the label. The remaining urine is discarded. Both aliquots (with and without chlorhexidine) are labeled with the same Sample ID. The lab technician should write the Sample ID on the existing labels.
4. Urine specimens should be stored at -80C until shipping or processing. BMMI samples will be shipped to Washington University.
5. Collection information is completed on the Urine Collection Form (UCF) and are placed in drawer #A-1 in the data management room for double entry into the database.
6. **Documentation**

All collections are noted on the Urine Collection and Receiving Form (UCF) as mentioned above. Please ensure that the samples are labeled clearly (for Iodine or for Lactulose:Mannitol). Any notes about spillage or other required information should be noted in the Observations field. Any problems during aliquoting or transport should be noted in the lower Observations field.

| # | Question | Guidance |
| --- | --- | --- |
|  | Participant ID | Write the child’s participant ID in the space provided at the upper left corner. |
|  | **Urine collection** | |
| 01 | Fieldworker ID | Enter the fieldworker’s unique ID number who collected the urine sample. |
| 02 | Date | Enter the date of collection (DD/MMM/YY) of the urine sample. Should be collected during the monthly surveillance visit within the window DOB -2/+7 days. |
| 03 | Start time (solution ingestion completed) | Record the time of completed ingestion of the lactulose:mannitol solution by the study subject. Enter the time using the 24 hour time scale (HH:MM). |
| 04 | End time of urine collection | Record the time of completed urine collection. Enter the time using the 24 hour time scale (HH:MM). |
| 05 | Total Urine Volume | Measure the volume of collected urine in a graduated container. Record the total volume in mL. |
| 06 | Time of departure from field | Enter the time of departure from the field (either the site of urine collection or the field laboratory if applicable). Enter the time using the 24 hour time scale (HH:MM). |
| **B.** | **Urine transport and receiving** | |
| 07 | Lab Technician ID | Enter the Lab Technician’s unique ID number who processes or receives the urine sample, if different from the FW who collected the sample. If not applicable, strike through the first box and enter NA. |
| 08 | Was a 2 mL aliquot of urine without chlorhexidine obtained | Indicate whether an aliquot of urine without chlorhexidine was obtained. Possible answers: 00= No, 01= Yes. |
| 09 | Was a 5-15 mL aliquot of urine with chlorhexidine obtained | Indicate whether an aliquot of urine with chlorhexidine was obtained. Possible answers: 00= No, 01= Yes. |
| 10 | Time of arrival at research lab | Enter the time of arrival at the Research Laboratory. Enter the time using the 24 hour time scale (HH:MM). |
| 11 | Sample ID | Write the 9 digit Sample ID. |
| 12 | How many loose stools were passed during the 5 hr urine collection? | Record the number of loose stools passed during the collection period. Range (00-15; NA allowed for sites not wanting to collect this information.) |
|  | Observations | Note any spillage, information, or problems |

NOTE: This section added April 2012.

This section is to be completed by sites participating in the BMMI project (BR, BG, IN, PE, & SA only)

| **BMMI urine collection** | | |
| --- | --- | --- |
| 13 | Is this child in the BMMI cohort? | Indicate whether the child is in the BMMI cohort.  Possible answers: Yes=01, No=00  (IF NO, THE FORM IS COMPLETE) |
| 14 | Was urine without chlorhexidine obtained for BMMI? | Yes=01, No=00 |
| 15 | Volume of urine collected for BMMI (ml) | #.# (maximum of 2ml) |
| **BMMI urine receiving** | | |
| 16 | Number of aliquots for BMMI  (maximum of 0.5ml per tube) | Range (00-04) |

**VII. Verification**

The study agent ingestion is always directly observed by the trained Study Researcher / Nurse / Fieldworker. When informed by the data staff, FW will review any form that requires revision or clarification. These forms will be kept in the data office.

Use of Urine Collection Bag

1. **Purpose**

The urine collection bag is used for collection of urine from infants and young children. Most often the bags are used in conjunction with the Lactulose:Mannitol assay (see Lactulose Mannitol Assay SOP) and iodine micronutrient assay.

1. **Material**

Pediatric urine collection bags (Fisher cat. #22275347) or Hollister U-bag #7501)

Pre-printed labels

**III. Methods** – see below diagram

- Wash hands before attaching the bag.
- Cleanse the infant’s genital area with bath soap, rinse, dry and wipe with cleasing towelette.
- Allow the newly cleansed area to air-dry. DO NOT wipe over the area to speed drying.
- Firmly attach the pediatric urine collection bag to the child’s genital area.

### USV—Small Volume Urine Collection, Processing and Transport SOP

1. **Purpose**

As part of the MAL-ED Extension protocol, a small volume of urine (chlorhexidine free) will be collected from EVERY enrolled child at 24 and 36 months of age in order to determine iodine concentration. The small volumes collections will NOT be preceded by Lactulose Mannitol ingestion and will be relatively simple to obtain.

Children enrolled in the BMMI project will provide a small volume of urine (chlorhexidine free) at 12, 18, 21, and 24 months of age for archiving at -80C and subsequent shipment to Washington University in St. Louis, USA.

At 12, 18, 21, and 24 months, a 2 ml sample of chlorhexidine-free urine will be placed in 4 x 0.05ml aliquots in cryotubes and frozen at -80C for those enrolled in the BMMI project. At 24 and 36 months a 2 ml sample of chlorhexidine-free urine will be placed in a cryotube and frozen for those enrolled in the MAL-ED cohort project. **Please note that at 24 months of age, those enrolled in the BMMI project will need to collect a total of 4 ml in order to fulfill both the MAL-ED cohort and BMMI requriements.**

1. **Material**

Urine collection jars/cups (graduated 100 mL)

Pediatric urine collection bags (Fisher cat. #22275347 or Hollister U-bag #7501)

Pasteur pipets

2 ml cryotube (for iodine urine sample)

cryotubes for BMMI if applicable (4 tubes of 0.5ml of urine each)

2 Coolers with ice packs (1 dedicated food-only for L:M solution & 1 for urine samples)

Min/Max thermometers (Fisher cat. #S90201)

Pre-printed specimen labels

Bath soap and paper towels and cleansing towelettes

Small Volume Urine Collection Form (USV)

1. **Methods**
   - - 1. At 12, 18, and 21 months of age, for BMMI participants only, FW attaches a pediatric urine collection bag to the infant/child following the SOP for cleaning the urogenital area and attaching the bag (see prior Urine Collection Bag SOP).
       2. As soon as the bag has been filled with at least 2 ml of urine, the bag is removed and urine is collected by pipet and transferred to a labeled cryotube for archiving and subsequent shipment to Washington University. This sample is chorhexidine-free.
       3. For all children in MAL-ED, at 24 and 36 months of age, depending on the ability of the child, the FW may choose to follow the Urine Collection Bag SOP or may ask the child to urinate on command. Should the FW choose to use the collection bag, the same procedure as in steps 1 and 2 are followed for all MAL-ED children.

**Note at 24 months that children enrolled in BMMI will need to collect a total of at least 4 ml in order to aliquot into cryotubes for both iodine testing and shipment of 2 ml to Washington University.**

- - - 1. Should the FW choose to let the child urinate on command, the FW should wash her hands first, cleanse the infant’s genital area with bath soap, rinse, dry and wipe with cleasing towelette, and allow the newly cleansed area to air-dry. DO NOT wipe over the area to speed drying. Once the cleanse and dry is performed, the child should urinate into a collection cup. The contents of the cup should be transferred via pipet to appropriately labeled cryotubes.

1. Urine collected for BMMI should be divided into 4 x 0.5ml aliquots in cryotubes.
2. Enter the date of collection (DD/MMM/YY) and time of collection (hh:mm; 24 hour time scale) On the pre-printed labels, circle the U indicating that the sample is a urine sample. Please also indicate by circling if the urine sample is for iodine testing (I) or BMMI (BM). The Sample ID will be entered once it is assigned in the laboratory.
3. The FW places the urine sample(s) into a ziplock bag containing absorbent toweling. All specimens are refrigerated (or kept cold in a cooler with a frozen ice pack) until transported to the research laboratory for analysis. For transport, urines are placed into a plastic cooler (labeled Biohazardous, BSL#2) containing a frozen ice pack. The cooler is securely sealed with tape and sent to the Laboratory, preferrably on the same day as collection.
[truncated: 516,292 more chars]
