# Supplementary material for: Childhood stunting in relation to the pre- and postnatal environment during the first 2 years of life: The MAL-ED longitudinal birth cohort study
Source: PLoS Med. 2017 Oct 25;14(10):e1002408. doi: 10.1371/journal.pmed.1002408 (PMC5656304; doi:10.1371/journal.pmed.1002408)
Supplement: S2 Text — (DOCX) [file pmed.1002408.s014.docx]

**S2 Text. Sample size**

The enrollment goal for the study was 200 children per site. While this sample size was established as the maximum number of children who could be followed intensively at each site, tested the power of the study for a single site and all sites combined to detect an association between the rates of any given enteropathogen infection and incident malnutrition outcomes at two years age. We started with a set of assumptions based on a recent birth cohort study in Bangladesh:

- 200 children will be enrolled and followed for two years at eight independent sites (1600 total children) from birth to two years of age;
- 40% would become malnourished (weight-for-age Z-score < -2 or height-for-age Z-score < -2) between birth and two years of age;
- 50% of children have diarrhea due to enterotoxigenic *E. coli* (ETEC) over the two year study;
- Infection with ETEC increases the risk of malnutrition by 50%.

Specifically, we tested the power of the study a priori under varying rates of enteropathogen detection, proportions of incident malnutrition outcomes at 24 months, and effect sizes. Under the assumption that 8% of children would be malnourished at birth, the effective sample size was estimated at 184 children per site, or 1472 children for all sites. Therefore, if 40% of the children became malnourished (i.e., a weight-for-age Z-score < -2 or height-for-age Z-score < -2) at the end of two years and if an enteropathogen infection (eg., ETEC) affected 50% of the children over two years, we would have 60% power for a single site and 100% power for all sites combined to detect a 50% higher risk of being malnourished with 95% confidence. The power of the study to detect a 70% increase in malnutrition risk associated with an infection (all other assumptions being the same) was 82% for a single site and 100% for all sites combined.
