# Supplementary material for: Childhood stunting in relation to the pre- and postnatal environment during the first 2 years of life: The MAL-ED longitudinal birth cohort study
Source: PLoS Med. 2017 Oct 25;14(10):e1002408. doi: 10.1371/journal.pmed.1002408 (PMC5656304; doi:10.1371/journal.pmed.1002408)
Supplement: S3 Text — (DOCX) [file pmed.1002408.s015.docx]

**S3 Text. Analytical sample**

We included the data of children who met the following minimal criteria for longitudinal follow-up:

1. Children with baseline anthropometry and with at least one weight and length measurement in each time period (0-2 months, 3-5 months, 6-8 months, 9-11months, 12-17 months, and 18-24 months);
2. Last anthropometry at ≥22 months;
3. Children with at least one fully tested surveillance stool in each time period (0-2 months, 3-5 months, 6-8 months, 9-11 months, 12-17 months, and 18-24 months),
4. Children with at least one quantitative complementary diet recall in each time period (9-11 months, 12-17 months, and 18-24 months).

In addition, there were additional missing data with the largest fractions being biomarkers for gut dysfunction, nutrition, and inflammation (Figure S1). In Table S4, we show a comparison of key variables between children included in the analytical dataset and those who were excluded. We used t-tests with a Bonferroni correction for multiple comparisons given that we had 7 countries.

For this analysis, we excluded data from Pakistan because quality assurance procedures identified unexplained bias in a subset of length measurements. In speaking with anthropometric experts (with more years of experience than the significant experience among MAL-ED investigators), it is clear that the error pattern is highly unusual. Although we had procedures in place to identify extreme values for re-measurement, our procedures did not pick up problems in length measurements for several possible reasons:

1. Our focus for quality assurance was on detecting extreme values or obvious errors (e.g., child losing length) for re-measurement and to assess reliability of the data on approximately 5% of all values, rather than measurement bias *per se.*
2. There were issues with some length boards in Pakistan which led to bias in the measures, and it is likely (but not documentable as we did not identify the length boards used for each child/field worker) that the same length boards were used for the re-measurements, which may have reduced the likelihood of bias detection
3. Although some digit preference was ultimately detected, it was a minor source of bias.
4. The problem was not identifiable at the beginning of the study, suggesting that training and equipment were adequate.
5. Our quality assurance focus was on errors in the anthropometric measurements – the problem became apparent over time as a clustering or tightening of the length-for-age z-score distributions by child age, and more so by month of the study.
6. Data in Pakistan were collected in a sample of a few hundred children living in a rural community that had never been studied before by our colleagues. Moreover, this community has a considerable marriage among relatives, which might lead one to think that either we do not know how this sample of children might grow over time, and the sample might exhibit reduced variance in length as children age compared with WHO growth standards.
