# Supplementary material for: Childhood stunting in relation to the pre- and postnatal environment during the first 2 years of life: The MAL-ED longitudinal birth cohort study
Source: PLoS Med. 2017 Oct 25;14(10):e1002408. doi: 10.1371/journal.pmed.1002408 (PMC5656304; doi:10.1371/journal.pmed.1002408)
Supplement: S4 Text — (DOCX) [file pmed.1002408.s016.docx]

**S4 Text. Model specification**

The longitudinal, multiple variable, ordinal logistic regression model was specified as follows:

$$logit\left[ \Pr\left( Y_{ij}\geq y \right) \right]=\beta_{1}+\beta_{2}+B_{i}\left( \boldsymbol{X;\tau} \right)+T_{ij}\left( t_{ij}\boldsymbol{; \alpha} \right)+M_{ij}\left( t_{ij}\boldsymbol{,X;\lambda,\delta} \right)+\varepsilon_{ij}$$

$$B\left( \boldsymbol{X;\tau} \right)=\sum_{k=1}^{29} \tau_{i}X_{i,k}$$

$$T_{ij}\left( t_{ij}\boldsymbol{;\alpha} \right)=\alpha_{1}t_{ij}+\alpha_{2}\left( t_{ij}-6 \right)_{+}+\alpha_{3}\left( t_{ij}-12 \right)_{+}+\alpha_{4}\left( t_{ij}-18 \right)_{+}$$

$$M_{ij}\left( t_{ij}\boldsymbol{,X;\lambda,\delta} \right)=\sum_{p=1}^{p=27} \left[ \lambda_{1,p}t_{ij}X_{i,p}\left( t_{ij} \right)+\lambda_{2,p}\left( t_{ij}-6 \right)_{+}X_{i,p}\left( t_{ij} \right)+\lambda_{3,p}\left( t_{ij}-12 \right)_{+}X_{i,p}\left( t_{ij} \right)+\lambda_{4,p}\left( t_{ij}-18 \right)_{+}X_{i,p}\left( t_{ij} \right) \right]+\delta_{1}I\left( t_{ij}>9 \right)X_{i,28}\left( t_{ij} \right)+\delta_{2}I\left( t_{ij}>9 \right)X_{i,29}\left( t_{ij} \right)$$

In this model, $Y_{ij}$ was coded 0, 1, or 2 for -1 SD or greater (reference); between -1 SD and -2 SD, and less than -2 SD, respectively, at age $t_{ij}$ months for the $i^{th}$ child; and $y$ was either 1 or 2. The coefficient vector $\boldsymbol{\tau}\mathrm{in}B_{i}$ was the log cumulative odds for each risk factor at enrolment ($\boldsymbol{X}$) including indicators for site and sex. $T_{ij}$ was the log cumulative odds at age $t_{ij}$ modeled as a linear piecewise spline with knots at 6, 12, and 18 months, and $M_{ij}$ was the interaction between components of the linear piecewise splines and multiple risk factors ($\boldsymbol{X}$) including sex and site, and between and indicator variable for $t_{ij}$ > 9 months and nutrient intake variables collected after 9 months. We included a total of 22 risk factors in addition to site (6 variables) and sex. The variable $t_{ij}$ represented the $j^{th}$ measurement of age for the $i^{th}$ child. Model coefficients were represented by $\boldsymbol{\alpha,}\boldsymbol{\beta,\tau}\boldsymbol{, \lambda}, \text{and} \boldsymbol{\delta}$; and, $\boldsymbol{\varepsilon}$ were the terms of the error structure. For this analysis, we used a marginal (population-averaged) approach to account for clustering by child. Specifically, we used the Huber-White method to correct the variance-covariance matrix for correlated responses of repeated measurements from child clusters. Because there were >1000 clusters of children, robust variances give a consistent estimate of the variance-covariance matrix despite mis-specification of the underlying model. An important assumption for an ordinal logistic regression model was that effect sizes were the same for every change in cumulative categories (ie, the increase in the cumulative odds for risk factor $X_{.k}$ was the same between the groups of children with length-for-age ≥ -2 when compared to length-for-age < -2, and groups of children with length-for-age ≥ -1 when compared to length-for-age < -1 at age $t$months).
