# Supplementary material for: Childhood stunting in relation to the pre- and postnatal environment during the first 2 years of life: The MAL-ED longitudinal birth cohort study
Source: PLoS Med. 2017 Oct 25;14(10):e1002408. doi: 10.1371/journal.pmed.1002408 (PMC5656304; doi:10.1371/journal.pmed.1002408)
Supplement: S5 Text — (DOCX) [file pmed.1002408.s017.docx]

**S5 Text. Testing the proportionality of odds assumption and goodness of fit**

To graphically assess for proportionality of odds, we compared the ORs and corresponding 95% confidence intervals for each variable obtained from a logistic regression model evaluated at each of the threshold points in length-for-age (-1 SD and -2 SD, respectively). There was substantial overlap in the 95% confidence intervals for each variable between threshold points, and as observed in the below figure, departures from proportionality did not substantively impact the relative importance or factor-specific effects (Figure S4).

To assess goodness-of-fit, we visually assessed overlap between observed and model-predicted probabilities of length-for-age categories for children stratified by site. We calculated two times standard error bands for the observed probabilities using standard methods, and 90%, 95%, and 99% prediction error bands for the predicted probabilities will be obtained using 2000 bootstrap replications in which children within site are chosen with replacement, but the original numbers of children within site were maintained. We noted an overall good fit between observed and model-predicted probabilities, with the exception of SAV for which the model overestimated observed probabilities in the length-for-age category between less than -1 and -2 (Figure S5).
