# Supplementary material for: Childhood stunting in relation to the pre- and postnatal environment during the first 2 years of life: The MAL-ED longitudinal birth cohort study
Source: PLoS Med. 2017 Oct 25;14(10):e1002408. doi: 10.1371/journal.pmed.1002408 (PMC5656304; doi:10.1371/journal.pmed.1002408)
Supplement: S6 Text — (DOCX) [file pmed.1002408.s018.docx]

**S6 Text. Proposed analytical plan.**

**Hypothesis to be explored**

Identify risk factors for stunting using pooled data of the MAL-ED study during the first 24 months of life: Birth weight, maternal height, breastfeeding, child feeding practices and dietary intake, WAMI index (Water/sanitation, Assets, Maternal education, household Income), food security status, infectious disease morbidity variables, micronutrient status, and biomarkers of enteropathy.

**Variables of interest**

Independent variable: Stunting status at 12 and 24 months

Dependent variables: Birth weight, maternal height, breastfeeding, child feeding practices and dietary intake, WAMI index (Water/sanitation, Assets, Maternal education, household Income), food security status, infectious disease morbidity and antibiotic use, micronutrient status, child sex, and biomarkers of enteropathy.

**Statistical analysis plan and methods**

To identify risk factors for of stunting at 12 and 24 months of age in children enrolled in the MAL-ED cohort. We will use ordinal logistic regression with length-for-age stratified into three categories (< -2, -2 to -1, ≥ -1) and perform a multivariable analysis using all factors described above. For adjustment for clustering we may use a generalized estimating equation (GEE) approach or other robust methods for clustering. We may consider to include site as a fixed effect. We may also consider other approaches to model stunting. These include logistic regression, time-to-event models using Cox-proportional hazards or other parametric models like the Weibull, Gamma or Generalized Gamma distributions. Different modelling approaches may provide different insights into the relationship between stunting and risk factors.
